# CYP27B1 Downregulation: A New Molecular Mechanism Regulating EZH2 in Ovarian Cancer Tumorigenicity

**DOI:** 10.3389/fcell.2020.561804

**Published:** 2020-10-14

**Authors:** Xiao Huo, Hengzi Sun, Qiuhong Qian, Xiangwen Ma, Peng Peng, Mei Yu, Ying Zhang, Jiaxin Yang, Dongyan Cao, Ting Gui, Keng Shen

**Affiliations:** ^1^Department of Obstetrics and Gynecology, Peking Union Medical College Hospital, Chinese Academy of Medical Sciences and Peking Union Medical College, Beijing, China; ^2^Department of Obstetrics and Gynecology, Beijing Chao-Yang Hospital, Capital Medical University, Beijing, China; ^3^Department of Obstetrics and Gynecology, Qilu Hospital, Shan Dong University, Jinan, China

**Keywords:** ovarian cancer, EZH2, ChIP-seq, steroid biosynthesis, CYP27B1

## Abstract

**Background:**

Ovarian cancer has the highest mortality rate among gynecologic cancers, and most patients are diagnosed in advanced stages. Enhancer of zeste homolog 2 (EZH2) is a major tumor marker and an effective therapeutic target for ovarian cancer, but the underlying molecular mechanism remains unclear. The present study investigated the biological effects of EZH2 knockout in SKOV3 cells *in vitro* and *in vivo* and explored the molecular mechanism by integrated analysis of messenger RNA sequencing (mRNA-seq) and chromatin immunoprecipitation sequencing (ChIP-seq) data.

**Methods:**

The CRISPR/Cas9 system was used to establish EZH2 knockout SKOV3 cells. Protein expression was evaluated by Western blotting. The effect of EZH2 on ovarian cancer was evaluated *in vitro* with MTT, wound healing, Transwell, and apoptosis assays and *in vivo* with a xenograft model. mRNA-seq and ChIP-seq were performed to explore the molecular mechanism underlying the biological function of EZH2. Immunohistochemical staining (IHC) of tissue arrays was used to analyze the correlations among EZH2 and CYP27B1 expressions and prognosis.

**Results:**

We obtained three EZH2 knockout subclones. EZH2 knockout SKOV3 cells exhibited significantly suppressed proliferation, migration, and invasion and a significantly increased apoptosis rate. The subcutaneous tumor formation rate decreased from 100 to 0% in the EZH2 knockout group. Integrated analysis of the mRNA-seq and ChIP-seq data identified 1,455 significantly upregulated genes with matching downregulated trimethylation of histone H3 lysine 27 (H3K27me3) methylation binding sites in 1b11H cells compared to SKOV3 cells. The set of downregulated genes in EZH2 knockout cells was highly enriched in genes regulating the activation of steroid biosynthesis; the top-ranked hub gene was CYP27B1. The EZH2 and CYP27B1 expression levels showed a statistically significant inverse correlation, which was also associated with unfavorable prognosis. The *in vitro* experiment demonstrated that CYP27B1 can suppress the proliferation, migration, and invasion of ovarian cancer cells. Moreover, the levels of AKT and p-AKT were significantly increased, whereas STAT3 was downregulated, in 1b11H cells compared to SKOV3 cells. Moreover, STAT3 and AKT overexpression was observed in 1b11H siRNA for CYP27B1 (siCYP27B1) cells.

**Conclusion:**

EZH2 plays an important role in promoting cell proliferation, migration, and invasion in ovarian cancer by regulating the core steroid biosynthesis gene *via* H3K27me3 methylation. Moreover, CYP27B1, the steroid biosynthesis hub gene, might be a novel therapeutic target for ovarian cancer.

## Introduction

Ovarian cancer has the highest mortality rate among gynecologic cancers, and most patients are diagnosed in advanced stages. Based on the Global Cancer Incidence, Mortality and Prevalence (GLOBOCAN) estimates, ∼295,414 new ovarian cancer cases and 184,799 deaths occurred worldwide in 2018 (Bray et al., 2018). Many patients still experience relapse even if treated with satisfactory cytoreductive surgery (CRS) combined with standard platinum-based chemotherapy. The 5-year survival rate for patients with advanced ovarian cancer is ∼22–30% (Siegel et al., 2019). The search for appropriate tumor markers and effective therapeutic targets for ovarian cancer has been a major research focus.

Enhancer of zeste homolog 2 (EZH2) is a histone methyltransferase that catalyzes the trimethylation of histone H3 lysine 27 (H3K27me3) (Zhang et al., 2014). As a core member of the Polycomb group (PcG) protein family, EZH2 plays an important role in stem cell maintenance and tumor development (Chang and Hung, 2012) and is overexpressed in various cancers (Varambally et al., 2002; Kleer et al., 2003; Bachmann et al., 2006; Saramaki et al., 2006; Bryant et al., 2007). In our previous study, we found significantly elevated expression levels of EZH2 in lymph node metastases and recurrent tumors compared with primary ovarian tumors. In addition, intense expression of EZH2 in recurrent tumors was significantly associated with shortened overall survival (OS) times (Gui et al., 2014). However, the biological effects and molecular mechanism of EZH2 in ovarian cancer are still unclear. Thus, investigating the functions and potential mechanisms of EZH2 may provide important insights into the treatment of ovarian cancer.

In the present study, we knocked out EZH2 in ovarian cancer cells *via* the clustered regularly interspaced short palindromic repeats (CRISPR)/Cas9 system to explore its biological effects *in vitro* and *in vivo*. In addition, we investigated the molecular mechanism, which centers around CYP27B1, according to integrated analysis of the messenger RNA sequencing (mRNA-seq) and chromatin immunoprecipitation sequencing (ChIP-seq) data. CYP27B1 is a member of the cytochrome P450 superfamily of enzymes which catalyze drug metabolism and the synthesis of steroids, cholesterol, and other lipids. Studies have demonstrated that CYP27B1 regulates the level of biologically active vitamin D and plays an important role in calcium homeostasis. More importantly, CYP27B1 also plays an important role in various types of malignant tumors. Brożyna et al. (2013) reported an inverse correlation between CYP27B1 and Ki-67 expression. Moreover, a lower CYP27B1 expression was related to worse prognosis in patients with melanoma, indicating that CYP27B1 plays an important role in melanoma pathogenesis and progression. The data from another study, which focused on non-melanoma skin cancer (NMSC), showed that the expression of CYP27B1 was diminished in NMSC tissue compared to normal skin (Nemazannikova et al., 2019). Studies have also demonstrated that a polymorphism of CYP27B1 (rs10877012) is significantly associated with an increased risk of colorectal cancer, according to gene sequencing of relevant peripheral blood samples (Vidigal et al., 2017). In addition, in the polyoma middle T-antigen mouse mammary tumor virus (PyMT-MMTV) breast cancer model in mice, mammary tumorigenesis was found to be significantly accelerated after targeted ablation of CYP27B1.

## Materials and Methods

### Cell Culture

The human epithelial ovarian cancer cell line SKOV3 was obtained from the Institute of Basic Medical Sciences, Chinese Academy of Medical Sciences & School of Basic Medicine, Peking Union Medical College. SKOV3 cells were cultured in McCoy’s 5A medium supplemented with 10% fetal bovine serum (FBS; United States), 100 μg/ml streptomycin, and 100 U/ml penicillin. All cells were incubated in a humidified atmosphere containing 5% CO_2_ at 37°C.

### CRISPR/Cas9 Vector Construction and Transfection

Guide RNA (gRNA) target sequences were selected as 20-nt sequences preceding an NGG protospacer adjacent motif (PAM) sequence in the genome. Two single-guide RNA (sgRNA) sequences targeting the coding sequence of the EZH2 exon were selected using the online CRISPR design tool^1^ from the Zhang Laboratory (Cong and Zhang, 2015) at the Broad Institute, and the selected gRNA sequence was predicted to have a very low probability of complementarity to off-target sites. The two sgRNAs were hybridized with their reverse and complementary oligonucleotide. A CACCG motif was added upstream of the forward sequence, and an AAAC motif was added upstream of the reverse sequence to increase the specificity of the gRNA. Plasmid pX330 was used for the construction of the CRISPR/Cas9-gRNA expression vector, which contains a restriction enzyme (*Bbs*I) site and expresses Cas9. The hybrid constructs were then inserted into the *Bbs*I site of the pX330 plasmid after annealing, and the two expression vectors were named pX330-gRNA1 and pX330-gRNA2.

SKOV3 cells were plated in six-well plates (Corning) at a density of 3 × 10^5^ cells/well and cultured overnight to a confluence of 50–80% to obtain maximum transfection efficiency. Cells were co-transfected with 500 ng of pX330-gRNA1 and 500 ng of pX330-gRNA2 as well as 100 ng of pLLexp-puro (a plasmid containing a puromycin resistance cassette) using a FuGENE HD transfection reagent (Roche); cells in one well were left untreated as a negative control. Six hours after transfection, the medium was replaced with complete growth medium. Forty-eight hours after transfection, puromycin (1 μg/ml, Sigma) was added to the culture medium to screen successfully transfected cells, and the medium was replaced every 2 or 3 days. When all cells in the negative control culture were dead, the medium was changed back to complete growth medium and cells were cultured sequentially to 90% confluence for subsequent amplification. The sequences of the gRNAs targeting the EZH2 exon coding sequence were as follows:

EZH2-gRNA1 (sequence 5′–3′): F, CACCGTGCGACT GAGACAGCTCAAG; PAM sequence: AGG; R, AAACCTTGAGCTGTCTCAGTCGCAC.
EZH2-gRNA2 (sequence 5′–3′): F, CACCGTGCGACT GAGACAGCTCAAG; PAM sequence: TGG; R, AAACTTCTTGGTTTAAGATTTCCGC.

### Isolation and Amplification of Single-Cell Subclones

The limiting dilution method was used to obtain single-cell subclones. After transfection, SKOV3 cells were collected, adjusted to a density of 10 cells/ml, and seeded in 96-well plates (100 μl/well). Four 96-well plates were seeded and were named 1a, 1b, 1c, and 1d. The 96-well plates were examined under a microscope. Wells containing a single cell were marked with the plate name, row number, and column number (for example, 1a7B). Wells with two or more masses of cells were excluded. The single-cell clones proliferated to a certain density after culture for 2–3 weeks; then, the clones were successively transferred to 48-, 24-, 12-, and 6-well plates and, finally, to culture dishes. All single cell-derived clones were amplified and backup clones were stored in liquid nitrogen for further validation.

### Genomic DNA Extraction, PCR, and Sanger Sequencing to Detect Sequence Deletion

Genomic DNA was extracted from harvested single-cell subclones with a DNAamp PCR kit (CoWin Biosciences) according to the manufacturer’s instructions. DNA preparations were stored at −20°C and used as templates for polymerase chain reaction (PCR). PCR products were amplified using the primer pairs listed below. A portion of the PCR products was used for agarose gel electrophoresis. PCR products with decreased sizes were purified by gel extraction and then directly subjected to Sanger sequencing.

EZH2 forward primer sequence (5′–3′): CATATTCAG GCTGGTTAGATTAGTG
EZH2 reverse primer sequence (5′–3′): GATCTACAG CAGTCATTAACAGTTG

### Western Blot Analysis

Total protein was extracted from single-cell subclones with RIPA buffer [25 mmol/L Tris–HCl (pH 7.6), 150 mmol/L NaCl, 1% Non-idet-P40, 1% sodium deoxycholate, 0.1% sodium dodecyl sulfate (SDS)] plus a protease inhibitor cocktail (Sigma). The protein concentration was determined by the bicinchoninic acid (BCA) method. Fifty micrograms of total protein from each sample was heated for 5 min at 95°C, separated by 10–12% SDS-polyacrylamide gel electrophoresis (SDS-PAGE), and transferred to polyvinylidene difluoride (PVDF; Millipore) membranes. The PVDF membranes were then blocked with 5% non-fat milk in TBST [20 mM Tris, 150 mM NaCl, 0.2% Tween 20 (pH 7.6)] for 1 h at room temperature and were then incubated with primary antibodies overnight at 4°C. After washing with Tris-buffered saline with Tween (TBST), the PVDF membranes were incubated for 1 h at room temperature with the appropriate secondary antibody. Finally, the PVDF membranes were washed four times for 20 min each with TBST at room temperature. After washing, the PVDF membranes were incubated in reagent from an enhanced chemiluminescence detection kit (Thermo Scientific^TM^) and exposed to X-ray films (Kodak). Each assay was performed in triplicate. Tubulin was used as the loading control (bottom panel). Antibodies specific for the following molecules were used: EZH2, 1:2,000 (CST, United States); β-tubulin, 1:3,000 (CST, United States); and DAPI, 1:2,000 (Sigma, United States).

### Cell Proliferation Assays

Cell proliferation was assessed at 24-h intervals for 7 days using 3-(4,5-dimethylthiazol-2-yl)-2,5-diphenyl tetrazolium bromide (MTT) reagent to detect metabolically active cells (Roche Molecular Biochemicals, United States). The cells were seeded in a 96-well plate at a density of 1 × 10^3^ cells/well and cultured in growth medium. The plate was incubated in a humidified incubator (37°C, 5% CO_2_) for 1–7 days. Four hours before measuring the absorbance, 20 μl of the MTT reagent (5 mg/ml) was added to each well. The absorbance was measured at wavelengths of 570 and 630 nm in a microplate reader (Biotek Instruments, Winooski, United States). Experiments were repeated three times.

### Cell Invasion and Migration Assays

For the invasion and migration assays, the cells were harvested and resuspended in serum-free medium. For the *in vitro* migration assays, 1 × 10^4^ cells were seeded in 24-well cell culture inserts with 8-mm pores (BD Biosciences). For the *in vitro* invasion assays, 1 × 10^5^ cells were seeded in BioCoat Matrigel Invasion Chambers containing BD Falcon Cell Culture Inserts (8-mm pore size) that had been coated with Matrigel matrix (BD Biosciences). In both assays, the lower chambers were filled with 400 μl of McCoy’s 5A medium containing 10% FBS. After a 24-h incubation at 37°C, the cells on the upper side of the membranes were gently removed with wet cotton swabs. The cells on the lower surface of the membranes were fixed with 4% paraformaldehyde for 1 h and were then stained with crystal violet hydrate solution (Sigma-Aldrich, St. Louis, MO, United States) for 30 min. The migratory and invasive potentials of the cells were determined in both the migration and invasion assays by counting the number of cells that migrated to the lower surface of the filter in 12 different areas under an inverted light microscope (Nikon TS 100, Milan, Italy). Each assay was performed in triplicate, and three independent experiments were performed.

Cell migration was also evaluated by a wound healing assay. A confluent cell monolayer was scratched with a pipette tip, rinsed with phosphate-buffered saline (PBS), and cultured in serum-free medium. The wound areas were marked and imaged at different time points with a phase contrast microscope. The width of the wound areas was calculated using ImageJ. The wound healing percentage was calculated as follows: WH% = {[wound area (*n* h) – wound area (0 h)]/[wound area(0 h)]} × 100. Comparisons were performed in GraphPad Prism 5.

### Apoptosis Analysis

An Annexin V-PE/7-AAD Apoptosis Detection Kit (BD Pharmingen) was used for the apoptosis assay according to the manufacturer’s recommendations. In brief, the cells were harvested, washed twice with pre-cooled PBS, and resuspended in 100 μl of 1 × binding buffer. The cells were then incubated with 5 μl of annexin V-PE and 5 μl of 7-aminoactinomycin D (7-AAD) at room temperature for 15 min before cell cycle analysis. The apoptosis distribution was assessed with a FACSCalibur flow cytometer (FCM; BD, United States). The results were analyzed following the manufacturer’s protocol with the flow cytometer. In the flow cytometry scatter plot, the right upper quadrant (annexin V PE + /7-AAD +) indicates late apoptotic cells and necrotic cells, the right lower quadrant (annexin V PE + /7-AAD-) indicates early apoptotic cells, and the left lower quadrant (annexin V PE-/7-AAD-) indicates viable cells. The apoptosis rate was calculated from the number of early apoptotic, late apoptotic, and necrotic cells among all cells.

### Mouse Xenograft Experiment

BALB/c nude mice were purchased from the National Cancer Institute (Bethesda, MD, United States). All procedures for animal care and use were approved by the Institutional Animal Care and Use Committee. Cells (2 × 10^6^) were suspended in PBS and implanted subcutaneously in the flanks of 5-week-old female BALB/c nude mice (*N* = 6 mice/group). Tumor growth was measured twice weekly using a caliper. Palpable tumors of any size were considered to indicate tumor incidence. Mice were monitored for the appearance of signs of disease, such as subcutaneous tumors or weight loss due to potential tumor growth at internal sites. Mice were sacrificed when the tumor diameter reached 2 cm or they exhibited ulceration. The tumor volume was calculated according to the following formula: tumor volume (in cubic millimeters) = (*ab*^2^)/2.

### Cell Immunofluorescence Assay

Cells in the logarithmic phase were collected, centrifuged, and then fixed in 10% formalin. Then, the cells were dehydrated through increasing concentrations of ethanol and placed in a transparent agent, xylene. The cell mass was then placed into a melted paraffin wax and embedded to a cell wax block. The cell wax block was sliced into cell sections for cell immunofluorescence assay. The cell sections were dewaxed in xylene, rehydrated through decreasing concentrations of ethanol, and washed in TBST. Then antigen retrieval buffers (pH 6.0) were used for 15 min and washed in TBST. The cell sections were blocked by 10% goat serum for 30 min and then incubated in primary antibody (anti-EZH2 rabbit antibody; dilution, 1:2,000) overnight at 4°C. Following washing with TBST, the sections were incubated for 45 min at room temperature with a secondary antibody (FITC-labeled goat anti-rabbit IgG; dilution 1:3,000). Tyramide signal amplification (TSA) reagent was used for 15 min and then washed by TBST. Nuclei were stained with DAPI (1:2,000) for 10 min and mounted by 50% glycerol. Images were collected on a Leica SP5 confocal microscope and processed with LASAF software (Leica).

### ChIP-Seq and RNA-Seq

For ChIP-seq, SKOV3 and SKOV3/sgEZH2 cells were fixed and lysed, and chromatin was then sheared into fragments of 200–500 bp using a Bioruptor sonicator (Diagenode). Chromatin was incubated overnight with an anti-H3K27me3 antibody (Millipore 07-622) bound to protein G beads (Thermo Fisher). After immunoprecipitation, the samples were reversely cross-linked in a 65°C water bath and DNA was extracted with a QIAquick PCR Purification Kit (Qiagen). The ChIP-seq libraries were prepared using an NEBNext^®^ Ultra^TM^II DNA Kit (NEB E7645S) and were then sequenced on the Illumina HiSeq 2500 platform. For RNA-seq, SKOV3 and SKOV3/sgEZH2 cells were harvested for RNA extraction using RNeasy Mini Kit (Qiagen). RNA-seq libraries were prepared with a KAPA Stranded mRNA-Seq Kit (Illumina^®^ platform) and were then sequenced on the Illumina HiSeq 4000 platform.

For data analysis, the ChIP-seq raw reads were aligned to the hg38 reference genome using BWA software (version 0.7.2). The resulting SAM files were converted to BAM files with SAMtools (version 0.1.18). MACS2 (version 2.0.10.20131216) was used to call peaks with a cutoff *p*-value of < 0.00001. Peaks were annotated using HOMER’s annotatePeaks.pl tool. The annotations were counted and the distribution was plotted using R (version 3.6.1). HOMER’s (version 4.10) findMotifsGenome.pl tool was used for motif analysis with the peak file and the genome FASTA file as input. The DNA sequence was extracted according to the peak file, and the sequence was compared with the motif database to obtain the motif. ChIP-seq read distributions (from bigwig) across gene bodies are presented as an average plot (average of read signals across all genes), and plotFingerprint analysis was performed with deepTools (version 2.0). Differential gene expression analysis was performed on the RNA-seq data using the DESeq2 algorithm after evaluation of significance and the false discovery rate (FDR) with the following criteria: | log_2_FC| > 1 and FDR < 0.05. Gene Ontology (GO) analysis, Kyoto Encyclopedia of Genes and Genomes (KEGG) pathway analysis, and gene set enrichment analysis (GSEA) were performed as previously described (Ashburner et al., 2000; Draghici et al., 2007).

### siRNA Treatment and Overexpression

The CYP27B1 small interfering RNA (siRNA), CYP27B1 plasmid, and corresponding control constructs were purchased from Shanghai RiboBio. Before transfection, SKOV3 and 1b11H cells (5 × 10^5^) were seeded in a 55-cm^2^ flask. After 6 h, the cell density was ∼30%. The cells were transfected with CYP27B1 siRNA (siCYP27B1), the CYP27B1 expression plasmid, or the corresponding control construct according to the manufacturer’s protocol. Seventy-two hours post-transfection, siCYP27B1- and CYP27B1-overexpressing cells were obtained.

### Immunohistochemical Staining

We collected 160 human epithelial ovarian cancer samples with accompanying patient follow-up information between January 2010 and January 2015 in the Department of Pathology of Peking Union Medical College Hospital. Follow-up was performed until January 1, 2020. Pathological diagnoses were reconfirmed by a pathologist. The project was approved by the Ethics Committee (Peking Union Medical College Hospital), and informed consent was obtained from patients or their family members. The details of immunohistochemical staining (IHC) and scoring were described previously (Li et al., 2010; Zhang et al., 2015). The following antibodies were used: anti-EZH2, 1:200, Abcam ab191080; anti-CYP27B1, 1:500, Abcam ab206655. The intensity of immunostaining was scored as follows: 1 +, weak; 2 +, moderate; 3 +, strong; or 4 +, very strong. The area of positive staining in each microscopic field was scored as follows: 1 +, 0–25%; 2 +, 25–50%; 3 +, 50–75%; or 4 +, 75–100%. A final score between 5 and 80 was obtained by multiplying the product of the two scores by 5. A final score between 0 and 42 was considered “low expression” and a final score between 43 and 80 considered “high expression.” All pathological diagnoses were confirmed in a blinded manner by three expert pathologists.

### Statistical Analysis

Primer Premier 6 was used for primer design and sequence analysis. BioEdit was used for comparison and analysis of sequences. An online CRISPR design tool^2^ was used for gRNA design. The UCSC and NCBI databases were used to obtain gene sequences and coding regions. GraphPad Prism 5.0 was used to construct cell growth curves and tumor growth curves and to perform comparisons between wild-type and EZH2 knockout SKOV3 cells. Each experiment was repeated at least three times. Values are expressed as the mean ± standard deviations (SDs). In histograms, *p < 0.05, **p < 0.01, and ***p < 0.0001. *p* < 0.05 was considered statistically significant.

## Results

### Design of the gRNA Targeting Human EZH2 and Construction of Vectors

**FIGURE 1:**
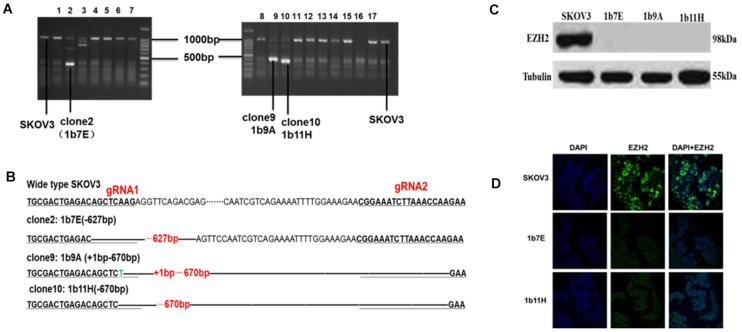
**(A)** Clones with obviously shortened nucleic acid sequences were identified on agarose gels after PCR. **(B)** Changes in target fragments were identified by Sanger sequencing. **(C)** Western blot results. **(D)** Cell immunofluorescence assay results (*green fluorescence* represents EZH2 and *blue fluorescence* represents DAPI).

According to the online CRISPR design tool^3^ from the website of the Zhang Laboratory at the Broad Institute, we selected two gRNA target sequences on exon 1 and exon 2 of human EZH2 (Supplementary Figure S1). The NGG PAM sequence is also shown in Supplementary Table S1. The target sites for the two gRNA sequences were separated by an interval of 650 bp. The two gRNAs were successfully inserted into pX330 vectors downstream of the U6 promoter, as shown in Figure 1B, with an insertion success rate of 100%. The two constructed expression vectors were named pX330-gRNA1 and pX330-gRNA2.

### Knockout of EZH2 in SKOV3 Cells With the CRISPR/Cas9 System

After transfection and drug screening, we obtained 56 subclones by the limiting dilution method. Figure 1 shows the validation results of the EZH2 knockout in SKOV3 cells. The agarose electrophoresis results revealed that three PCR products had obviously shorter nucleic acid sequence lengths in EZH2 knockout cells than in wild-type SKOV3 cells (Figure 1A). The target fragments from these three clones and wild-type SKOV3 cells were further confirmed by Sanger sequencing (Figure 1B). Figure 1B shows that we successfully deleted a 627-bp sequence in clone 2 (1b7E) and a 670-bp sequence in clone 10 (1b11H). In addition, we deleted a 627-bp sequence and added a 1-bp sequence in clone 9 (1b9A). The results of the Western blot experiments further verified that all three clones completely lacked EZH2 protein expression (Figure 1C). We selected two clones for further study. EZH2 knockout in 1b7E and 1b11H cells was also verified by cell immunofluorescence assays (Figure 1D).

### Knockout of EZH2 Reduces Cell Proliferation, Migration, and Invasion and Promotes Apoptosis in SKOV3 Cells

**FIGURE 2:**
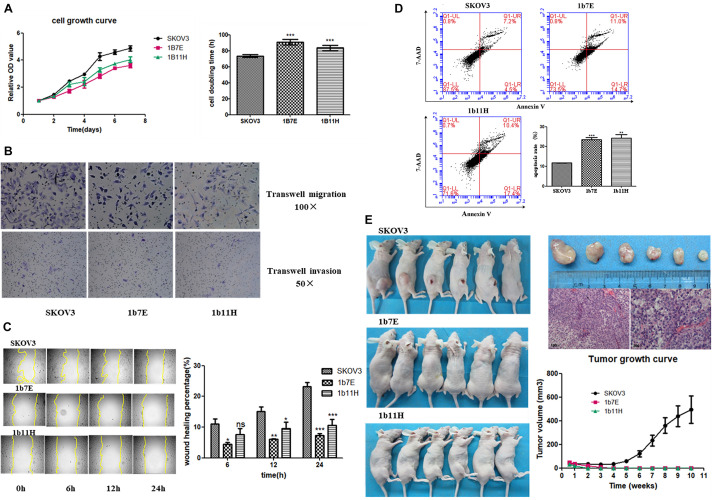
Changes in biological behaviors after knockout of EZH2. **(A)** Cell growth curve and doubling time. **(B)** Transwell migration and invasion assay results. **(C)** Wound healing assay results. **(D)** Flow cytometric analysis results. **(E)** *In vivo* experiment results. ns, *p* > 0.05; *p < 0.05; **p < 0.01; ***p < 0.001.

The results of the cell proliferation assays showed that knockout of EZH2 reduced cell proliferation from the second day through the seventh day after seeding in a 96-well plate compared to that of wild-type SKOV3 cells (Figure 2A). The doubling times for the EZH2 knockout clones compared with SKOV3 were as follows: 1b7E, 90.64 ± 1.55 h vs. 73.57 ± 0.81 h (*p* = 0.0006); 1b11H, 83.69 ± 1.48 h vs. 73.57 ± 0.81 h (*p* = 0.0039) (Figure 2A). Transwell migration and invasion assays showed reduced migration and invasion of 1b7E cells (39.33 ± 6.65 vs. 92.33 ± 6.34, *p* = 0.0006; 13.00 ± 0.82 vs. 27.00 ± 2.45, *p* = 0.0007) and 1b11H cells (31.33 ± 2.62 vs. 92.33 ± 6.34, *p* = 0.0001; 11.67 ± 1.70 vs. 27.00 ± 2.45, *p* = 0.0009) compared with SKOV3 cells (Figure 2B). The wound healing assay results showed that wound closure percentage was increased significantly in wild-type SKOV3 cells compared with 1b7E cells at different time points (6 h, 10.25 ± 2.37% vs. 3.76 ± 0.83%, *p* = 0.011; 12 h, 15.34 ± 1.97% vs. 6.83 ± 0.30%, *p* = 0.0018; 24 h, 24.65 ± 1.68% vs. 7.96 ± 0.77%, *p* < 0.0001) and with 1b11H cells at different time points (12 h, 15.34 ± 1.97% vs. 9.85 ± 2.03%, *p* = 0.028; 24 h, 24.65 ± 1.68% vs. 12.36 ± 1.85%, *p* = 0.001) (Figure 2C). Flow cytometric analysis revealed a higher apoptosis rate in EZH2 knockout SKOV3 cells (1b7E and 1b11H) than in wild-type SKOV3 cells (23.47 ± 1.13% vs. 11.73 ± 0.088%, *p* = 0.0005, and 24.20 ± 1.80% vs. 11.73 ± 0.088%, *p* = 0.0023) (Figure 2D). In addition, *in vivo*, the subcutaneous tumor formation rate in mice implanted with EZH2 knockout clones was obviously reduced (from 100 to 0%) compared to that in mice implanted with wild-type SKOV3 cells (*p* = 0.0021; Figure 2E).

### EZH2 Can Regulate Steroid Biosynthesis by Repressing CYP27B1 Expression via H3K27me3 Methylation

**FIGURE 3:**
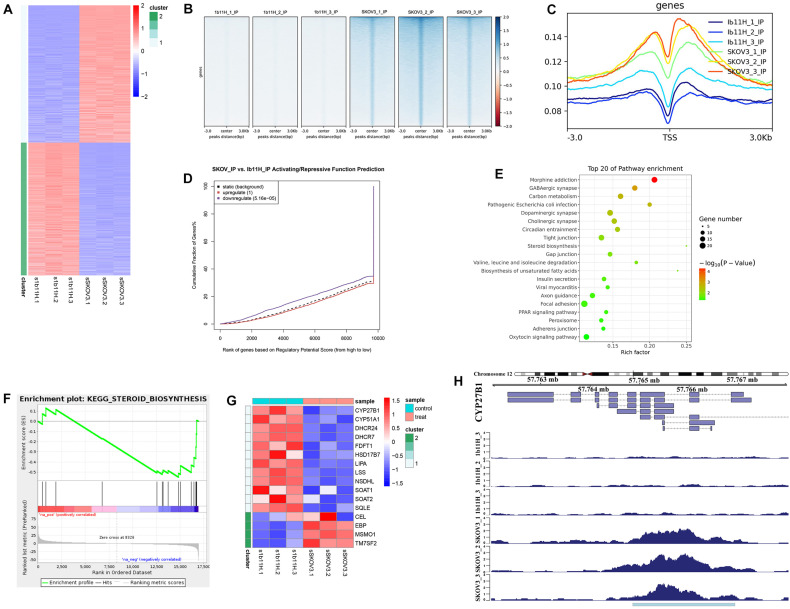
Integrated analysis of RNA sequencing (RNA-seq) and chromatin immunoprecipitation sequencing (ChIP-seq) data. **(A)** Heatmap of the differentially expressed genes identified by RNA-seq. **(B)** Heatmap of the ChIP-seq read distributions within peaks. **(C)** Average read signals across all transcription start sites (TSSs). **(D)** Functional prediction according to integrated analysis of RNA-seq and ChIP-seq data. **(E)** Pathway enrichment of matched downregulated genes. **(F)** Gene set enrichment analysis (GSEA) of matched downregulated genes. **(G)** Heatmap of the differentially expressed genes in steroid biosynthesis. **(H)** ChIP-seq read distributions at the TSS of CYP27B1.

The RNA-seq results showed that 2,333 genes were upregulated and that 2,230 genes were downregulated in 1b11H cells compared to SKOV3 cells (Supplementary Table S2 and Figure 3A). After EZH2 knockout, the number of sites with H3K27me3 was significantly decreased (*p* = 0.003; Figure 3B). Moreover, the number of read signals across the transcription start site (TSS) was also significantly decreased (*p* = 0.0026; Figure 3C). According to the integrated analysis of the RNA-seq and ChIP-seq data, 1,455 genes with matching decreases in H3K27me3-occupied sites were significantly upregulated in 1b11H cells compared to SKOV3 cells (Supplementary Table S3). The combination of the different peaks in the ChIP-seq data and the different genes in the RNA-seq data identified using BETA showed that EZH2 exerted mainly suppressive effects on downstream genes by activating H3K27me3 installation (Figure 3D). To further determine the signaling pathways most strongly impacted by EZH2, we performed KEGG pathway analysis and GSEA on genes downregulated in EZH2 knockout cells. As seen in Figures 3E,F, the set of genes downregulated in EZH2 knockout cells was highly enriched in genes regulating the activation of steroid biosynthesis (the pathway with the highest rich factor), indicating that EZH2 may act to suppress steroid biosynthesis. The GSEA results also revealed that the top-ranked hub gene in steroid biosynthesis was CYP27B1 (Figure 3G). Subsequent analysis focusing on CYP27B1 also showed that the number of H3K27me3-occupied sites on CYP27B1 was significantly decreased after knockout of EZH2 in SKOV3 cells (Figure 3H). The data on SKOV3 + siCYP27B1 have also been added and showed that there is no significant difference in migration, invasion, or proliferation after silencing the CYP27B1 in SKOV3 cells (Supplementary Table S4).

### CYP27B1 Can Suppress the Proliferation, Migration, and Invasion of SKOV3 Cells

**FIGURE 4:**
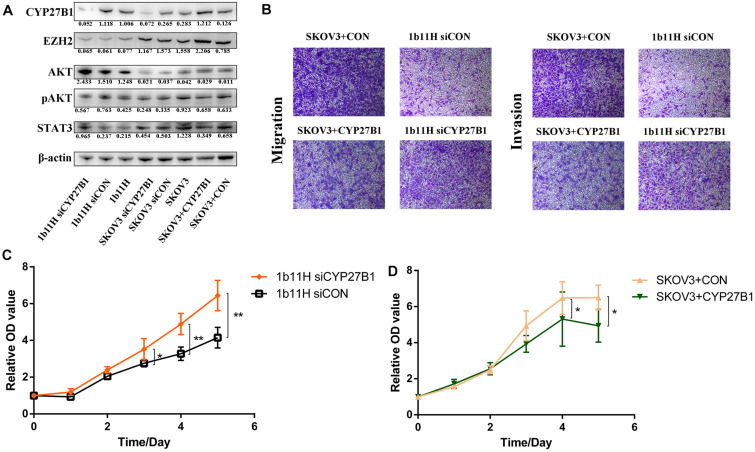
Changes in biological behaviors and relative gene expression levels after silencing or overexpression of CYP27B1. **(A)** Western blot results. **(B)** Transwell migration and invasion assay results. **(C,D)** Cell growth curves. *p < 0.05; **p < 0.01.

The Western blot results showed that CYP27B1 was significantly upregulated in 1b11H cells compared to SKOV3 cells. After transfection with siCYP27B1, the expression of CYP27B1 was significantly decreased in 1b11H cells (1b11H siCYP27B1) compared to the corresponding control (1b11H siCON) cells. Moreover, after transfection with the CYP27B1 overexpression plasmid, CYP27B1 was significantly overexpressed in SKOV3 (SKOV3 + CYP27B1) cells compared to the corresponding control (SKOV3 + CON) cells (Figure 4A). The Transwell migration and invasion assays showed reduced numbers of migrated and invaded cells in the SKOV3 + CYP27B1 groups (52.42 ± 12.60 vs. 136.5 ± 28.53, *p* = 0.0095; 32.18 ± 8.643 vs. 89.74 ± 17.63, *p* = 0.0071) and increased numbers of migrated and invaded cells in the 1b11H siCYP27B1 groups (96.53 ± 10.78 vs. 30.08 ± 7.665, *p* = 0.001; 74.85 ± 13.30 vs. 20.19 ± 11.52, *p* = 0.0058) compared to each corresponding control group (Figure 4B). The cell growth curves showed that silencing CYP27B1 in 1b11H cells reduced cell proliferation from the third day through the fifth day compared to that of 1b11H siCON cells (Figure 4C). The overexpression of CYP27B1 in SKOV3 cells reduced cell proliferation from the fourth day through the fifth day compared to that of SKOV3 CON cells (Figure 4D). The levels of AKT and p-AKT were significantly increased, whereas the level of STAT3 was decreased, in 1b11H cells compared to SKOV3 cells. Moreover, STAT3 and AKT overexpression was observed in 1b11H siCYP27B1 cells, although the differences with respect to SKOV3 cells were not statistically significant. EZH2 expression was upregulated after CYP27B1 overexpression in SKOV3 cells. The levels of p-AKT were not significantly different between the control and experimental groups (Figure 4A). Moreover, after downregulating CYP27B1, the expression of EZH2 also showed no significant difference.

The results of the rescue experiments have shown that after transfection with siCYP27B1, the expression of CYP27B1 was significantly decreased in SKOV3 + CYP27B1 cells. The Transwell migration and invasion assays showed that the numbers of migrated and invaded cells in the SKOV3 + CYP27B1 groups have re-increased after recovering the expression of CYP27B1 in SKOV3 + CYP27B1 + siCYP27B1 (44.21 ± 8.592 vs. 113.6 ± 24.37, *p* = 0.0097; 30.64 ± 6.684 vs. 77.96 ± 12.61, *p* = 0.0046). The cell growth curves also showed the same trend. Above all, the rescue experiments demonstrated that the function of EZH2 in ovarian cancer might be dependent on the expression of CYP27B1 (Supplementary Table S4).

### Evaluation of the Relationship Between Ovarian Cancer Prognosis and EZH2/CYP27B1 Expression as Assessed by IHC

**FIGURE 5:**
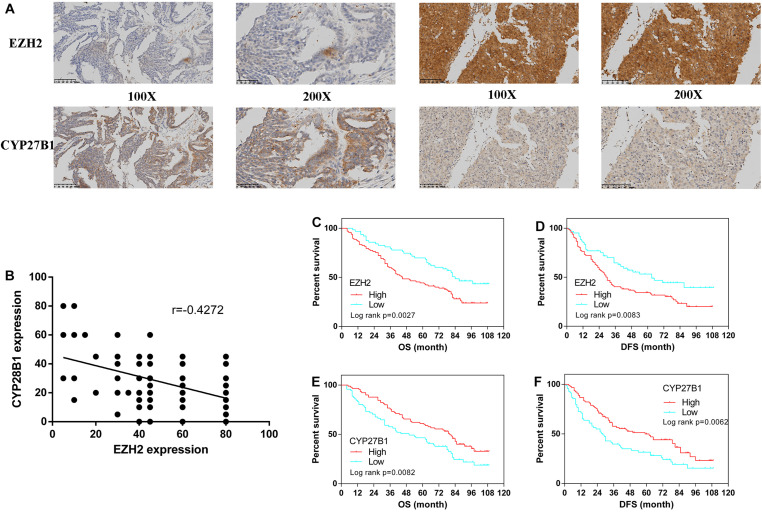
Immunohistochemistry for enhancer of zeste homolog 2 (EZH2) and CYP27B1 and survival analysis of patients stratified by the EZH2 and CYP27B1 expression status. Ovarian cancer samples (*N* = 160). Ovarian cancer samples with weak and strong immunostaining scores for EZH2 and CYP27B1, respectively **(A)** Correlation between the expression levels of EZH2 and CYP27B1 in ovarian tissues **(B)** Overall (OS) and disease-free (DFS) survival curves for patients with ovarian cancer (*N* = 160) stratified according to EZH2 **(C,D)** or CYP27B1 **(E,F)** gene expression status (low or high). Stratification by the gene expression status was conducted according to the median value.

From January 2010 to January 2015, 160 human epithelial ovarian cancer samples with accompanying patient follow-up information were collected from the Department of Pathology of Peking Union Medical College Hospital. Follow-up was performed until January 1, 2020. Supplementary Table S5 summarizes the characteristics of all patients, including their age, disease stage, and tumor grade. The expressions of EZH2 and CYP27B1 in ovarian cancer tissues are shown in Figure 5A.

We examined the association between EZH2 and CYP27B1 in these 160 paired ovarian tissue samples. A statistically significant inverse correlation between EZH2 and CYP27B1 was found (Figure 5B); a high expression of EZH2 was correlated with a low expression of CYP27B1 (Pearson’s correlation test: *r* = −0.4272, *p* < 0.0001). In addition, the correlations between the expressions of EZH2/CYP27B1 and ovarian cancer prognosis are indicated in Figure 5. These data revealed that a high expression of EZH2 [OS: hazard ratio (HR) = 1.851, 95% CI = 1.240–2.689, *p* = 0.0027; progression-free survival (DFS): HR = 1.733, 95% CI = 1.152–2.516, *p* = 0.0083] (Figures 5C,D) and a low expression of CYP27B1 (OS: HR = 1.645, 95% CI = 1.153–2.520, *p* = 0.0082; DFS: HR = 1.674, 95% CI = 1.175–2.573, *p* = 0.0062) (Figures 5E,F) were associated with poor prognosis in patients with ovarian cancer. We also separated the cohort into four groups: EZH2-H/CYP27B1-H, EZH2-L/CYP27B1-H, EZH2-H/CYP27B1-L, and EZH2-L/CYP27B1-L. The data revealed that the group of patients with a high EZH2 expression combined with a low CYP27B1 expression (EZH2-H/CYP27B1-L) had the worst survival, whereas EZH2-L/CYP27B1-H group had the best survival (Supplementary Tables S6A,B).

## Discussion

EZH2 is the catalytic subunit of the multiprotein histone methyltransferase complex called Polycomb repressive complex 2 (PRC2), which can promote transcriptional silencing by installing H3K27me3 (Muller et al., 2002; Margueron and Reinberg, 2011). Recent studies have demonstrated that missense mutations in EZH2 are often identified in various malignancies. Moreover, due to the function of PRC2 in maintaining transcriptional gene silencing, EZH2 mutations always exert both oncogenic and tumor-suppressive effects and both gain-of-function and loss-of-function behavior (Di Croce and Helin, 2013; Comet et al., 2016; Kim and Roberts, 2016). Twenty-two percent of diffuse large cell B-cell lymphomas and 7–12% of follicular lymphomas show recurrent heterozygous point mutations at tyrosine 641 (Y641) within the C-terminal catalytic SET domain of EZH2 (Morin et al., 2010; Bödör et al., 2011). Functional analysis has demonstrated that the mutated EZH2 shows more function on methylates dimethyl H3K27 (Yap et al., 2011). Monoallelic mutation results in the expression of wild-type and mutated EZH2, which causes the dysregulation of transcriptome in lymphoma cells according to the accumulation of H3K27me3. EZH2 inactivating deletions, frameshift, and nonsense and missense mutations have been identified in parts of T-cell acute lymphocytic leukemia, myeloproliferative neoplasms, and myelodysplastic syndromes (Ernst et al., 2010; Nikoloski et al., 2010; Ntziachristos et al., 2012). The gain-of-function role for mutant EZH2 in cancer is always resulted by the other chromatin regulators’ loss-of-function mutations which can normally antagonize EZH2 activity, such as the ubiquitously transcribed tetratricopeptide repeat gene on X chromosome (UTX) (van Haaften et al., 2009; Gui et al., 2011).

Solid tumors often exhibit EZH2 overexpression, as reported in various cancer types, with adverse outcomes, including prostate cancer, breast cancer, endometrial cancer, colorectal cancer, melanoma, cholangiocarcinoma, and ovarian cancer. In several tumor models, the knockdown of EZH2 protein expression results in proliferation inhibition, further demonstrating that EZH2 is a bona fide oncogene (Varambally et al., 2002; Kleer et al., 2003; Karanikolas et al., 2009; Li et al., 2009; Eskander et al., 2013; Nakagawa et al., 2013; Katona et al., 2014; Tang et al., 2014). Moreover, overexpression of EZH2 can promote tumorigenesis *in vivo* (Jiang et al., 2016).

As more in-depth studies were conducted, the possible molecular mechanisms of overexpressed EZH2 in tumors were implied. EZH2 overexpression can arise not only *via* gene amplification but also *via* alterations in various signaling pathways (Saramäki et al., 2006). Fujii et al. reported that the MEK–ERK–Elk-1 signal transduction pathway was upregulated in breast cancer cells and that this upregulation was associated with the overexpression of EZH2 (Fujii et al., 2011). Moreover, EZH2 expression has been shown to be linked to the activity of the pRb–E2F complex, which binds to the EZH2 promoter to activate its transcription (Bracken et al., 2003) and to the loss of expression of many different microRNAs (miRNAs), including miR-101, miR-26a, and miR-124, in different tumors (Varambally et al., 2008; Lu et al., 2011; Zheng et al., 2012). EZH2 can also epigenetically repress tumor suppressor mRNAs or miRNAs to promote tumor proliferation. Moreover, the tumor suppressor locus Ink4a/Arf, E-cadherin, FOXC1, and components of DNA damage repair pathways, which can be silenced by EZH2, have been shown to contribute to oncogenesis (Bruggeman et al., 2005; Cao et al., 2008; Chang et al., 2011; Du et al., 2012).

Later studies demonstrated that EZH2 is upregulated in ovarian cancer and that this upregulation is associated with a high proliferation index, a high tumor grade, and poor prognosis (Di Croce and Helin, 2013). However, EZH2 mutation has not been reported in epithelial ovarian cancer. Moreover, there is also no EZH2 mutation data in epithelial ovarian cancer on the advanced The Cancer Genome Atlas (TCGA) database. Recently, studies have demonstrated that the overexpression of EZH2 correlates with a high proliferative index and tumor grade in ovarian cancer. Moreover, it is reported that NF-YA is a key regulator of EZH2 expression in ovarian cancer, and it is required for cell proliferation (Garipov et al., 2013). Besides, EZH2 expression was also positively correlated with KDM2B, which was significantly associated with tumor histological type, stage, and lymph node metastasis. Furthermore, the knockdown of KDM2B decreases the expression of EZH2 and reduced proliferation and migration. Therefore, EZH2 may also be regulated by KDM2B in ovarian cancer (Kuang et al., 2017). In addition, miRNAs have also been demonstrated to play an important role in the regulation of EZH2 expression. EZH2 has been identified as a target of miRNA-101 and miRNA-298. Studies have shown that the downregulation of miRNA-101 or miRNA-298 results in the overexpression of EZH2, thereby resulting in cancer progression (Varambally et al., 2008; Liu et al., 2014; Zhou et al., 2016).

The results of the present study showed that the knockout of EZH2 by CRISPR/Cas9 significantly suppressed the proliferation, migration, and invasion and promoted the apoptosis of SKOV3 cells, consistent with previous studies that focused on the function of EZH2 (Müller et al., 2002; Di Croce and Helin, 2013). According to a previous study, the overexpression of EZH2 promotes the survival of ovarian cancer cells *via* trimethylation of ARHI, a negative regulator of cancer cell growth and progression. However, the H3K27me3-related molecular mechanism affected by EZH2 in ovarian cancer remains unclear. Therefore, in the present study, we investigated the relevant hub genes and signaling pathways according to the results of an integrated analysis of ChIP-seq and mRNA-seq data. The results showed that steroid biosynthesis was activated after EZH2 knockout in ovarian cancer cells and that the hub gene of steroid biosynthesis, CYP27B1, was upregulated.

Cell proliferation, angiogenesis, and cell cycle progression are upregulated in tumors by CYP27B1 ablation, and apoptosis is decreased *in vivo* (Li et al., 2016). However, Urbschat et al. (2013) showed that the expression of CYP27B1 was significantly higher in renal cell carcinoma tissue than in non-tumor tissue and suggested that the upregulation of CYP27B1 contributed to the pathogenesis of renal cell carcinoma due to an imbalance in vitamin D metabolites (Urbschat et al., 2013). Recently, Brożyna et al. (2015) evaluated the expression of CYP27B1 in samples from ovarian cancer, metastases, and normal ovaries and found that the expression of CYP27B1 was significantly higher in normal ovarian epithelium than in ovarian cancer samples, especially in poorly differentiated primary and metastatic tumors. A previous study also suggested that CYP27B1 can regulate the behavior of ovarian cancer cells and induce a less aggressive phenotype by affecting the concentration of active vitamin D within the tumor microenvironment. In the present study, our data showed that CYP27B1 was upregulated after the knockout of the oncogene EZH2, which may reduce the level of H3K27me3 in ovarian cancer cells; this finding was also consistent with previous data (Sharma et al., 2017; Camilleri et al., 2018). After silencing CYP27B1 with siRNA, the proliferation, migration, and invasion of ovarian cancer cells were significantly suppressed, indicating that the function of EZH2 in promoting ovarian cancer progression was achieved by the inhibition of CYP27B1. Interestingly, after ectopic overexpression of CYP27B1 in SKOV3 cells, the EZH2 expression was significantly upregulated, reminding us that a closed feedback pathway may exist between EZH2 and CYP27B1. Further study should be performed. Previous studies have shown that the expression of CYP27B1 in normal epithelium was significantly higher than that in ovarian cancer tissues and that CYP27B1 expression in the low-grade group was higher than that in the high-grade group (Brożyna et al., 2015). In the present study, the IHC results for the 160 ovarian cancer samples revealed an inverse correlation between the expression levels of EZH2 and CYP27B1, which was significantly associated with the prognosis of ovarian cancer.

As research has progressed, studies have also shown that EZH2 exerts not only a repressive but also a PRC2-independent effect on transcriptional activation to directly modulate the activity of transcription factors and other proteins, such as the androgen receptor in prostate cancer and NF-κB, NOTCH1, the estrogen receptor (ER), and Wnt signaling transcription factors in breast cancer (Shi et al., 2007; Lee et al., 2011; Xu et al., 2012; Jung et al., 2013; Gonzalez et al., 2014). In addition, the tumorigenicity of cancer stem cells was found to be promoted by EZH2 *via* the binding and methylation of STAT3 in glioblastoma and in a model of prostate cancer (Xu et al., 2012; Kim et al., 2013). A later study also reported that AKT phosphorylation, integrin expression, and activation of STAT3 signaling pathways were upregulated after CYP27B1 ablation in breast tumors (Li et al., 2016). However, the contribution of these PRC2-independent functions to the oncogenic transformation activity of EZH2 remains unclear. Therefore, the levels of AKT, p-AKT, and STAT3 were evaluated in the present study. The results indicated that EZH2 can suppress the expression and phosphorylation of AKT *via* a PRC-dependent mechanism, consistent with a previous study in hepatic carcinogenesis (Lee et al., 2018), whereas it can promote the expression of STAT3 *via* a PRC2-independent mechanism and simultaneous CYP27B1 downregulation. However, the relationship between STAT3 and CYP27B1 remains to be further explored.

## Conclusion

In summary, EZH2 plays an important role in promoting cell proliferation, migration, and invasion in ovarian cancer by regulating the core gene of steroid biosynthesis *via* H3K27me3. Moreover, CYP27B1, the hub gene of steroid biosynthesis, might be a novel therapeutic target for ovarian cancer.

## Data Availability Statement

The datasets generated for this study can be found in online repositories. The names of the repository/repositories and accession number(s) can be found below: https://www.ncbi.nlm.nih.gov/geo/, 145692.

## Ethics Statement

The studies involving human participants were reviewed and approved by the all procedures performed in studies involving human participants were in accordance with the ethical standards of the institutional and national research committee and with the 1964 Helsinki declaration and its later amendments. (The name and affiliation of the ethics committee that approved this study: The institutional ethics committee of Peking Union Medical College Hospital, CAMS Chinese Academy of Medical Sciences, No. S-341 2019). The patients/participants provided their written informed consent to participate in this study. The animal study was reviewed and approved by the all procedures performed in studies involving animal were in accordance with the ethical standards of the institutional and national research committee and 3Rs. (The name and affiliation of the ethics committee that approved this study: The institutional ethics committee of Peking Union Medical College Hospital, CAMS Chinese Academy of Medical Sciences, No. S-0081 2014).

## Author Contributions

XH contributed to the study design and data analysis. HS did the *in vivo* experiment and data analysis and wrote the manuscript. QQ did the *in vivo* and *in vitro* experiments and data analysis. XM, PP, MY, YZ, JY, and DC did the *in vivo* experiment. TG contributed to the study design and data analysis. KS helped in the study design and funding. All authors have read, edited, and approved the final version of the manuscript.

## Conflict of Interest

The authors declare that the research was conducted in the absence of any commercial or financial relationships that could be construed as a potential conflict of interest.

## Footnotes

1 http://crispr.mit.edu/

2 http://crispr.mit.edu/

3 http://crispr.mit.edu/
