# Supplementary figures and images for: CYP27B1 Downregulation: A New Molecular Mechanism Regulating EZH2 in Ovarian Cancer Tumorigenicity
Source: Front Cell Dev Biol. 2020 Oct 14;8:561804. doi: 10.3389/fcell.2020.561804 (PMC7591459; doi:10.3389/fcell.2020.561804)

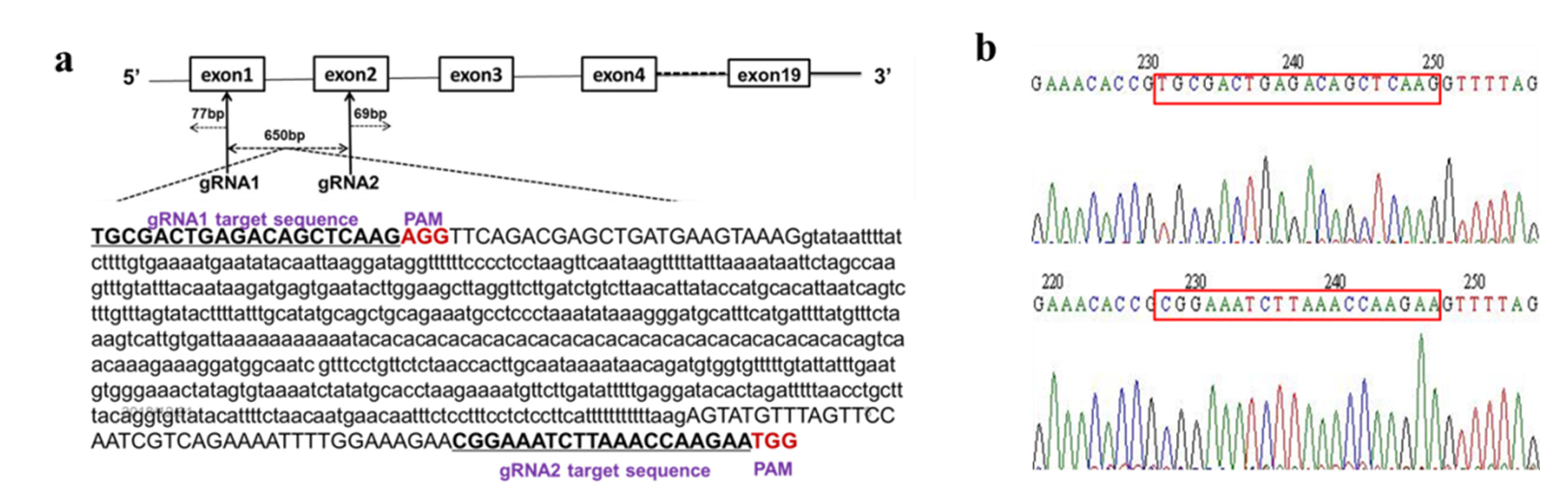

Supplement: Supplementary file 1 [file Image_1.JPEG]

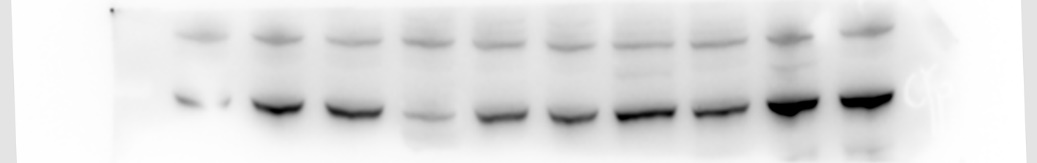

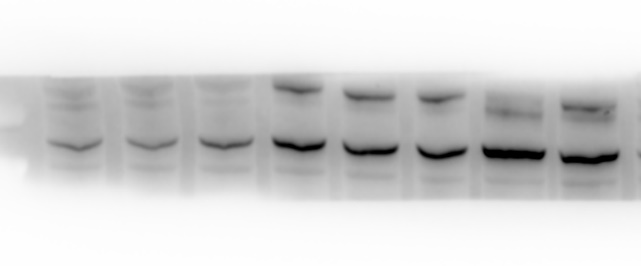

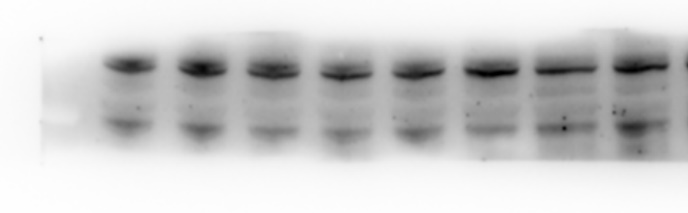

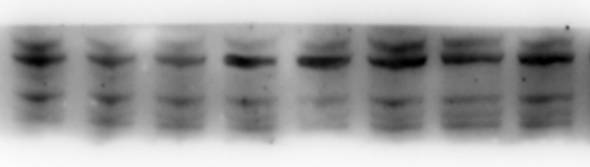

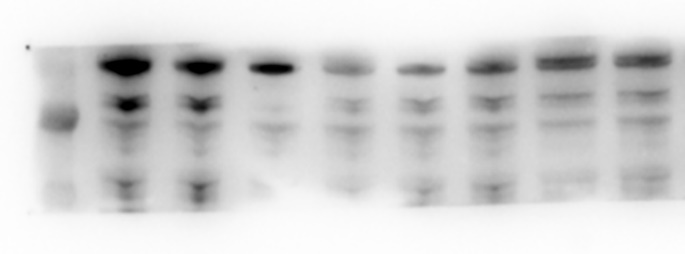

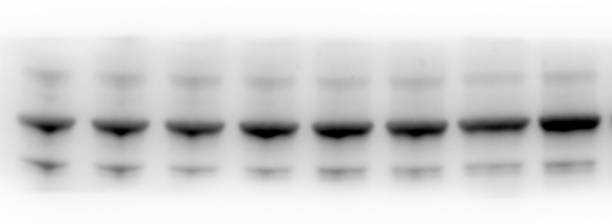


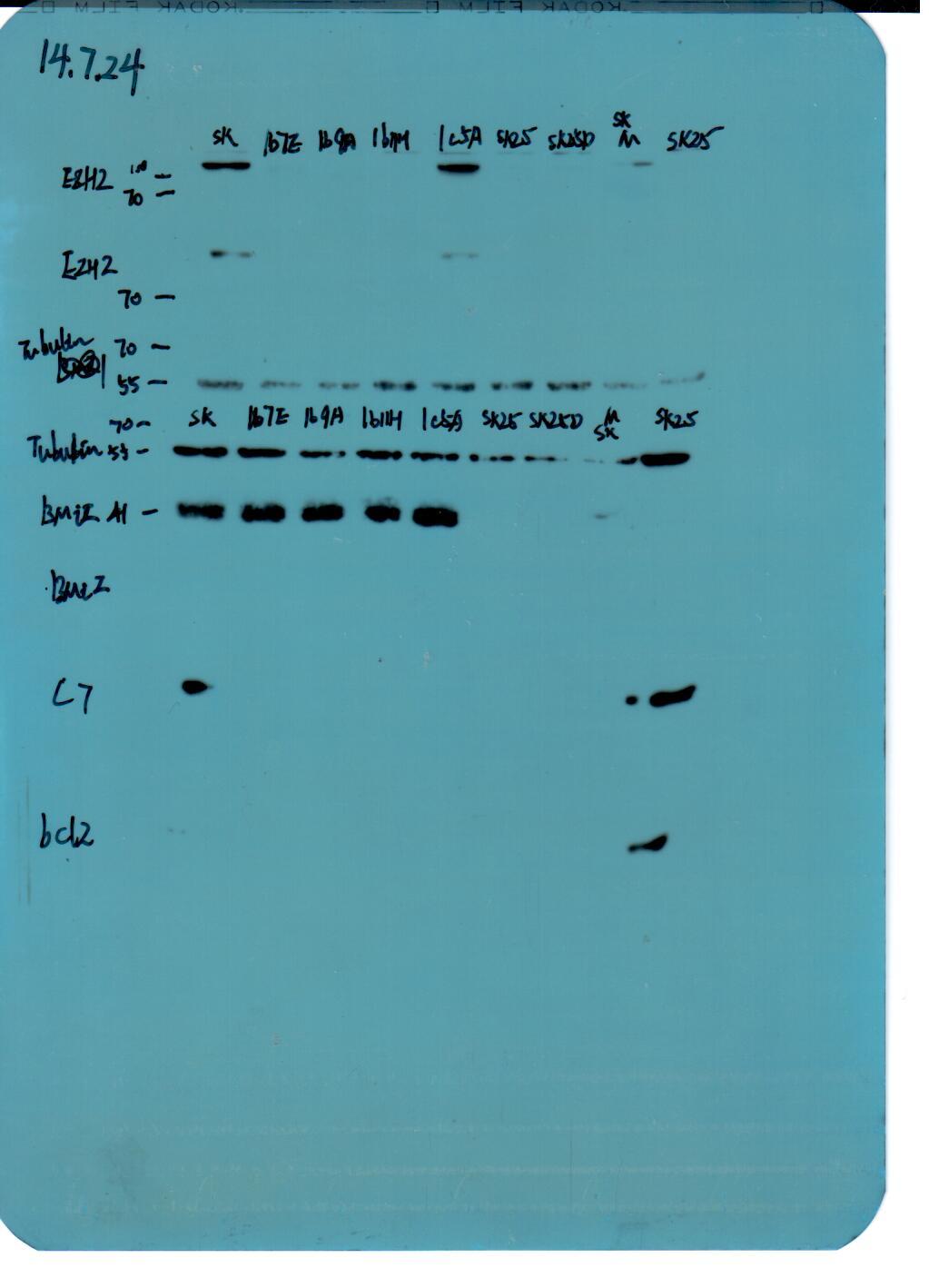

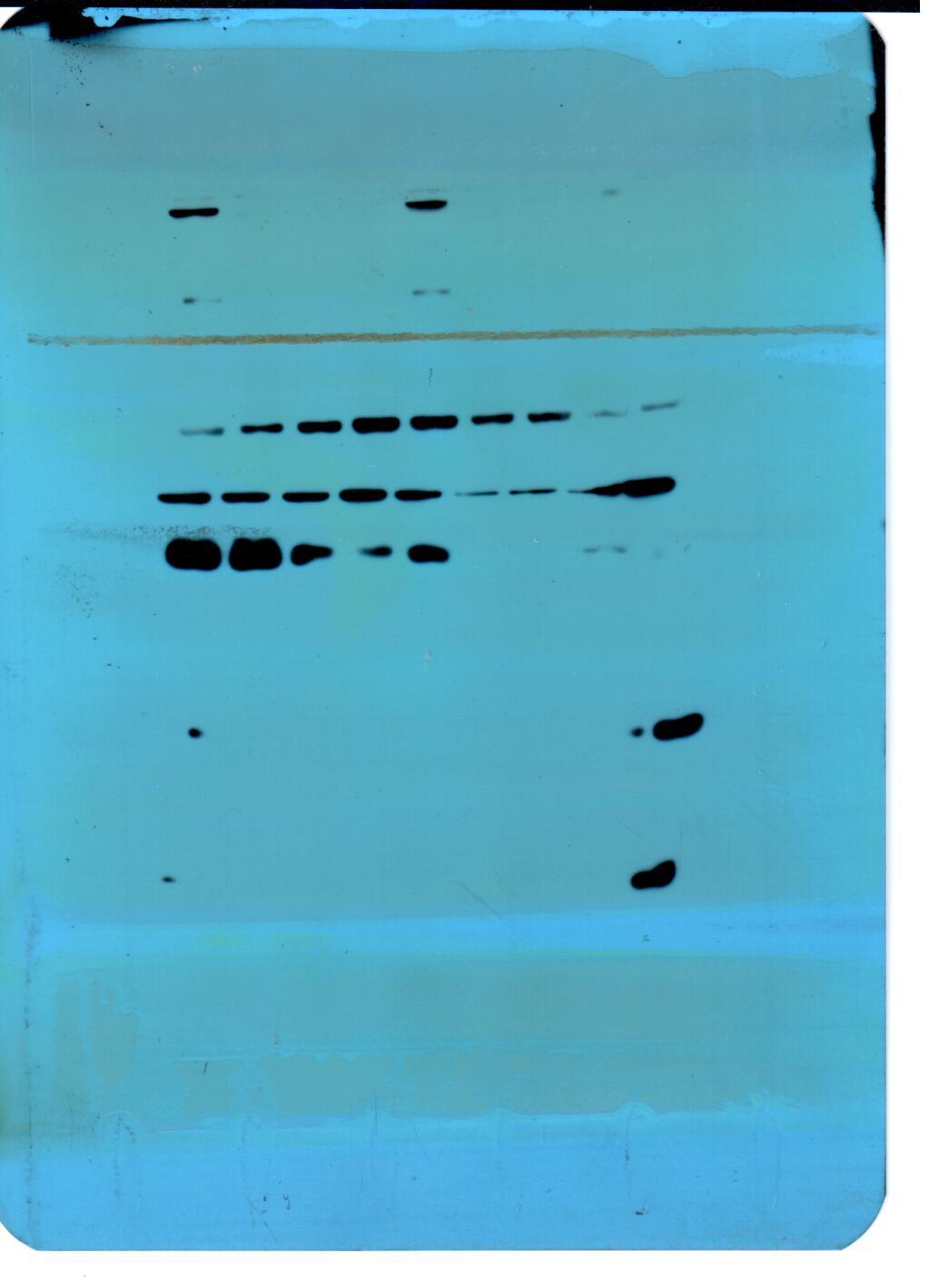

Supplement: Supplementary file 4 [file Table_3.DOCX]
