# Supplementary material for: CYP27B1 Downregulation: A New Molecular Mechanism Regulating EZH2 in Ovarian Cancer Tumorigenicity
Source: Front Cell Dev Biol. 2020 Oct 14;8:561804. doi: 10.3389/fcell.2020.561804 (PMC7591459; doi:10.3389/fcell.2020.561804)
Supplement: Supplementary file 2 [file Table_1.DOCX]

gene_id log2FoldChange pvalue padj s1B11H.1 s1B11H.2 s1B11H.3 sSKOV3.1 sSKOV3.2 sSKOV3.3 Style s1B11H-1 s1B11H-2 s1B11H-3 sSKOV3-1 sSKOV3-2 sSKOV3-3

MCAM 3.31614132429761 0 0 3383.50493537046 3801.6528476079 3703.16774415066 367.925088106643 375.470037085283 348.187578163427 up 3176.0 3693.0 3488.0 389.0 453.0 325.0

CHST2 -2.87185129984866 0 0 518.818294560899 571.328819502406 549.954384022375 4235.3947160451 3689.22104871213 4087.18649444146 down 487.0 555.0 518.0 4478.0 4451.0 3815.0

CXCL12 6.18677980302163 0 0 3366.45957045676 3326.06020867077 3349.62564013628 36.8870910955246 52.2176872767613 48.2105877457052 up 3160.0 3231.0 3155.0 39.0 63.0 45.0

CXCL1 -4.16375300656647 0 0 252.484467784257 210.001944465749 207.029160008423 4025.4220436552 3854.16263233238 4117.18419348323 down 237.0 204.0 195.0 4256.0 4650.0 3843.0

PLXDC2 4.30809010335463 0 0 1996.43836551771 1788.10479184807 1933.33384807866 92.690639163113 102.77767019553 92.1357899140145 up 1874.0 1737.0 1821.0 98.0 124.0 86.0

DYSF 3.45301890563673 0 0 5915.80696036278 6556.38423677626 6056.93004024642 559.927126373091 570.250299149393 561.385510638879 up 5553.0 6369.0 5705.0 592.0 688.0 524.0

TENM2 2.40125363205995 0 0 4236.83851636282 4482.10032452879 4327.44028817606 851.240563742875 810.617431058294 807.79518133915 up 3977.0 4354.0 4076.0 900.0 978.0 754.0

KRT8 -3.53385745698723 0 0 1586.28427228168 1589.42648164273 1680.65210406838 20022.123882081 18281.9925121194 17940.7667197684 down 1489.0 1544.0 1583.0 21169.0 22057.0 16746.0

KRT7 -6.40820318167416 0 0 266.333826776642 307.796967623819 315.321336012829 26176.593157942 24274.593766262 25116.6448691181 down 250.0 299.0 297.0 27676.0 29287.0 23444.0

KRT5 -5.17493319878136 0 0 87.3574951827386 97.7950231580696 101.922048004147 3511.84023686366 3396.63622952647 3465.80558571903 down 82.0 95.0 96.0 3713.0 4098.0 3235.0

FAM84B -2.9689524685975 0 0 597.653107286785 617.652777840439 592.421904024102 4595.75322136292 4892.71441261463 4664.64220099557 down 561.0 600.0 558.0 4859.0 5903.0 4354.0

RRAD -4.84559700789607 0 0 93.749507025378 108.089236122077 99.7986720040603 2979.34197310006 2833.84559998804 2865.85160488359 down 88.0 105.0 94.0 3150.0 3419.0 2675.0

NID2 -4.21790063559892 0 0 279.117850461921 290.296805585006 300.457704012224 5720.33658835212 5224.25528421311 5243.16925394403 down 262.0 282.0 283.0 6048.0 6303.0 4894.0

AKR1C1 -5.28349660516044 0 0 53.2667653553284 40.1474305596286 55.2077760022461 1873.67506308293 1998.36260355986 1904.85388915253 down 50.0 39.0 52.0 1981.0 2411.0 1778.0

LOC100505817 -4.68281624557447 0 0 137.428254616747 143.089560199702 147.574632006004 3750.18759471167 3689.22104871213 3554.72733645 down 129.0 139.0 139.0 3965.0 4451.0 3318.0

ERBB2 -2.13628500813186 0 0 12445.2470576189 14456.1632653555 13435.6616405466 60861.8086619184 57753.590980277 58723.709913452 down 11682.0 14043.0 12655.0 64348.0 69679.0 54813.0

CEMIP 4.897173704788 0 0 2303.2549339644 2296.63891227003 2313.41815209412 67.1534222508268 93.6602962265718 68.5661692383363 up 2162.0 2231.0 2179.0 71.0 113.0 64.0

AKT3 3.65839317656663 0 0 2281.94822782227 2059.87201409787 2107.45068008574 154.169124322321 183.176331558163 172.48676949019 up 2142.0 2001.0 1985.0 163.0 221.0 161.0

FRAS1 -4.06613522511684 0 0 168.322978522838 165.736828720518 182.610336007429 3033.25387547044 2817.26855640812 2801.57082122265 down 158.0 161.0 172.0 3207.0 3399.0 2615.0

MATN2 2.35871995859426 0 0 6302.52367684246 6405.05930620536 6678.01752027169 1307.12717676961 1268.9726860432 1200.97930806523 up 5916.0 6222.0 6290.0 1382.0 1531.0 1121.0

FBN2 2.70087996893256 1.02466527275532e-307 8.26563320022624e-305 3332.36884062935 3129.44074105823 3084.20364012548 488.044589879248 516.374907514639 460.678949570072 up 3128.0 3040.0 2905.0 516.0 623.0 430.0

PAPLN -2.93991315275924 7.20867683801092e-296 5.55068116526841e-293 316.404586210651 347.944398183448 338.678472013779 2706.94499270234 2512.25095453751 2479.0955565236 down 297.0 338.0 319.0 2862.0 3031.0 2314.0

SLC7A11 -2.95619168762465 2.65858883829201e-292 1.95810847481159e-289 354.756657266487 304.708703734617 321.691464013088 2447.78953218507 2644.03845099791 2517.66402672016 down 333.0 296.0 303.0 2588.0 3190.0 2350.0

EMP1 -4.06007278974552 6.81247361118861e-272 4.80847095723062e-269 84.1614892614189 89.5596527868637 99.7986720040603 1496.29174649025 1519.28604410005 1547.02419343996 down 79.0 87.0 94.0 1582.0 1833.0 1444.0

AKR1C3 -3.04437945561171 3.93854274483954e-266 2.66875656390327e-263 241.831114713191 236.766898172168 215.522664008768 1944.61177672817 1920.45049873422 1860.92868698422 down 227.0 230.0 203.0 2056.0 2317.0 1737.0

SLC25A6 -1.89177919306255 8.20412857690925e-262 5.34530531126318e-259 2741.1077451852 3030.61629660376 2969.54133612081 11035.8609975021 10512.3321862089 10894.521484135 down 2573.0 2944.0 2797.0 11668.0 12683.0 10169.0

BST2 -4.01508322599837 1.94601432376618e-252 1.22094380165182e-249 89.4881657969518 118.383449086084 75.3798480030668 1554.93276310365 1525.91686153202 1507.38437684905 down 84.0 115.0 71.0 1644.0 1841.0 1407.0

CD320 -3.84627967122437 9.34659144778037e-251 5.65468782590713e-248 107.598866017763 125.589398160889 96.6136080039307 1678.83555627067 1476.18573079225 1595.23478118567 down 101.0 122.0 91.0 1775.0 1781.0 1489.0

LAMB3 -2.03459282807478 2.72665219507926e-242 1.59274097188423e-239 1011.00320644413 1075.74525473877 1023.46723204164 4398.07624600485 4196.47858225781 4149.3245853137 down 949.0 1045.0 964.0 4650.0 5063.0 3873.0

LOXL1 -3.5060029188126 3.91764355666579e-242 2.21216272833061e-239 138.493589923854 151.324930570908 122.094120004967 1632.49023668911 1476.18573079225 1575.95054608739 down 130.0 147.0 115.0 1726.0 1781.0 1471.0

CLDN1 -2.97717082211312 4.11011510367789e-237 2.20719338423204e-234 255.680473705576 235.737476875768 227.201232009244 1826.38392065277 2012.4530906028 1815.93213842156 down 240.0 229.0 214.0 1931.0 2428.0 1695.0

LTBP1 -3.33163033918454 4.16943260303574e-237 2.20719338423204e-234 138.493589923854 147.207245385305 143.327880005831 1411.16769011597 1452.14901760136 1457.03109631465 down 130.0 143.0 135.0 1492.0 1752.0 1360.0

GDA -4.28716596897856 7.34669915448988e-235 3.77130556597147e-232 76.7041421116729 80.2948611192571 82.8116640033692 1392.2512331439 1760.48202818795 1526.66861194733 down 72.0 78.0 78.0 1472.0 2124.0 1425.0

FBN1 -2.08593242132273 1.17449419537119e-231 5.85174460870232e-229 747.865385588811 760.742338040141 781.402368031791 3173.23565706372 3229.20808936924 3321.17382248192 down 702.0 739.0 736.0 3355.0 3896.0 3100.0

FNDC1 -2.38970376564651 3.45625599318662e-229 1.67282790070232e-226 573.150395223334 593.976088023222 538.2758160219 3163.77742857769 2819.75511294511 2956.91604840325 down 538.0 577.0 507.0 3345.0 3402.0 2760.0

PDGFB 3.71549635687506 1.13584684859542e-227 5.34479044866846e-225 1205.95956764464 1387.65990754819 1311.18468005335 92.690639163113 103.606522374526 100.706561068806 up 1132.0 1348.0 1235.0 98.0 125.0 94.0

TNIK 2.97552590906054 9.6120267817977e-225 4.40074955901765e-222 1593.74161943143 1464.86650477824 1614.8274480657 194.839506812258 198.095670780094 201.413122137613 up 1496.0 1423.0 1521.0 206.0 239.0 188.0

PHLDA1 -1.99352386304673 7.63661186894999e-224 3.40432118578981e-221 1168.67283189591 1187.95217604644 1074.42825604371 4459.55473116406 4726.94397681539 4475.01388919579 down 1097.0 1154.0 1012.0 4715.0 5703.0 4177.0

IDO1 -6.23914936546522 3.20277022318434e-222 1.39115198924981e-219 31.9600592131971 13.3824768532095 21.2337600008639 1684.51049336229 1646.92927966547 1676.65710715619 down 30.0 13.0 20.0 1781.0 1987.0 1565.0

AMIGO2 -1.80810202023827 6.98111515881422e-219 2.95650226975782e-216 1197.43688518778 1185.89333345364 1175.28861604782 4146.4873682764 4183.21694739387 4131.11169660976 down 1124.0 1152.0 1107.0 4384.0 5047.0 3856.0

MFAP3L -3.05253616159556 6.59291838076405e-218 2.7240009114669e-215 218.393737956847 215.149050947753 186.857088007602 1582.36162571314 1801.92463713776 1763.43616509846 down 205.0 209.0 176.0 1673.0 2174.0 1646.0

SRGN 2.66678104079897 3.42814294823242e-210 1.38268432245374e-207 1857.94477559386 1685.162662208 1845.21374407507 286.584323126768 266.061549457784 297.834297629023 up 1744.0 1637.0 1738.0 303.0 321.0 278.0

S100A4 -1.67528290671351 5.99251300403347e-210 2.36077140205411e-207 2034.79043657355 2038.25416687345 2156.28832808773 6732.36703635754 6460.07388309646 6704.48573583608 down 1910.0 1980.0 2031.0 7118.0 7794.0 6258.0

TNS1 2.37357982127716 6.94051117437909e-208 2.67209680213595e-205 2667.59960899485 3069.73430586698 2713.6745281104 538.173200855218 523.834577125605 571.02762818802 up 2504.0 2982.0 2556.0 569.0 632.0 533.0

SPP1 -2.65306163008082 1.40711728749132e-206 5.29701485557845e-204 283.379191690347 273.826064842595 291.964200011878 1688.2937847567 1777.88792394687 1875.92753650511 down 266.0 266.0 275.0 1785.0 2145.0 1751.0

KIAA1551 -2.66213748201416 1.7908821545808e-205 6.59511819534757e-203 686.07593777663 565.152291724002 557.386200022677 3474.95314576814 4215.54218237473 3751.85507301022 down 644.0 549.0 525.0 3674.0 5086.0 3502.0

KLF5 -2.66121712671651 1.00480518332237e-204 3.62157442669807e-202 272.725838619282 282.061435213801 277.100568011274 1675.99808772486 1861.60199402549 1722.7250021132 down 256.0 274.0 261.0 1772.0 2246.0 1608.0

SERPINE1 1.51682209301719 1.80413698504186e-204 6.36710010971024e-202 13498.8636763473 12935.7080105716 13852.9050245636 4794.37601956959 4664.78006339067 4618.57430603856 up 12671.0 12566.0 13048.0 5069.0 5628.0 4311.0

TPM2 -2.40002271929664 2.2884333992388e-204 7.91144118022556e-202 352.625986652274 355.150347258253 343.986912013995 1860.43354320248 1860.77314184649 1829.8596415481 down 331.0 345.0 324.0 1967.0 2245.0 1708.0

SYK 5.56883672139762 3.6636220350282e-201 1.24123514546756e-198 1163.34615536037 1102.51020844518 1168.91848804756 23.6455712150799 20.7213044749053 28.9263526474231 up 1092.0 1071.0 1101.0 25.0 25.0 27.0

RIPK4 -2.10894416913931 2.2331864409292e-198 7.41768202143933e-196 530.536982939071 541.475601906785 539.337504021943 2330.50749895827 2281.00119659757 2340.89187165258 down 498.0 526.0 508.0 2464.0 2752.0 2185.0

CXCL8 -2.99344748467746 5.05898723081942e-196 1.6480623786554e-193 156.604290144666 183.23699075933 157.129824006393 1341.17679931933 1315.38840806699 1303.82856192274 down 147.0 178.0 148.0 1418.0 1587.0 1217.0

SLC3A2 -1.49123796308864 1.58354953334011e-194 5.06138284807198e-192 7485.04586773075 8149.9284036046 7886.21846432085 22523.8253166365 21444.892427169 22160.8001671092 down 7026.0 7917.0 7428.0 23814.0 25873.0 20685.0

ST6GAL2 5.53477243723769 1.37254693532216e-191 4.3057305711773e-189 1078.11933079185 1037.65666677194 1145.56135204661 26.4830397608894 23.2078610118939 20.3555814926311 up 1012.0 1008.0 1079.0 28.0 28.0 19.0

GJA1 -3.29616037091176 1.9661109339725e-191 6.05562167663531e-189 117.186883781723 131.765925939294 116.785680004751 1225.78641178974 1126.41011125585 1244.90451023354 down 110.0 128.0 110.0 1296.0 1359.0 1162.0

CYP1B1 -1.42405001426706 3.93412944370889e-191 1.19007415672194e-188 4518.08703743896 4442.98231526556 4348.67404817692 11704.5577514645 12200.7040748242 11807.3086121204 down 4241.0 4316.0 4096.0 12375.0 14720.0 11021.0

RPS6KA2 -5.90691442271896 1.42370043064891e-186 4.2311377710864e-184 22.3720414492379 19.5590046316139 21.2337600008639 1305.23553107241 1209.29532915547 1273.83086288097 down 21.0 19.0 20.0 1380.0 1459.0 1189.0

ELOVL2 4.12587466312446 2.07118595411493e-186 6.04929139012187e-184 1021.6565595152 919.273217685854 1079.73669604393 62.4243080078108 63.8216177827082 44.9965485626582 up 959.0 893.0 1017.0 66.0 77.0 42.0

MAN1A1 3.21738738472188 9.78127089727478e-185 2.80838523728533e-182 1291.18639221316 1170.45201400763 1095.66201604458 125.794438864225 131.787496460397 124.276181744485 up 1212.0 1137.0 1032.0 133.0 159.0 116.0

NTN4 -2.00534642171383 4.45495915076367e-184 1.25778346689894e-181 710.578649840081 800.88976859977 701.775768028552 3014.33741849838 2907.61344391871 2967.62951234674 down 667.0 778.0 661.0 3187.0 3508.0 2770.0

NEURL1 -6.10683929876265 5.92008986880443e-182 1.64403807176307e-179 22.3720414492379 17.5001620388124 16.9870080006911 1375.22642186904 1288.86513833911 1250.26124220529 down 21.0 17.0 16.0 1454.0 1555.0 1167.0

CRISPLD2 2.8829583341503 6.24991956079049e-181 1.70763931225469e-178 1159.08481413195 1177.65796308244 1167.85680004751 146.602541533495 169.085844515227 158.559266363653 up 1088.0 1144.0 1100.0 155.0 204.0 148.0

CNN1 4.88251299748122 1.54055453084865e-177 4.14237996072638e-175 857.594922220788 995.450393619508 913.051680037147 33.1037997011118 31.496382801856 28.9263526474231 up 805.0 967.0 860.0 35.0 38.0 27.0

MARCKSL1 -4.26930169061668 2.0708689715663e-176 5.48133130911454e-174 49.0054241269022 38.0885879668271 64.7629680026348 960.010191332242 939.918370981703 1024.20715299765 down 46.0 37.0 61.0 1015.0 1134.0 956.0

ABCC3 1.76575743123638 1.07547781578235e-173 2.80286064605431e-171 3093.73373183748 3094.4404169806 2979.0965281212 886.236009141193 882.727570630964 928.857323900588 up 2904.0 3006.0 2806.0 937.0 1065.0 867.0

MKX 3.18243160805405 1.73003727693572e-169 4.44042901080168e-167 944.952417403526 942.949907503071 1008.60360004103 110.661273286574 104.435374553523 103.920600251854 up 887.0 916.0 950.0 117.0 126.0 97.0

TFPI2 -2.17337431031982 2.7737908054832e-167 7.01313675296798e-165 425.068787535521 456.033634305524 415.120008016889 2045.81482152871 1927.91016834519 1873.78484371641 down 399.0 443.0 391.0 2163.0 2326.0 1749.0

GLIPR2 1.71396677401798 1.44565938745569e-166 3.60139265051462e-164 3012.76824849738 3059.44009290298 3168.07699212889 951.497785694814 919.197066506797 947.070212604521 up 2828.0 2972.0 2984.0 1006.0 1109.0 884.0

MECOM -2.07941978485249 2.12485833077813e-166 5.21668117730166e-164 777.694774187795 641.329467657656 708.145896028811 2845.03512859841 3119.79960174174 3021.19683206419 down 730.0 623.0 667.0 3008.0 3764.0 2820.0

SPARC -1.45370628473455 2.81793288471501e-166 6.81939758101033e-164 3433.57569480447 3544.29752350772 3621.41776814734 10093.8214402933 9340.3352051083 9600.33503976144 down 3223.0 3443.0 3411.0 10672.0 11269.0 8961.0

ASTN2 2.27764725405479 1.56175313720093e-165 3.72621100622307e-163 1524.4948244695 1665.60365757638 1615.88913606574 335.767111254134 319.108088913541 337.474114219937 up 1431.0 1618.0 1522.0 355.0 385.0 315.0

SLC6A6 1.99979248391996 3.01446164147814e-163 7.09235836203329e-161 2143.45463789842 2176.19662059115 2188.13896808902 533.444086612202 511.401794440662 586.026477708906 up 2012.0 2114.0 2061.0 564.0 617.0 547.0

CREB3L1 2.3988447225002 2.92316257707993e-162 6.78333891174438e-160 1650.20439070807 1915.75303260176 1762.4020800717 359.412682469214 309.990714944583 342.830846191682 up 1549.0 1861.0 1660.0 380.0 374.0 320.0

HMGA1 -1.59666476609643 3.22990216487576e-162 7.39385711797235e-160 2223.35478593141 2469.58169006536 2225.29804809054 7195.8202321731 6760.94722407209 6971.25098802898 down 2087.0 2399.0 2096.0 7608.0 8157.0 6507.0

TAGLN -1.71298267301443 1.98613954586794e-161 4.4860271876004e-159 2397.00444098978 2544.72944470261 2806.04138411416 8781.01932643206 8080.47989303406 8540.77345575027 down 2250.0 2472.0 2643.0 9284.0 9749.0 7972.0

COL4A5 4.40656314733193 3.46922743761843e-160 7.7327253675337e-158 789.413462565967 749.418703779733 813.253008033087 30.2663311553022 36.4694958758333 44.9965485626582 up 741.0 728.0 766.0 32.0 44.0 42.0

HEXIM1 -1.58310594552521 4.43194213099249e-159 9.75027268818347e-157 2155.17332627659 2372.81608820369 2353.76229609576 6823.16602982344 6620.04235364273 7181.23488132138 down 2023.0 2305.0 2217.0 7214.0 7987.0 6703.0

IVNS1ABP -1.73685121084613 5.76128602465685e-159 1.25123314432932e-156 1327.40779265478 1248.68803253409 1334.5418160543 4137.97496263898 4635.77023712581 4255.38787835425 down 1246.0 1213.0 1257.0 4375.0 5593.0 3972.0

PRSS23 1.26817384923505 8.15699313962469e-157 1.74910713652205e-154 17451.2576657127 17693.6932425358 18326.8582567456 7514.56253215238 7188.63494843413 7500.49610683739 up 16381.0 17188.0 17262.0 7945.0 8673.0 7001.0

ADAMTS1 1.25729694813847 4.81543041799804e-156 1.01966739101109e-153 14776.2007095681 15155.1403256116 14402.859408586 6223.51434380902 6066.36909807326 6258.80563578689 up 13870.0 14722.0 13566.0 6580.0 7319.0 5842.0

TENM1 -5.5329707319776 1.2687768551021e-155 2.65346665746043e-153 19.1760355279182 13.3824768532095 30.7889520012526 922.177277388114 1041.86718899824 957.783676548011 down 18.0 13.0 29.0 975.0 1257.0 894.0

S100A2 1.59997471574911 1.60515757649469e-155 3.31602065192928e-153 7527.65928001501 6931.09358866613 7793.85160831709 2563.17991971466 2399.52705819403 2377.31764906044 up 7066.0 6733.0 7341.0 2710.0 2895.0 2219.0

ANKRD33B 1.6679301845506 2.89644708766247e-153 5.91154381506051e-151 3206.65927439077 3394.00201423321 3298.66461613421 992.168168184751 1037.72292810326 1087.41659026424 up 3010.0 3297.0 3107.0 1049.0 1252.0 1015.0

TNFRSF19 2.66316110229899 7.51852977101031e-153 1.51623683715375e-150 1325.27712204057 1153.98127326522 1218.81782404959 201.46026675248 176.545514126193 207.841200503707 up 1244.0 1121.0 1148.0 213.0 213.0 194.0

MFGE8 -1.58395839526181 3.86842283275392e-151 7.70953915139429e-149 2569.58876074104 2791.79055583879 2728.53816011101 8525.64715730919 7548.35679411849 8184.01510643205 down 2412.0 2712.0 2570.0 9014.0 9107.0 7639.0

PYCR1 1.74441723199012 1.88915025607702e-148 3.72118666720287e-146 4893.08506554047 5568.13979223156 5195.90107221139 1671.26897348184 1471.21261771827 1532.02534391908 up 4593.0 5409.0 4894.0 1767.0 1775.0 1430.0

GSN 1.63339529530222 2.52661790124112e-145 4.91964451115224e-143 5214.81632828665 5868.73081078057 5512.28409622427 1881.24164587175 1777.88792394687 1688.44191749403 up 4895.0 5701.0 5192.0 1989.0 2145.0 1576.0

CA12 -3.63146069939776 1.56259725941962e-144 3.00799972438277e-142 79.9001480329926 72.0594907480513 49.8993360020301 790.707901432271 870.294787946021 841.006919563969 down 75.0 70.0 47.0 836.0 1050.0 785.0

MFAP5 -8.42623622144847 3.42227042697596e-144 6.5138495542666e-142 6.39201184263941 5.14710648200366 9.55519200038875 2341.85737314151 2387.09427550909 2510.16460195972 down 6.0 5.0 9.0 2476.0 2880.0 2343.0

ADD2 4.36598384392214 4.5342561846691e-144 8.53447775203274e-142 670.095908170032 706.183009330902 696.467328028335 40.6703824899374 28.1809740858712 32.1403918304702 up 629.0 686.0 656.0 43.0 34.0 30.0

SLC7A5 -1.68533807133454 3.10237890862861e-143 5.77519766067788e-141 21613.5227105781 25971.2698868941 23141.6133369415 80356.1633944788 71015.2258442163 76102.0197761872 down 20288.0 25229.0 21797.0 84959.0 85679.0 71034.0

DUSP4 -4.77334202552094 3.69153636670848e-143 6.79724196217843e-141 34.0907298274102 32.9414814848234 19.1103840007775 831.378283922208 734.363030590642 793.867678212613 down 32.0 32.0 18.0 879.0 886.0 741.0

TINAGL1 1.6224057909911 5.6062837758949e-142 1.02118760391032e-139 8536.53181584493 9970.97467693749 9123.08498437117 3108.9197033587 2840.47641742001 3027.62491043029 up 8013.0 9686.0 8593.0 3287.0 3427.0 2826.0

MGAT3 -3.22318629810224 4.03150291545167e-141 7.26528291359056e-139 82.0308186472058 84.41254630486 97.6752960039739 843.673980954049 790.724978762385 832.436148409177 down 77.0 82.0 92.0 892.0 954.0 777.0

ADGRG1 1.45075779197029 6.60274194592673e-141 1.17737314277894e-138 16693.8042623599 18868.262941729 17339.4884167055 6728.58374496313 6197.32774235467 6429.14971248838 up 15670.0 18329.0 16332.0 7114.0 7477.0 6001.0

FGFR2 1.62921025368953 1.41478554252406e-139 2.49650698857891e-137 2485.42727147962 2462.37574099055 2561.85314410423 806.786889858525 799.013500552347 822.794030860036 up 2333.0 2392.0 2413.0 853.0 964.0 768.0

SPEG 2.84466699448171 2.91346068616128e-139 5.08804371377032e-137 1475.4894003426 1944.57682890098 1509.72033606142 251.58887772845 232.907462297935 200.341775743264 up 1385.0 1889.0 1422.0 266.0 281.0 187.0

MIEN1 -1.34458625914478 1.81057493763744e-138 3.12970810648757e-136 3784.07101084253 4092.97907448931 4150.13839216885 10370.0017120854 9892.35075631977 10284.9253857505 down 3552.0 3976.0 3909.0 10964.0 11935.0 9600.0

LMCD1 2.65744265331838 9.87879700420642e-138 1.69037193183088e-135 949.213758631953 995.450393619508 1011.78866404116 174.977226991591 145.049131324337 148.917148814512 up 891.0 967.0 953.0 185.0 175.0 139.0

ANXA3 -1.69634915556917 1.26575805745507e-137 2.14419414932889e-135 972.651135388297 960.450069541883 1080.79838404397 3103.24476626708 3298.83167240492 3365.09902465023 down 913.0 933.0 1018.0 3281.0 3980.0 3141.0

PSG1 2.18023258583617 6.34469913166748e-137 1.06415052762819e-134 1785.50197471061 1543.1025233047 1639.24627206669 387.78736792731 338.171689030454 371.757198839105 up 1676.0 1499.0 1544.0 410.0 408.0 347.0

ATP6V1B2 1.63892992403845 2.48727576706455e-136 4.13082857785034e-134 3154.45784434255 3013.11613456494 3284.86267213364 998.788928124973 971.414753783559 1068.13235516596 up 2961.0 2927.0 3094.0 1056.0 1172.0 997.0

TPD52L1 -1.86237564748058 1.27453366162657e-135 2.09617477941302e-133 553.974359695416 506.47527782916 502.178424020431 1851.92113756505 1936.19869013515 1891.99773242034 down 520.0 492.0 473.0 1958.0 2336.0 1766.0

SHISA9 3.41646770465486 5.05595462083883e-135 8.23537223817402e-133 844.810898535509 851.331412123406 789.895872032137 87.9615249200971 90.3448875105869 51.4246269287523 up 793.0 827.0 744.0 93.0 109.0 48.0

HBEGF 2.30417591249117 5.32906902808871e-135 8.59756469864979e-133 1325.27712204057 1324.86520846774 1479.99307206021 252.534700577053 282.638593037708 302.119683206419 up 1244.0 1287.0 1394.0 267.0 341.0 282.0

FGF2 -1.55664313817435 9.35630620383343e-135 1.49524365182017e-132 1377.47855208879 1262.0705093873 1285.70416805231 3670.738475429 3978.49045918181 3895.41548985298 down 1293.0 1226.0 1211.0 3881.0 4800.0 3636.0

MYO10 -1.50313469342661 1.43127626820393e-134 2.26596448442753e-132 1413.69995253042 1340.30652791375 1313.30805605343 3811.66607987087 3730.66365766194 3989.6939725557 down 1327.0 1302.0 1237.0 4030.0 4501.0 3724.0

COL4A4 -4.75056530938429 9.91744966239089e-134 1.55557034519353e-131 27.6987179847708 29.8532175956212 22.2954480009071 746.25422754792 738.507291485624 665.306110890732 down 26.0 29.0 21.0 789.0 891.0 621.0

PTPRB -2.23173902918648 1.13228281813812e-133 1.75971293020731e-131 253.549803091363 270.737800953393 253.743432010323 1212.5448919093 1249.08023374729 1192.40853691044 down 238.0 263.0 239.0 1282.0 1507.0 1113.0

FAM101B 1.52756274577404 1.30423044998134e-133 2.00851489297126e-131 3789.39768737806 3622.53354203418 3607.61582414677 1198.35754918025 1305.44218191903 1318.82741144363 up 3557.0 3519.0 3398.0 1267.0 1575.0 1231.0

AKR1C2 -5.45322452437821 1.48843675103926e-133 2.27154221284731e-131 19.1760355279182 15.441319446011 20.1720720008207 765.170684519984 851.231187829108 778.868828691727 down 18.0 15.0 19.0 809.0 1027.0 727.0

IGFBP3 -1.11174538820714 2.29557453710162e-133 3.47205648736621e-131 9422.8907913576 9744.50199172933 9712.32182439514 20374.91580461 21013.8892940909 21023.0302963105 down 8845.0 9466.0 9148.0 21542.0 25353.0 19623.0

CADM1 1.56509409815016 4.23994943535842e-132 6.35617198539572e-130 3588.04931433492 3398.11969941882 3573.64180814539 1129.31248123221 1172.82583327964 1269.54547730357 up 3368.0 3301.0 3366.0 1194.0 1415.0 1185.0

MYEOV -5.76984966660626 2.98147604826066e-131 4.43036879452067e-129 8.52268245685255 17.5001620388124 21.2337600008639 941.093734360178 832.996439891192 807.79518133915 down 8.0 17.0 20.0 995.0 1005.0 754.0

GTPBP6 -2.02661539185977 5.49097987708342e-130 8.08845209719941e-128 511.360947411153 561.034606538399 542.522568022072 2420.36066957557 2017.42620367678 2144.83548148671 down 480.0 545.0 511.0 2559.0 2434.0 2002.0

SLC44A2 1.72376385943516 1.80258491185105e-129 2.63239555230661e-127 2040.11711310908 2146.34340299553 2080.90848008466 630.863840018331 604.233238488237 665.306110890732 up 1915.0 2085.0 1960.0 667.0 729.0 621.0

STEAP3 2.0734551622914 2.35053981880159e-129 3.40326021628196e-127 1493.60010056341 1478.24898163145 1490.60995206064 366.033442409436 378.785445801268 311.761800755561 up 1402.0 1436.0 1404.0 387.0 457.0 291.0

PPP1R1B -10.1255561181186 5.01877961092696e-127 7.20492598382227e-125 2.13067061421314 3.0882638892022 7.43181600030236 4693.17297476905 4310.03133078029 5103.89422267866 down 2.0 3.0 7.0 4962.0 5200.0 4764.0

IGFBP7 -1.80536733581163 1.79726261177753e-126 2.55845618853037e-124 611.50246627917 592.946666726822 656.123184026694 2114.85988947674 2082.07667363848 2308.75147982211 down 574.0 576.0 618.0 2236.0 2512.0 2155.0

DPYD -1.88525059540605 1.02345767131675e-125 1.44478107934215e-123 525.210306403538 503.387013939958 495.808296020172 1763.95961264496 1864.08855056248 2005.56045022134 down 493.0 489.0 467.0 1865.0 2249.0 1872.0

CDH6 -1.44144012503556 1.20482150668392e-125 1.68675010935749e-123 1620.37500210909 1569.86747701112 1655.17159206734 4277.95674423225 4630.79712405183 4245.74576080511 down 1521.0 1525.0 1559.0 4523.0 5587.0 3963.0

SLC39A8 -2.11968219780802 1.27221096457209e-125 1.76649620818453e-123 288.70586822588 286.179120399404 317.444712012915 1256.99856579365 1341.91167779486 1277.04490206401 down 271.0 278.0 299.0 1329.0 1619.0 1192.0

PPME1 1.23258936125506 1.11765117986081e-123 1.53926918592213e-121 6830.92998916732 6596.53166733589 6859.56616827908 2932.0508306699 2783.28561706927 2920.49027099539 up 6412.0 6408.0 6461.0 3100.0 3358.0 2726.0

PCSK5 -2.7033276754753 1.42897306943721e-123 1.95216159647309e-121 151.277613609133 166.766250016919 124.217496005054 939.202088662972 1021.14588452333 920.286552745795 down 142.0 162.0 117.0 993.0 1232.0 859.0

FBLN2 -4.29517379761993 1.59581882431566e-123 2.16265367071258e-121 19.1760355279182 38.0885879668271 41.4058320016846 645.051182747379 643.189290901059 652.449954158544 down 18.0 37.0 39.0 682.0 776.0 609.0

LIMCH1 -1.61166958534774 1.80049331355741e-121 2.42066323267162e-119 774.498768266475 767.948287114946 788.834184032093 2257.67913961583 2462.51982379774 2403.02996252482 down 727.0 746.0 743.0 2387.0 2971.0 2243.0

SYNPO 1.81090223561204 1.92178713684766e-120 2.56339166127554e-118 11273.3782198017 13533.8017837804 11289.9901924593 3771.94152022954 3113.99763648876 3404.73884124114 up 10582.0 13147.0 10634.0 3988.0 3757.0 3178.0

TRPC4 3.09918419361724 9.08003428886935e-120 1.20168578791755e-117 721.232002911147 668.094421364075 679.480320027644 89.8531706173035 83.7140700786173 66.4234764496383 up 677.0 649.0 640.0 95.0 101.0 62.0

FBXL16 -3.33855947909792 3.35933920880301e-119 4.41141133311031e-117 78.8348127258861 87.5008101940622 60.5162160024621 772.73726730881 686.289604208862 841.006919563969 down 74.0 85.0 57.0 817.0 828.0 785.0

FGF18 -3.769671572695 6.98015428480129e-118 9.09567796804107e-116 36.2214004416233 47.3533796344337 52.0227120021165 646.942828444585 607.548647204222 595.668595258047 down 34.0 46.0 49.0 684.0 733.0 556.0

GRB7 -3.37441375456729 1.72090867321956e-117 2.22535823849919e-115 61.789447812181 67.9418055624483 64.7629680026348 719.771187787031 684.63189985087 611.738791173282 down 58.0 66.0 61.0 761.0 826.0 571.0

TSPAN1 -1.99576542893471 1.90109194262747e-116 2.43973465970526e-114 348.364645423848 307.796967623819 349.295352014211 1314.69375955844 1380.86773020769 1311.32798668318 down 327.0 299.0 329.0 1390.0 1666.0 1224.0

PSG3 4.20746385948575 7.79539923909001e-116 9.9288769255778e-114 559.301036230949 541.475601906785 552.077760022461 23.6455712150799 26.5232697278787 40.7111629852622 up 525.0 526.0 520.0 25.0 32.0 38.0

SLPI -1.88917430259654 4.07741232852552e-115 5.15457946606137e-113 378.194034022832 369.562245407863 409.811568016673 1417.78845005619 1395.78706942962 1475.24398501858 down 355.0 359.0 386.0 1499.0 1684.0 1377.0

SLC4A11 -3.21466428916177 4.14955223422885e-115 5.20691961835828e-113 66.0507890406073 70.0006481552498 73.2564720029804 658.292702627823 616.666021173181 669.591496468128 down 62.0 68.0 69.0 696.0 744.0 625.0

MISP -1.98184377975 4.87554884838011e-115 6.07292628614404e-113 743.604044360385 928.53800935346 731.503032029761 3457.92833449328 3002.93144450327 3037.26702797943 down 698.0 902.0 689.0 3656.0 3623.0 2835.0

TFAP2A -1.57832351405818 1.83520775380289e-114 2.26922768973876e-112 809.654833400992 895.596527868637 805.821192032785 2451.57282357948 2454.23130200778 2596.94365990199 down 760.0 870.0 759.0 2592.0 2961.0 2424.0

ARID5B 1.56126943704275 5.75281973949871e-114 7.06179466573248e-112 2297.92825742887 2215.31462985438 2310.23308809399 724.500302030047 790.724978762385 797.08171739566 up 2157.0 2152.0 2176.0 766.0 954.0 744.0

DSC3 2.83429603496637 8.76237043161282e-114 1.06787449720519e-111 848.006904456829 766.918865818546 781.402368031791 99.3113991033354 101.119965837538 138.203684871022 up 796.0 745.0 736.0 105.0 122.0 129.0

HR -3.19660445218031 3.28925370023895e-113 3.97999697728913e-111 83.0961539543123 77.2065972300549 83.8733520034124 836.107398165224 655.622073586002 747.799783255606 down 78.0 75.0 79.0 884.0 791.0 698.0

PDXK -1.06596182857783 8.2826917820947e-113 9.95097863749533e-111 9565.64572250988 10065.6814362064 9923.59773640374 20489.360369291 20175.9197411258 21214.801300899 down 8979.0 9778.0 9347.0 21663.0 24342.0 19802.0

ZNF486 -3.28206490980458 1.56668935394309e-111 1.86899420111239e-109 75.6388068045664 66.9123842660476 53.0844000021597 622.351434380902 660.59518665998 619.238215933725 down 71.0 65.0 50.0 658.0 797.0 578.0

MXRA5 -4.21663386524312 3.15159239492158e-111 3.73342483706094e-109 27.6987179847708 30.882638892022 32.912328001339 541.010669401027 608.377499383218 550.672046695389 down 26.0 30.0 31.0 572.0 734.0 514.0

S1PR1 1.49776085165541 2.37381520145428e-110 2.79252982726636e-108 2316.03895764968 2380.02203727849 2433.388896099 860.698792228907 799.842352731343 866.719233028345 up 2174.0 2312.0 2292.0 910.0 965.0 809.0

MT2A -1.24946697378237 4.23584933296147e-110 4.94864053105981e-108 8650.52269370534 8137.57534804779 8951.09152836417 20364.5117532754 19323.8597011176 21511.5642521337 down 8120.0 7905.0 8431.0 21531.0 23314.0 20079.0

SIRPA 1.25279596172041 1.50879576084567e-109 1.75061645128258e-107 5347.98324167497 5730.78835706288 5485.74189622318 2356.04471587056 2221.32383970984 2377.31764906044 up 5020.0 5567.0 5167.0 2491.0 2680.0 2219.0

FBXL7 5.71839787775908 7.87188463492075e-109 9.07140991262296e-107 572.085059916227 665.006157474873 643.382928026176 8.51240563742875 10.7750783269507 17.1415423095841 up 537.0 646.0 606.0 9.0 13.0 16.0

GABRA3 3.60006832957721 9.33484188726845e-108 1.06846095655627e-105 501.772929647194 523.975439867973 544.645944022159 38.778736792731 44.7580176657954 46.0678949570072 up 471.0 509.0 513.0 41.0 54.0 43.0

NLRP1 1.77025104670539 3.2116061255354e-106 3.6513159574879e-104 1716.25517974868 1690.30976869 1615.88913606574 539.119023703821 479.905411638806 452.10817841528 up 1611.0 1642.0 1522.0 570.0 579.0 422.0

AMPH 2.0914263555676 1.37820254769114e-105 1.55645007719253e-103 985.435159073576 957.361805652681 966.136080039307 214.701786632925 237.051723192916 230.339474785036 up 925.0 930.0 910.0 227.0 286.0 215.0

DOK7 -2.53554067415476 3.06513662011277e-105 3.43863671157022e-103 143.820266459387 196.61946761254 142.266192005788 972.305888364084 890.18724024193 940.642134238427 down 135.0 191.0 134.0 1028.0 1074.0 878.0

TMEM54 1.71830184361529 7.75158620064501e-105 8.63893883150832e-103 3287.62475773087 3987.97810225644 3415.45029613896 1167.14539517634 1059.27308475716 1022.06446020895 up 3086.0 3874.0 3217.0 1234.0 1278.0 954.0

TRIM2 -2.41127405510293 5.31849332118948e-104 5.88858018699019e-102 157.669625451772 157.501458349312 145.451256005918 849.348918045669 830.509883354203 769.226711142586 down 148.0 153.0 137.0 898.0 1002.0 718.0

WNT7A -1.72417918877722 1.78606705031603e-103 1.96467375534763e-101 471.94354104821 480.739745419142 504.301800020517 1689.23960760531 1520.11489627905 1605.94824512916 down 443.0 467.0 475.0 1786.0 1834.0 1499.0

AUTS2 4.26022695904332 3.71503314599515e-103 4.06017170923599e-101 484.727564733489 459.121898194727 476.697912019394 26.4830397608894 24.0367131908901 23.5696206756781 up 455.0 446.0 449.0 28.0 29.0 22.0

TNFAIP2 1.31424455736729 7.77205622603897e-103 8.43965592750642e-101 6305.71968276378 7045.35935256661 6641.92012827022 2640.73739330012 2562.81093745628 2841.21063781356 up 5919.0 6844.0 6256.0 2792.0 3092.0 2652.0

CD9 1.50031881288707 2.81397784232512e-101 3.03622832159156e-99 2115.75591991365 2122.66671317831 2042.68771208311 781.249672946239 700.380091251798 740.300358495163 up 1986.0 2062.0 1924.0 826.0 845.0 691.0

JUP -1.64607095806154 6.72073400683981e-101 7.20564772632066e-99 674.357249398458 751.477546372535 678.418632027601 2323.88673901805 2034.8320994357 2231.61453942898 down 633.0 730.0 639.0 2457.0 2455.0 2083.0

SOX7 2.45459445568976 6.79275398102105e-101 7.23705990179224e-99 812.850839322312 1013.97997695472 869.522472035376 172.139758445781 164.941583620246 154.273880786257 up 763.0 985.0 819.0 182.0 199.0 144.0

FAT1 -1.04965967367369 3.35202546968342e-99 3.52691375505821e-97 4492.5189900684 4437.83520878356 4306.2065281752 9064.76618101302 9391.72404020606 8940.38566084245 down 4217.0 4311.0 4056.0 9584.0 11331.0 8345.0

PRR5L -1.38815035462806 6.26755875594413e-99 6.55385464973417e-97 887.424310819772 965.597176023887 899.249736036586 2386.31104702586 2409.47328434198 2409.45804089091 down 833.0 938.0 847.0 2523.0 2907.0 2249.0

VAV3 -4.72131401190014 6.34060401967649e-99 6.58956025112391e-97 20.2413708350248 20.5884259280146 16.9870080006911 532.498263763598 492.338194323749 501.390112555334 down 19.0 20.0 16.0 563.0 594.0 468.0

ZBED2 -1.96912031289472 9.37673372341993e-99 9.68547983382522e-97 255.680473705576 255.296481507382 263.298624010712 1024.32614503726 983.847536468502 1024.20715299765 down 240.0 248.0 248.0 1083.0 1187.0 956.0

SLC16A3 1.17974667256666 1.71791574005515e-97 1.76372682645662e-95 7559.61933922821 8336.25365825313 7631.41334431048 3519.40681965249 3356.02247275566 3512.94482707039 up 7096.0 8098.0 7188.0 3721.0 4049.0 3279.0

COL6A2 1.51668748551746 1.85065212562611e-97 1.88855704868111e-95 7686.39424077389 9135.0845842601 8158.01059233191 3186.47717694416 2710.34662531761 2834.78255944747 up 7215.0 8874.0 7684.0 3369.0 3270.0 2646.0

ADAM12 1.5394357492828 2.18082703263279e-97 2.21216825944907e-95 1827.05005168777 1696.48629646841 1801.6845360733 590.193457528393 629.098803858124 611.738791173282 up 1715.0 1648.0 1697.0 624.0 759.0 571.0

CHCHD7 2.15901458876284 3.68143862713855e-97 3.7121172823647e-95 958.801776395912 876.037523237023 989.493216040257 190.110392569242 220.474679612992 221.768703630244 up 900.0 851.0 932.0 201.0 266.0 207.0

CLIC3 -2.80853081855341 4.25117804573845e-96 4.26124000561002e-94 86.2921598756321 89.5596527868637 88.1201040035851 647.888651293188 571.079151328389 632.094372665913 down 81.0 87.0 83.0 685.0 689.0 590.0

ZSCAN31 -2.63829416631242 1.35163173239712e-95 1.34686126745925e-93 105.46819540355 121.471712975286 93.4285440038011 646.942828444585 688.776160745851 659.949378918987 down 99.0 118.0 88.0 684.0 831.0 616.0

MAL2 -1.66986339767728 1.57352406983943e-95 1.55880103760702e-93 488.988905961915 449.85710652712 513.856992020906 1480.212758064 1551.61127908091 1589.87804921392 down 459.0 437.0 484.0 1565.0 1872.0 1484.0

DAB2 1.74101622765969 4.64983943472257e-95 4.5795511641977e-93 1184.6528615025 1158.09895845082 1219.87951204963 352.791922528992 351.433323894393 362.115081289964 up 1112.0 1125.0 1149.0 373.0 424.0 338.0

AKAP17A -1.61683692061713 9.71468560899398e-95 9.51253030152359e-93 567.823718687801 627.946990804447 645.506304026262 1953.1241823656 1763.79743690394 1933.78024179995 down 533.0 610.0 608.0 2065.0 2128.0 1805.0

IFITM2 -1.5542948415936 1.05567713364062e-94 1.02776842780874e-92 572.085059916227 606.329143580031 586.051776023843 1745.9889785215 1645.27157530748 1794.50521053458 down 537.0 589.0 552.0 1846.0 1985.0 1675.0

SH3BP4 1.09465903578676 2.32864609807781e-94 2.25412942293932e-92 4823.83827057854 5062.6939356988 4906.0602481996 2325.77838471526 2310.01102286244 2290.53859111817 up 4528.0 4918.0 4621.0 2459.0 2787.0 2138.0

SLC4A4 2.3904980730355 7.00759123301162e-94 6.74480656177369e-92 702.055967383229 669.123842660476 710.269272028897 139.981781593273 121.012418133447 137.132338476673 up 659.0 650.0 669.0 148.0 146.0 128.0

ARL2BP -1.25010609046201 4.74129235914836e-93 4.53771144429228e-91 1334.86513980453 1384.57164365898 1464.06775205957 3389.82908939385 3260.70447217109 3300.81824098929 down 1253.0 1345.0 1379.0 3584.0 3934.0 3081.0

AFAP1 -1.46509937824227 6.5274332206282e-93 6.21206285154167e-91 694.598620233483 694.859375070494 750.613416030538 1891.64569720639 2011.6242384238 2004.48910382699 down 652.0 675.0 707.0 2000.0 2427.0 1871.0

FAM198B -2.61374384345452 8.01278366751748e-93 7.58304778367297e-91 90.5535011040583 127.648240753691 109.353864004449 676.263336751284 660.59518665998 670.662842862477 down 85.0 124.0 103.0 715.0 797.0 626.0

SALL1 -2.51922665630771 1.08560763291707e-92 1.02167740564529e-90 108.66420132487 114.265763900481 119.970744004881 683.82991954011 651.477812691021 629.951679877215 down 102.0 111.0 113.0 723.0 786.0 588.0

RHOB 1.37386936544417 1.24973736003342e-92 1.16964369497051e-90 9324.8799431038 10737.893542756 9779.20816839786 4157.83724245964 3558.26240443073 3801.13700715027 up 8753.0 10431.0 9211.0 4396.0 4293.0 3548.0

C3 -1.13204170456137 1.82677041831779e-92 1.70030169704964e-90 1963.41297099741 2092.81349558269 2061.79809608388 4545.62461038695 4399.54736611188 4465.37177164665 down 1843.0 2033.0 1942.0 4806.0 5308.0 4168.0

DPYSL3 -1.06300071225638 4.27812349185729e-92 3.96018644546789e-90 3405.8769768197 3232.3828706983 3473.84313614133 7089.88807312954 6906.82520757542 7130.88160078698 down 3197.0 3140.0 3272.0 7496.0 8333.0 6656.0

CCND1 -1.25986581557354 5.90335377277027e-92 5.43493548427872e-90 1413.69995253042 1408.2483334762 1343.03532005464 3433.3369404296 3374.25722069357 3163.68590251261 down 1327.0 1368.0 1265.0 3630.0 4071.0 2953.0

PDLIM1 1.51542716127748 1.34359295389647e-91 1.22510645921766e-89 1978.3276652969 2015.60689835263 2213.61948009006 715.987896392618 692.091569461836 766.012671959539 up 1857.0 1958.0 2085.0 757.0 835.0 715.0

LFNG 1.71240159738367 1.34515821378091e-91 1.22510645921766e-89 3251.40335728925 4165.03856523736 3659.63853614889 1223.89476609253 1065.07505001013 1091.70197584164 up 3052.0 4046.0 3447.0 1294.0 1285.0 1019.0

ARMCX3 1.78806654374989 2.11405932027669e-91 1.91508903130947e-89 1225.13560317255 1129.2751621516 1268.71716005162 341.442048345753 365.523810937329 340.688153402984 up 1150.0 1097.0 1195.0 361.0 441.0 318.0

APBA1 1.79823416957071 5.80852786580069e-91 5.23385436418424e-89 1095.16469570555 1042.80377325394 1051.07112004276 293.20508306699 309.990714944583 313.904493544259 up 1028.0 1013.0 990.0 310.0 374.0 293.0

H1F0 1.93185699911856 1.25854379785882e-90 1.12802814474754e-88 921.515040647182 932.655694539063 965.074392039264 229.834952210576 256.944175488825 251.766402672016 up 865.0 906.0 909.0 243.0 310.0 235.0

KDELC1 1.29520098098053 1.488583919181e-90 1.32719008373295e-88 2463.05523003039 2409.87525487411 2498.15186410164 1040.40513346351 994.622614795453 967.425794097152 up 2312.0 2341.0 2353.0 1100.0 1200.0 903.0

KRT19 -8.87694192588862 3.67420619197403e-90 3.25869386869319e-88 0 5.14710648200366 6.37012800025917 1868.94594883991 1787.83415009483 1782.72040019674 down 0.0 5.0 6.0 1976.0 2157.0 1664.0

FAM127C 2.91728107562439 5.05542849777817e-90 4.46036243501886e-88 490.054241269022 553.828657463594 526.597248021424 72.828359342446 62.1639134247158 73.9229012100814 up 460.0 538.0 496.0 77.0 75.0 69.0

COL9A2 2.34583889691468 5.66320827061496e-90 4.97071233700609e-88 708.447979225868 850.301990827005 824.931576033562 142.819250139082 156.653061830284 170.344076701492 up 665.0 826.0 777.0 151.0 189.0 159.0

TCEAL8 2.4580777490101 8.70237549990997e-90 7.59887840043685e-88 649.854537335007 632.06467599005 615.779040025053 122.011147469812 103.606522374526 121.062142561438 up 610.0 614.0 580.0 129.0 125.0 113.0

CPT1A -1.88718258983811 2.32690869696054e-89 2.02142735007752e-87 281.248521076134 319.120601884227 315.321336012829 1077.29222455904 1209.29532915547 1099.20140060208 down 264.0 310.0 297.0 1139.0 1459.0 1026.0

MYEF2 2.51403470595982 3.49885262103425e-89 3.02400833675103e-87 662.638561020286 678.388634328083 599.853720024405 104.986336194955 107.750783269507 128.561567321881 up 622.0 659.0 565.0 111.0 130.0 120.0

F3 -1.10792177757727 1.22794819419716e-88 1.05591078221827e-86 1985.78501244664 1893.10576408095 1985.35656008077 4143.64989973059 4287.6523219474 4206.10594421419 down 1864.0 1839.0 1870.0 4381.0 5173.0 3926.0

FGFRL1 -1.59740413487857 2.1641284532583e-88 1.85153212112099e-86 1188.91420273093 1413.39543995821 1130.697720046 4075.55065463116 3602.19156991753 3621.15081289964 down 1116.0 1373.0 1065.0 4309.0 4346.0 3380.0

ABLIM3 2.37489583285142 8.59725412251052e-88 7.31846657463961e-86 642.397190185261 673.241527846079 663.555000026996 124.848616015622 115.210452880473 143.560416842767 up 603.0 654.0 625.0 132.0 139.0 134.0

TENM4 3.29181206204987 1.65587297771376e-87 1.40252441212355e-85 434.65680529948 468.386689862333 497.931672020258 56.7493709161917 38.9560524128219 48.2105877457052 up 408.0 455.0 469.0 60.0 47.0 45.0

PDZK1 1.33336996801343 8.07293246357502e-87 6.80375502154034e-85 2000.69970674614 1972.3712039038 1928.02540807844 798.274484221096 769.174822108483 774.583443114331 up 1878.0 1916.0 1816.0 844.0 928.0 723.0

GPAT3 -2.31221313410533 1.44627413215302e-86 1.21286553458773e-84 153.408284223346 134.854189828496 127.402560005183 679.100805297094 702.866647788787 681.376306805967 down 144.0 131.0 120.0 718.0 848.0 636.0

CALD1 -1.30195377592759 2.77746971654039e-86 2.31630391165966e-84 1143.10478452535 1093.24541677758 1190.15224804842 2730.59056391742 2965.63309644844 2748.0035015052 down 1073.0 1062.0 1121.0 2887.0 3578.0 2565.0

PAPSS2 1.57700041128475 2.78940966929499e-86 2.31630391165966e-84 1347.64916348981 1318.68868068934 1370.63920805576 424.674459022834 462.499515879885 466.035681541817 up 1265.0 1281.0 1291.0 449.0 558.0 435.0

JAG1 -1.4173015172624 4.5817884691283e-86 3.78612178863578e-84 615.763807507597 617.652777840439 607.285536024707 1645.73175656956 1620.40600993759 1650.94479369182 down 578.0 600.0 572.0 1740.0 1955.0 1541.0

FOS -1.46799951129729 6.54879027985565e-86 5.38526734663858e-84 577.41173645176 557.946342649197 607.285536024707 1602.22390553381 1578.96340098778 1640.23132974833 down 542.0 542.0 572.0 1694.0 1905.0 1531.0

ALDH4A1 1.48093662788693 2.73035192114288e-85 2.23440393933142e-83 1675.77243807863 1901.34113445215 1811.23972807369 656.401056930617 645.675847438048 627.808987088517 up 1573.0 1847.0 1706.0 694.0 779.0 586.0

NGEF -1.74426796047919 3.13736040692059e-85 2.55513871602091e-83 303.620562525372 313.973495402223 318.506400012958 1044.18842485793 1026.11899759731 1067.06100877161 down 285.0 305.0 300.0 1104.0 1238.0 996.0

TACSTD2 -5.43602851489316 3.98881320289863e-85 3.23303806971784e-83 50.0707594340087 53.5299074128381 23.3571360009503 1804.62999513489 1888.12526375337 1808.43271366112 down 47.0 52.0 22.0 1908.0 2278.0 1688.0

FLRT2 -2.76389021775215 6.1881003042656e-85 4.99173424544092e-83 109.729536631977 73.088912044452 80.6882880032828 609.109914500457 563.619481717423 616.024176750678 down 103.0 71.0 76.0 644.0 680.0 575.0

HSPB1 -1.16927643407772 6.84431654846286e-85 5.49491575028251e-83 3119.30177920803 3354.88400496999 3233.90164813157 7446.46328705295 6762.60492843008 7631.20036694796 down 2928.0 3259.0 3046.0 7873.0 8159.0 7123.0

MEGF6 -1.50717956402942 1.03754111320126e-84 8.29054078190064e-83 1857.94477559386 2200.90273170477 1736.92156807067 5839.51026727612 5443.90111164711 5192.81597340963 down 1744.0 2138.0 1636.0 6174.0 6568.0 4847.0

IL18 -4.31226035616347 1.15492037886362e-84 9.18514141687782e-83 15.9800296065985 23.6766898172168 25.4805120010367 427.511927568644 470.788037669848 395.326819514783 down 15.0 23.0 24.0 452.0 568.0 369.0

SORL1 -1.86454804274321 2.31982318809311e-84 1.83634601898585e-82 365.410010337553 453.974791712723 335.493408013649 1380.90135896066 1441.37393927441 1385.25088789326 down 343.0 441.0 316.0 1460.0 1739.0 1293.0

EPAS1 1.21004991963413 8.92447052571958e-84 7.03165259096231e-82 2664.40360307353 2640.46562526788 2746.58685611174 1169.98286372215 1117.29273728689 1195.62257609349 up 2501.0 2565.0 2587.0 1237.0 1348.0 1116.0

RNASET2 -1.3631140880386 2.87429046760362e-83 2.25418891301877e-81 780.890780109115 896.625949165038 876.954288035679 2183.90495742478 2200.60253523494 2187.68933726067 down 733.0 871.0 826.0 2309.0 2655.0 2042.0

THBD -2.09662368429663 5.39091277035517e-83 4.20838996911597e-81 169.388313829944 197.648888908941 179.4252720073 818.136764041763 761.715152497517 758.513247199096 down 159.0 192.0 169.0 865.0 919.0 708.0

CKB -1.23312172887628 8.97902407347497e-83 6.97727833966358e-81 1204.89423233753 1293.98256957572 1202.89250404894 2981.23361879727 2782.45676489028 2941.91719888237 down 1131.0 1257.0 1133.0 3152.0 3357.0 2746.0

THOC6 -1.38284301735031 6.27865249580776e-82 4.85663804926865e-80 780.890780109115 821.478194527784 869.522472035376 2236.87103694655 2034.8320994357 2176.97587331718 down 733.0 798.0 819.0 2365.0 2455.0 2032.0

UGCG 1.19755579473394 2.08321440269656e-81 1.60407509007635e-79 3853.31780580446 3586.50379666015 3666.00866414915 1518.04567200813 1695.00270604725 1627.37517301614 up 3617.0 3484.0 3453.0 1605.0 2045.0 1519.0

HLA-B -1.08982202430389 6.30422921434337e-81 4.83229153352836e-79 3732.93491610142 4117.68518560293 4055.648160165 8796.15249200971 8079.65104085506 8470.06459372323 down 3504.0 4000.0 3820.0 9300.0 9748.0 7906.0

ASRGL1 -1.458337550724 1.48336291822992e-80 1.13189945201869e-78 547.582347852776 617.652777840439 564.818016022979 1623.03200820308 1564.04406176585 1568.45112132694 down 514.0 600.0 532.0 1716.0 1887.0 1464.0

ITGB6 -2.88646922439726 3.31303187990108e-80 2.51671569710871e-78 78.8348127258861 60.7358564876432 106.168800004319 550.468897887059 655.622073586002 607.453405595886 down 74.0 59.0 100.0 582.0 791.0 567.0

ZBED6CL -1.50584269443308 1.09382761222588e-79 8.27207131745824e-78 450.636834906078 468.386689862333 469.266096019092 1351.58085065396 1302.12677320305 1288.82971240185 down 423.0 455.0 442.0 1429.0 1571.0 1203.0

SDC1 -1.06135287369102 1.40963276589925e-78 1.05660084311209e-76 2143.45463789842 2330.60981505126 2186.01559208894 4632.64031245845 4546.25420179421 4723.56625268476 down 2012.0 2264.0 2059.0 4898.0 5485.0 4409.0

GNG11 -1.28175359676627 1.66503482959442e-78 1.24254141027883e-76 1207.02490295174 1072.65699084956 1205.01588004903 2820.44373453473 2709.51777313861 2944.05989167107 down 1133.0 1042.0 1135.0 2982.0 3269.0 2748.0

WNT7B -1.15821564632495 1.74233828446218e-78 1.29452677801707e-76 2630.31287324612 3018.26324104695 2786.93100011339 6499.69461560115 5994.25895850059 6337.01392257437 down 2469.0 2932.0 2625.0 6872.0 7232.0 5915.0

COL1A2 -4.59465896087248 2.04337393862316e-78 1.51156133276316e-76 56.4627712766481 50.4416435236359 26.5422000010799 1026.21779073447 1049.3268586092 1150.62602753083 down 53.0 49.0 25.0 1085.0 1266.0 1074.0

PLCD3 -1.27953149142287 5.32948128545782e-78 3.9252788250285e-76 2531.23668968521 3072.82256975619 2743.40179211161 7112.58782149602 6545.44565753307 6609.13590673901 down 2376.0 2985.0 2584.0 7520.0 7897.0 6169.0

TNFRSF11B -2.55329695953496 7.41545653252851e-78 5.43800145718758e-76 95.8801776395912 89.5596527868637 81.749976003326 533.444086612202 507.257533545681 528.17377241406 down 90.0 87.0 77.0 564.0 612.0 493.0

OLFML2A 1.13252018674655 1.07445126959896e-77 7.84534677026139e-76 2959.50148314205 3177.82354198906 3032.18092812336 1380.90135896066 1425.62574787348 1374.53742394977 up 2778.0 3087.0 2856.0 1460.0 1720.0 1283.0

CRIP2 -1.39291810109912 2.40094568876054e-77 1.74558025612033e-75 5063.53871467752 6121.96844969515 5191.65432021122 15624.0476360762 13105.8106542881 14283.1901294609 down 4753.0 5947.0 4890.0 16519.0 15812.0 13332.0

LHX2 3.68661361788637 4.92526671632333e-77 3.55038375210711e-75 343.037968888315 370.591666704264 362.035608014729 23.6455712150799 29.0098262648674 31.0690454361212 up 322.0 360.0 341.0 25.0 35.0 29.0

COL5A1 1.1022173052821 6.01753671753924e-77 4.31936745742011e-75 45957.4998132703 53588.5844267329 46805.5771699043 23260.6213156984 22236.4462581103 22675.0464363967 up 43139.0 52057.0 44086.0 24593.0 26828.0 21165.0

MAP3K6 -1.38228658856384 7.32627495477761e-77 5.23658640227564e-75 1099.42603693398 1268.2470371657 1033.02242404203 3023.79564698441 2841.30526959901 3004.05528975461 down 1032.0 1232.0 973.0 3197.0 3428.0 2804.0

COL5A2 -3.10102292237665 8.27386285331855e-77 5.8890435603032e-75 44.7440828984759 57.647592598441 79.6266000032396 477.640538544613 585.169638371325 497.104726977938 down 42.0 56.0 75.0 505.0 706.0 464.0

IFITM3 -1.01137197153932 1.22476836169825e-76 8.68099416199516e-75 2180.74137364715 2299.72717615924 2256.08700009179 4626.96537536683 4453.42275774664 4500.72620266017 down 2047.0 2234.0 2125.0 4892.0 5373.0 4201.0

LAMA3 -1.10220404919452 1.19349838912393e-75 8.42410946323306e-74 1519.16814793397 1496.77856496666 1576.60668006414 3193.09793688438 3402.43819477944 3261.17842439837 down 1426.0 1454.0 1485.0 3376.0 4105.0 3044.0

DMTN 1.80971491513692 1.33437869542539e-75 9.37940875539673e-74 1397.71992292382 1521.48467608028 1367.45414405563 470.073955755788 343.973654283427 411.397015430018 up 1312.0 1478.0 1288.0 497.0 415.0 384.0

TRPM2 1.66243291745502 1.49348054259459e-75 1.04113417249186e-73 1493.60010056341 1584.27937516073 1454.51256005918 550.468897887059 435.14739397301 446.751446443535 up 1402.0 1539.0 1370.0 582.0 525.0 417.0

SCN8A 1.60040644762311 4.21471993273955e-75 2.92612113363148e-73 1029.11390666495 1109.71615751999 1102.03214404484 343.33369404296 367.181515295321 357.829695712568 up 966.0 1078.0 1038.0 363.0 443.0 334.0

NUDT21 -1.1389733591413 5.00352070657911e-75 3.45957717426327e-73 2667.59960899485 2360.46303264688 2754.01867211205 5618.18772070297 5746.43215698073 5771.34302635809 down 2504.0 2293.0 2594.0 5940.0 6933.0 5387.0

LZTS1 3.96213474388669 5.32142849675964e-75 3.66443084289058e-73 336.645957045676 437.504050970311 348.233664014168 27.4288626094926 25.6944175488825 18.2128887039331 up 316.0 425.0 328.0 29.0 31.0 17.0

ARFGEF3 2.29350670379133 5.91482823601525e-75 4.05656640963961e-73 556.105030309629 621.770463026042 583.928400023757 129.577730258638 107.750783269507 123.204835350136 up 522.0 604.0 550.0 137.0 130.0 115.0

SLCO4C1 4.17928181929951 8.66832581299023e-75 5.92102577709898e-73 355.821992573594 333.532500033837 328.061592013347 20.8081026692703 20.7213044749053 13.9275031265371 up 334.0 324.0 309.0 22.0 25.0 13.0

SLC2A6 2.21111427758921 8.93331340235326e-75 6.07752325445238e-73 566.758383380694 628.976412100847 628.519296025571 122.011147469812 135.931757355379 136.060992082324 up 532.0 611.0 592.0 129.0 164.0 127.0

NT5C 2.12813255588768 9.27731324242338e-75 6.28630745306608e-73 627.482495885769 706.183009330902 734.688096029891 146.602541533495 167.428140157235 158.559266363653 up 589.0 686.0 692.0 155.0 202.0 148.0

CPNE2 -1.0801590992095 1.15149331839202e-74 7.77143299345051e-73 2001.76504205324 2166.93182892354 2221.05129609036 4604.26562700035 4373.852948563 4533.93794088499 down 1879.0 2105.0 2092.0 4868.0 5277.0 4232.0

LRRC4B 4.39995393876484 1.26955233354323e-74 8.53421290881838e-73 343.037968888315 390.150671335878 315.321336012829 15.1331655776511 18.2347479379166 16.0701959152351 up 322.0 379.0 297.0 16.0 22.0 15.0

KRT81 1.43357191107212 2.42813660038743e-74 1.61939503978595e-72 4509.5643549821 5182.10680608129 4618.3428001879 1997.57785624995 1698.31811476324 1600.59151315741 up 4233.0 5034.0 4350.0 2112.0 2049.0 1494.0

FMN2 3.86262638101212 8.83869300837496e-74 5.87166508085772e-72 340.907298274102 359.268032443856 320.629776013045 25.5372169122862 25.6944175488825 18.2128887039331 up 320.0 349.0 302.0 27.0 31.0 17.0

SFRP1 -2.07485939162073 1.66208721990364e-73 1.09983427754561e-71 168.322978522838 156.472037052911 209.152536008509 746.25422754792 733.534178411646 769.226711142586 down 158.0 152.0 197.0 789.0 885.0 718.0

ROR2 5.2488887790112 2.58232802558025e-73 1.70212594370932e-71 367.540680951766 378.827037075469 358.8505440146 4.72911424301597 12.4327826849432 11.7848103378391 up 345.0 368.0 338.0 5.0 15.0 11.0

ARL4C 1.00430126225994 2.73912622979252e-72 1.79848055553044e-70 3928.95661260902 3882.97713002356 3974.95987216172 1911.50797702706 1934.54098577716 2032.34411008006 up 3688.0 3772.0 3744.0 2021.0 2334.0 1897.0

SEMA7A 1.51504367133268 3.73050074528419e-72 2.43994913610479e-70 2193.52539733242 2568.40613451983 2261.395440092 917.448163145099 752.597778528559 788.510946240868 up 2059.0 2495.0 2130.0 970.0 908.0 736.0

NRP2 -1.1987735050829 4.58897393611551e-72 2.9898930183768e-70 1049.35527749997 1090.15715288838 1087.16851204423 2496.02649746383 2333.21888387433 2581.9448103811 down 985.0 1059.0 1024.0 2639.0 2815.0 2410.0

PYGL -1.09490640358612 1.13708136492785e-71 7.35196882514421e-70 1950.62894731213 1767.51636592006 1957.75267207965 3912.86912467142 4047.2851900385 4163.25208844023 down 1831.0 1717.0 1844.0 4137.0 4883.0 3886.0

KCTD1 -1.38421498245596 2.75302313088863e-71 1.77323999381192e-69 580.60774237308 636.182361175653 610.470600024837 1555.87858595225 1532.54767896399 1685.22787831099 down 545.0 618.0 575.0 1645.0 1849.0 1573.0

PORCN -1.62243159444815 2.80192727474468e-71 1.79790333462784e-69 467.682199819784 551.769814870793 611.53228802488 1765.85125834216 1563.21520958685 1694.86999586013 down 439.0 536.0 576.0 1867.0 1886.0 1582.0

SIAE 1.61528705567875 2.97710407950943e-71 1.90309973988263e-69 1045.09393627154 973.832546395093 1100.97045604479 346.171162588769 329.883167240492 342.830846191682 up 981.0 946.0 1037.0 366.0 398.0 320.0

TM6SF2 -1.4849588433613 4.43284030876609e-71 2.82301935452999e-69 438.918146527906 504.416435236359 453.340776018444 1281.58995985733 1348.54249522683 1279.18759485271 down 412.0 490.0 427.0 1355.0 1627.0 1194.0

PSG5 1.61152940678835 6.29258844033884e-71 3.97747940967686e-69 958.801776395912 961.479490838284 973.567896039609 335.767111254134 311.648419302575 298.905644023372 up 900.0 934.0 917.0 355.0 376.0 279.0

ITPRIPL2 -1.0436314517862 7.64974270300334e-71 4.81734726352701e-69 2984.0041952055 3420.76696793963 3107.56077612643 6555.49816366874 6517.2646834472 6538.42704471198 down 2801.0 3323.0 2927.0 6931.0 7863.0 6103.0

FLJ26245 -1.01336012190148 1.14288705982787e-70 7.17055807166076e-69 4314.6079937816 3927.24224576879 4386.89481617848 8175.69270332601 8695.48820984924 8618.98174253775 down 4050.0 3815.0 4132.0 8644.0 10491.0 8045.0

TLL2 2.08351786252054 3.06358219995074e-70 1.91502149325334e-68 660.507890406073 786.477870450159 683.727072027817 157.00659286813 159.968470546269 187.485619011076 up 620.0 764.0 644.0 166.0 193.0 175.0

ZBED1 -1.3960047144301 4.97282966256613e-70 3.08570455984873e-68 573.150395223334 628.976412100847 558.44788802272 1617.35707111146 1481.98769604522 1536.31072949647 down 538.0 611.0 526.0 1710.0 1788.0 1434.0

UNG -1.16775023406803 9.37484225587247e-70 5.79597911731678e-68 1045.09393627154 950.155856577876 1021.34385604155 2285.10800222532 2258.62218776467 2231.61453942898 down 981.0 923.0 962.0 2416.0 2725.0 2083.0

SYNE2 -1.22710024884849 1.27822137485906e-69 7.87384366913184e-68 1680.03377930706 1538.98483811909 1423.72360805792 3719.92126355636 3734.80791855692 3410.09557321288 down 1577.0 1495.0 1341.0 3933.0 4506.0 3183.0

MXRA7 1.12675482557703 1.58367473572434e-69 9.72009058810517e-68 2962.69748906337 3062.52835679218 3213.72957613075 1471.70035242657 1337.76741689988 1423.81935808983 up 2781.0 2975.0 3027.0 1556.0 1614.0 1329.0

SESN3 5.29087558789291 1.64383542728206e-69 1.00529141292989e-67 435.722140606587 333.532500033837 334.431720013606 9.45822848603194 8.28852178996211 10.7134639434901 up 409.0 324.0 315.0 10.0 10.0 10.0

SLC16A9 -4.28884726982841 1.87354323593605e-69 1.14164828837254e-67 26.6333826776642 17.5001620388124 10.6168800004319 363.195973863627 385.416263233238 319.261225516004 down 25.0 17.0 10.0 384.0 465.0 298.0

CTNND2 3.57895542034089 2.360244942856e-69 1.43306628430038e-67 323.861933360397 402.503726892686 329.12328001339 35.9412682469214 24.8655653698863 27.8550062530741 up 304.0 391.0 310.0 38.0 30.0 26.0

PNP -1.27826972575895 3.18353720696205e-69 1.92604001021204e-67 685.010602469524 767.948287114946 700.714080028508 1750.71809276451 1735.61646281806 1738.79519802844 down 643.0 746.0 660.0 1851.0 2094.0 1623.0

SH3PXD2A -1.75238846984541 3.84166399879867e-69 2.31593552098397e-67 248.22312655583 246.031689839775 225.077856009157 818.136764041763 816.419396311267 788.510946240868 down 233.0 239.0 212.0 865.0 985.0 736.0

FAM131A -3.00319880795722 6.56581116039523e-69 3.94414329989699e-67 54.332100662435 73.088912044452 42.4675200017278 488.990412727851 403.651011171154 473.53510630226 down 51.0 71.0 40.0 517.0 487.0 442.0

NLRC5 -1.35513611434081 9.741678180119e-69 5.83123775163307e-67 597.653107286785 661.917893585671 579.681648023584 1596.54896844219 1522.60145281604 1588.80670281957 down 561.0 643.0 546.0 1688.0 1837.0 1483.0

MMP24 1.25740515567293 5.2061231919697e-68 3.10534249549179e-66 1853.68343436543 2058.84259280146 1940.76566407896 826.649169679192 779.121048256438 845.292305141365 up 1740.0 2000.0 1828.0 874.0 940.0 789.0

DMKN 2.63397128852351 5.28330534313232e-68 3.14032254430391e-66 562.497042152268 643.388310250458 612.593976024923 102.148867649145 67.9658786776893 126.418874533183 up 528.0 625.0 577.0 108.0 82.0 118.0

F2R 1.13757396910618 3.70519041610351e-67 2.19461278492285e-65 2189.264056104 2230.75594930039 2299.61620809356 1012.03044800542 1060.10193693615 979.210604434991 up 2055.0 2167.0 2166.0 1070.0 1279.0 914.0

ADA 1.39504218175243 7.10665762403019e-67 4.18009653302331e-65 1310.36242774108 1461.77824088904 1335.60350405434 540.064846552424 508.086385724677 514.246269287523 up 1230.0 1420.0 1258.0 571.0 613.0 480.0

IL11 2.46987854993866 1.10386641291359e-66 6.47041419887758e-65 467.682199819784 479.710324122741 428.921952017451 90.7989934659067 72.9389917516665 85.7077115479204 up 439.0 466.0 404.0 96.0 88.0 80.0

MMP1 -6.88863414645824 2.00084776853648e-66 1.16877107582786e-64 7.45734714974598 2.05884259280146 5.30844000021597 554.252189281472 589.313899266306 602.096673624141 down 7.0 2.0 5.0 586.0 711.0 562.0

CDKN2AIP -1.48912258629721 2.38746788576377e-66 1.38981807508036e-64 380.324704637045 394.26835652148 434.230392017666 1109.45020141155 1166.19501584767 1116.34294291166 down 357.0 383.0 409.0 1173.0 1407.0 1042.0

LCN2 -3.50811118419844 2.55773781314647e-66 1.47877401210584e-64 46.874753512689 27.7943750028198 21.2337600008639 347.116985437372 360.550697863352 382.470662782595 down 44.0 27.0 20.0 367.0 435.0 357.0

FAM20C -1.70193444295694 6.78709003950643e-66 3.88423328612293e-64 248.22312655583 252.208217618179 253.743432010323 836.107398165224 774.147935182461 845.292305141365 down 233.0 245.0 239.0 884.0 934.0 789.0

COL27A1 1.57709483268048 1.71319075789678e-65 9.73874209354746e-64 1380.67455801011 1600.75011590314 1307.99961605322 506.015224002709 490.680489965757 439.252021683092 up 1296.0 1555.0 1232.0 535.0 592.0 410.0

MPP7 2.1256775315419 3.87070138698355e-65 2.18565604985004e-63 572.085059916227 525.004861164373 518.103744021079 127.686084561431 125.985531207424 115.705410589693 up 537.0 510.0 488.0 135.0 152.0 108.0

RARRES3 -2.14459635497132 4.09823100423818e-65 2.30644628610614e-63 119.317554395936 117.354027789683 142.266192005788 556.143834978678 541.240472884526 578.527052948463 down 112.0 114.0 134.0 588.0 653.0 540.0

LINC00346 1.8352735184294 1.17333080822697e-64 6.55980986513694e-63 682.87993185531 656.770787103667 690.097200028076 187.272924023432 203.068783854072 176.772155067586 up 641.0 638.0 650.0 198.0 245.0 165.0

LBH -2.23354800305649 1.8258536499037e-64 1.01743292201871e-62 139.55892523096 156.472037052911 122.094120004967 736.795999061888 573.565707865378 658.878032524638 down 131.0 152.0 115.0 779.0 692.0 615.0

MARK1 2.69023339975224 2.46553008707872e-64 1.36937966147913e-62 372.867357487299 450.886527823521 419.366760017062 54.8577252189853 65.4793221407006 72.8515548157324 up 350.0 438.0 395.0 58.0 79.0 68.0

DDAH2 -1.48191323423846 3.68235175865372e-64 2.03189051438417e-62 501.772929647194 549.710972277991 479.882976019524 1563.44516874108 1353.51560830081 1361.68126721759 down 471.0 534.0 452.0 1653.0 1633.0 1271.0

HCFC1R1 -1.27947391428414 3.99966865849051e-64 2.19981776216978e-62 902.339005119264 844.1254630486 851.473776034642 2265.24572240465 1937.02754231414 2106.26701129014 down 847.0 820.0 802.0 2395.0 2337.0 1966.0

IGFBP5 1.25611204836689 1.10523594365135e-63 6.03957964046901e-62 1566.04290144666 1708.83935202522 1624.38264006609 708.421313603793 651.477812691021 693.161117143806 up 1470.0 1660.0 1530.0 749.0 786.0 647.0

NCAPH2 -1.11424793771736 1.11887927164523e-63 6.0944742320483e-62 1322.08111611925 1436.04270847902 1423.72360805792 3157.15666863746 3061.779949212 2831.56852026442 down 1241.0 1395.0 1341.0 3338.0 3694.0 2643.0

DTNA 6.28519937889085 1.18699419298815e-63 6.4447697529549e-62 449.571499598972 370.591666704264 427.860264017407 7.56658278882556 1.65770435799242 7.49942476044304 up 422.0 360.0 403.0 8.0 2.0 7.0

ST6GAL1 -2.77721559943866 1.53423460916973e-63 8.30349337997928e-62 53.2667653553284 55.5887500056395 54.1460880022029 365.087619560833 375.470037085283 377.11393081085 down 50.0 54.0 51.0 386.0 453.0 352.0

ILDR2 5.37850816012389 2.04290470575702e-63 1.10212757055809e-61 332.384615817249 321.179444477028 325.938216013261 2.83746854580958 6.63081743196968 14.9988495208861 up 312.0 312.0 307.0 3.0 8.0 14.0

ARRDC3 -1.48803140498785 8.25274826477061e-63 4.43814462238775e-61 471.94354104821 387.062407446675 478.821288019481 1234.29881742717 1268.9726860432 1247.04720302224 down 443.0 376.0 451.0 1305.0 1531.0 1164.0

CDA -1.28159112046097 9.35345971263574e-63 5.01416479531802e-61 1084.51134263449 1274.42356494411 1260.22365605127 2997.31260722352 2695.42728609568 3111.18992918951 down 1018.0 1238.0 1187.0 3169.0 3252.0 2904.0

CDCP1 -2.16109821985236 1.48719948295684e-62 7.94736884583247e-61 125.709566238575 161.619143534915 129.52593600527 616.676497289283 565.277186075416 686.733038777712 down 118.0 157.0 122.0 652.0 682.0 641.0

C6orf132 1.51512084369855 3.98276879175974e-62 2.12163846957264e-60 918.319034725862 962.508912134685 897.126360036499 330.092174162515 309.990714944583 333.188728642541 up 862.0 935.0 845.0 349.0 374.0 311.0

C1orf106 -4.56533537519429 5.7281513694491e-62 3.03234013120212e-60 18.1107002208117 11.3236342604081 9.55519200038875 314.959008584864 289.269410469677 318.189879121655 down 17.0 11.0 9.0 333.0 349.0 297.0

ADGRE5 1.77713005054658 7.52950565173814e-62 3.97351482057458e-60 679.683925933991 716.47722229491 652.938120026565 194.839506812258 193.122557706117 211.055239686754 up 638.0 696.0 615.0 206.0 233.0 197.0

HNRNPUL2 -1.1196655144031 1.64108488998458e-61 8.6068043456157e-60 1277.33703322078 1136.48111122641 1210.32432004924 2545.2092855912 2572.75716360424 2758.71696544869 down 1199.0 1104.0 1140.0 2691.0 3104.0 2575.0

ADAMTS15 -1.99318135377003 1.76876584571346e-61 9.24780661308213e-60 145.9509370736 195.590046316139 176.24020800717 744.362581850714 643.189290901059 676.019574834222 down 137.0 190.0 166.0 787.0 776.0 631.0

ATP2B4 1.03944847675372 6.17045598777497e-61 3.21623152101255e-59 2456.66321818775 2530.317546553 2471.60966410056 1224.84058894114 1249.08023374729 1151.69737392518 up 2306.0 2458.0 2328.0 1295.0 1507.0 1075.0

C1orf116 -2.59474455171817 7.22461802802993e-61 3.75414200597629e-59 59.6587771979678 61.7652777840439 80.6882880032828 396.299773564738 426.858872183048 396.398165909132 down 56.0 60.0 76.0 419.0 515.0 370.0

SC5D -1.02091286465751 7.94294126701141e-61 4.11478364107563e-59 2679.31829737302 2328.55097245846 2599.01222410574 4991.10717207906 5290.56345853281 5151.03346403002 down 2515.0 2262.0 2448.0 5277.0 6383.0 4808.0

CRTAC1 3.64849632189497 8.36222488191627e-61 4.3187832164531e-59 274.856509233495 301.620439845415 286.655760011662 28.3746854580958 15.748191400928 25.7123134643761 up 258.0 293.0 270.0 30.0 19.0 24.0

SLC12A3 -4.35082642002795 2.0005958550078e-60 1.03009403598274e-58 19.1760355279182 9.26479166760659 14.8636320006047 302.663311553022 287.611706111685 290.33487286858 down 18.0 9.0 14.0 320.0 347.0 271.0

TMEM246 2.59876308320127 5.38613911782447e-60 2.76488474714989e-58 397.37006955075 380.885879668271 401.318064016327 79.4491192826683 62.992765603712 51.4246269287523 up 373.0 370.0 378.0 84.0 76.0 48.0

LIMA1 -1.03084137320732 9.03880639854723e-60 4.61196928889729e-58 1430.74531744412 1286.77662050092 1373.82427205589 2735.31967816044 2835.50330434604 2786.57197170176 down 1343.0 1250.0 1294.0 2892.0 3421.0 2601.0

FAM171A1 1.38792063970908 2.47505281300978e-59 1.2515640194742e-57 1359.36785186798 1507.07277793067 1265.53209605149 544.79396079544 523.834577125605 509.960883710126 up 1276.0 1464.0 1192.0 576.0 632.0 476.0

RASSF2 1.65273810765502 2.92673461767012e-59 1.47556203640868e-57 784.086786030434 805.007453785373 765.477048031143 222.268369421751 266.89040163678 259.265827432459 up 736.0 782.0 721.0 235.0 322.0 242.0

SCN9A 3.37795851885528 4.99058927925198e-59 2.50120066244167e-57 313.208580289331 305.738125031017 289.840824011792 26.4830397608894 39.7849045918181 19.2842350982821 up 294.0 297.0 273.0 28.0 48.0 18.0

YARS2 -1.07867368851101 6.51585668132853e-59 3.25600625904735e-57 921.515040647182 920.302638982255 970.38283203948 1960.69076515442 1955.26229025206 2024.84468531962 down 865.0 894.0 914.0 2073.0 2359.0 1890.0

C8orf4 -7.57014413790211 9.03284521357573e-59 4.50048229170509e-57 6.39201184263941 2.05884259280146 2.12337600008639 648.834474141791 722.759100084696 625.666294299819 down 6.0 2.0 2.0 686.0 872.0 584.0

FOSL2 1.10115648511529 9.93541475067755e-59 4.93565765033659e-57 14665.405837629 17832.6651175499 15663.0830646372 7857.89622619534 7064.3071215847 7530.49380587916 up 13766.0 17323.0 14753.0 8308.0 8523.0 7029.0

ZSWIM5 4.64474293778804 1.30229442436896e-58 6.45054606690357e-57 254.61513839847 266.62011576779 276.038880011231 12.2956970318415 10.7750783269507 8.57077115479204 up 239.0 259.0 260.0 13.0 13.0 8.0

TPCN1 -1.19283125418115 1.46754070352114e-58 7.24785408677786e-57 759.584073966983 875.008101940622 828.116640033692 1850.97531471645 1825.13249814966 1957.34986247563 down 713.0 850.0 780.0 1957.0 2202.0 1827.0

MAL -6.15430059691609 1.7600448322085e-58 8.66719751674766e-57 10.6533530710657 3.0882638892022 3.18506400012958 386.841545078707 397.020193739185 415.682401007414 down 10.0 3.0 3.0 409.0 479.0 388.0

TSPAN18 1.50539932777198 1.85545155892504e-58 9.110536060345e-57 1268.81435076392 1517.36699089468 1505.47358406125 487.098767030645 466.643776774867 561.385510638879 up 1191.0 1474.0 1418.0 515.0 563.0 524.0

CYP27C1 -2.76833981047665 1.97482837208812e-58 9.66866838820023e-57 46.874753512689 62.7946990804447 53.0844000021597 391.570659321722 333.198575956477 386.756048359991 down 44.0 61.0 50.0 414.0 402.0 361.0

TCF4 2.61075957656126 3.38565732874585e-58 1.65282522043097e-56 372.867357487299 382.944722261072 399.194688016241 67.1534222508268 73.7678439306627 46.0678949570072 up 350.0 372.0 376.0 71.0 89.0 43.0

OGFOD1 -1.06523184723508 3.7918518133921e-57 1.83002762731801e-55 958.801776395912 904.861319536244 933.223752037968 1911.50797702706 1991.73178612789 1947.70774492649 down 900.0 879.0 879.0 2021.0 2403.0 1818.0

NT5DC2 1.63901059234404 4.28907868823718e-57 2.06411911871414e-55 1161.21548474616 1502.95509274507 1158.30160804713 417.107876234009 399.506750276173 411.397015430018 up 1090.0 1460.0 1091.0 441.0 482.0 384.0

HES2 -2.49483704483807 5.9998155009092e-57 2.87923157465728e-55 73.5081361903532 74.1183333408527 58.3928400023757 398.191419261945 395.362489381192 367.471813261709 down 69.0 72.0 55.0 421.0 477.0 343.0

PCDH9 -3.19126105902296 7.74676157981011e-57 3.7070661345193e-55 33.0253945203036 32.9414814848234 32.912328001339 278.071917489339 322.423497629526 302.119683206419 down 31.0 32.0 31.0 294.0 389.0 282.0

PDE11A -2.52302380268047 1.03258726348635e-56 4.92733189956586e-55 96.9455129466977 59.7064351912425 87.0584160035419 514.527629640138 468.301481132859 414.611054613065 down 91.0 58.0 82.0 544.0 565.0 387.0

SRGAP1 -1.05044271973964 1.25363042247214e-56 5.96530880805564e-55 1176.13017904565 1174.56969919324 1203.95419204898 2493.18902891802 2561.15323309829 2303.39474785036 down 1104.0 1141.0 1134.0 2636.0 3090.0 2150.0

ECI1 -1.18167878862529 1.25792528440196e-56 5.96897880049556e-55 1145.23545513956 1317.65925939294 1193.33731204855 2948.12981909616 2545.40504169736 2804.7848604057 down 1075.0 1280.0 1124.0 3117.0 3071.0 2618.0

LGALS3 -1.09692605417084 1.78312124445496e-56 8.41394815628609e-55 858.660257527894 800.88976859977 849.350400034555 1806.5216408321 1748.878097682 1811.64675284417 down 806.0 778.0 800.0 1910.0 2110.0 1691.0

ATRNL1 3.04121059632964 2.3462207127929e-56 1.10402719096421e-54 315.339250903544 330.444236144635 266.483688010842 36.8870910955246 35.6406436968371 38.5684701965642 up 296.0 321.0 251.0 39.0 43.0 36.0

VWA5A 3.03998170762701 2.37256130711335e-56 1.11332932250693e-54 305.751233139585 281.0320139174 353.542104014384 44.4536738843501 37.2983480548295 32.1403918304702 up 287.0 273.0 333.0 47.0 45.0 30.0

TBX1 -1.65987544654218 6.46540348374743e-56 3.02552306670391e-54 290.836538840093 378.827037075469 331.246656013477 1167.14539517634 969.757049425566 1028.49253857505 down 273.0 368.0 312.0 1234.0 1170.0 960.0

ITGB8 -2.7910722091858 7.24109990397448e-56 3.37917995518809e-54 51.1360947411153 50.4416435236359 63.7012800025917 318.742299979277 419.399202572083 404.968937063924 down 48.0 49.0 60.0 337.0 506.0 378.0

HERPUD1 -1.063163620639 8.10518540794966e-56 3.77202859369965e-54 957.736441088805 904.861319536244 966.136080039307 1920.02038266448 1965.20851640002 2025.91603171397 down 899.0 879.0 910.0 2030.0 2371.0 1891.0

CA2 -3.48331441386176 1.04019051508858e-55 4.82762392482208e-54 20.2413708350248 25.7355324100183 28.6655760011662 273.342803246323 266.89040163678 295.691604840325 down 19.0 25.0 27.0 289.0 322.0 276.0

DMD 3.17740622480436 1.13277482191139e-55 5.22866634419047e-54 271.660503312175 284.120277806602 266.483688010842 33.1037997011118 30.6675306228598 26.7836598587251 up 255.0 276.0 251.0 35.0 37.0 25.0

ESPN -2.89151849255591 2.19136269819264e-55 1.00600769938708e-53 36.2214004416233 59.7064351912425 44.5908960018142 332.929642708324 326.567758524507 386.756048359991 down 34.0 58.0 42.0 352.0 394.0 361.0

AMPD2 1.33326302821107 2.43951579594885e-55 1.11690263738847e-53 1127.12475491875 1240.45266216288 1111.58733604522 482.369652787629 446.751324478957 452.10817841528 up 1058.0 1205.0 1047.0 510.0 539.0 422.0

NT5C3B -1.07986347976467 2.84941382698649e-55 1.30105310590704e-53 886.358975512665 847.213726937803 925.791936037665 1902.04974854102 1890.61182029036 1826.64560236505 down 832.0 823.0 872.0 2011.0 2281.0 1705.0

TMEM47 -1.53398735417974 3.03812563470338e-55 1.38349054440525e-53 296.163215375626 269.708379656992 259.051872010539 794.491192826683 808.130874521305 785.296907057821 down 278.0 262.0 244.0 840.0 975.0 733.0

IL6 -4.37720971746595 3.65666809089157e-55 1.66069591044781e-53 7.45734714974598 9.26479166760659 23.3571360009503 261.047106214482 276.836627784734 293.548912051627 down 7.0 9.0 22.0 276.0 334.0 274.0

PCDHA1 1.92741291588496 5.48573718820395e-55 2.48471625583356e-53 580.60774237308 509.563541718362 553.139448022504 128.631907410034 154.995357472291 147.845802420163 up 545.0 495.0 521.0 136.0 187.0 138.0

IRS2 1.31824971272207 1.11438527616605e-54 5.02066132400342e-53 3173.63387987047 4007.53710688805 3240.27177613183 1484.94187230702 1297.15366012907 1399.1783910198 up 2979.0 3893.0 3052.0 1570.0 1565.0 1306.0

RARRES2 1.31243415293023 1.22536509100606e-54 5.50601714632432e-53 1290.12105690605 1207.51118067806 1338.78856805447 554.252189281472 513.059498798654 475.677799090958 up 1211.0 1173.0 1261.0 586.0 619.0 444.0

NAT8L -1.45516703484602 1.46829339727303e-54 6.56276784955278e-53 346.233974809635 436.47462967391 371.590800015118 1048.91753910094 1041.86718899824 1076.70312632075 down 325.0 424.0 350.0 1109.0 1257.0 1005.0

SCARF2 1.42465087119369 1.50960588767951e-54 6.72966414139236e-53 1038.7019244289 1248.68803253409 1158.30160804713 448.320030237914 407.795272066136 428.538557739602 up 975.0 1213.0 1091.0 474.0 492.0 400.0

SYTL2 1.31044256340785 1.784279798717e-54 7.93325453812755e-53 1050.42061280708 1052.06856492155 1081.86007204401 423.728636174231 450.895585373939 407.111629852622 up 986.0 1022.0 1019.0 448.0 544.0 380.0

ALDH1A1 6.00440416220539 3.17822682075545e-54 1.40940215559155e-52 319.600592131971 299.561597252613 342.925224013952 2.83746854580958 7.45966961096589 4.28538557739602 up 300.0 291.0 323.0 3.0 9.0 4.0

HOGA1 1.70657120041977 4.1896761066997e-54 1.85308389680138e-52 734.016026596426 630.005833397248 673.110192027385 207.1352038441 195.609114243106 222.840050024593 up 689.0 612.0 634.0 219.0 236.0 208.0

GNG4 4.16132067234865 6.06506606487745e-54 2.67557862341208e-52 223.720414492379 275.884907435396 241.003176009805 17.9706341234607 9.94622614795453 13.9275031265371 up 210.0 268.0 227.0 19.0 12.0 13.0

KLHL5 -1.16499604409784 7.90263661876058e-54 3.46815192543534e-52 743.604044360385 660.88847228927 664.61668802704 1527.50390049416 1573.16143573481 1537.38207589082 down 698.0 642.0 626.0 1615.0 1898.0 1435.0

LINC01111 -3.16864050387137 4.43453893366501e-53 1.9361105550589e-51 41.5480769771562 29.8532175956212 27.6038880011231 267.667866154704 327.396610703503 293.548912051627 down 39.0 29.0 26.0 283.0 395.0 274.0

LRRN4 1.41491884856312 4.82942098098988e-53 2.10309489506346e-51 1351.91050471824 1525.60236126589 1386.56452805641 566.547886313313 458.355254984904 578.527052948463 up 1269.0 1482.0 1306.0 599.0 553.0 540.0

ZNF462 2.8650650098041 1.1953566513345e-52 5.17885976307072e-51 278.052515154814 310.885231513021 273.915504011144 38.778736792731 37.2983480548295 42.8538557739602 up 261.0 302.0 258.0 41.0 45.0 40.0

PLCXD1 -1.98037098898583 1.73086320644382e-52 7.46076913922602e-51 113.990877860403 127.648240753691 127.402560005183 515.473452488741 461.670663700889 479.963184668354 down 107.0 124.0 120.0 545.0 557.0 448.0

PNMA2 1.48076048786915 2.70124176991345e-52 1.15553120157409e-50 744.669379667491 764.860023225744 746.366664030366 256.317991971466 278.494332142727 273.193330558996 up 699.0 743.0 703.0 271.0 336.0 255.0

TEAD2 -1.49977553856285 2.87280306447863e-52 1.22582579124101e-50 360.08333380202 410.739097263892 405.5648160165 1159.57881238752 977.216719036532 1194.55122969914 down 338.0 399.0 382.0 1226.0 1179.0 1115.0

CPS1 -2.34348237650206 3.36763118476984e-52 1.42976622230579e-50 79.9001480329926 63.8241203768454 76.44153600311 366.979265258039 375.470037085283 373.899891627803 down 75.0 62.0 72.0 388.0 453.0 349.0

LURAP1L -3.00419907162126 5.11746445643605e-52 2.16724619730067e-50 34.0907298274102 33.9709027812242 36.0973920014686 282.801031732355 274.350071247746 278.550062530741 down 32.0 33.0 34.0 299.0 331.0 260.0

IRF2BPL -1.16162848579284 5.1423081585885e-52 2.17233666350347e-50 1381.73989331722 1690.30976869 1515.02877606164 3617.77239590722 3160.41335851255 3488.30386000036 down 1297.0 1642.0 1427.0 3825.0 3813.0 3256.0

ANXA8L1 1.13604176438957 5.56661824048901e-52 2.34573415407671e-50 1376.41321678169 1410.307176069 1337.72688005442 621.405611532299 610.864055920207 646.02187579245 up 1292.0 1370.0 1260.0 657.0 737.0 603.0

CXorf57 5.61086542424148 8.09914887532704e-52 3.40445612774293e-50 265.268491469536 289.267384288606 271.792128011058 2.83746854580958 5.80196525297347 8.57077115479204 up 249.0 281.0 256.0 3.0 7.0 8.0

TMOD1 -3.41699808983292 1.18197385732452e-51 4.95609830274191e-50 25.5680473705576 17.5001620388124 31.8506400012958 249.697232031243 265.232697278787 283.906794502486 down 24.0 17.0 30.0 264.0 320.0 265.0

KIAA1324L 2.40860190178149 3.19031553232183e-51 1.33113165314118e-49 346.233974809635 342.797291701444 367.344048014945 73.7741821910492 54.7042438137499 71.7802084213833 up 325.0 333.0 346.0 78.0 66.0 67.0

HAS3 -1.20318175159507 5.09157111386606e-51 2.11919446360912e-49 497.511588418768 520.887175978771 520.227120021165 1140.66235541545 1204.32221608149 1197.76526888219 down 467.0 506.0 490.0 1206.0 1453.0 1118.0

PLAUR 1.34068512116906 6.06469631080268e-51 2.51803812512249e-49 903.40434042637 960.450069541883 952.334136038745 379.274962289881 383.758558875245 347.116231769078 up 848.0 933.0 897.0 401.0 463.0 324.0

PRKX -1.75941480728922 9.49163830242918e-51 3.93125557073717e-49 224.785749799486 183.23699075933 169.870080006911 619.513965835092 686.289604208862 649.235914975497 down 211.0 178.0 160.0 655.0 828.0 606.0

LINC00839 1.49610472534375 1.18617390523144e-50 4.88899901572279e-49 742.538709053278 765.889444522145 690.097200028076 261.047106214482 261.088436383806 257.123134643761 up 697.0 744.0 650.0 276.0 315.0 240.0

GNAL 2.61615532618032 1.49654904507358e-50 6.1532866076569e-49 294.032544761413 325.297129662631 385.39274401568 57.6951937647949 56.3619481717423 49.2819341400542 up 276.0 316.0 363.0 61.0 68.0 46.0

ASMTL -1.7086738355607 1.89559736905737e-50 7.77516209003191e-49 175.780325672584 203.825416687345 238.879800009719 691.396502328935 663.910595375965 666.377457285081 down 165.0 198.0 225.0 731.0 801.0 622.0

ADAMTSL1 3.18565434256724 2.12517692742553e-50 8.69577225859623e-49 254.61513839847 245.002268543374 245.249928009978 32.1579768525086 22.3790088328977 27.8550062530741 up 239.0 238.0 231.0 34.0 27.0 26.0

LIPA 1.36954977398202 2.51621295430026e-50 1.02709993845413e-48 1313.5584336624 1117.9515278912 1232.61976805015 425.620281871437 508.086385724677 483.177223851401 up 1233.0 1086.0 1161.0 450.0 613.0 451.0

NIPAL3 1.02612872909249 2.61102141714699e-50 1.06323804823245e-48 1837.70340475883 1903.39997704495 1967.30786408004 948.660317149004 892.673796778919 964.211754914105 up 1725.0 1849.0 1853.0 1003.0 1077.0 900.0

STMN3 1.59105270716332 4.96201433851243e-50 2.01574395430217e-48 685.010602469524 807.066296378174 758.045232030841 273.342803246323 237.880575371912 235.696206756781 up 643.0 784.0 714.0 289.0 287.0 220.0

RAB9A -1.2320883756736 5.30927444314469e-50 2.15165332695864e-48 532.667653553284 475.592638937138 526.597248021424 1201.19501772606 1156.24878969971 1249.18989581094 down 500.0 462.0 496.0 1270.0 1395.0 1166.0

IPO13 1.02824619370203 6.3773090017631e-50 2.57832015488942e-48 1978.3276652969 2215.31462985438 2043.74940008315 1042.29677916072 1016.17277144935 999.566185927622 up 1857.0 2152.0 1925.0 1102.0 1226.0 933.0

ALS2CL -1.73178768311849 1.54232269785378e-49 6.16201568434978e-48 176.84566097969 161.619143534915 165.623328006738 586.410166133981 532.123098915567 556.028778667134 down 166.0 157.0 156.0 620.0 642.0 519.0

PSG4 1.36706059704531 1.58481014114549e-49 6.31686677435404e-48 987.565829687789 906.920162129045 1005.4185360409 343.33369404296 382.929706696249 398.54085869783 up 927.0 881.0 947.0 363.0 462.0 372.0

LIMK2 -1.18129529373866 1.66045495035455e-49 6.60284198568219e-48 610.437130972064 545.593287092388 604.100472024578 1285.37325125174 1320.36152114096 1386.32223428761 down 573.0 530.0 569.0 1359.0 1593.0 1294.0

ZFHX4 -1.81759436288364 2.26515109429689e-49 8.98633712819423e-48 154.473619530452 143.089560199702 146.512944005961 492.773704122264 557.81751646445 513.174922893174 down 145.0 139.0 138.0 521.0 673.0 479.0

BTBD11 1.71387320471833 2.2784413643472e-49 9.01794315701904e-48 568.889053994908 565.152291724002 599.853720024405 175.923049840194 193.951409885113 156.416573574955 up 534.0 549.0 565.0 186.0 234.0 146.0

SESN2 1.37620993166594 4.31414484382823e-49 1.69957241056861e-47 824.569527700484 931.626273242663 852.535464034685 338.604579799944 332.36972377748 334.26007503689 up 774.0 905.0 803.0 358.0 401.0 312.0

CYB5A 1.80590578992975 5.15814772433635e-49 2.02735550928672e-47 598.718442593891 566.181713020403 556.324512022634 174.977226991591 136.760609534375 183.20023343368 up 562.0 550.0 524.0 185.0 165.0 171.0

ITGA6 -1.00956927001366 7.9394973082627e-49 3.11331213893449e-47 1477.62007095681 1280.60009272251 1338.78856805447 2647.35815324034 2870.31509586388 2727.64792001257 down 1387.0 1244.0 1261.0 2799.0 3463.0 2546.0

SH3RF2 -1.98630313143523 8.33017536215276e-49 3.25896467978909e-47 133.166913388321 115.295185196882 98.7369840040171 466.290664361375 450.895585373939 458.536256781374 down 125.0 112.0 93.0 493.0 544.0 428.0

LLGL2 -1.43042906023069 8.40360278176026e-49 3.28011592449352e-47 385.651381172578 444.710000045116 451.217400018358 1291.04818834336 1077.50783269507 1086.34524386989 down 362.0 432.0 425.0 1365.0 1300.0 1014.0

CFAP97 -1.15744667210161 1.34412463490704e-48 5.23436122191385e-47 596.587771979678 558.975763945598 551.016072022418 1220.11147469812 1305.44218191903 1280.25894124706 down 560.0 543.0 519.0 1290.0 1575.0 1195.0

MEX3B 1.68330182609741 1.57210713181077e-48 6.10814101212715e-47 541.190336010137 625.888148211645 611.53228802488 190.110392569242 174.058957589204 190.699658194123 up 508.0 608.0 576.0 201.0 210.0 178.0

RSPRY1 -1.25876123975133 1.66251122614791e-48 6.44460873477016e-47 860.790928142107 696.918217663296 844.041960034339 1841.51708623042 2077.1035605645 1823.43156318201 down 808.0 677.0 795.0 1947.0 2506.0 1702.0

GLB1L2 -6.91844351316389 2.25749379037168e-48 8.73103762760189e-47 6.39201184263941 4.11768518560293 0 400.083064959151 408.624124245132 464.964335147468 down 6.0 4.0 0.0 423.0 493.0 434.0

FLNC 6.68126491336117 2.95253805661135e-48 1.13931650749422e-46 336.645957045676 400.444884299885 321.691464013088 0 4.97311307397726 5.35673197174503 up 316.0 389.0 303.0 0.0 6.0 5.0

GABRQ 4.62225415768578 3.11776980623739e-48 1.19761951287214e-46 224.785749799486 212.060787058551 211.275912008596 11.3498741832383 6.63081743196968 8.57077115479204 up 211.0 206.0 199.0 12.0 8.0 8.0

SPTBN2 -1.25748502035988 5.8245703139647e-48 2.22727361441449e-46 617.89447812181 653.682523214465 543.584256022115 1563.44516874108 1378.3811736707 1399.1783910198 down 580.0 635.0 512.0 1653.0 1663.0 1306.0

KCNMA1 1.44803430013363 8.45085227338306e-48 3.22426661061057e-46 964.128452931445 984.1267593591 1033.02242404203 415.216230536802 365.523810937329 309.619107966862 up 905.0 956.0 973.0 439.0 441.0 289.0

PRPS2 -1.24130749578183 1.89022216453306e-47 7.16339227453916e-46 457.028846748718 456.033634305524 493.684920020085 1037.5676649177 1148.78912008875 1138.84121719299 down 429.0 443.0 465.0 1097.0 1386.0 1063.0

GALNT16 -2.06110332493852 2.27322510091461e-47 8.59563241283338e-46 92.6841717182715 90.5890740832644 102.98373600419 395.353950716135 382.100854517253 417.825093796112 down 87.0 88.0 97.0 418.0 461.0 390.0

BMF 1.09739490454969 2.29674489329283e-47 8.66522460854799e-46 1290.12105690605 1355.74784735976 1333.48012805425 628.972194321124 636.55847346909 592.454556075 up 1211.0 1317.0 1256.0 665.0 768.0 553.0

CNRIP1 1.78537456660206 3.71255727217302e-47 1.39447273149913e-45 542.255671317243 525.004861164373 502.178424020431 166.464821354162 130.958644281401 159.630612758002 up 509.0 510.0 473.0 176.0 158.0 149.0

RAB36 -2.00327052147885 6.84873876860243e-47 2.56109568962749e-45 111.86020724619 101.912708343672 100.860360004103 417.107876234009 449.237881015946 393.184126726085 down 105.0 99.0 95.0 441.0 542.0 367.0

HSPB8 2.23853153733166 9.28900091777864e-47 3.46598404288921e-45 356.8873278807 457.063055601925 428.921952017451 104.986336194955 75.4255482886552 83.5650187592224 up 335.0 444.0 404.0 111.0 91.0 78.0

BNC1 -5.38443781157899 1.6928958304623e-46 6.27519811116659e-45 1.06533530710657 9.26479166760659 8.49350400034555 290.367614521181 227.934349223958 273.193330558996 down 1.0 9.0 8.0 307.0 275.0 255.0

AFF3 1.76169940072161 1.94201431383247e-46 7.18290883762492e-45 610.437130972064 590.88782413402 608.34722402475 205.243558146893 185.662888095151 140.34637765972 up 573.0 574.0 573.0 217.0 224.0 131.0

BIRC3 -2.33551610069577 2.22110625242972e-46 8.19728538478419e-45 72.4428008832467 65.8829629696469 63.7012800025917 307.392425796038 347.289062999412 365.329120473011 down 68.0 64.0 60.0 325.0 419.0 341.0

EHF -7.0975264483822 2.65011800385719e-46 9.75934760550886e-45 0 6.17652777840439 3.18506400012958 418.053699082612 467.472628953863 409.25432264132 down 0.0 6.0 3.0 442.0 564.0 382.0

FAT3 1.68422188611042 2.79003565950287e-46 1.02523219245073e-44 647.723866720794 650.594259325263 601.977096024491 163.627352808353 222.132383970984 204.62716132066 up 608.0 632.0 567.0 173.0 268.0 191.0

FBXO17 -1.36515441752142 3.91299440546917e-46 1.4347646153387e-44 367.540680951766 400.444884299885 332.30834401352 1005.4096880652 897.646909852896 933.142709477984 down 345.0 389.0 313.0 1063.0 1083.0 871.0

TSHZ3 4.85111427539717 4.0042902301304e-46 1.46506860687708e-44 657.311884484753 640.300046361255 575.434896023411 34.9954453983182 14.9193392219318 14.9988495208861 up 617.0 622.0 542.0 37.0 18.0 14.0

TMCO4 1.56218017013742 4.49423114955447e-46 1.64078180330717e-44 576.346401144654 598.093773208825 592.421904024102 209.026849541306 203.897636033068 184.271579828029 up 541.0 581.0 558.0 221.0 246.0 172.0

FKBP5 1.0727547918319 5.38131938704643e-46 1.96042043906595e-44 1484.01208279945 1361.92437513817 1439.64892805857 654.509411233411 699.551239072802 682.447653200316 up 1393.0 1323.0 1356.0 692.0 844.0 637.0

TMEM98 3.07708760253318 6.7162257000559e-46 2.43624975072692e-44 213.067061421314 299.561597252613 306.827832012483 29.320508306699 38.1272002338257 28.9263526474231 up 200.0 291.0 289.0 31.0 46.0 27.0

KCNK1 -3.14904255776978 8.41490001464517e-46 3.03294481378913e-44 94.8148423324846 165.736828720518 149.69800800609 1219.16565184952 1239.96285977833 1181.69507296695 down 89.0 161.0 141.0 1289.0 1496.0 1103.0

MYPN -2.12183094286254 8.54176230268846e-46 3.07213276873763e-44 87.3574951827386 83.3831250084593 77.5032240031532 364.14179671223 351.433323894393 365.329120473011 down 82.0 81.0 73.0 385.0 424.0 341.0

TBX3 2.8866856659136 8.75689275789618e-46 3.14283396861782e-44 222.655079185273 262.502430582187 271.792128011058 30.2663311553022 34.8117915178408 37.4971238022152 up 209.0 255.0 256.0 32.0 42.0 35.0

TBC1D12 1.46898715858205 1.12842370617062e-45 4.0328053971583e-44 813.916174629418 716.47722229491 755.921856030754 267.667866154704 255.286471130833 305.333722389467 up 764.0 696.0 712.0 283.0 308.0 285.0

MST1R -1.86571772625097 1.89710685366726e-45 6.75146850863938e-44 127.840236852788 173.972199091724 170.931768006954 636.53877710995 520.51916840962 567.813589004973 down 120.0 169.0 161.0 673.0 628.0 530.0

SYT17 -1.49071371515247 2.07933000500151e-45 7.38445498631563e-44 231.177761642125 223.384421318959 227.201232009244 657.34687977922 645.675847438048 611.738791173282 down 217.0 217.0 214.0 695.0 779.0 571.0

NID1 1.1470464082592 3.33628818560316e-45 1.17988980927176e-43 1132.45143145428 1213.68770845646 1167.85680004751 555.198012130075 500.626716113711 532.459157991456 up 1063.0 1179.0 1100.0 587.0 604.0 497.0

CAPRIN2 -1.02122040743451 4.63875805268472e-45 1.63369150545695e-43 1118.6020724619 957.361805652681 1044.7009920425 2145.12622063204 2167.44844807509 2017.34526055918 down 1050.0 930.0 984.0 2268.0 2615.0 1883.0

EYA2 6.44632594040225 6.2168490044019e-45 2.18492577042672e-43 288.70586822588 288.237962992205 323.814840013174 6.62075994022236 1.65770435799242 2.14269278869801 up 271.0 280.0 305.0 7.0 2.0 2.0

SLCO4A1 -2.32598110657556 9.4510948242098e-45 3.30788318847343e-43 63.9201184263941 63.8241203768454 65.8246560026781 345.225339740166 294.242523543655 332.117382248192 down 60.0 62.0 62.0 365.0 355.0 310.0

SOD3 -6.68381053009219 1.00935636001993e-44 3.51820920550158e-43 6.39201184263941 0 4.24675200017278 340.49622549715 339.00054120945 403.897590669575 down 6.0 0.0 4.0 360.0 409.0 377.0

FRMD4A 1.29880000938393 1.19927356999647e-44 4.16305210568446e-43 786.217456644648 796.772083414167 786.710808032007 315.904831433467 314.134975839564 334.26007503689 up 738.0 774.0 741.0 334.0 379.0 312.0

SLCO2B1 -1.86398258524352 1.44582848346588e-44 5.00865736399017e-43 105.46819540355 138.971875014099 131.649312005356 446.428384540708 456.697550626912 467.107027936166 down 99.0 135.0 124.0 472.0 551.0 436.0

MSRB2 1.34836599388585 1.7118981040961e-44 5.91827630273223e-43 813.916174629418 747.359861186932 827.054952033648 312.121540039054 298.386784438636 328.903343065145 up 764.0 726.0 779.0 330.0 360.0 307.0

MAF 7.82551095543118 2.1964457914214e-44 7.56255928997532e-43 483.662229426382 573.387662095208 436.353768017753 1.89164569720639 0 5.35673197174503 up 454.0 557.0 411.0 2.0 0.0 5.0

ZNF22 1.3395606342543 2.21431251962891e-44 7.60861137576343e-43 862.921598756321 794.713240821365 772.908864031445 306.446602947435 334.856280314469 318.189879121655 up 810.0 772.0 728.0 324.0 404.0 297.0

ITGB4 -1.15700367141156 2.42568301406888e-44 8.30122631481351e-43 2110.42924337811 2705.31916694112 2155.22664008768 5530.22619578288 4816.46001214698 5202.45809095877 down 1981.0 2628.0 2030.0 5847.0 5811.0 4856.0

TGFB2 1.60682700170416 3.42043061389934e-44 1.16583691346992e-42 572.085059916227 527.063703757175 591.360216024059 174.031404142988 180.689775021174 201.413122137613 up 537.0 512.0 557.0 184.0 218.0 188.0

ADI1 -1.0042798130341 3.88238853325637e-44 1.31798921349425e-42 801.13215094414 740.153912112126 749.551728030495 1531.28719188857 1554.92668779689 1507.38437684905 down 752.0 719.0 706.0 1619.0 1876.0 1407.0

ANK2 2.44630602454815 8.26395653296877e-44 2.77760761247006e-42 270.595168005068 276.914328731797 274.977192011187 45.3994967329533 53.8753916347537 51.4246269287523 up 254.0 269.0 259.0 48.0 65.0 48.0

PSG9 1.78895261445864 9.93908828244538e-44 3.3340228812797e-42 435.722140606587 435.44520837751 421.490136017148 118.227856075399 122.670122491439 133.918299293626 up 409.0 423.0 397.0 125.0 148.0 125.0

TTC9 -1.28918808678769 1.34213101126014e-43 4.47553136432023e-42 367.540680951766 364.415138925859 400.256376016284 977.0350026071 860.348561798066 931.000016689286 down 345.0 354.0 377.0 1033.0 1038.0 869.0

GUCY1A2 2.12616603172441 2.20027877648856e-43 7.32273526006213e-42 355.821992573594 372.650509297065 325.938216013261 69.0450679480332 82.0563657206248 91.0644435196654 up 334.0 362.0 307.0 73.0 99.0 85.0

LOC101928841 1.94362461110006 2.5197037682507e-43 8.36936898709156e-42 362.214004416233 396.327199114282 387.516120015766 106.877981892161 99.4622614795453 91.0644435196654 up 340.0 385.0 365.0 113.0 120.0 85.0

DIRAS2 6.14363598932335 2.95768742896972e-43 9.80493640836538e-42 230.112426335019 268.678958360591 299.396016012181 4.72911424301597 1.65770435799242 5.35673197174503 up 216.0 261.0 282.0 5.0 2.0 5.0

SPHK1 -1.30158890798516 3.35116695258857e-43 1.10876500345411e-41 400.56607547207 474.563217640738 411.934944016759 1029.05525928028 996.280319153445 1151.69737392518 down 376.0 461.0 388.0 1088.0 1202.0 1075.0

TYSND1 1.26881880549418 4.11214846270473e-43 1.35789073992628e-41 873.574951827386 913.09668990745 833.425080033908 339.550402648547 367.181515295321 381.399316388246 up 820.0 887.0 785.0 359.0 443.0 356.0

MALL -4.26304087821896 4.34155045034892e-43 1.43085339744963e-41 3.19600592131971 16.4707407424117 12.7402560005183 213.755963784322 208.041896928049 203.555814926311 down 3.0 16.0 12.0 226.0 251.0 190.0

EPB41 2.13036653037226 5.17755074373748e-43 1.70306232230899e-41 345.168639502528 336.620763923039 367.344048014945 78.5032964340651 92.8314440475756 66.4234764496383 up 324.0 327.0 346.0 83.0 112.0 62.0

C14orf159 -2.08234558464456 6.66054912444774e-43 2.18239269184032e-41 74.5734714974598 85.4419676012608 106.168800004319 388.733190775913 377.956593622272 359.972388501266 down 70.0 83.0 100.0 411.0 456.0 336.0

THRB 1.22062575947113 8.33390473539282e-43 2.72541208914198e-41 1596.93762535275 1354.71842606336 1588.28524806462 719.771187787031 619.152577710169 608.524751990235 up 1499.0 1316.0 1496.0 761.0 747.0 568.0

ADAMTS14 -2.78110955979187 1.98623034378793e-42 6.42113397400142e-41 37.2867357487299 35.0003240776249 36.0973920014686 259.155460517275 262.746140741799 221.768703630244 down 35.0 34.0 34.0 274.0 317.0 207.0

DRAM1 -1.17462115698466 2.33972933470521e-42 7.54952665331549e-41 404.827416700496 442.651157452315 446.970648018185 988.384876790338 974.730162499544 958.85502294236 down 380.0 430.0 421.0 1045.0 1176.0 895.0

WSCD1 2.92998093431709 3.11842709850044e-42 1.00239383393923e-40 226.916420413699 215.149050947753 208.090848008466 29.320508306699 29.0098262648674 26.7836598587251 up 213.0 209.0 196.0 31.0 35.0 25.0

VANGL2 3.14722184268968 4.33012959954128e-42 1.38924991318616e-40 212.001726114207 263.531851878587 228.262920009287 37.8329139441278 24.8655653698863 16.0701959152351 up 199.0 256.0 215.0 40.0 30.0 15.0

BOD1L1 -1.18044644362981 5.19077082772232e-42 1.65908788342672e-40 645.593196106581 619.711620433241 559.509576022763 1325.09781089308 1511.82637448909 1295.25779076795 down 606.0 602.0 527.0 1401.0 1824.0 1209.0

CAMK1 1.65332505095887 5.37534873002152e-42 1.71484759861704e-40 512.426282718259 545.593287092388 574.373208023368 194.839506812258 150.85109657731 174.629462278888 up 481.0 530.0 541.0 206.0 182.0 163.0

TRHDE -1.79244812073561 7.19684807160712e-42 2.29162793859069e-40 122.513560317255 121.471712975286 139.081128005658 425.620281871437 473.274594206836 426.395864950904 down 115.0 118.0 131.0 450.0 571.0 398.0

SUSD1 1.90655343762694 7.87420851980251e-42 2.4979230772557e-40 357.952663187807 366.473981518661 385.39274401568 100.257221951939 101.948818016534 93.2071363083635 up 336.0 356.0 363.0 106.0 123.0 87.0

F2RL2 2.531541076312 8.14761884895646e-42 2.57982548226771e-40 246.092455941617 264.561273174988 242.064864009848 43.5078510357469 45.5868698447916 40.7111629852622 up 231.0 257.0 228.0 46.0 55.0 38.0

DYRK1B -2.30284123376985 8.7078230890173e-42 2.75206199865584e-40 50.0707594340087 80.2948611192571 70.0714080028508 350.900276831785 309.990714944583 329.974689459494 down 47.0 78.0 66.0 371.0 374.0 308.0

SEMA3B 1.02748412696217 1.66401891590162e-41 5.24925147772318e-40 1391.32791108118 1537.95541682269 1464.06775205957 756.658278882555 701.208943430794 697.446502721202 up 1306.0 1494.0 1379.0 800.0 846.0 651.0

FAM132B 2.0312049329332 1.66863974356337e-41 5.25404409962148e-40 341.972633581209 359.268032443856 368.405736014988 71.8825364938428 94.489148405568 95.3498290970615 up 321.0 349.0 347.0 76.0 114.0 89.0

SLC22A18 2.2962998554684 1.72874328828728e-41 5.43319319176001e-40 296.163215375626 382.944722261072 294.087576011965 65.2617765536204 64.6504699617044 68.5661692383363 up 278.0 372.0 277.0 69.0 78.0 64.0

LHX1 -1.13441331443239 2.01630682964987e-41 6.32522920264238e-40 761.714744581197 846.184305641402 669.925128027256 1728.01834439804 1583.93651406176 1691.65595667708 down 715.0 822.0 631.0 1827.0 1911.0 1579.0

ENPP4 3.06282293485099 2.12507013545758e-41 6.65410131139582e-40 227.981755720806 226.472685208161 305.76614401244 23.6455712150799 24.8655653698863 43.9252021683092 up 214.0 220.0 288.0 25.0 30.0 41.0

LRRC41 -1.7565687528093 2.74271197798012e-41 8.5722400197386e-40 128.905572159895 134.854189828496 126.34087200514 469.128132907184 416.912646035094 432.823943316998 down 121.0 131.0 119.0 496.0 503.0 404.0

UBE2L6 -1.19985291178715 3.17243011888497e-41 9.8970471848824e-40 397.37006955075 450.886527823521 410.873256016716 989.330699638941 920.025918685794 985.638682801085 down 373.0 438.0 387.0 1046.0 1110.0 920.0

ADRB2 -1.99134696436578 4.29862844834422e-41 1.33612414522846e-39 79.9001480329926 90.5890740832644 100.860360004103 371.708379501055 354.748732610378 352.472963740823 down 75.0 88.0 95.0 393.0 428.0 329.0

SMOC1 -4.08790966258669 4.92866787708045e-41 1.52079478757273e-39 11.7186883781723 9.26479166760659 12.7402560005183 186.327101174829 187.320592453144 199.270429348915 down 11.0 9.0 12.0 197.0 226.0 186.0

KCNK5 -4.08058522247152 5.3896189432702e-41 1.66000263452722e-39 11.7186883781723 10.2942129640073 12.7402560005183 199.568621055274 171.572401052216 217.483318052848 down 11.0 10.0 12.0 211.0 207.0 203.0

STS -2.84685012756181 5.83459419451347e-41 1.79379356905732e-39 36.2214004416233 25.7355324100183 49.8993360020301 306.446602947435 237.880575371912 259.265827432459 down 34.0 25.0 47.0 324.0 287.0 242.0

RAPGEF2 -1.10309075149018 8.54641913523595e-41 2.62275978534234e-39 492.184911883235 511.622384311164 538.2758160219 1111.34184710875 1139.67174611979 1059.56158401117 down 462.0 497.0 507.0 1175.0 1375.0 989.0

CDKN1C 1.33174312861788 1.11823179129665e-40 3.41928637988544e-39 1408.37327599488 1666.63307887279 1384.44115205633 612.89320589487 502.284420471704 661.020725313336 up 1322.0 1619.0 1304.0 648.0 606.0 617.0

FGFR3 -1.19752137241435 1.320194900013e-40 4.02232043277341e-39 596.587771979678 712.359537109307 573.311520023325 1551.14947170924 1363.46183444877 1404.53512299155 down 560.0 692.0 540.0 1640.0 1645.0 1311.0

FGFBP1 -9.6752072916447 1.62030011505306e-40 4.92780681310571e-39 1.06533530710657 1.02942129640073 2.12337600008639 1151.06640675009 1169.51042456365 1118.48563570036 down 1.0 1.0 2.0 1217.0 1411.0 1044.0

ANKRD20A5P 4.25189231545441 2.55737411937673e-40 7.76378451294655e-39 203.479043657355 165.736828720518 167.746704006825 6.62075994022236 9.94622614795453 11.7848103378391 up 191.0 161.0 158.0 7.0 12.0 11.0

HAPLN4 -1.29903261762658 3.80996157437341e-40 1.15251337624796e-38 480.466223505062 622.799884322443 467.142720019006 1353.47249635117 1254.05334682127 1258.83201336008 down 451.0 605.0 440.0 1431.0 1513.0 1175.0

FLYWCH2 -1.07695910456597 4.87215342655348e-40 1.46337374194709e-38 769.172091730942 865.743310273016 756.983544030798 1832.05885774439 1620.40600993759 1594.16343479132 down 722.0 841.0 713.0 1937.0 1955.0 1488.0

NPDC1 1.38861810938857 6.31398053564182e-40 1.89307664201367e-38 698.859961461909 814.272245452979 701.775768028552 302.663311553022 280.152036500719 262.479866615506 up 656.0 791.0 661.0 320.0 338.0 245.0

CYP4F11 2.14888390632427 6.80828644994276e-40 2.03408064306932e-38 287.640532918773 301.620439845415 324.876528013217 76.6116507368587 67.9658786776893 61.0667444778933 up 270.0 293.0 306.0 81.0 82.0 57.0

GALC 3.33323529364947 2.29180155194431e-39 6.79914505953356e-38 176.84566097969 175.001620388124 196.412280007991 18.9164569720639 18.2347479379166 17.1415423095841 up 166.0 170.0 185.0 20.0 22.0 16.0

ADGRA3 -1.05714445315529 2.48234768496329e-39 7.35156814392974e-38 502.8382649543 526.034282460774 534.029064021727 1091.47956728809 1062.58849347314 1099.20140060208 down 472.0 511.0 503.0 1154.0 1282.0 1026.0

FZD8 -2.35742239841696 2.60530118707046e-39 7.70223422495176e-38 62.8547831192875 51.4710648200366 78.5649120031964 385.895722230103 298.386784438636 303.191029600769 down 59.0 50.0 74.0 408.0 360.0 283.0

CCNG2 -1.14768905033257 2.81492591818299e-39 8.29301653113388e-38 459.159517362931 431.327523191907 494.746608020129 977.0350026071 1065.07505001013 1025.278499392 down 431.0 419.0 466.0 1033.0 1285.0 957.0

NAP1L2 4.18519781186562 3.19385840180402e-39 9.3930488414167e-38 166.192307908625 164.707407424117 202.78240800825 10.4040513346351 4.97311307397726 14.9988495208861 up 156.0 160.0 191.0 11.0 6.0 14.0

ARSJ -1.37678013883265 4.77283722358274e-39 1.3964052256907e-37 243.961785327404 256.325902803782 246.311616010021 663.021816870839 673.027969344923 601.025327229792 down 229.0 249.0 232.0 701.0 812.0 561.0

UPK3B -2.69849850712802 1.41951709292261e-38 4.13172157287095e-37 45.8094182055824 36.0297453740256 26.5422000010799 233.618243604989 232.078610118939 237.838899545479 down 43.0 35.0 25.0 247.0 280.0 222.0

DHX58 -4.24743799351533 1.47507214992089e-38 4.27140550763415e-37 10.6533530710657 6.17652777840439 11.6785680004751 176.868872688797 184.834035916155 177.843501461935 down 10.0 6.0 11.0 187.0 223.0 166.0

RNF144A -1.7870181569036 1.69891112990631e-38 4.90282019431224e-37 165.126972601518 125.589398160889 123.155808005011 427.511927568644 508.915237903673 490.676648611844 down 155.0 122.0 116.0 452.0 614.0 458.0

SVIP -1.72628010169262 2.56019730198062e-38 7.3632839211463e-37 140.624260538067 124.559976864489 174.116832007084 514.527629640138 484.049672533787 453.179524809629 down 132.0 121.0 164.0 544.0 584.0 423.0

CDC42EP1 -4.36921784452017 3.21577156422423e-38 9.21745690320785e-37 10.6533530710657 11.3236342604081 4.24675200017278 186.327101174829 169.914696694223 187.485619011076 down 10.0 11.0 4.0 197.0 205.0 175.0

ZNF608 1.23367253801089 3.53287077721309e-38 1.00922143281601e-36 765.976085809623 710.300694516505 758.045232030841 311.175717190451 328.225462882499 309.619107966862 up 719.0 690.0 714.0 329.0 396.0 289.0

WDR91 -1.1018278217352 3.58162960995999e-38 1.02142770358118e-36 513.491618025366 540.446180610384 622.149168025312 1222.94894324393 1199.34910300752 1174.19564820651 down 482.0 525.0 586.0 1293.0 1447.0 1096.0

RHOBTB1 1.07694884428254 4.82767527600995e-38 1.37446754916989e-36 1163.34615536037 1066.48046307116 1147.68472804669 504.123578305503 566.934890433408 528.17377241406 up 1092.0 1036.0 1081.0 533.0 684.0 493.0

ADRA2C -1.28989054558596 7.56628676575392e-38 2.14694971209165e-36 560.366371538055 637.211782472053 535.09075202177 1572.90339722711 1203.4933639025 1464.53052107509 down 526.0 619.0 504.0 1663.0 1452.0 1367.0

SOX17 -2.24541670652923 1.06536467929032e-37 3.00287481982994e-36 424.003452228414 631.035254693649 526.597248021424 2547.1009312884 2277.68578788159 2677.29463947816 down 398.0 613.0 496.0 2693.0 2748.0 2499.0

TSPAN5 -1.03449026039413 1.08884254669698e-37 3.06395228256591e-36 527.340977017751 571.328819502406 595.606968024232 1169.03704087355 1165.36616366867 1135.62717800995 down 495.0 555.0 561.0 1236.0 1406.0 1060.0

ADAMTS3 1.65404869069244 1.2967707610126e-37 3.64300110971037e-36 434.65680529948 421.0333102279 425.736888017321 148.494187230702 137.589461713371 119.990796167089 up 408.0 409.0 401.0 157.0 166.0 112.0

CRYAB -2.0803549554874 1.56865320570044e-37 4.3995008782393e-36 67.1161243477138 70.0006481552498 71.133096002894 293.20508306699 300.044488796628 287.120833685533 down 63.0 68.0 67.0 310.0 362.0 268.0

UCHL3 1.49239382508445 1.79803088072358e-37 5.03448646602603e-36 591.261095444146 521.916597275171 536.152440021813 188.218746872036 182.347479379166 217.483318052848 up 555.0 507.0 505.0 199.0 220.0 203.0

LOC102724279 1.19752507578794 1.93472942111272e-37 5.4083030352557e-36 1077.05399548474 1177.65796308244 1148.74641604674 561.818772070297 437.633950509999 486.391263034448 up 1011.0 1144.0 1082.0 594.0 528.0 454.0

UBTD1 1.09764843839659 2.08047656748838e-37 5.80614053595604e-36 1530.88683631214 1767.51636592006 1574.48330406406 847.457272348462 702.03779560979 728.515548157324 up 1437.0 1717.0 1483.0 896.0 847.0 680.0

WFDC21P -1.31394927979405 2.26594679745849e-37 6.31334518897153e-36 287.640532918773 273.826064842595 257.990184010496 721.662833484237 654.793221407006 661.020725313336 down 270.0 266.0 243.0 763.0 790.0 617.0

HEATR5A -1.3390341316109 2.8169710918095e-37 7.83571269215975e-36 334.515286431463 289.267384288606 336.555096013693 726.391947727253 880.241014093976 820.651338071338 down 314.0 281.0 317.0 768.0 1062.0 766.0

MDGA1 1.13801588051038 5.25040718800786e-37 1.45329898308584e-35 1316.75443958372 1585.30879645713 1388.6879040565 699.908907966364 590.142751445302 662.092071707685 up 1236.0 1540.0 1308.0 740.0 712.0 618.0

MAGEE1 -2.532787230489 5.2797274890706e-37 1.45903072862734e-35 35.1560651345168 54.5593287092388 36.0973920014686 246.859763485434 234.565166655928 248.552363488969 down 33.0 53.0 34.0 261.0 283.0 232.0

CCDC71L 1.38993087998095 9.36720461022928e-37 2.58016985524039e-35 874.640287134493 1095.30425937038 908.804928036974 421.836990477025 337.342836851458 339.616807008635 up 821.0 1064.0 856.0 446.0 407.0 317.0

MRLN -9.21340286513976 9.98533385534377e-37 2.74151629675079e-35 0 0 4.24675200017278 875.831957806558 851.231187829108 769.226711142586 down 0.0 0.0 4.0 926.0 1027.0 718.0

NMU 1.03840519436468 1.25916480446884e-36 3.44036319156487e-35 1159.08481413195 1073.68641214596 1198.64575204877 542.902315098234 546.213585958503 582.812438525859 up 1088.0 1043.0 1129.0 574.0 659.0 544.0

CRISPLD1 1.86339772861804 1.6688589411962e-36 4.54509171444754e-35 364.344675030446 329.414814848234 382.20768001555 86.0698792228907 110.237339806496 98.5638682801085 up 342.0 320.0 360.0 91.0 133.0 92.0

LYAR -1.29542452190042 1.75254444958648e-36 4.76534558202166e-35 247.157791248724 259.414166692985 256.928496010453 630.863840018331 610.864055920207 633.165719060262 down 232.0 252.0 242.0 667.0 737.0 591.0

CNTNAP1 -2.5534343920149 1.94997801015246e-36 5.29369030320236e-35 36.2214004416233 53.5299074128381 44.5908960018142 294.150905915593 216.330418718011 280.692755319439 down 34.0 52.0 42.0 311.0 261.0 262.0

FBXL21 2.56701773226278 2.44178528244535e-36 6.5971040964313e-35 209.871055499994 222.355000022558 201.720720008207 33.1037997011118 36.4694958758333 37.4971238022152 up 197.0 216.0 190.0 35.0 44.0 35.0

SERINC2 1.24233442760775 2.81733262337459e-36 7.59962016559961e-35 746.800050281705 854.419676012608 850.412088034599 351.846099680388 318.279236734545 368.543159656058 up 701.0 830.0 801.0 372.0 384.0 344.0

ACSL1 -1.08070810144886 3.3110091122259e-36 8.91130888156486e-35 531.602318246178 593.976088023222 624.272544025398 1154.8496981445 1308.75759063502 1236.33373907875 down 499.0 577.0 588.0 1221.0 1579.0 1154.0

SEMA3A -2.57012802115082 4.15697339881066e-36 1.11599254161415e-34 33.0253945203036 46.323958338033 36.0973920014686 231.726597907783 210.528453465037 245.338324305922 down 31.0 45.0 34.0 245.0 254.0 229.0

TMEM178B 1.23792085078296 4.84989152605502e-36 1.2979014605272e-34 807.524162786779 767.948287114946 733.626408029847 333.875465556928 348.946767357405 293.548912051627 up 758.0 746.0 691.0 353.0 421.0 274.0

ZC3HAV1L 2.80441654251739 5.20514088745045e-36 1.39077423712004e-34 186.433678743649 191.472361130536 193.227216007861 23.6455712150799 27.3521219068749 31.0690454361212 up 175.0 186.0 182.0 25.0 33.0 29.0

PKD2 -1.07534619110249 5.41084570576817e-36 1.44346025599548e-34 836.288216078656 782.360185264557 727.256280029588 1514.26238061371 1833.42101993962 1592.02074200262 down 785.0 760.0 685.0 1601.0 2212.0 1486.0

DDX60L -1.36428105697834 7.65439980578754e-36 2.03556566263801e-34 275.921844540601 251.178796321779 274.977192011187 661.130171173633 765.859413392498 634.237065454611 down 259.0 244.0 259.0 699.0 924.0 592.0

PKP2 -1.99222237259766 9.70955193211687e-36 2.57401893161283e-34 99.0761835609109 76.1771759336542 73.2564720029804 320.633945676483 355.577584789374 310.690454361211 down 93.0 74.0 69.0 339.0 429.0 290.0

FBXL5 -1.04679741190721 1.03396102052028e-35 2.73249605110975e-34 550.778353774096 482.798588011943 560.571264022807 1095.2628586825 1099.88684152797 1097.05870781338 down 517.0 469.0 528.0 1158.0 1327.0 1024.0

MSLN -3.28582118340972 1.63148670857393e-35 4.27823294787033e-34 23.4373767563445 23.6766898172168 11.6785680004751 195.785329660861 163.283879262253 216.411971658499 down 22.0 23.0 11.0 207.0 197.0 202.0

RAC2 4.54265224814238 1.72786984555706e-35 4.52397452607986e-34 144.885601766493 153.383773163709 186.857088007602 8.51240563742875 2.48655653698863 10.7134639434901 up 136.0 149.0 176.0 9.0 3.0 10.0

ARRB1 -1.18107375942005 1.92624809310371e-35 5.03559300882359e-34 323.861933360397 326.326550959032 320.629776013045 751.92916463954 707.010908683768 743.51439767821 down 304.0 317.0 302.0 795.0 853.0 694.0

SEZ6L2 1.57368944083702 2.55564266988661e-35 6.67066052817861e-34 517.752959253792 683.535740810086 566.941392023066 221.322546573147 186.491740274147 186.414272616727 up 486.0 664.0 534.0 234.0 225.0 174.0

NCAM1 7.92278466128269 2.89195900414034e-35 7.53689008155959e-34 1132.45143145428 1053.09798621795 1106.27889604501 0.945822848603194 9.94622614795453 2.14269278869801 up 1063.0 1023.0 1042.0 1.0 12.0 2.0

LOC389906 -2.03292529114671 3.75982583741803e-35 9.76862725243272e-34 60.7241125050744 94.7067592688674 81.749976003326 332.929642708324 303.359897512613 336.402767825588 down 57.0 92.0 77.0 352.0 366.0 314.0

HS3ST3A1 -1.23486309989397 3.91362652295517e-35 1.0152654410239e-33 461.290187977144 577.505347280811 467.142720019006 1294.83147973777 1070.04816308411 1182.7664193613 down 433.0 561.0 440.0 1369.0 1291.0 1104.0

LINC01508 2.75633684355263 4.30912559164212e-35 1.11615577251403e-33 230.112426335019 213.090208354952 191.103840007775 32.1579768525086 21.5501566539015 41.7825093796112 up 216.0 207.0 180.0 34.0 26.0 39.0

LPPR3 3.46287011687635 5.51259372691622e-35 1.42569981273223e-33 160.865631373092 241.914004654172 183.672024007473 23.6455712150799 8.28852178996211 22.4982742813291 up 151.0 235.0 173.0 25.0 10.0 21.0

C3orf70 5.58661502325457 5.77674885775061e-35 1.49173972027889e-33 171.518984444158 190.442939834135 179.4252720073 1.89164569720639 4.14426089498105 5.35673197174503 up 161.0 185.0 169.0 2.0 5.0 5.0

PPP2R3B -1.72864121398738 5.95823975149317e-35 1.5362645569299e-33 167.257643215731 154.41319446011 109.353864004449 491.827881273661 440.120507046988 498.176073372287 down 157.0 150.0 103.0 520.0 531.0 465.0

CACNG4 -3.5691330006651 6.56722296239719e-35 1.68814502250392e-33 17.0453649137051 9.26479166760659 15.9253200006479 163.627352808353 174.058957589204 161.7733055467 down 16.0 9.0 15.0 173.0 210.0 151.0

SNAI2 -1.11095637478674 7.70826821095396e-35 1.97845550747818e-33 418.676775692881 465.298425973131 412.996632016803 960.010191332242 881.069866272972 963.140408519756 down 393.0 452.0 389.0 1015.0 1063.0 899.0

GBP5 -3.0611816728786 8.4982364004057e-35 2.1746242390162e-33 26.6333826776642 18.5295833352132 25.4805120010367 162.681529959749 200.582227317083 226.05408920764 down 25.0 18.0 24.0 172.0 242.0 211.0

DHRSX -2.73450342572031 8.62050842987626e-35 2.20258541179644e-33 41.5480769771562 30.882638892022 26.5422000010799 200.514443903877 203.068783854072 256.051788249412 down 39.0 30.0 25.0 212.0 245.0 239.0

ZFX -1.25478664421548 8.99334974440375e-35 2.29093751383759e-33 474.074211662423 412.797939856694 383.269368015593 937.310442965766 1131.38322432983 958.85502294236 down 445.0 401.0 361.0 991.0 1365.0 895.0

LRRC17 -1.84831442554822 9.04695237474903e-35 2.30113173015388e-33 112.925542553296 103.971550936474 84.9350400034556 331.983819859721 392.047080665208 362.115081289964 down 106.0 101.0 80.0 351.0 473.0 338.0

GABARAPL1 1.06867686107179 1.25803651699561e-34 3.1950732530593e-33 965.193788238551 907.949583425446 975.691272039696 474.803069998804 425.201167825056 459.607603175723 up 906.0 882.0 919.0 502.0 513.0 429.0

EPS8 1.03022714826313 1.30918516491377e-34 3.31999950503582e-33 1025.91790074363 941.92048620667 990.5549040403 473.8572471502 478.247707280813 497.104726977938 up 963.0 915.0 933.0 501.0 577.0 464.0

IRF2 -1.50310995248178 1.68673665591181e-34 4.23936482954689e-33 162.996301987305 153.383773163709 193.227216007861 482.369652787629 477.418855101817 484.24857024575 down 153.0 149.0 182.0 510.0 576.0 452.0

VGF 4.00676994911197 1.73584761464837e-34 4.35633460624346e-33 139.55892523096 190.442939834135 138.019440005615 9.45822848603194 9.11737396895832 10.7134639434901 up 131.0 185.0 130.0 10.0 11.0 10.0

ZBTB18 1.2222638271287 2.26867167746424e-34 5.6683330702425e-33 829.896204236017 704.124166738101 785.649120031964 347.116985437372 311.648419302575 336.402767825588 up 779.0 684.0 740.0 367.0 376.0 314.0

CCAT1 -6.51206304416973 3.059148665054e-34 7.62087917441393e-33 2.13067061421314 5.14710648200366 1.06168800004319 276.180271792133 263.574992920795 227.125435601989 down 2.0 5.0 1.0 292.0 318.0 212.0

RBM20 4.69481306215758 3.32118262907969e-34 8.26150275133772e-33 160.865631373092 138.971875014099 165.623328006738 3.78329139441278 3.31540871598484 11.7848103378391 up 151.0 135.0 156.0 4.0 4.0 11.0

WDR17 2.86769564476643 5.95102086751409e-34 1.47383470022937e-32 186.433678743649 180.148726870128 170.931768006954 25.5372169122862 19.0636001169128 29.9976990417721 up 175.0 175.0 161.0 27.0 23.0 28.0

HCLS1 -3.57875116063148 7.84908035426136e-34 1.93542097818322e-32 10.6533530710657 20.5884259280146 9.55519200038875 161.735707111146 164.941583620246 162.844651941049 down 10.0 20.0 9.0 171.0 199.0 152.0

ALDH3A1 -8.86835205861991 8.86999570637089e-34 2.18397859398144e-32 3.19600592131971 0 1.06168800004319 780.303850097635 575.22341222337 611.738791173282 down 3.0 0.0 1.0 825.0 694.0 571.0

CHST9 3.30895977474974 1.31026362525481e-33 3.20749505951105e-32 188.564349357863 146.177824088904 182.610336007429 15.1331655776511 11.6039305059469 26.7836598587251 up 177.0 142.0 172.0 16.0 14.0 25.0

SNCA 2.84241129164455 2.37381458509062e-33 5.78595957862375e-32 167.257643215731 173.972199091724 194.288904007905 23.6455712150799 29.8386784438636 20.3555814926311 up 157.0 169.0 183.0 25.0 36.0 19.0

LPXN -2.21472241931076 3.11928598794772e-33 7.59205526376932e-32 51.1360947411153 48.3828009308344 56.2694640022893 234.564066453592 256.115323309829 231.410821179385 down 48.0 47.0 53.0 248.0 309.0 216.0

CAMK2D -1.73933316788143 4.25666525274153e-33 1.03306460431865e-31 101.206854175124 114.265763900481 104.045424004233 339.550402648547 388.731671949223 337.474114219937 down 95.0 111.0 98.0 359.0 469.0 315.0

TBC1D4 1.21066823162904 4.39078321408393e-33 1.06408966590246e-31 845.876233842615 780.301342671755 884.386104035981 314.959008584864 392.875932844204 376.042584416501 up 794.0 758.0 833.0 333.0 474.0 351.0

WWC3 -1.22831598648604 5.15435555078297e-33 1.24735404328948e-31 494.315582497448 371.621088000664 420.428448017105 1093.37121298529 958.153118919619 960.997715731058 down 464.0 361.0 396.0 1156.0 1156.0 897.0

SUSD2 -3.50065178404351 5.69561131986739e-33 1.37637169413058e-31 23.4373767563445 11.3236342604081 10.6168800004319 159.84406141394 153.337653114299 200.341775743264 down 22.0 11.0 10.0 169.0 185.0 187.0

ZNF469 3.89879089955529 6.69031234499379e-33 1.61444289350705e-31 147.016272380706 160.589722238514 135.896064005529 11.3498741832383 14.0904870429356 3.21403918304702 up 138.0 156.0 128.0 12.0 17.0 3.0

MUC5AC -4.24432622764197 7.71602547484592e-33 1.8566686298848e-31 6.39201184263941 9.26479166760659 9.55519200038875 188.218746872036 160.797322725265 128.561567321881 down 6.0 9.0 9.0 199.0 194.0 120.0

SNAI1 1.63999030710085 8.4251643201472e-33 2.02156209041492e-31 400.56607547207 398.386041707083 339.740160013822 121.065324621209 119.354713775454 125.347528138834 up 376.0 387.0 320.0 128.0 144.0 117.0

ARHGAP6 4.58478281450388 1.08829635653155e-32 2.60024545552107e-31 129.970907467001 144.118981496103 153.944760006263 5.67493709161917 5.80196525297347 6.42807836609403 up 122.0 140.0 145.0 6.0 7.0 6.0

VASH1 -1.5097708828221 1.17198639783069e-32 2.79232764827734e-31 181.107002208117 162.648564831316 142.266192005788 435.078510357469 464.157220237878 485.319916640099 down 170.0 158.0 134.0 460.0 560.0 453.0

RUNX1T1 6.07273385626763 1.552923967313e-32 3.6740966489221e-31 182.172337515223 185.295833352132 201.720720008207 1.89164569720639 2.48655653698863 4.28538557739602 up 171.0 180.0 190.0 2.0 3.0 4.0

TMEM198 2.26664410015708 1.59809647892687e-32 3.77043932493331e-31 200.283037736035 236.766898172168 248.434992010107 45.3994967329533 49.7311307397726 47.1392413513562 up 188.0 230.0 234.0 48.0 60.0 44.0

LRCH2 2.18736191545653 1.82528494609064e-32 4.30046272416904e-31 349.429980730954 262.502430582187 252.68174401028 69.0450679480332 52.2176872767613 69.6375156326853 up 328.0 255.0 238.0 73.0 63.0 65.0

NSG1 -3.398746928281 1.84227610084769e-32 4.33446627060554e-31 17.0453649137051 15.441319446011 11.6785680004751 157.00659286813 156.653061830284 152.131187997559 down 16.0 15.0 11.0 166.0 189.0 142.0

PSD3 1.26415278968542 1.98449700086616e-32 4.6626046039768e-31 920.449705340075 753.536388965336 802.636128032655 300.771665855816 363.866106579336 366.40046686736 up 864.0 732.0 756.0 318.0 439.0 342.0

LOXL1-AS1 -1.89296048571365 2.55576117605034e-32 5.98818731981918e-31 87.3574951827386 73.088912044452 114.662304004665 340.49622549715 358.892993505359 320.332571910353 down 82.0 71.0 108.0 360.0 433.0 299.0

HDHD1 -1.42645234054055 3.18330937045531e-32 7.40731603509793e-31 198.152367121822 183.23699075933 160.314888006522 504.123578305503 478.247707280813 473.53510630226 down 186.0 178.0 151.0 533.0 577.0 442.0

TK2 -1.04715314121975 3.96620120905855e-32 9.19116942290724e-31 412.284763850242 416.915625042297 441.662208017969 854.078032288685 854.546596545093 919.215206351446 down 387.0 405.0 416.0 903.0 1031.0 858.0

PPM1E 1.52795932079177 4.03514518471385e-32 9.33816385642794e-31 458.094182055824 438.533472266712 401.318064016327 132.415198804447 153.337653114299 164.987344729747 up 430.0 426.0 378.0 140.0 185.0 154.0

AMOT 1.70210979131301 4.89997188633855e-32 1.12932685380374e-30 383.520710558365 325.297129662631 394.947936016068 108.769627589367 125.156679028428 103.920600251854 up 360.0 316.0 372.0 115.0 151.0 97.0

ANKRD34B -2.54039526533528 5.53690020408403e-32 1.27438980240738e-30 37.2867357487299 36.0297453740256 28.6655760011662 212.810140935719 188.978296811136 191.771004588472 down 35.0 35.0 27.0 225.0 228.0 179.0

XYLT1 -3.43987695815463 7.59538938214678e-32 1.74108113847857e-30 18.1107002208117 14.4118981496103 12.7402560005183 185.381278326226 130.958644281401 175.700808673237 down 17.0 14.0 12.0 196.0 158.0 164.0

MAPT -1.26202125240552 9.1274576261528e-32 2.08662796473723e-30 243.961785327404 230.590370393764 250.558368010194 561.818772070297 564.448333896419 613.88148396198 down 229.0 224.0 236.0 594.0 681.0 573.0

ITGA4 1.51055060682887 1.1600846272923e-31 2.64137548203381e-30 412.284763850242 412.797939856694 410.873256016716 129.577730258638 142.562574787348 162.844651941049 up 387.0 401.0 387.0 137.0 172.0 152.0

PLA2G16 -1.09496834771134 2.09000185809008e-31 4.71433175446684e-30 405.892752007603 353.091504665451 412.996632016803 802.057775615509 839.627257323161 861.3625010566 down 381.0 343.0 389.0 848.0 1013.0 804.0

ATP5I -1.05644354351232 2.94364516792781e-31 6.61344153112693e-30 564.627712766481 523.975439867973 632.766048025744 1109.45020141155 1173.65468545863 1298.47182995099 down 530.0 509.0 596.0 1173.0 1416.0 1212.0

FAT4 -2.1890826990494 3.26698301498013e-31 7.33015791705475e-30 54.332100662435 55.5887500056395 41.4058320016846 229.834952210576 226.276644865965 234.624860362432 down 51.0 54.0 39.0 243.0 273.0 219.0

PIK3R3 1.58251527948386 4.09736660801298e-31 9.18113628832538e-30 462.355523284251 437.504050970311 397.071312016155 118.227856075399 152.508800935303 162.844651941049 up 434.0 425.0 374.0 125.0 184.0 152.0

HRCT1 -2.12204491408651 4.19397039899497e-31 9.38518607119878e-30 60.7241125050744 51.4710648200366 58.3928400023757 236.455712150799 229.59205358195 277.478716136392 down 57.0 50.0 55.0 250.0 277.0 259.0

OTUD4 -1.05168883921947 4.55785871300343e-31 1.01860325327544e-29 713.774655761401 566.181713020403 608.34722402475 1213.4907147579 1376.72346931271 1322.04145062667 down 670.0 550.0 573.0 1283.0 1661.0 1234.0

TMEM33 -1.08954892107696 4.62712010750319e-31 1.03271956022535e-29 602.979783822318 508.534120421962 492.623232020042 1165.25374947914 1207.63762479748 1037.06330972984 down 566.0 494.0 464.0 1232.0 1457.0 968.0

HES1 -1.11115998185832 5.2293291076093e-31 1.16558993530134e-29 327.057939281717 354.120925961852 333.370032013563 695.179793723348 740.993848022612 756.370554410398 down 307.0 344.0 314.0 735.0 894.0 706.0

CAMK4 2.11407222002149 7.11316322140505e-31 1.58132526208139e-29 235.439102870552 232.649212986565 235.694736009589 45.3994967329533 63.8216177827082 52.4959733231013 up 221.0 226.0 222.0 48.0 77.0 49.0

COL23A1 -5.26169056330859 7.79558010995544e-31 1.73076182257726e-29 3.19600592131971 4.11768518560293 5.30844000021597 165.518998505559 168.256992336231 149.988495208861 down 3.0 4.0 5.0 175.0 203.0 140.0

ABCB1 5.05062745007857 8.08026441193996e-31 1.79161883688826e-29 153.408284223346 162.648564831316 118.909056004838 5.67493709161917 4.14426089498105 3.21403918304702 up 144.0 158.0 112.0 6.0 5.0 3.0

MAFF -1.53936625072376 8.80023646468058e-31 1.94616195446069e-29 126.774901545682 128.677662050092 127.402560005183 370.762556652452 361.379550042348 381.399316388246 down 119.0 125.0 120.0 392.0 436.0 356.0

XIST -13.7130126532434 9.63244131093515e-31 2.12210377949749e-29 0 0 0 2898.00120812019 2697.08499045367 2199.47414759851 down 0.0 0.0 0.0 3064.0 3254.0 2053.0

UNC13D 1.38819875070163 1.5054626544037e-30 3.29916395415248e-29 574.215730530441 741.183333408527 564.818016022979 244.968117788227 232.907462297935 241.052938728526 up 539.0 720.0 532.0 259.0 281.0 225.0

KLHL8 -1.11514767930747 1.57771826766116e-30 3.4530423067416e-29 363.27933972334 367.503402815061 340.801848013865 710.312959300999 810.617431058294 800.295756578707 down 341.0 357.0 321.0 751.0 978.0 747.0

MMP15 -1.09556906145784 1.65441067538827e-30 3.61622152788094e-29 615.763807507597 826.625301009788 700.714080028508 1658.0274536014 1438.05853055843 1485.95744896207 down 578.0 803.0 660.0 1753.0 1735.0 1387.0

SCARA3 -1.20717666961692 1.94046025342723e-30 4.23055298494946e-29 283.379191690347 242.943425950573 279.22394401136 628.026371472521 633.243064753105 596.739941652396 down 266.0 236.0 263.0 664.0 764.0 557.0

SCN2A -1.60447401840061 3.19783909292554e-30 6.92728826523768e-29 132.101578081215 118.383449086084 138.019440005615 441.699270297692 383.758558875245 354.615656529521 down 124.0 115.0 130.0 467.0 463.0 331.0

IFT27 2.71094441619267 4.05066379152456e-30 8.75232712097271e-29 169.388313829944 148.236666681705 191.103840007775 25.5372169122862 28.1809740858712 23.5696206756781 up 159.0 144.0 180.0 27.0 34.0 22.0

MATK -1.21573033038275 4.34867581131954e-30 9.37233692668614e-29 263.137820855322 336.620763923039 281.347320011447 683.82991954011 702.866647788787 659.949378918987 down 247.0 327.0 265.0 723.0 848.0 616.0

CDKN1A 1.52785637311912 4.49655019755108e-30 9.67872431340727e-29 414.415434464455 440.592314859513 449.094024018271 169.302289899972 160.797322725265 119.990796167089 up 389.0 428.0 423.0 179.0 194.0 112.0

HERC6 -1.18185409261588 4.90924976611733e-30 1.05536409946735e-28 339.841962966995 288.237962992205 294.087576011965 720.717010635634 722.759100084696 646.02187579245 down 319.0 280.0 277.0 762.0 872.0 603.0

PRKG1 5.46167270201537 5.72485747013796e-30 1.22758336131819e-28 168.322978522838 146.177824088904 138.019440005615 0.945822848603194 6.63081743196968 2.14269278869801 up 158.0 142.0 130.0 1.0 8.0 2.0

KALRN 1.30798396415254 6.54161933097674e-30 1.39917969023669e-28 487.923570654808 557.946342649197 559.509576022763 233.618243604989 202.239931675075 213.197932475452 up 458.0 542.0 527.0 247.0 244.0 199.0

NUDT11 1.28132163064194 9.30826711610554e-30 1.97844472957124e-28 493.250247190341 542.505023203186 508.54855202069 199.568621055274 209.699601286041 227.125435601989 up 463.0 527.0 479.0 211.0 253.0 212.0

MSR1 -2.02626180141332 1.12678730312292e-29 2.38299337264698e-28 61.789447812181 70.0006481552498 55.2077760022461 230.780775059179 280.980888679715 249.623709883318 down 58.0 68.0 52.0 244.0 339.0 233.0

EDN2 2.13677197892551 1.13703847320706e-29 2.39868390238202e-28 194.956361200502 245.002268543374 235.694736009589 51.0744338245725 53.0465394557575 49.2819341400542 up 183.0 238.0 222.0 54.0 64.0 46.0

NBEA 1.12974166065076 1.40331331469714e-29 2.95305932310182e-28 645.593196106581 617.652777840439 606.223848024664 289.421791672577 285.954001753693 278.550062530741 up 606.0 600.0 571.0 306.0 345.0 260.0

B4GALNT1 1.51145861966361 1.62109834099787e-29 3.4071223196655e-28 377.128698715725 451.915949119921 377.960928015377 148.494187230702 135.931757355379 139.275031265371 up 354.0 439.0 356.0 157.0 164.0 130.0

PDGFD 2.15782641193085 2.04082246300898e-29 4.27865501526882e-28 223.720414492379 203.825416687345 202.78240800825 48.2369652787629 50.5599829187688 41.7825093796112 up 210.0 198.0 191.0 51.0 61.0 39.0

C2orf88 -2.09639349694731 2.11156036351372e-29 4.42148733719683e-28 43.6787475913693 57.647592598441 59.4545280024189 226.051660816163 234.565166655928 227.125435601989 down 41.0 56.0 56.0 239.0 283.0 212.0

CD55 -1.0453445679981 2.63961945310618e-29 5.50679230734219e-28 359.017998494914 346.914976887047 352.480416014341 734.904353364682 711.984021757745 738.157665706465 down 337.0 337.0 332.0 777.0 859.0 689.0

KLHDC8B -1.39773233697316 2.68938429645235e-29 5.60371094488349e-28 167.257643215731 208.972523169349 165.623328006738 496.556995516677 455.03984626892 477.820491879656 down 157.0 203.0 156.0 525.0 549.0 446.0

RGS14 1.71649235452228 2.85456024223727e-29 5.92601109111512e-28 287.640532918773 380.885879668271 335.493408013649 98.3655762547322 105.264226732519 101.777907463156 up 270.0 370.0 316.0 104.0 127.0 95.0

SFN -1.09009958758657 3.06645328835038e-29 6.35810510460898e-28 402.696746086283 504.416435236359 485.19141601974 1043.24260200932 913.395101253824 1010.27964987111 down 378.0 490.0 457.0 1103.0 1102.0 943.0

GCHFR -2.87449364547619 6.17720766863528e-29 1.26992594546944e-27 22.3720414492379 19.5590046316139 21.2337600008639 162.681529959749 144.220279145341 156.416573574955 down 21.0 19.0 20.0 172.0 174.0 146.0

FOXO1 -1.80439786962052 6.71388965659166e-29 1.3752514000322e-27 86.2921598756321 90.5890740832644 71.133096002894 266.722043306101 290.92711482767 309.619107966862 down 81.0 88.0 67.0 282.0 351.0 289.0

TTLL7 2.52319494962702 8.4662846521947e-29 1.73002246089479e-27 173.649655058371 170.883935202522 170.931768006954 25.5372169122862 26.5232697278787 38.5684701965642 up 163.0 166.0 161.0 27.0 32.0 36.0

PAOX 1.55176329758207 8.98188751538876e-29 1.83317077723718e-27 343.037968888315 438.533472266712 391.762872015939 122.956970318415 140.07601825036 137.132338476673 up 322.0 426.0 369.0 130.0 169.0 128.0

AP1M2 1.55937560928992 9.13672732024023e-29 1.8602903942893e-27 366.47534564466 449.85710652712 427.860264017407 165.518998505559 130.129792102405 126.418874533183 up 344.0 437.0 403.0 175.0 157.0 118.0

CASC8 -4.09706237019984 1.00026325669333e-28 2.0341488077293e-27 7.45734714974598 9.26479166760659 6.37012800025917 120.119501772606 128.472087744413 147.845802420163 down 7.0 9.0 6.0 127.0 155.0 138.0

FAM167A -1.03803417731724 1.17315166254149e-28 2.38002265430574e-27 381.390039944152 407.65083337469 379.02261601542 847.457272348462 798.184648373351 752.085168833002 down 358.0 396.0 357.0 896.0 963.0 702.0

FAHD2B 2.431837214053 1.18180058251589e-28 2.39470118036115e-27 173.649655058371 198.678310205341 167.746704006825 28.3746854580958 38.1272002338257 33.2117382248192 up 163.0 193.0 158.0 30.0 46.0 31.0

ARMCX6 1.75141261160348 1.28050028742286e-28 2.59159795327877e-27 296.163215375626 338.679606515841 376.899240015334 118.227856075399 84.5429222576135 98.5638682801085 up 278.0 329.0 355.0 125.0 102.0 92.0

PALD1 -3.55748166480786 1.30941912710015e-28 2.64696420203777e-27 17.0453649137051 10.2942129640073 7.43181600030236 145.656718684892 133.44520081839 129.63291371623 down 16.0 10.0 7.0 154.0 161.0 121.0

MAP1LC3C 3.05661539631192 1.41584232571312e-28 2.85868522021219e-27 141.689595845174 131.765925939294 152.88307200622 15.1331655776511 22.3790088328977 12.8561567321881 up 133.0 128.0 144.0 16.0 27.0 12.0

EBP -2.14816575745282 1.53120316368606e-28 3.08792638010022e-27 57.5281065837547 41.1768518560293 72.1947840029372 282.801031732355 246.997949340871 226.05408920764 down 54.0 40.0 68.0 299.0 298.0 211.0

SPANXB1 -13.0468281745021 2.20647092838036e-28 4.42862766904778e-27 0 0 0 1775.3094868282 1610.45978378964 1525.59726555298 down 0.0 0.0 0.0 1877.0 1943.0 1424.0

PLAG1 3.86031119238133 2.44896117389481e-28 4.90371185411089e-27 148.081607687813 127.648240753691 104.045424004233 6.62075994022236 11.6039305059469 7.49942476044304 up 139.0 124.0 98.0 7.0 14.0 7.0

BTN3A3 -1.32436855363467 2.61956923480562e-28 5.23296024028386e-27 188.564349357863 169.854513906121 192.165528007818 435.078510357469 470.788037669848 472.463759907911 down 177.0 165.0 181.0 460.0 568.0 441.0

TRIQK 1.41645294076922 2.94769878816548e-28 5.86768712943869e-27 369.671351565979 387.062407446675 394.947936016068 151.331655776511 141.733722608352 138.203684871022 up 347.0 376.0 372.0 160.0 171.0 129.0

SLC39A11 1.46209448896734 3.24516383148122e-28 6.45223888559764e-27 373.932692794406 399.415463003484 401.318064016327 125.794438864225 159.139618367272 140.34637765972 up 351.0 388.0 378.0 133.0 192.0 131.0

PADI2 1.33544387819976 4.91036447055087e-28 9.66104229165294e-27 448.506164291865 492.06337967955 513.856992020906 210.918495238512 170.743548873219 196.056390165868 up 421.0 478.0 484.0 223.0 206.0 183.0

N4BP3 -1.44227291002829 5.84112404219465e-28 1.14656594756405e-26 160.865631373092 167.795671313319 132.711000005399 392.516482170326 416.912646035094 445.680100049186 down 151.0 163.0 125.0 415.0 503.0 416.0

BMP7 5.06175881720942 6.04324578789298e-28 1.18486786628365e-26 99.0761835609109 152.354351867308 153.944760006263 5.67493709161917 4.97311307397726 1.07134639434901 up 93.0 148.0 145.0 6.0 6.0 1.0

KRT17 -12.9228190453477 6.4119755757507e-28 1.25425942555678e-26 0 0 1.06168800004319 3082.43666359781 2871.14394804287 2826.21178829268 down 0.0 0.0 1.0 3259.0 3464.0 2638.0

REEP6 1.61047800421059 6.42557296934152e-28 1.25546950519775e-26 370.736686873086 345.885555590646 321.691464013088 122.011147469812 94.489148405568 125.347528138834 up 348.0 336.0 303.0 129.0 114.0 117.0

SNX25 -1.14282984605668 6.44915081510958e-28 1.25862459456171e-26 275.921844540601 248.090532432576 265.422000010799 586.410166133981 572.736855686381 583.883784920208 down 259.0 241.0 250.0 620.0 691.0 545.0

CTH -2.18272821398482 6.54007512828948e-28 1.27490072121086e-26 46.874753512689 37.0591666704264 50.9610240020733 199.568621055274 201.411079496079 211.055239686754 down 44.0 36.0 48.0 211.0 243.0 197.0

RELN 6.71442094200681 6.81014224459014e-28 1.32602080026847e-26 205.609714271568 186.325254648533 199.597344008121 1.89164569720639 1.65770435799242 2.14269278869801 up 193.0 181.0 188.0 2.0 2.0 2.0

DEFB1 7.31877710557728 9.68725588990597e-28 1.88406561165335e-26 237.569773484765 198.678310205341 315.321336012829 0 0.82885217899621 4.28538557739602 up 223.0 193.0 297.0 0.0 1.0 4.0

CALB1 -12.8794601690081 1.04412109916638e-27 2.02837286925212e-26 0 0 0 1412.11351296457 1544.98046164894 1415.24858693504 down 0.0 0.0 0.0 1493.0 1864.0 1321.0

DLGAP3 2.35862390061539 1.12099812132413e-27 2.17039518849054e-26 171.518984444158 181.178148166529 231.447984009416 39.7245596413342 42.2714611288067 31.0690454361212 up 161.0 176.0 218.0 42.0 51.0 29.0

FAR2P1 4.55857785404347 1.38459367773376e-27 2.67446030796008e-26 119.317554395936 112.20692130768 122.094120004967 5.67493709161917 4.97311307397726 4.28538557739602 up 112.0 109.0 115.0 6.0 6.0 4.0

CCDC144NL-AS1 5.94879047769577 1.71948569930086e-27 3.31000997115415e-26 151.277613609133 138.971875014099 171.993456006997 1.89164569720639 4.14426089498105 1.07134639434901 up 142.0 135.0 162.0 2.0 5.0 1.0

CA8 4.32601038604316 3.60839854739238e-27 6.89134964969864e-26 132.101578081215 126.61881945729 101.922048004147 2.83746854580958 4.14426089498105 11.7848103378391 up 124.0 123.0 96.0 3.0 5.0 11.0

FBXO32 1.47205293444843 3.97737588328944e-27 7.57893672248854e-26 394.17406362943 397.356620410683 373.714176015204 124.848616015622 130.129792102405 167.130037518445 up 370.0 386.0 352.0 132.0 157.0 156.0

LRRC61 -1.23609721112954 4.73457087486186e-27 8.98136961032025e-26 224.785749799486 200.737152798143 198.535656008077 506.015224002709 476.590002922821 487.462609428797 down 211.0 195.0 187.0 535.0 575.0 455.0

DAW1 2.49367675164722 8.46192207955771e-27 1.58567433658969e-25 175.780325672584 160.589722238514 167.746704006825 39.7245596413342 22.3790088328977 27.8550062530741 up 165.0 156.0 158.0 42.0 27.0 26.0

PAQR5 -1.46513637568502 9.18638811642514e-27 1.71763150874439e-25 144.885601766493 135.883611124897 144.389568005874 385.895722230103 435.976246152007 349.258924557776 down 136.0 132.0 136.0 408.0 526.0 326.0

MIR100HG -3.07497012101783 9.31678228825983e-27 1.74009142186463e-25 18.1107002208117 22.6472685208161 9.55519200038875 128.631907410034 147.535687861325 148.917148814512 down 17.0 22.0 9.0 136.0 178.0 139.0

HDAC4 -1.03051777795067 1.09532928619618e-26 2.03899759430367e-25 339.841962966995 347.944398183448 332.30834401352 709.367136452396 716.128282652726 656.73533973594 down 319.0 338.0 313.0 750.0 864.0 613.0

SOGA3 2.60223593008028 1.10265614771163e-26 2.05038365995994e-25 173.649655058371 177.060462980926 135.896064005529 23.6455712150799 33.1540871598484 22.4982742813291 up 163.0 172.0 128.0 25.0 40.0 21.0

C9orf89 1.01035620675506 1.14866570142438e-26 2.13359616032116e-25 1343.38782226138 1638.83870386997 1384.44115205633 809.624358404334 650.648960512025 709.231313059041 up 1261.0 1592.0 1304.0 856.0 785.0 662.0

IFI27 -2.35381000079742 1.31475662049204e-26 2.43675898808919e-25 28.7640532918774 52.5004861164373 48.8376480019869 220.376723724544 262.746140741799 181.057540644982 down 27.0 51.0 46.0 233.0 317.0 169.0

RAB6B 1.07623006270911 1.33068998962618e-26 2.46359436330791e-25 659.442555098966 802.948611192571 745.304976030322 356.575213923404 350.604471715397 339.616807008635 up 619.0 780.0 702.0 377.0 423.0 317.0

HOXB5 -1.12617222107948 1.47945843253615e-26 2.73006817507216e-25 252.484467784257 274.855486138996 266.483688010842 554.252189281472 606.719795025226 571.02762818802 down 237.0 267.0 251.0 586.0 732.0 533.0

TMEM132B -2.43545658535726 1.59800102754083e-26 2.94240623984148e-25 38.3520710558365 25.7355324100183 30.7889520012526 158.898238565337 179.860922842178 173.558115884539 down 36.0 25.0 29.0 168.0 217.0 162.0

LINC01605 4.10500205459427 1.69541635527368e-26 3.11838795421673e-25 105.46819540355 110.148078714878 135.896064005529 4.72911424301597 11.6039305059469 3.21403918304702 up 99.0 107.0 128.0 5.0 14.0 3.0

TRIM6 1.00075166013061 1.82840802144509e-26 3.35571309678004e-25 1058.94329526393 882.214051015428 1021.34385604155 497.50281836528 523.834577125605 456.393563992676 up 994.0 857.0 962.0 526.0 632.0 426.0

ALX4 2.82943841516011 1.96043312561838e-26 3.59412739696703e-25 140.624260538067 181.178148166529 141.204504005745 30.2663311553022 14.0904870429356 21.4269278869801 up 132.0 176.0 133.0 32.0 17.0 20.0

SYNDIG1 1.55506535243741 2.10668743353387e-26 3.85808487827715e-25 319.600592131971 370.591666704264 341.863536013909 113.498741832383 135.102905176382 100.706561068806 up 300.0 360.0 322.0 120.0 163.0 94.0

SPOCK1 -1.02022612318682 2.12613944566247e-26 3.88950347835013e-25 457.028846748718 423.092152820701 439.538832017882 794.491192826683 917.539362148805 965.283101308454 down 429.0 411.0 414.0 840.0 1107.0 901.0

MAML2 -1.12676951133476 2.28652689549911e-26 4.16940426369805e-25 410.154093236029 308.82638892022 340.801848013865 747.200050396524 815.590544132271 748.871129649955 down 385.0 300.0 321.0 790.0 984.0 699.0

THNSL2 3.60601492688563 2.38161729246014e-26 4.33812870261019e-25 124.644230931469 111.177500011279 106.168800004319 8.51240563742875 10.7750783269507 8.57077115479204 up 117.0 108.0 100.0 9.0 13.0 8.0

LINC01139 1.00361668550028 2.9933903207779e-26 5.4407759693109e-25 779.825444802008 732.947963037321 856.782216034858 394.408127867532 402.822158992158 383.542009176944 up 732.0 712.0 807.0 417.0 486.0 358.0

NRXN3 7.80254697538439 3.08591102420143e-26 5.60292955519532e-25 259.941814934003 286.179120399404 291.964200011878 1.89164569720639 0.82885217899621 1.07134639434901 up 244.0 278.0 275.0 2.0 1.0 1.0

NOV -1.66017220274916 3.20867871491599e-26 5.80096237253756e-25 93.749507025378 90.5890740832644 112.538928004579 287.530145975371 298.386784438636 353.544310135172 down 88.0 88.0 106.0 304.0 360.0 330.0

SOCS2 1.2738475715105 3.64160830285296e-26 6.57663589022699e-25 434.65680529948 422.0627315243 466.081032018962 193.893683963655 171.572401052216 182.128887039331 up 408.0 410.0 439.0 205.0 207.0 170.0

CNOT6L -1.16610911933307 3.79278810048328e-26 6.83508834278583e-25 355.821992573594 281.0320139174 317.444712012915 649.780296990395 784.094161330415 704.945927481645 down 334.0 273.0 299.0 687.0 946.0 658.0

GPR3 -2.32547242538514 7.88368138289144e-26 1.41172899182009e-24 29.8293885989839 36.0297453740256 36.0973920014686 177.814695537401 172.401253231212 160.701959152351 down 28.0 35.0 34.0 188.0 208.0 150.0

EPHA5 3.22335417263358 8.60760026851802e-26 1.53973335320692e-24 151.277613609133 126.61881945729 112.538928004579 9.45822848603194 20.7213044749053 10.7134639434901 up 142.0 123.0 106.0 10.0 25.0 10.0

CLEC4E -5.25679748133763 9.44123099413168e-26 1.68529455258789e-24 7.45734714974598 1.02942129640073 2.12337600008639 144.710895836289 134.274052997386 123.204835350136 down 7.0 1.0 2.0 153.0 162.0 115.0

SLAIN1 1.71494400048574 1.03401950009186e-25 1.83994646339874e-24 248.22312655583 265.590694471389 247.373304010064 72.828359342446 85.3717744366097 72.8515548157324 up 233.0 258.0 233.0 77.0 103.0 68.0

SERPINB5 -4.26250784350273 1.09331936908235e-25 1.94138680421961e-24 5.32667653553284 2.05884259280146 11.6785680004751 104.040513346351 130.129792102405 129.63291371623 down 5.0 2.0 11.0 110.0 157.0 121.0

PCDH10 -1.60293899841225 1.12935104268157e-25 2.00326771340585e-24 91.6188364111649 112.20692130768 91.3051680037147 281.855208883752 305.846454049602 309.619107966862 down 86.0 109.0 86.0 298.0 369.0 289.0

CACNA2D2 -2.32740043327904 1.14934679856297e-25 2.03660405519422e-24 36.2214004416233 54.5593287092388 32.912328001339 237.401534999402 168.256992336231 217.483318052848 down 34.0 53.0 31.0 251.0 203.0 203.0

AMTN -10.71382650241 1.19317439759171e-25 2.10985117904003e-24 0 1.02942129640073 1.06168800004319 1179.44109220818 1166.19501584767 1188.12315133305 down 0.0 1.0 1.0 1247.0 1407.0 1109.0

MAP7D2 1.44971642134937 1.38245212197218e-25 2.44199571910414e-24 339.841962966995 325.297129662631 333.370032013563 119.173678924002 132.616348639394 112.491371406646 up 319.0 316.0 314.0 126.0 160.0 105.0

DIRAS1 1.57679521782143 1.42776837321617e-25 2.51941627523769e-24 270.595168005068 292.355648177808 283.470696011533 93.6364620117162 92.0025918685794 98.5638682801085 up 254.0 284.0 267.0 99.0 111.0 92.0

TMCC3 -1.94743887684813 1.5212150408675e-25 2.67873001998914e-24 54.332100662435 53.5299074128381 59.4545280024189 203.351912449687 208.041896928049 234.624860362432 down 51.0 52.0 56.0 215.0 251.0 219.0

CPVL 4.20736835854817 1.58578176936e-25 2.78663310922805e-24 113.990877860403 98.8244444544703 119.970744004881 3.78329139441278 3.31540871598484 11.7848103378391 up 107.0 96.0 113.0 4.0 4.0 11.0

TMEM38A 2.14057669835113 1.69650850428118e-25 2.97811959197132e-24 185.368343436543 186.325254648533 191.103840007775 45.3994967329533 33.1540871598484 50.3532805344032 up 174.0 181.0 180.0 48.0 40.0 47.0

CDC42EP2 -1.21229059933833 1.72046333147184e-25 3.01410896823461e-24 191.760355279182 231.619791690165 220.831104008984 497.50281836528 477.418855101817 519.603001259268 down 180.0 225.0 208.0 526.0 576.0 485.0

CAMK2N1 -1.74763118582213 1.72056869674313e-25 3.01410896823461e-24 76.7041421116729 80.2948611192571 73.2564720029804 230.780775059179 268.548105994772 274.264676953345 down 72.0 78.0 69.0 244.0 324.0 256.0

CREB5 1.13452603310362 1.98314216796103e-25 3.45977634657671e-24 502.8382649543 512.651805607565 496.869984020215 224.160015118957 237.051723192916 227.125435601989 up 472.0 498.0 468.0 237.0 286.0 212.0

CDHR1 1.88675370104165 2.28851180048474e-25 3.97614255386784e-24 219.459073263953 276.914328731797 247.373304010064 72.828359342446 75.4255482886552 51.4246269287523 up 206.0 269.0 233.0 77.0 91.0 48.0

SDK1 1.17459148597226 3.24922407598398e-25 5.61651590277232e-24 519.883629868005 586.770138948417 551.016072022418 261.992929063085 216.330418718011 258.19448103811 up 488.0 570.0 519.0 277.0 261.0 241.0

FOXF2 -1.05227365139067 3.2540668879282e-25 5.6191532193174e-24 515.622288639579 637.211782472053 488.376480019869 1100.93779577412 1052.64226732519 1254.54662778269 down 484.0 619.0 460.0 1164.0 1270.0 1171.0

ZFYVE28 -4.18415396716885 3.54508338471303e-25 6.11544934185731e-24 5.32667653553284 10.2942129640073 3.18506400012958 110.661273286574 114.381600701477 118.91944977274 down 5.0 10.0 3.0 117.0 138.0 111.0

GSTM3 1.34188671480962 3.58735295041093e-25 6.18207110681192e-24 503.903600261407 510.592963014763 609.408912024794 179.706341234607 208.870749107045 253.909095460714 up 473.0 496.0 574.0 190.0 252.0 237.0

CHRNA7 3.7584802596952 4.82080589735216e-25 8.27400728481718e-24 106.533530710657 109.118657418478 100.860360004103 7.56658278882556 9.11737396895832 6.42807836609403 up 100.0 106.0 95.0 8.0 11.0 6.0

SLC12A7 -4.43643107695863 4.90759206373915e-25 8.4144341659657e-24 31.9600592131971 16.4707407424117 8.49350400034555 429.40357326585 396.191341560189 406.040283458273 down 30.0 16.0 8.0 454.0 478.0 379.0

STARD8 1.47117453134467 4.96371635866999e-25 8.50205815125073e-24 324.927268667503 313.973495402223 296.210952012051 110.661273286574 116.868157238466 109.277332223599 up 305.0 305.0 279.0 117.0 141.0 102.0

MRPL1 -1.14352300110863 5.14435049140007e-25 8.79367278751939e-24 243.961785327404 233.678634282966 220.831104008984 526.823326671979 525.492281483597 489.605302217495 down 229.0 227.0 208.0 557.0 634.0 457.0

SFXN2 1.60775567652393 6.31873089829358e-25 1.07577187353863e-23 284.444526997454 294.414490770609 295.149264012008 109.715450437971 98.633409300549 77.1369403931284 up 267.0 286.0 278.0 116.0 119.0 72.0

COL6A1 1.0256966132158 6.51252157663187e-25 1.10654077741368e-23 841.614892614189 1043.83319455034 993.73996804043 518.310921034551 444.264767941969 452.10817841528 up 790.0 1014.0 936.0 548.0 536.0 422.0

SORBS2 -2.60455007001293 7.16298491750956e-25 1.21584132768148e-23 24.5027120634511 24.7061111136176 21.2337600008639 143.765072987686 148.364540040322 136.060992082324 down 23.0 24.0 20.0 152.0 179.0 127.0

TSPAN10 1.96738767276593 7.28861218464397e-25 1.2359268309096e-23 201.348373043141 260.443587989385 214.460976008725 55.8035480675885 48.9022785607764 69.6375156326853 up 189.0 253.0 202.0 59.0 59.0 65.0

PKIA 1.26884795362553 1.19893227872215e-24 2.02491653056364e-23 425.068787535521 436.47462967391 410.873256016716 161.735707111146 195.609114243106 169.272730307143 up 399.0 424.0 387.0 171.0 236.0 158.0

WNT10A -3.5768866124076 1.45600296721286e-24 2.44919670295744e-23 11.7186883781723 8.23537037120586 8.49350400034555 110.661273286574 121.841270312443 106.063293040552 down 11.0 8.0 8.0 117.0 147.0 99.0

UGT8 1.54523770886485 1.75999752310189e-24 2.95191663775703e-23 279.117850461921 269.708379656992 282.40900801149 99.3113991033354 89.5160353315907 96.4211754914105 up 262.0 262.0 266.0 105.0 108.0 90.0

RAB7B 3.24023676141493 2.10914738319069e-24 3.53052931534094e-23 98.0108482538043 138.971875014099 115.723992004708 14.1873427290479 14.0904870429356 8.57077115479204 up 92.0 135.0 109.0 15.0 17.0 8.0

EMX2OS 5.1600720214768 2.75516324938776e-24 4.60280724306002e-23 115.056213167509 106.030393529275 113.600616004622 2.83746854580958 4.14426089498105 2.14269278869801 up 108.0 103.0 107.0 3.0 5.0 2.0

CLIC6 2.69175374773792 3.26884135160537e-24 5.43950613911542e-23 126.774901545682 146.177824088904 135.896064005529 24.5913940636831 24.8655653698863 12.8561567321881 up 119.0 142.0 128.0 26.0 30.0 12.0

CXCL2 -1.5729991865405 3.3298858893383e-24 5.53564935872334e-23 101.206854175124 159.560300942113 168.808392006868 379.274962289881 435.14739397301 464.964335147468 down 95.0 155.0 159.0 401.0 525.0 434.0

NME3 -1.46229934366798 4.24921248590033e-24 7.03633035299625e-23 112.925542553296 152.354351867308 164.561640006695 430.349396114453 393.7047850232 359.972388501266 down 106.0 148.0 155.0 455.0 475.0 336.0

GREB1L 1.03194032821648 4.30647442337098e-24 7.12418718085004e-23 640.266519571048 624.858726915244 664.61668802704 324.417237070896 284.2962973957 337.474114219937 up 601.0 607.0 626.0 343.0 343.0 315.0

ANKRD20A19P -6.77210557710299 4.93777135305651e-24 8.13675551758534e-23 1.06533530710657 3.0882638892022 1.06168800004319 216.593432330132 187.320592453144 170.344076701492 down 1.0 3.0 1.0 229.0 226.0 159.0

DSCAML1 -7.45208200201227 5.94842157254561e-24 9.76417262005065e-23 1.06533530710657 1.02942129640073 2.12337600008639 221.322546573147 255.286471130833 260.337173826808 down 1.0 1.0 2.0 234.0 308.0 243.0

BBS2 -1.00178971603164 6.58735000913174e-24 1.08024887855462e-22 325.99260397461 308.82638892022 341.863536013909 668.696753962458 668.054856270946 617.095523145027 down 306.0 300.0 322.0 707.0 806.0 576.0

BMI1 1.65102911956195 6.61456406525621e-24 1.08366262345687e-22 239.700444098978 264.561273174988 239.941488009762 72.828359342446 84.5429222576135 79.2796331818264 up 225.0 257.0 226.0 77.0 102.0 74.0

RBP7 3.82683077609874 6.6690768775644e-24 1.0915378000574e-22 99.0761835609109 106.030393529275 99.7986720040603 10.4040513346351 7.45966961096589 3.21403918304702 up 93.0 103.0 94.0 11.0 9.0 3.0

NXF3 5.35462106586273 6.9766439737488e-24 1.13638797033947e-22 101.206854175124 131.765925939294 114.662304004665 4.72911424301597 0 4.28538557739602 up 95.0 128.0 108.0 5.0 0.0 4.0

GRID1 -1.18898590016017 6.98879218652555e-24 1.13727319538658e-22 206.675049578674 264.561273174988 226.1395440092 526.823326671979 498.140159576723 567.813589004973 down 194.0 257.0 213.0 557.0 601.0 530.0

GABRB3 11.9089483518775 7.32478722334109e-24 1.1896634282205e-22 655.18121387054 651.623680621664 695.405640028292 0 0 0 up 615.0 633.0 655.0 0.0 0.0 0.0

QPCT -4.07589370680926 8.38290081566156e-24 1.35761319137005e-22 8.52268245685255 4.11768518560293 6.37012800025917 108.769627589367 107.750783269507 102.849253857505 down 8.0 4.0 6.0 115.0 130.0 96.0

ULBP1 2.29416147416032 8.64981699372249e-24 1.3995023865679e-22 197.087031814715 159.560300942113 170.931768006954 35.9412682469214 46.4157220237878 23.5696206756781 up 185.0 155.0 161.0 38.0 56.0 22.0

RIN1 1.03945237419033 1.07937433030182e-23 1.73642935947889e-22 783.021450723328 943.979328799471 829.178328033735 447.374207389311 363.03725440034 436.037982500045 up 735.0 917.0 781.0 473.0 438.0 407.0

SDSL 1.33089361931325 1.13686853357464e-23 1.82372660594265e-22 332.384615817249 373.679930593466 355.66548001447 134.306844501654 144.220279145341 143.560416842767 up 312.0 363.0 335.0 142.0 174.0 134.0

CECR7 5.12447880631587 1.43928532931063e-23 2.30014089420019e-22 106.533530710657 116.324606493283 102.98373600419 0 5.80196525297347 3.21403918304702 up 100.0 113.0 97.0 0.0 7.0 3.0

SERTAD4 -1.54204873820706 1.54425979454665e-23 2.46093705734903e-22 100.141518868017 91.6184953796652 106.168800004319 264.830397608894 289.269410469677 313.904493544259 down 94.0 89.0 100.0 280.0 349.0 293.0

PDE4D -1.76415528633079 1.82686308834378e-23 2.89766486109959e-22 78.8348127258861 86.4713888976615 69.0097200028076 257.263814820069 310.819567123579 226.05408920764 down 74.0 84.0 65.0 272.0 375.0 211.0

TRIM29 -2.38988174666496 2.18390225869877e-23 3.45750507124833e-22 30.8947239060905 30.882638892022 24.4188240009935 157.952415716733 158.310766188276 134.989645687975 down 29.0 30.0 23.0 167.0 191.0 126.0

TBC1D8B 1.78004974648242 2.23314616943311e-23 3.53216583661968e-22 270.595168005068 207.943101872948 222.954480009071 66.2075994022236 64.6504699617044 73.9229012100814 up 254.0 202.0 210.0 70.0 78.0 69.0

MMP7 -3.68729866496675 2.92484087348802e-23 4.61329649877905e-22 6.39201184263941 11.3236342604081 8.49350400034555 134.306844501654 117.697009417462 85.7077115479204 down 6.0 11.0 8.0 142.0 142.0 80.0

RTKN2 1.13993983770197 3.13002304483247e-23 4.93233398878716e-22 689.27194369795 615.593935247638 590.298528024016 284.692677429562 326.567758524507 245.338324305922 up 647.0 598.0 556.0 301.0 394.0 229.0

PLAT -1.20820222212631 3.44811705613656e-23 5.42350073639307e-22 218.393737956847 266.62011576779 200.659032008164 528.714972369186 490.680489965757 567.813589004973 down 205.0 259.0 189.0 559.0 592.0 530.0

GRK5 1.23148975942475 3.63737795234795e-23 5.70529467710872e-22 381.390039944152 412.797939856694 384.331056015636 170.248112748575 159.968470546269 172.48676949019 up 358.0 401.0 362.0 180.0 193.0 161.0

GPR153 -1.17363274383693 4.00828931440812e-23 6.28126003571448e-22 245.027120634511 318.091180587826 231.447984009416 628.972194321124 590.142751445302 574.241667371067 down 230.0 309.0 218.0 665.0 712.0 536.0

DNM1 1.13988171286066 4.23327982526899e-23 6.62158450970052e-22 514.556953332473 546.622708388789 480.944664019567 251.58887772845 208.870749107045 241.052938728526 up 483.0 531.0 453.0 266.0 252.0 225.0

KRT14 -11.7069488163437 4.32182308570672e-23 6.75384530183319e-22 0 0 0 667.750931113855 585.169638371325 689.947077960759 down 0.0 0.0 0.0 706.0 706.0 644.0

TFAP2C 3.46763572510548 4.87629889682898e-23 7.60630785564299e-22 107.598866017763 112.20692130768 91.3051680037147 4.72911424301597 11.6039305059469 11.7848103378391 up 101.0 109.0 86.0 5.0 14.0 11.0

COG8 -1.00338462865683 5.92753319645791e-23 9.2375724331184e-22 324.927268667503 415.886203745896 368.405736014988 733.958530516079 718.614839189715 773.512096719982 down 305.0 404.0 347.0 776.0 867.0 722.0

SOX9 2.70195296153753 6.61528611267502e-23 1.02810042888729e-21 135.297584002534 149.266087978106 149.69800800609 31.2121540039054 25.6944175488825 8.57077115479204 up 127.0 145.0 141.0 33.0 31.0 8.0

TRO -2.23939354355464 6.64391629554413e-23 1.03160350180126e-21 33.0253945203036 28.8237962992205 40.3441440016414 146.602541533495 163.283879262253 172.48676949019 down 31.0 28.0 38.0 155.0 197.0 161.0

GLDC 1.2927508645489 7.76598745569317e-23 1.20362147757953e-21 360.08333380202 352.06208336905 386.454432015723 154.169124322321 135.931757355379 159.630612758002 up 338.0 342.0 364.0 163.0 164.0 149.0

SLC43A2 1.56443940896579 8.70856110163009e-23 1.3484737208557e-21 292.967209454306 430.298101895506 350.357040014254 138.090135896066 113.552748522481 111.420025012297 up 275.0 418.0 330.0 146.0 137.0 104.0

MEIS1 -1.40361298770276 8.90531156638113e-23 1.37768016378535e-21 126.774901545682 117.354027789683 145.451256005918 361.30432816642 358.892993505359 308.547761572513 down 119.0 114.0 137.0 382.0 433.0 288.0

RTN4RL2 -2.23569740232009 8.96514545820855e-23 1.38567120494574e-21 37.2867357487299 62.7946990804447 32.912328001339 243.076472091021 168.256992336231 217.483318052848 down 35.0 61.0 31.0 257.0 203.0 203.0

TRHDE-AS1 -1.38203263526726 9.09426082549751e-23 1.40434620222359e-21 120.382889703042 136.913032421297 114.662304004665 331.983819859721 320.765793271533 317.118532727306 down 113.0 133.0 108.0 351.0 387.0 296.0

PEG10 -1.00330574304244 1.22709387827831e-22 1.88800820145636e-21 487.923570654808 362.356296333058 441.662208017969 865.427906471923 897.646909852896 823.865377254385 down 458.0 352.0 416.0 915.0 1083.0 769.0

ABCA7 1.00706035681015 1.38895579953573e-22 2.13510991326092e-21 633.874507728408 748.389282483332 638.07448802596 332.929642708324 348.946767357405 322.475264699051 up 595.0 727.0 601.0 352.0 421.0 301.0

MCC 1.35603275492203 1.42539888745358e-22 2.18716097404562e-21 345.168639502528 434.415787081109 403.441440016414 133.36102165305 170.743548873219 157.487919969304 up 324.0 422.0 380.0 141.0 206.0 147.0

DDAH1 1.08306407888603 1.49532090108448e-22 2.29237430446796e-21 509.23027679694 469.416111158734 476.697912019394 223.214192270354 231.249757939943 232.482167573734 up 478.0 456.0 449.0 236.0 279.0 217.0

PDE7B -1.36528376968749 1.55060574363725e-22 2.37283299884509e-21 144.885601766493 120.442291678886 158.191512006436 349.954453983182 346.460210820416 395.326819514783 down 136.0 117.0 149.0 370.0 418.0 369.0

PGM5P2 2.80227860786489 1.57323892684914e-22 2.40529489357621e-21 127.840236852788 132.795347235694 99.7986720040603 20.8081026692703 14.9193392219318 16.0701959152351 up 120.0 129.0 94.0 22.0 18.0 15.0

ETV5 -2.3134617086898 1.91765001292403e-22 2.92657578548946e-21 42.6134122842627 26.764953706419 25.4805120010367 158.898238565337 165.770435799242 145.703109631465 down 40.0 26.0 24.0 168.0 200.0 136.0

LOC728743 1.27555776422833 2.49530237813343e-22 3.78089644772632e-21 364.344675030446 421.0333102279 364.158984014816 147.548364382098 155.824209651288 172.48676949019 up 342.0 409.0 343.0 156.0 188.0 161.0

SPATA5 -1.37092425733249 2.51552576328134e-22 3.80813283556621e-21 166.192307908625 135.883611124897 125.279184005097 336.712934102737 370.496924011306 398.54085869783 down 156.0 132.0 118.0 356.0 447.0 372.0

C4orf46 -1.14428798262449 2.84961577979266e-22 4.3100438669364e-21 204.544378964461 256.325902803782 265.422000010799 521.14838958036 571.079151328389 512.103576498825 down 192.0 249.0 250.0 551.0 689.0 478.0

LRRN1 -8.18502417292754 3.30494230783311e-22 4.9765086839727e-21 1.06533530710657 0 2.12337600008639 321.579768525086 290.92711482767 306.405068783815 down 1.0 0.0 2.0 340.0 351.0 286.0

ZNF699 1.18383092654909 3.64206135733541e-22 5.47440278556005e-21 483.662229426382 459.121898194727 398.133000016198 206.189380995496 192.293705527121 191.771004588472 up 454.0 446.0 375.0 218.0 232.0 179.0

TMEM37 1.36037690336108 4.28206258860628e-22 6.42499027909569e-21 292.967209454306 336.620763923039 322.753152013131 127.686084561431 125.985531207424 116.776756984042 up 275.0 327.0 304.0 135.0 152.0 109.0

SGPP2 -3.08447517380687 4.62961340576337e-22 6.94032310563111e-21 18.1107002208117 16.4707407424117 8.49350400034555 126.740261712828 94.489148405568 145.703109631465 down 17.0 16.0 8.0 134.0 114.0 136.0

NEURL1B 1.01591919585911 5.36799744556261e-22 8.03302797949033e-21 535.863659474604 560.005185241998 526.597248021424 266.722043306101 255.286471130833 281.764101713788 up 503.0 544.0 496.0 282.0 308.0 263.0

DTX1 -4.00478484042823 5.69186252283032e-22 8.51016338364922e-21 7.45734714974598 8.23537037120586 4.24675200017278 141.873427290479 83.7140700786173 95.3498290970615 down 7.0 8.0 4.0 150.0 101.0 89.0

ZNF365 1.64614328770676 5.94937540038166e-22 8.87950830682514e-21 266.333826776642 246.031689839775 261.175248010626 84.1782335256843 98.633409300549 62.1380908722423 up 250.0 239.0 246.0 89.0 119.0 58.0

ZNF536 11.3824985237386 6.99870306332022e-22 1.0390712523457e-20 464.486193898464 487.945694493947 437.415456017796 0 0 0 up 436.0 474.0 412.0 0.0 0.0 0.0

PCDH11X -7.15339617280894 7.57865306184575e-22 1.12124351849491e-20 1.06533530710657 0 3.18506400012958 205.243558146893 229.59205358195 162.844651941049 down 1.0 0.0 3.0 217.0 277.0 152.0

BAAT 3.86516733877094 7.75655212742281e-22 1.14656189387908e-20 89.4881657969518 92.6479166760659 91.3051680037147 9.45822848603194 4.97311307397726 4.28538557739602 up 84.0 90.0 86.0 10.0 6.0 4.0

FLJ32255 -1.3237503143684 7.95961391660672e-22 1.17555239535587e-20 119.317554395936 147.207245385305 155.006448006306 361.30432816642 339.00054120945 355.68700292387 down 112.0 143.0 146.0 382.0 409.0 332.0

KLF14 1.93782115547753 9.30107424196629e-22 1.37008867529486e-20 168.322978522838 208.972523169349 185.795400007559 58.6410166133981 47.244574202784 40.7111629852622 up 158.0 203.0 175.0 62.0 57.0 38.0

GABRA5 6.03338635927013 1.51502280628538e-21 2.20864770554857e-20 101.206854175124 147.207245385305 118.909056004838 0.945822848603194 3.31540871598484 1.07134639434901 up 95.0 143.0 112.0 1.0 4.0 1.0

CD82 1.32882649218799 1.62681832585341e-21 2.36755175601003e-20 321.731262746184 342.797291701444 304.704456012397 135.252667350257 117.697009417462 133.918299293626 up 302.0 333.0 287.0 143.0 142.0 125.0

CNKSR2 2.71912858217799 1.9619196021626e-21 2.84545531341048e-20 112.925542553296 146.177824088904 135.896064005529 19.8622798206671 29.0098262648674 9.64211754914105 up 106.0 142.0 128.0 21.0 35.0 9.0

RBP4 -11.2013790748727 2.52603893218882e-21 3.64800507342528e-20 1.06533530710657 0 0 905.152466113257 847.086926934127 911.715781591003 down 1.0 0.0 0.0 957.0 1022.0 851.0

S1PR3 1.37063295209586 2.99633038952936e-21 4.30151159310401e-20 305.751233139585 282.061435213801 289.840824011792 121.065324621209 111.895044164488 106.063293040552 up 287.0 274.0 273.0 128.0 135.0 99.0

AP1AR -1.05953791618386 3.01844764820098e-21 4.32959383238989e-20 356.8873278807 284.120277806602 347.171976014125 607.218268803251 730.218769695661 721.016123396881 down 335.0 276.0 327.0 642.0 881.0 673.0

CLDN2 2.81318462548284 3.30814576229978e-21 4.73310719707417e-20 98.0108482538043 116.324606493283 108.292176004406 17.9706341234607 14.9193392219318 12.8561567321881 up 92.0 113.0 102.0 19.0 18.0 12.0

DISP2 1.40128188814075 3.48846019669174e-21 4.9868789647222e-20 320.665927439077 380.885879668271 329.12328001339 145.656718684892 106.093078911515 140.34637765972 up 301.0 370.0 310.0 154.0 128.0 131.0

SULT4A1 2.12473524441635 3.71320410944383e-21 5.29921462628293e-20 140.624260538067 171.913356498922 141.204504005745 35.9412682469214 34.8117915178408 33.2117382248192 up 132.0 167.0 133.0 38.0 42.0 31.0

RASSF4 1.5116642219835 3.78272929250501e-21 5.39389176894233e-20 301.489891911159 305.738125031017 324.876528013217 121.065324621209 125.156679028428 78.2082867874774 up 283.0 297.0 306.0 128.0 151.0 73.0

SERPINA1 1.75469894973683 4.21886768797202e-21 5.99560559012131e-20 258.876479626896 203.825416687345 190.042152007732 62.4243080078108 64.6504699617044 66.4234764496383 up 243.0 198.0 179.0 66.0 78.0 62.0

DPY19L2 2.37758626876864 4.31157485485789e-21 6.11709196325734e-20 183.23767282233 116.324606493283 164.561640006695 29.320508306699 36.4694958758333 22.4982742813291 up 172.0 113.0 155.0 31.0 44.0 21.0

ALOX5 -3.51186706323918 8.57009714482668e-21 1.19783370984624e-19 6.39201184263941 10.2942129640073 8.49350400034555 98.3655762547322 105.264226732519 83.5650187592224 down 6.0 10.0 8.0 104.0 127.0 78.0

PTGES -2.65055696980264 1.04869851272444e-20 1.46093361887763e-19 30.8947239060905 14.4118981496103 14.8636320006047 131.469375955844 124.327826849432 121.062142561438 down 29.0 14.0 14.0 139.0 150.0 113.0

SCN5A 2.40447267505103 1.22587482590123e-20 1.70076327197107e-19 136.362919309641 118.383449086084 129.52593600527 22.6997483664767 30.6675306228598 18.2128887039331 up 128.0 115.0 122.0 24.0 37.0 17.0

SH3GL2 2.65873006180652 1.61049974768886e-20 2.22527452902523e-19 120.382889703042 103.971550936474 122.094120004967 20.8081026692703 22.3790088328977 10.7134639434901 up 113.0 101.0 115.0 22.0 27.0 10.0

TNFSF10 -2.52782476564028 1.6206678043264e-20 2.23603921137988e-19 26.6333826776642 15.441319446011 24.4188240009935 112.55291898378 125.985531207424 144.631763237116 down 25.0 15.0 23.0 119.0 152.0 135.0

ESR1 -10.9252359203682 2.93103598466578e-20 4.00094678325852e-19 0 0 0 369.816733803849 378.785445801268 380.327969993897 down 0.0 0.0 0.0 391.0 457.0 355.0

MMP16 -1.39443949769231 3.54802068097947e-20 4.82758797877849e-19 128.905572159895 117.354027789683 93.4285440038011 289.421791672577 311.648419302575 291.406219262929 down 121.0 114.0 88.0 306.0 376.0 272.0

LINC00941 -1.55139176742781 3.57417011898182e-20 4.85926499322248e-19 86.2921598756321 68.9712268588491 93.4285440038011 241.184826393815 256.944175488825 229.268128390687 down 81.0 67.0 88.0 255.0 310.0 214.0

PPP1R14C -2.44083077940854 4.59901295622912e-20 6.21765997434328e-19 29.8293885989839 18.5295833352132 25.4805120010367 159.84406141394 119.354713775454 121.062142561438 down 28.0 18.0 24.0 169.0 144.0 113.0

ESRP2 -4.00438240052133 5.37254140688981e-20 7.25186067192936e-19 3.19600592131971 3.0882638892022 10.6168800004319 89.8531706173035 85.3717744366097 95.3498290970615 down 3.0 3.0 10.0 95.0 103.0 89.0

SMAD1 -1.04367968993563 5.62197526426524e-20 7.58250485482908e-19 214.13239672842 219.266736133356 236.756424009632 454.940790178137 447.580176657954 479.963184668354 down 201.0 213.0 223.0 481.0 540.0 448.0

NNT 1.2422186707733 6.24253221753745e-20 8.38608213838894e-19 330.253945203036 322.208865773429 318.506400012958 135.252667350257 147.535687861325 126.418874533183 up 310.0 313.0 300.0 143.0 178.0 118.0

PAIP2B 1.80537676087305 6.29607706029814e-20 8.44461958839672e-19 170.453649137051 180.148726870128 168.808392006868 48.2369652787629 54.7042438137499 44.9965485626582 up 160.0 175.0 159.0 51.0 66.0 42.0

SEL1L3 -1.26904709255671 6.32409408498722e-20 8.47548685124079e-19 153.408284223346 158.530879645713 121.032432004924 363.195973863627 348.117915178408 332.117382248192 down 144.0 154.0 114.0 384.0 420.0 310.0

TMEFF2 3.46994270953788 6.54010025803619e-20 8.75804730206585e-19 99.0761835609109 77.2065972300549 82.8116640033692 7.56658278882556 9.11737396895832 6.42807836609403 up 93.0 75.0 78.0 8.0 11.0 6.0

KIAA1324 1.84299073571606 7.31587930870638e-20 9.76603589357652e-19 162.996301987305 168.82509260972 180.486960007343 47.2911424301597 53.8753916347537 40.7111629852622 up 153.0 164.0 170.0 50.0 65.0 38.0

CDH1 -2.5503704717209 7.56423173381355e-20 1.00816747105273e-18 24.5027120634511 18.5295833352132 15.9253200006479 116.336210378193 115.210452880473 113.562717800995 down 23.0 18.0 15.0 123.0 139.0 106.0

KIRREL3 3.36046055113315 7.78312482929648e-20 1.03571197649868e-18 80.9654833400992 125.589398160889 80.6882880032828 6.62075994022236 12.4327826849432 8.57077115479204 up 76.0 122.0 76.0 7.0 15.0 8.0

SYTL4 1.70087834290614 8.09601543726581e-20 1.07565883535124e-18 190.695019972076 199.707731501742 190.042152007732 67.1534222508268 61.3350612457196 49.2819341400542 up 179.0 194.0 179.0 71.0 74.0 46.0

LYNX1 -3.01582542761293 8.1786556024108e-20 1.08578703687178e-18 8.52268245685255 13.3824768532095 18.0486960007343 133.36102165305 98.633409300549 91.0644435196654 down 8.0 13.0 17.0 141.0 119.0 85.0

DDX60 -1.51445665408569 9.44479140980487e-20 1.24898334490316e-18 74.5734714974598 98.8244444544703 93.4285440038011 238.347357848005 280.152036500719 243.195631517224 down 70.0 96.0 88.0 252.0 338.0 227.0

ANK3 3.38446941774507 9.50341837262039e-20 1.25575590664734e-18 117.186883781723 110.148078714878 71.133096002894 8.51240563742875 4.97311307397726 16.0701959152351 up 110.0 107.0 67.0 9.0 6.0 15.0

SIX1 -1.29085851367968 9.87207040705448e-20 1.30345185265396e-18 125.709566238575 171.913356498922 174.116832007084 345.225339740166 399.506750276173 410.325669035669 down 118.0 167.0 164.0 365.0 482.0 383.0

C1R -1.80615204610383 9.90820502403433e-20 1.30720399616154e-18 43.6787475913693 71.0300694516505 53.0844000021597 181.597986931813 200.582227317083 205.698507715009 down 41.0 69.0 50.0 192.0 242.0 192.0

GPRC5B -1.46339815211677 1.00740412855587e-19 1.32804871110789e-18 110.794871939083 81.3242824156578 104.045424004233 269.55951185191 251.971062414848 295.691604840325 down 104.0 79.0 98.0 285.0 304.0 276.0

ARL14EPL -2.60294835319113 1.01504161788693e-19 1.33603768508195e-18 17.0453649137051 19.5590046316139 18.0486960007343 116.336210378193 112.723896343485 102.849253857505 down 16.0 19.0 17.0 123.0 136.0 96.0

PTK2B 1.75572511784238 1.25979892822816e-19 1.65050223079544e-18 185.368343436543 193.531203723338 246.311616010021 73.7741821910492 54.7042438137499 56.7813589004973 up 174.0 188.0 232.0 78.0 66.0 53.0

CD163L1 3.17130591207342 1.27707375133629e-19 1.67184152609249e-18 85.2268245685255 103.971550936474 81.749976003326 12.2956970318415 8.28852178996211 9.64211754914105 up 80.0 101.0 77.0 13.0 10.0 9.0

MAP3K5 -1.24036091604106 1.34588817256053e-19 1.76056723113323e-18 158.734960758879 152.354351867308 199.597344008121 392.516482170326 445.093620120965 366.40046686736 down 149.0 148.0 188.0 415.0 537.0 342.0

TRPC6 -6.1466764358584 1.43859904256516e-19 1.87749366572063e-18 2.13067061421314 2.05884259280146 1.06168800004319 112.55291898378 133.44520081839 126.418874533183 down 2.0 2.0 1.0 119.0 161.0 118.0

GALNT6 2.67853401490224 1.57813178137169e-19 2.05484645476068e-18 109.729536631977 111.177500011279 92.3668560037579 19.8622798206671 13.2616348639394 16.0701959152351 up 103.0 108.0 87.0 21.0 16.0 15.0

ROR1 1.15351550014631 1.73736081369345e-19 2.25697025950667e-18 365.410010337553 371.621088000664 355.66548001447 174.031404142988 150.022244398314 168.201383912794 up 343.0 361.0 335.0 184.0 181.0 157.0

CES3 1.82890914293555 1.7476942803401e-19 2.26865449110814e-18 176.84566097969 189.413518537735 200.659032008164 69.9908907966364 45.5868698447916 43.9252021683092 up 166.0 184.0 189.0 74.0 55.0 41.0

TNFAIP3 -1.0804719571484 2.10239913474695e-19 2.71453059013822e-18 213.067061421314 202.795995390944 220.831104008984 489.936235576455 413.597237319109 443.537407260488 down 200.0 197.0 208.0 518.0 499.0 414.0

SLC16A13 3.55952325991572 2.69425244671289e-19 3.47077083249554e-18 89.4881657969518 78.2360185264557 90.2434800036715 4.72911424301597 4.14426089498105 13.9275031265371 up 84.0 76.0 85.0 5.0 5.0 13.0

NME4 -2.07566024044183 3.44276636817397e-19 4.40819820686825e-18 35.1560651345168 36.0297453740256 28.6655760011662 143.765072987686 135.102905176382 142.489070448418 down 33.0 35.0 27.0 152.0 163.0 133.0

CLEC2L 2.33829973540418 3.51031137373209e-19 4.49128962772066e-18 109.729536631977 126.61881945729 138.019440005615 32.1579768525086 22.3790088328977 19.2842350982821 up 103.0 123.0 130.0 34.0 27.0 18.0

ITM2A 5.70018550607651 3.79488946735197e-19 4.85173038316546e-18 89.4881657969518 111.177500011279 91.3051680037147 1.89164569720639 2.48655653698863 1.07134639434901 up 84.0 108.0 86.0 2.0 3.0 1.0

PPIP5K1 1.06035172474365 3.87576732985571e-19 4.94766379561082e-18 396.304734243644 453.974791712723 424.675200017278 208.081026692703 203.897636033068 199.270429348915 up 372.0 441.0 400.0 220.0 246.0 186.0

HAVCR1 1.46987663347989 3.95765907901479e-19 5.04080787958725e-18 257.81114431979 216.178472244154 246.311616010021 88.9073477687003 92.0025918685794 78.2082867874774 up 242.0 210.0 232.0 94.0 111.0 73.0

ACTBL2 1.70426181615466 6.21482689680212e-19 7.863876829078e-18 188.564349357863 188.384097241334 164.561640006695 51.0744338245725 56.3619481717423 58.9240516891953 up 177.0 183.0 155.0 54.0 68.0 55.0

PXMP4 1.12445852076032 6.31778566141991e-19 7.98681261973532e-18 368.606016258873 347.944398183448 386.454432015723 165.518998505559 163.283879262253 177.843501461935 up 346.0 338.0 364.0 175.0 197.0 166.0

GAS1 3.47458760825745 6.66364091792608e-19 8.41775370243608e-18 70.3121302690335 114.265763900481 76.44153600311 9.45822848603194 6.63081743196968 7.49942476044304 up 66.0 111.0 72.0 10.0 8.0 7.0

PPP1R3F 1.69203798318026 6.96879030404792e-19 8.79011971337094e-18 259.941814934003 338.679606515841 212.337600008639 99.3113991033354 67.9658786776893 84.6363651535714 up 244.0 329.0 200.0 105.0 82.0 79.0

SGCG 2.15525573913669 7.67798371522672e-19 9.66307905913378e-18 142.75493115228 111.177500011279 143.327880005831 27.4288626094926 30.6675306228598 31.0690454361212 up 134.0 108.0 135.0 29.0 37.0 29.0

HAPLN3 -1.60217805409193 8.78513940675016e-19 1.10237230778035e-17 68.1814596548204 63.8241203768454 79.6266000032396 224.160015118957 188.978296811136 230.339474785036 down 64.0 62.0 75.0 237.0 228.0 215.0

HOXC8 1.85297411689938 9.03043398847742e-19 1.13231348456556e-17 158.734960758879 161.619143534915 166.685016006782 54.8577252189853 43.1003133078029 36.4257774078662 up 149.0 157.0 157.0 58.0 52.0 34.0

PCYOX1L 1.43698336741518 9.05239277725014e-19 1.13422731987143e-17 248.22312655583 289.267384288606 260.113560010583 117.282033226796 83.7140700786173 94.2784827027125 up 233.0 281.0 245.0 124.0 101.0 88.0

FAM46A -1.62173833141651 1.21942867026809e-18 1.51445173565553e-17 75.6388068045664 66.9123842660476 56.2694640022893 205.243558146893 188.978296811136 218.554664447197 down 71.0 65.0 53.0 217.0 228.0 204.0

HMGA2 -1.83001268908984 1.23304245860324e-18 1.53023730759991e-17 36.2214004416233 52.5004861164373 55.2077760022461 164.573175656956 169.914696694223 177.843501461935 down 34.0 51.0 52.0 174.0 205.0 166.0

UGT1A6 -1.15814111479086 1.40122112170356e-18 1.73513785099841e-17 241.831114713191 179.119305573727 227.201232009244 531.552440914995 492.338194323749 419.96778658481 down 227.0 174.0 214.0 562.0 594.0 392.0

EXOSC1 1.01887122782705 1.45285113618094e-18 1.79513481013167e-17 430.395464071054 410.739097263892 456.525840018574 209.026849541306 213.015010002026 218.554664447197 up 404.0 399.0 430.0 221.0 257.0 204.0

BTG1 -1.03539074662542 1.65625693348203e-18 2.03458973554646e-17 210.936390807101 214.119629651352 260.113560010583 480.478007090423 451.724437552935 472.463759907911 down 198.0 208.0 245.0 508.0 545.0 441.0

FRY 6.01439892032779 1.70863187553108e-18 2.09721262598683e-17 100.141518868017 106.030393529275 97.6752960039739 1.89164569720639 0.82885217899621 2.14269278869801 up 94.0 103.0 92.0 2.0 1.0 2.0

NOL4L 1.05139980847034 1.7786188225201e-18 2.18015939605575e-17 428.264793456841 393.23893522508 390.701184015896 190.110392569242 201.411079496079 192.842350982821 up 402.0 382.0 368.0 201.0 243.0 180.0

HCN2 -1.6209757740635 2.34308720057051e-18 2.85758798975267e-17 517.752959253792 807.066296378174 652.938120026565 2066.62292419798 1917.13509001823 2100.9102793184 down 486.0 784.0 615.0 2185.0 2313.0 1961.0

STC1 -1.77919108693154 2.90465993072297e-18 3.52470911364235e-17 52.2014300482219 54.5593287092388 54.1460880022029 209.026849541306 193.122557706117 148.917148814512 down 49.0 53.0 51.0 221.0 233.0 139.0

IL20RB -2.22299145617909 3.04275633042786e-18 3.68173515981771e-17 21.3067061421314 37.0591666704264 29.7272640012094 151.331655776511 112.723896343485 148.917148814512 down 20.0 36.0 28.0 160.0 136.0 139.0

SAA1 -3.28601582176948 3.47272118440015e-18 4.19002114414093e-17 5.32667653553284 9.26479166760659 12.7402560005183 93.6364620117162 72.1101395726703 101.777907463156 down 5.0 9.0 12.0 99.0 87.0 95.0

JPH1 2.29009623435315 3.71313847051246e-18 4.47690859006982e-17 122.513560317255 106.030393529275 112.538928004579 20.8081026692703 21.5501566539015 27.8550062530741 up 115.0 103.0 106.0 22.0 26.0 26.0

ZDHHC1 1.403496946578 3.83648152023e-18 4.61904740246597e-17 276.987179847708 295.44391206701 238.879800009719 114.444564680987 85.3717744366097 108.20598582925 up 260.0 287.0 225.0 121.0 103.0 101.0

RGS2 1.87607188060845 3.95910944484133e-18 4.7599229237482e-17 155.538954837559 165.736828720518 146.512944005961 32.1579768525086 48.0734263817802 47.1392413513562 up 146.0 161.0 138.0 34.0 58.0 44.0

SPTLC3 -1.37762543941383 3.97807026938503e-18 4.77932697612642e-17 162.996301987305 108.089236122077 130.587624005313 296.988374461403 394.533637202196 350.330270952125 down 153.0 105.0 123.0 314.0 476.0 327.0

MOSPD1 1.17627579796296 5.39863444852795e-18 6.44488143467677e-17 640.266519571048 446.768842637918 573.311520023325 243.076472091021 213.015010002026 280.692755319439 up 601.0 434.0 540.0 257.0 257.0 262.0

TECPR1 1.05882652376314 5.78194277545848e-18 6.89275936778795e-17 494.315582497448 628.976412100847 495.808296020172 257.263814820069 282.638593037708 235.696206756781 up 464.0 611.0 467.0 272.0 341.0 220.0

S100A3 1.04681352845678 6.54949587024939e-18 7.79132444115342e-17 411.219428543135 493.092800975951 496.869984020215 247.805586334037 217.159270897007 213.197932475452 up 386.0 479.0 468.0 262.0 262.0 199.0

CATSPER1 1.16306662508412 6.66089341153586e-18 7.9182831151872e-17 327.057939281717 320.150023180628 325.938216013261 148.494187230702 154.995357472291 129.63291371623 up 307.0 311.0 307.0 157.0 187.0 121.0

TBC1D19 -1.18709369769892 7.10981203802416e-18 8.44009922383527e-17 144.885601766493 130.736504642893 147.574632006004 331.037997011118 333.198575956477 297.834297629023 down 136.0 127.0 139.0 350.0 402.0 278.0

EFNA2 -1.55120270392272 9.26271018939894e-18 1.09345164187051e-16 66.0507890406073 77.2065972300549 65.8246560026781 224.160015118957 188.978296811136 200.341775743264 down 62.0 75.0 62.0 237.0 228.0 187.0

SLC52A3 1.62845058730576 1.11835491193305e-17 1.31288511490963e-16 198.152367121822 188.384097241334 174.116832007084 65.2617765536204 49.7311307397726 67.4948228439873 up 186.0 183.0 164.0 69.0 60.0 63.0

ZNF788 1.28111790547476 1.17809458415722e-17 1.38110188620231e-16 344.103304195422 285.149699103003 306.827832012483 113.498741832383 150.022244398314 119.990796167089 up 323.0 277.0 289.0 120.0 181.0 112.0

KCTD4 -2.50831091681335 1.30688739019125e-17 1.52891383907733e-16 18.1107002208117 21.6178472244154 16.9870080006911 128.631907410034 101.948818016534 92.1357899140145 down 17.0 21.0 16.0 136.0 123.0 86.0

LOC100506085 -5.43592714602504 1.3541838916291e-17 1.58206035339289e-16 3.19600592131971 1.02942129640073 2.12337600008639 88.9073477687003 95.3180005845642 88.9217507309674 down 3.0 1.0 2.0 94.0 115.0 83.0

ING2 -1.237069399713 1.47847739555095e-17 1.72251768092387e-16 121.448225010149 112.20692130768 129.52593600527 279.017740337942 297.55793225964 278.550062530741 down 114.0 109.0 122.0 295.0 359.0 260.0

CALHM3 -4.51358620867228 1.48721220112143e-17 1.73150341491388e-16 2.13067061421314 2.05884259280146 6.37012800025917 95.5281077089226 67.9658786776893 77.1369403931284 down 2.0 2.0 6.0 101.0 82.0 72.0

WDR19 -1.02041155702104 1.59713524886129e-17 1.85565645512416e-16 242.896450020298 201.766574094544 203.844096008293 434.132687508866 456.697550626912 423.181825767857 down 228.0 196.0 192.0 459.0 551.0 395.0

BDH2 -1.13299613763088 1.70704464746746e-17 1.98063947452731e-16 174.714990365477 157.501458349312 146.512944005961 337.65875695134 337.342836851458 376.042584416501 down 164.0 153.0 138.0 357.0 407.0 351.0

LRRC37A3 1.00716041521423 1.9501669260395e-17 2.25500530560472e-16 387.782051786791 428.239259302705 426.798576017364 212.810140935719 208.870749107045 196.056390165868 up 364.0 416.0 402.0 225.0 252.0 183.0

CFD -1.06960125859748 2.12058566402011e-17 2.44705184935291e-16 251.41913247715 213.090208354952 182.610336007429 469.128132907184 426.030020004052 463.892988753119 down 236.0 207.0 172.0 496.0 514.0 433.0

SEMA5A 4.40743118685586 2.32594677235688e-17 2.67673494047048e-16 61.789447812181 71.0300694516505 84.9350400034556 0.945822848603194 5.80196525297347 3.21403918304702 up 58.0 69.0 80.0 1.0 7.0 3.0

SLC13A3 1.40113680261062 2.32986554932415e-17 2.67942446745085e-16 246.092455941617 286.179120399404 235.694736009589 86.0698792228907 89.5160353315907 116.776756984042 up 231.0 278.0 222.0 91.0 108.0 109.0

ITGA11 -4.65611744550336 2.37711457164886e-17 2.73190779129794e-16 3.19600592131971 4.11768518560293 2.12337600008639 89.8531706173035 64.6504699617044 84.6363651535714 down 3.0 4.0 2.0 95.0 78.0 79.0

CCDC186 1.10239889411305 2.6635318933713e-17 3.05072550870249e-16 826.700198314697 625.888148211645 588.17515202393 319.68812282788 358.064141326363 269.979291375949 up 776.0 608.0 554.0 338.0 432.0 252.0

PLD1 1.10005430948193 2.66980288489798e-17 3.05584195068728e-16 355.821992573594 336.620763923039 323.814840013174 160.789884262543 161.626174904261 151.05984160321 up 334.0 327.0 305.0 170.0 195.0 141.0

CRLF1 -2.99953650843812 2.87692399813859e-17 3.28846778194789e-16 10.6533530710657 11.3236342604081 10.6168800004319 97.419753406129 92.0025918685794 70.7088620270343 down 10.0 11.0 10.0 103.0 111.0 66.0

ATP2A3 1.40829026663045 2.97687608828599e-17 3.39813213851514e-16 221.589743878166 289.267384288606 255.86680801041 92.690639163113 110.237339806496 84.6363651535714 up 208.0 281.0 241.0 98.0 133.0 79.0

SFMBT2 9.99445928179322 3.62422436145142e-17 4.11490353103131e-16 359.017998494914 371.621088000664 303.642768012354 0 0.82885217899621 0 up 337.0 361.0 286.0 0.0 1.0 0.0

CDSN 1.34734902592344 3.66213724657141e-17 4.15516443113996e-16 231.177761642125 261.473009285786 228.262920009287 91.7448163145099 101.948818016534 88.9217507309674 up 217.0 254.0 215.0 97.0 123.0 83.0

TIMP3 -7.20477803990151 3.79710703386188e-17 4.29966531775537e-16 1.06533530710657 1.02942129640073 1.06168800004319 140.927604441876 147.535687861325 177.843501461935 down 1.0 1.0 1.0 149.0 178.0 166.0

CD200 -3.25984002820473 4.69744764150297e-17 5.27533930118628e-16 8.52268245685255 12.3530555568088 4.24675200017278 77.5574735854619 84.5429222576135 79.2796331818264 down 8.0 12.0 4.0 82.0 102.0 74.0

MAP3K8 -1.48441850149352 4.74472374652689e-17 5.31584790120142e-16 121.448225010149 78.2360185264557 106.168800004319 233.618243604989 329.054315061496 291.406219262929 down 114.0 76.0 100.0 247.0 397.0 272.0

AADAT -1.24769065472458 4.91952486860368e-17 5.50440893488416e-16 144.885601766493 127.648240753691 153.944760006263 282.801031732355 348.946767357405 381.399316388246 down 136.0 124.0 145.0 299.0 421.0 356.0

ANKRD36BP2 4.00566324228356 5.18617097439314e-17 5.7989264888594e-16 66.0507890406073 73.088912044452 70.0714080028508 1.89164569720639 7.45966961096589 3.21403918304702 up 62.0 71.0 66.0 2.0 9.0 3.0

OAS2 -1.92510535575005 5.29069184819994e-17 5.91189445306775e-16 42.6134122842627 38.0885879668271 30.7889520012526 140.927604441876 154.166505293295 127.490220927532 down 40.0 37.0 29.0 149.0 186.0 119.0

PSG2 3.89824603345759 5.80128100133031e-17 6.47389329133962e-16 68.1814596548204 59.7064351912425 81.749976003326 2.83746854580958 5.80196525297347 5.35673197174503 up 64.0 58.0 77.0 3.0 7.0 5.0

PLEKHH2 1.79803688725486 6.54278439668136e-17 7.26785361834638e-16 147.016272380706 167.795671313319 147.574632006004 34.049622549715 50.5599829187688 48.2105877457052 up 138.0 163.0 139.0 36.0 61.0 45.0

PNPLA4 -9.94708294529609 6.57103496247505e-17 7.29445165559157e-16 0 0 0 181.597986931813 196.437966422102 194.985043771519 down 0.0 0.0 0.0 192.0 237.0 182.0

ZNF721 -1.19057784825966 7.1765115255882e-17 7.95615872012199e-16 191.760355279182 171.913356498922 170.931768006954 349.008631134579 488.193933428768 380.327969993897 down 180.0 167.0 161.0 369.0 589.0 355.0

MCOLN2 2.2566514412566 7.37395076847283e-17 8.16436117764246e-16 119.317554395936 98.8244444544703 119.970744004881 18.9164569720639 30.6675306228598 20.3555814926311 up 112.0 96.0 113.0 20.0 37.0 19.0

ARRDC4 -1.20328838939557 7.44835536333491e-17 8.23597518635074e-16 162.996301987305 123.530555568088 147.574632006004 318.742299979277 368.839219653314 309.619107966862 down 153.0 120.0 139.0 337.0 445.0 289.0

RWDD4 -1.00856079197948 7.46142675637525e-17 8.2450469180037e-16 189.629684664969 207.943101872948 225.077856009157 439.807624600485 397.849045918181 415.682401007414 down 178.0 202.0 212.0 465.0 480.0 388.0

AIM1 -1.20002266252007 8.6015504474468e-17 9.48634535024406e-16 149.14694299492 127.648240753691 118.909056004838 323.471414222292 290.92711482767 294.620258445976 down 140.0 124.0 112.0 342.0 351.0 275.0

ACTC1 -4.12095375850928 8.9922358383079e-17 9.91076610936472e-16 6.39201184263941 4.11768518560293 2.12337600008639 79.4491192826683 65.4793221407006 74.9942476044304 down 6.0 4.0 2.0 84.0 79.0 70.0

ENPP2 -3.79218226801014 9.25401198007216e-17 1.0182549619804e-15 5.32667653553284 7.20594907480513 3.18506400012958 70.9367136452396 74.596696109659 72.8515548157324 down 5.0 7.0 3.0 75.0 90.0 68.0

DUSP6 -2.4914010743253 9.25686329073092e-17 1.0182549619804e-15 17.0453649137051 18.5295833352132 15.9253200006479 90.7989934659067 95.3180005845642 103.920600251854 down 16.0 18.0 15.0 96.0 115.0 97.0

HSD17B8 -1.61287763535325 1.10804434180255e-16 1.2133336231503e-15 62.8547831192875 54.5593287092388 56.2694640022893 191.056215417845 180.689775021174 158.559266363653 down 59.0 53.0 53.0 202.0 218.0 148.0

LYRM7 1.02132564201378 1.12242753438887e-16 1.22828956282606e-15 454.898176134505 411.768518560293 402.379752016371 200.514443903877 232.907462297935 189.628311799774 up 427.0 400.0 379.0 212.0 281.0 177.0

GGT8P 3.70365900942109 1.28597562670536e-16 1.40454075540869e-15 79.9001480329926 71.0300694516505 58.3928400023757 3.78329139441278 4.14426089498105 8.57077115479204 up 75.0 69.0 55.0 4.0 5.0 8.0

EPCAM -2.07023834895802 1.40992135433898e-16 1.53595290948568e-15 22.3720414492379 30.882638892022 35.0357040014254 121.065324621209 132.616348639394 116.776756984042 down 21.0 30.0 33.0 128.0 160.0 109.0

ASXL3 5.74771806976758 1.45475699897306e-16 1.58276066554937e-15 84.1614892614189 86.4713888976615 81.749976003326 4.72911424301597 0 0 up 79.0 84.0 77.0 5.0 0.0 0.0

RGL1 1.7130866175735 1.51702069338782e-16 1.64838553854969e-15 182.172337515223 188.384097241334 141.204504005745 43.5078510357469 53.0465394557575 59.9953980835443 up 171.0 183.0 133.0 46.0 64.0 56.0

ADAMTSL5 1.03807805196598 1.82663013358707e-16 1.97719581233003e-15 457.028846748718 615.593935247638 510.671928020776 281.855208883752 231.249757939943 259.265827432459 up 429.0 598.0 481.0 298.0 279.0 242.0

MLF1 1.1390713318318 1.82865273802131e-16 1.97812116105242e-15 310.012574368011 305.738125031017 360.973920014686 135.252667350257 155.824209651288 152.131187997559 up 291.0 297.0 340.0 143.0 188.0 142.0

RAB9B 1.45951774782096 1.85532198042321e-16 2.00568949255706e-15 263.137820855322 226.472685208161 274.977192011187 68.09924509943 113.552748522481 95.3498290970615 up 247.0 220.0 259.0 72.0 137.0 89.0

KCNQ3 -3.78179487078752 1.98406082778596e-16 2.14212813401492e-15 7.45734714974598 2.05884259280146 6.37012800025917 65.2617765536204 72.9389917516665 79.2796331818264 down 7.0 2.0 6.0 69.0 88.0 74.0

SPNS2 2.49596053818251 2.03494772274228e-16 2.19287623557596e-15 93.749507025378 103.971550936474 82.8116640033692 12.2956970318415 18.2347479379166 19.2842350982821 up 88.0 101.0 78.0 13.0 22.0 18.0

SPATC1L 1.83878739604595 2.06000362527858e-16 2.21846544260771e-15 139.55892523096 178.089884277327 145.451256005918 31.2121540039054 48.0734263817802 50.3532805344032 up 131.0 173.0 137.0 33.0 58.0 47.0

GDF15 -2.08196813265589 2.28234277804113e-16 2.45322884898583e-15 36.2214004416233 24.7061111136176 26.5422000010799 125.794438864225 135.102905176382 108.20598582925 down 34.0 24.0 25.0 133.0 163.0 101.0

POTEF 2.77786821034518 2.40148204890008e-16 2.57801685097385e-15 92.6841717182715 94.7067592688674 69.0097200028076 16.0789884262543 12.4327826849432 8.57077115479204 up 87.0 92.0 65.0 17.0 15.0 8.0

SHROOM4 1.82980557291898 2.41553371687835e-16 2.59145922507405e-15 167.257643215731 149.266087978106 164.561640006695 29.320508306699 57.1908003507385 48.2105877457052 up 157.0 145.0 155.0 31.0 69.0 45.0

SLC24A3 -2.15745073807553 2.42716042142629e-16 2.60228465436464e-15 23.4373767563445 28.8237962992205 23.3571360009503 122.956970318415 108.579635448504 106.063293040552 down 22.0 28.0 22.0 130.0 131.0 99.0

FAM184A 2.3390958877049 2.81191458696662e-16 3.00718643328374e-15 143.820266459387 88.530231490463 141.204504005745 34.049622549715 23.2078610118939 16.0701959152351 up 135.0 86.0 133.0 36.0 28.0 15.0

NOVA2 -3.82968192805463 3.30156785429889e-16 3.50649275560021e-15 5.32667653553284 4.11768518560293 5.30844000021597 72.828359342446 72.1101395726703 64.2807836609403 down 5.0 4.0 5.0 77.0 87.0 60.0

CXADR 2.57389549884291 3.40031187284493e-16 3.60910295275647e-15 87.3574951827386 81.3242824156578 88.1201040035851 16.0789884262543 14.0904870429356 12.8561567321881 up 82.0 79.0 83.0 17.0 17.0 12.0

FAM149A -1.08946878330814 5.07851491947376e-16 5.3468019102477e-15 141.689595845174 156.472037052911 146.512944005961 323.471414222292 313.306123660568 309.619107966862 down 133.0 152.0 138.0 342.0 378.0 289.0

RELL2 1.4317262422246 5.20915272421918e-16 5.47753241143841e-15 188.564349357863 184.266412055731 210.214224008552 70.9367136452396 70.4524352146779 74.9942476044304 up 177.0 179.0 198.0 75.0 85.0 70.0

BCAN 1.21046139374031 5.97213684902511e-16 6.26039592960924e-15 255.680473705576 331.473657441036 294.087576011965 137.144313047463 122.670122491439 121.062142561438 up 240.0 322.0 277.0 145.0 148.0 113.0

KCNS3 1.0102781487919 6.94964738942473e-16 7.25366770036074e-15 413.350099157349 377.797615779069 460.772592018746 193.893683963655 200.582227317083 228.196781996338 up 388.0 367.0 434.0 205.0 242.0 213.0

ENO2 1.06289840250517 7.8797588594671e-16 8.1891481643787e-15 422.938116921308 472.504375047936 431.045328017537 258.209637668672 187.320592453144 189.628311799774 up 397.0 459.0 406.0 273.0 226.0 177.0

SFRP4 2.50896450717698 8.29750067287192e-16 8.61272435039524e-15 120.382889703042 119.412870382485 71.133096002894 23.6455712150799 14.0904870429356 17.1415423095841 up 113.0 116.0 67.0 25.0 17.0 16.0

GPR15 -2.43246150903721 8.4072791557895e-16 8.72132938757343e-15 18.1107002208117 19.5590046316139 16.9870080006911 78.5032964340651 104.435374553523 112.491371406646 down 17.0 19.0 16.0 83.0 126.0 105.0

KCNA7 1.72629322058393 1.00288416887168e-15 1.03590596467599e-14 190.695019972076 158.530879645713 130.587624005313 46.3453195815565 45.5868698447916 53.5673197174503 up 179.0 154.0 123.0 49.0 55.0 50.0

PTPN6 -3.91748993767072 1.01851193736704e-15 1.05140720408274e-14 7.45734714974598 3.0882638892022 3.18506400012958 80.3949421312715 62.1639134247158 64.2807836609403 down 7.0 3.0 3.0 85.0 75.0 60.0

HCP5 -3.07091998272704 1.03385660189628e-15 1.06659749306474e-14 5.32667653553284 11.3236342604081 11.6785680004751 69.0450679480332 96.9757049425566 71.7802084213833 down 5.0 11.0 11.0 73.0 117.0 67.0

PRDM1 2.0701941565461 1.07110368139215e-15 1.10300889743362e-14 110.794871939083 113.236342604081 105.107112004276 26.4830397608894 21.5501566539015 31.0690454361212 up 104.0 110.0 99.0 28.0 26.0 29.0

LOXL4 -1.87471310419117 1.23599712736147e-15 1.26818845169614e-14 30.8947239060905 33.9709027812242 40.3441440016414 125.794438864225 124.327826849432 136.060992082324 down 29.0 33.0 38.0 133.0 150.0 127.0

MME 2.34337428793384 1.29915466271793e-15 1.33137809960325e-14 87.3574951827386 113.236342604081 93.4285440038011 24.5913940636831 19.0636001169128 13.9275031265371 up 82.0 110.0 88.0 26.0 23.0 13.0

EMX2 2.55379041553415 1.39728145217274e-15 1.42934467390134e-14 76.7041421116729 87.5008101940622 83.8733520034124 13.2415198804447 14.0904870429356 14.9988495208861 up 72.0 85.0 79.0 14.0 17.0 14.0

ALDH2 -9.54350328666481 1.40285317497046e-15 1.43417820060347e-14 0 0 0 155.114947170924 127.643235565416 151.05984160321 down 0.0 0.0 0.0 164.0 154.0 141.0

GATA4 8.26463948668206 1.46035434942449e-15 1.49116351291446e-14 192.825690586289 198.678310205341 184.733712007516 0 1.65770435799242 0 up 181.0 193.0 174.0 0.0 2.0 0.0

TRAPPC6A 1.02679771818396 1.5448282969893e-15 1.5745722834536e-14 363.27933972334 403.533148189087 379.02261601542 160.789884262543 202.239931675075 199.270429348915 up 341.0 392.0 357.0 170.0 244.0 186.0

CEACAM19 1.18833199024617 1.56769803940389e-15 1.59692151458219e-14 270.595168005068 317.061759291426 290.902512011835 130.523553107241 110.237339806496 146.774456025814 up 254.0 308.0 274.0 138.0 133.0 137.0

PCDHB6 1.09683038713897 1.5751666492429e-15 1.60356508642877e-14 300.424556604052 380.885879668271 385.39274401568 157.00659286813 172.401253231212 169.272730307143 up 282.0 370.0 363.0 166.0 208.0 158.0

FRMD5 1.6128600870791 1.66951318403073e-15 1.69859179204088e-14 143.820266459387 152.354351867308 170.931768006954 52.0202566731757 52.2176872767613 48.2105877457052 up 135.0 148.0 161.0 55.0 63.0 45.0

IFITM1 -5.52568442228596 1.71032557709871e-15 1.73907054478105e-14 3.19600592131971 1.02942129640073 1.06168800004319 80.3949421312715 79.5698091836362 82.4936723648734 down 3.0 1.0 1.0 85.0 96.0 77.0

PCBP3 9.45729877865032 1.82872163411542e-15 1.85722688740499e-14 209.871055499994 248.090532432576 254.805120010367 0 0 1.07134639434901 up 197.0 241.0 240.0 0.0 0.0 1.0

PLEKHG1 -1.87635479197856 1.83232448818627e-15 1.85977093048984e-14 30.8947239060905 38.0885879668271 37.1590800015118 138.090135896066 114.381600701477 138.203684871022 down 29.0 37.0 35.0 146.0 138.0 129.0

IRF7 -1.00054663756642 1.99695709565105e-15 2.02323284690962e-14 263.137820855322 250.149375025378 191.103840007775 501.286109759693 468.301481132859 439.252021683092 down 247.0 243.0 180.0 530.0 565.0 410.0

TRIM47 -1.16128223612679 2.53982529888653e-15 2.56463991573395e-14 127.840236852788 136.913032421297 126.34087200514 288.475968823974 262.746140741799 325.689303882098 down 120.0 133.0 119.0 305.0 317.0 304.0

SLC40A1 1.57470122786168 2.76070811340699e-15 2.78371401435205e-14 153.408284223346 166.766250016919 163.499952006652 63.370130856414 49.7311307397726 49.2819341400542 up 144.0 162.0 154.0 67.0 60.0 46.0

RPE65 -9.44964938710533 2.88314347154652e-15 2.9024546493035e-14 0 0 0 122.956970318415 155.824209651288 126.418874533183 down 0.0 0.0 0.0 130.0 188.0 118.0

CSPG5 -1.06234931466518 2.88360754119114e-15 2.9024546493035e-14 153.408284223346 165.736828720518 142.266192005788 309.284071493245 334.856280314469 319.261225516004 down 144.0 161.0 134.0 327.0 404.0 298.0

ADGRB3 2.36628979684875 2.91260438660222e-15 2.92990013711648e-14 102.272189482231 78.2360185264557 95.5519200038875 14.1873427290479 19.0636001169128 20.3555814926311 up 96.0 76.0 90.0 15.0 23.0 19.0

ZNF775 1.06284316200621 3.00195158134131e-15 3.01262202535082e-14 313.208580289331 385.003564853874 326.999904013304 159.84406141394 169.914696694223 160.701959152351 up 294.0 374.0 308.0 169.0 205.0 150.0

SPRY4 -2.51065972483598 5.33851870995753e-15 5.30096758186873e-14 14.914694299492 16.4707407424117 13.8019440005615 97.419753406129 77.9121048256438 82.4936723648734 down 14.0 16.0 13.0 103.0 94.0 77.0

SLIT3 -1.01573734339817 5.40382833748888e-15 5.35953466259143e-14 190.695019972076 221.325578726157 162.438264006609 387.78736792731 379.614297980264 395.326819514783 down 179.0 215.0 153.0 410.0 458.0 369.0

DBP -1.12073192856443 5.46474648789351e-15 5.41678206582306e-14 127.840236852788 133.824768532095 124.217496005054 270.505334700514 271.863514710757 297.834297629023 down 120.0 130.0 117.0 286.0 328.0 278.0

GPHN 1.17256587882459 5.55207603423017e-15 5.49691221623957e-14 360.08333380202 289.267384288606 296.210952012051 117.282033226796 144.220279145341 158.559266363653 up 338.0 281.0 279.0 124.0 174.0 148.0

NTF4 1.78115740003047 5.65459693317463e-15 5.59514439532584e-14 135.297584002534 138.971875014099 141.204504005745 51.0744338245725 29.8386784438636 40.7111629852622 up 127.0 135.0 133.0 54.0 36.0 38.0

DSC2 1.05305735464444 5.96535143014744e-15 5.89918582759473e-14 367.540680951766 355.150347258253 371.590800015118 143.765072987686 184.005183737159 200.341775743264 up 345.0 345.0 350.0 152.0 222.0 187.0

ADGRV1 4.40576911300884 6.84663790005439e-15 6.74706492303207e-14 58.5934418908613 67.9418055624483 53.0844000021597 3.78329139441278 1.65770435799242 3.21403918304702 up 55.0 66.0 50.0 4.0 2.0 3.0

AMN1 -1.0077077923863 7.27255852333471e-15 7.13359243690156e-14 172.584319751264 161.619143534915 198.535656008077 348.062808285976 350.604471715397 372.828545233454 down 162.0 157.0 187.0 368.0 423.0 348.0

FAM43A -1.88225992251168 7.80436147467732e-15 7.63754381172927e-14 26.6333826776642 45.2945370416322 37.1590800015118 155.114947170924 119.354713775454 128.561567321881 down 25.0 44.0 35.0 164.0 144.0 120.0

NPAS1 -1.16399158238607 7.89026842918726e-15 7.71270324237924e-14 117.186883781723 133.824768532095 109.353864004449 287.530145975371 261.088436383806 259.265827432459 down 110.0 130.0 103.0 304.0 315.0 242.0

BST1 -1.02698186021816 8.9061141866473e-15 8.6756511973436e-14 170.453649137051 147.207245385305 167.746704006825 316.85065428207 341.487097746439 329.974689459494 down 160.0 143.0 158.0 335.0 412.0 308.0

CNTNAP3 -1.22172128720453 8.94990150033299e-15 8.71329490894488e-14 103.337524789337 95.7361805652681 112.538928004579 226.051660816163 257.773027667821 242.124285122875 down 97.0 93.0 106.0 239.0 311.0 226.0

B4GALNT4 -1.25144711774364 8.99105891081121e-15 8.74331446321136e-14 893.816322662411 1202.36407419606 883.324416035938 2555.61333692583 2286.80316185054 2251.97012092161 down 839.0 1168.0 832.0 2702.0 2759.0 2102.0

PKDCC 1.00650868871399 9.57806572075689e-15 9.30346521270766e-14 359.017998494914 399.415463003484 340.801848013865 186.327101174829 196.437966422102 162.844651941049 up 337.0 388.0 321.0 197.0 237.0 152.0

ULBP2 1.6374536380672 9.83140268812958e-15 9.54406656372006e-14 159.800296065985 152.354351867308 138.019440005615 48.2369652787629 39.7849045918181 57.8527052948463 up 150.0 148.0 130.0 51.0 48.0 54.0

TCF24 -1.18924911302162 1.05433811943896e-14 1.01885269499692e-13 107.598866017763 123.530555568088 101.922048004147 261.047106214482 260.25958420481 237.838899545479 down 101.0 120.0 96.0 276.0 314.0 222.0

SRRM3 -1.40203971638004 1.05753740444114e-14 1.02136166654692e-13 77.7694774187795 76.1771759336542 73.2564720029804 218.485078027338 211.357305644034 169.272730307143 down 73.0 74.0 69.0 231.0 255.0 158.0

RPH3AL 1.21259377679991 1.23642890442277e-14 1.18938703242031e-13 243.961785327404 296.473333363411 233.571360009503 115.39038752959 115.210452880473 102.849253857505 up 229.0 288.0 220.0 122.0 139.0 96.0

CPZ 2.95485500830249 1.29467632914686e-14 1.24330028433944e-13 62.8547831192875 76.1771759336542 70.0714080028508 11.3498741832383 10.7750783269507 4.28538557739602 up 59.0 74.0 66.0 12.0 13.0 4.0

CACNA2D1 1.04790591232653 1.30102965691979e-14 1.24869361972925e-13 373.932692794406 333.532500033837 306.827832012483 149.440010079305 176.545514126193 163.915998335398 up 351.0 324.0 289.0 158.0 213.0 153.0

GPC5 4.62320824947183 1.41147165832875e-14 1.35162972821306e-13 41.5480769771562 70.0006481552498 73.2564720029804 2.83746854580958 2.48655653698863 2.14269278869801 up 39.0 68.0 69.0 3.0 3.0 2.0

LAMC3 -1.56705200473625 1.42773245539068e-14 1.36642868894452e-13 53.2667653553284 62.7946990804447 45.6525840018574 148.494187230702 169.085844515227 161.7733055467 down 50.0 61.0 43.0 157.0 204.0 151.0

RBP1 -5.90185014214028 1.51278746697816e-14 1.4453818212414e-13 1.06533530710657 2.05884259280146 1.06168800004319 86.0698792228907 96.1468527635604 68.5661692383363 down 1.0 2.0 1.0 91.0 116.0 64.0

LOC728392 1.30206067694373 1.60792411541448e-14 1.53368437585142e-13 202.413708350248 235.737476875768 202.78240800825 76.6116507368587 90.3448875105869 93.2071363083635 up 190.0 229.0 191.0 81.0 109.0 87.0

BTN2A2 1.14094123746335 1.63466973791785e-14 1.55656578753954e-13 281.248521076134 340.738449108642 269.668752010971 137.144313047463 121.841270312443 146.774456025814 up 264.0 331.0 254.0 145.0 147.0 137.0

LINC00922 2.83818214200268 1.95346512737742e-14 1.85180186109532e-13 71.3774655761401 73.088912044452 76.44153600311 17.0248112748575 8.28852178996211 5.35673197174503 up 67.0 71.0 72.0 18.0 10.0 5.0

CALB2 3.96934304114938 1.95834188903442e-14 1.85538655482344e-13 51.1360947411153 57.647592598441 69.0097200028076 0.945822848603194 3.31540871598484 7.49942476044304 up 48.0 56.0 65.0 1.0 4.0 7.0

PLLP -2.54294664235901 2.00079549040543e-14 1.89349025740045e-13 12.7840236852788 14.4118981496103 13.8019440005615 85.1240563742875 73.7678439306627 80.3509795761754 down 12.0 14.0 13.0 90.0 89.0 75.0

BMP6 -2.98070244876804 2.25350989705634e-14 2.12671073293228e-13 8.52268245685255 15.441319446011 4.24675200017278 63.370130856414 82.0563657206248 78.2082867874774 down 8.0 15.0 4.0 67.0 99.0 73.0

RNF128 5.02346718888981 2.47032809734368e-14 2.32743926412692e-13 59.6587771979678 63.8241203768454 59.4545280024189 2.83746854580958 1.65770435799242 1.07134639434901 up 56.0 62.0 56.0 3.0 2.0 1.0

FAM174B -1.70437178865444 2.92003067869749e-14 2.74046092504906e-13 39.417406362943 44.2651157452315 49.8993360020301 141.873427290479 123.498974670435 171.415423095841 down 37.0 43.0 47.0 150.0 149.0 160.0

NR4A1 1.16606489701776 3.44390571170507e-14 3.22141152712777e-13 241.831114713191 337.65018521944 288.779136011749 126.740261712828 132.616348639394 127.490220927532 up 227.0 328.0 272.0 134.0 160.0 119.0

PPFIA4 1.79867939975267 3.45680857334275e-14 3.23169631525531e-13 116.121548474616 153.383773163709 122.094120004967 36.8870910955246 30.6675306228598 46.0678949570072 up 109.0 149.0 115.0 39.0 37.0 43.0

TUBB2B 1.82867648573666 3.46887482828181e-14 3.24118806349112e-13 748.930720895918 843.0960417522 712.392648028983 287.530145975371 166.599287978238 194.985043771519 up 703.0 819.0 671.0 304.0 201.0 182.0

IQCK -1.07709231005192 3.64924398204951e-14 3.40783864696354e-13 142.75493115228 129.707083346492 140.142816005702 292.259260218387 267.719253815776 311.761800755561 down 134.0 126.0 132.0 309.0 323.0 291.0

RASSF6 -2.43076496472981 3.6674911528924e-14 3.42299174269958e-13 12.7840236852788 13.3824768532095 22.2954480009071 74.7200050396524 96.1468527635604 89.9930971253164 down 12.0 13.0 21.0 79.0 116.0 84.0

HRASLS 1.79263972891827 3.7189947213749e-14 3.46533391529653e-13 122.513560317255 134.854189828496 112.538928004579 36.8870910955246 29.0098262648674 41.7825093796112 up 115.0 131.0 106.0 39.0 35.0 39.0

KIAA0040 -4.24962065100561 3.92023098667282e-14 3.64482507762007e-13 4.26134122842627 3.0882638892022 2.12337600008639 63.370130856414 58.8485047087309 57.8527052948463 down 4.0 3.0 2.0 67.0 71.0 54.0

LINC00475 2.96066193399448 4.50929082772544e-14 4.17189440861108e-13 47.9400888197956 99.853865750871 85.9967280034987 5.67493709161917 11.6039305059469 12.8561567321881 up 45.0 97.0 81.0 6.0 14.0 12.0

GPR63 1.34724086105336 4.57571242457798e-14 4.22642139980103e-13 184.303008129436 177.060462980926 199.597344008121 75.6658278882555 66.3081743196968 79.2796331818264 up 173.0 172.0 188.0 80.0 80.0 74.0

FAM196B -1.8304186525538 5.02174162914967e-14 4.62327734770627e-13 43.6787475913693 35.0003240776249 28.6655760011662 109.715450437971 126.81438338642 145.703109631465 down 41.0 34.0 27.0 116.0 153.0 136.0

ADORA1 1.76774367508858 5.11613222222124e-14 4.70506405235764e-13 138.493589923854 114.265763900481 123.155808005011 42.5620281871437 29.8386784438636 38.5684701965642 up 130.0 111.0 116.0 45.0 36.0 36.0

NCALD -4.68781728447212 5.542649718427e-14 5.08902364391075e-13 0 2.05884259280146 5.30844000021597 67.1534222508268 51.3888350977651 71.7802084213833 down 0.0 2.0 5.0 71.0 62.0 67.0

ZNF117 1.06959244278472 6.44022345824114e-14 5.87809188483863e-13 607.241125050744 486.916273197546 451.217400018358 240.239003545211 296.729080080643 196.056390165868 up 570.0 473.0 425.0 254.0 358.0 183.0

FOXA1 -2.14620663747016 6.55399224003317e-14 5.97549131034241e-13 23.4373767563445 22.6472685208161 19.1103840007775 100.257221951939 82.0563657206248 107.134639434901 down 22.0 22.0 18.0 106.0 99.0 100.0

DYNC1I1 3.07292870262122 7.9251566798094e-14 7.17925958053323e-13 56.4627712766481 72.0594907480513 61.5779040025053 11.3498741832383 4.97311307397726 6.42807836609403 up 53.0 70.0 58.0 12.0 6.0 6.0

NOS1 -1.95056321602579 9.10341902831962e-14 8.22025151064682e-13 24.5027120634511 24.7061111136176 33.9740160013822 98.3655762547322 113.552748522481 109.277332223599 down 23.0 24.0 32.0 104.0 137.0 102.0

CCDC120 1.18969090931587 9.36007566084242e-14 8.43851419343643e-13 245.027120634511 287.208541695804 221.892792009028 121.065324621209 102.77767019553 107.134639434901 up 230.0 279.0 209.0 128.0 124.0 100.0

NCF2 -3.93662586066021 9.4356037901472e-14 8.50208128750498e-13 2.13067061421314 7.20594907480513 2.12337600008639 62.4243080078108 59.6773568877272 54.6386661117993 down 2.0 7.0 2.0 66.0 72.0 51.0

N4BP2 -1.06685246203194 9.61975522260161e-14 8.65880199101335e-13 167.257643215731 142.060138903301 152.88307200622 284.692677429562 364.694958758333 317.118532727306 down 157.0 138.0 144.0 301.0 440.0 296.0

PPP1R1A 2.29704205023144 9.75472128651e-14 8.77096489349679e-13 72.4428008832467 110.148078714878 96.6136080039307 23.6455712150799 12.4327826849432 21.4269278869801 up 68.0 107.0 91.0 25.0 15.0 20.0

NEBL -1.19146642913021 9.78783271537841e-14 8.79606823334272e-13 112.925542553296 90.5890740832644 108.292176004406 246.859763485434 245.340244982878 218.554664447197 down 106.0 88.0 102.0 261.0 296.0 204.0

MAGEH1 -1.47996533825331 1.13600288173779e-13 1.01658155396926e-12 60.7241125050744 54.5593287092388 76.44153600311 207.1352038441 167.428140157235 159.630612758002 down 57.0 53.0 72.0 219.0 202.0 149.0

ZNF488 1.02034792110842 1.32888014908794e-13 1.18480156450262e-12 303.620562525372 338.679606515841 355.66548001447 157.952415716733 147.535687861325 188.556965405425 up 285.0 329.0 335.0 167.0 178.0 176.0

NLGN4X -8.90327267502052 1.34550880515595e-13 1.19899627350562e-12 0 0 0 90.7989934659067 96.9757049425566 89.9930971253164 down 0.0 0.0 0.0 96.0 117.0 84.0

SLC24A4 -4.44872225585142 1.38107058762944e-13 1.22874662575855e-12 3.19600592131971 4.11768518560293 1.06168800004319 52.0202566731757 48.9022785607764 83.5650187592224 down 3.0 4.0 1.0 55.0 59.0 78.0

SH2B2 -1.8464924785337 1.39314787256511e-13 1.23884120531512e-12 25.5680473705576 40.1474305596286 29.7272640012094 114.444564680987 112.723896343485 116.776756984042 down 24.0 39.0 28.0 121.0 136.0 109.0

ANGPTL4 -3.00873510850588 1.47629519091888e-13 1.31071491269213e-12 6.39201184263941 13.3824768532095 5.30844000021597 70.9367136452396 74.596696109659 56.7813589004973 down 6.0 13.0 5.0 75.0 90.0 53.0

GOLGA2P5 -1.01187716819912 1.89522666660493e-13 1.66606848636676e-12 143.820266459387 192.501782426937 179.4252720073 355.629391074801 319.936941092537 366.40046686736 down 135.0 187.0 169.0 376.0 386.0 342.0

HS6ST3 2.58439622264253 2.46062765626808e-13 2.14308650371112e-12 68.1814596548204 70.0006481552498 82.8116640033692 17.9706341234607 7.45966961096589 11.7848103378391 up 64.0 68.0 78.0 19.0 9.0 11.0

KCNJ16 -3.25701524654812 2.71554608542429e-13 2.3566265720844e-12 7.45734714974598 3.0882638892022 8.49350400034555 57.6951937647949 65.4793221407006 57.8527052948463 down 7.0 3.0 8.0 61.0 79.0 54.0

FGF5 1.63007461284905 3.29583081966239e-13 2.84273798803874e-12 157.669625451772 120.442291678886 165.623328006738 35.9412682469214 54.7042438137499 52.4959733231013 up 148.0 117.0 156.0 38.0 66.0 49.0

LMO2 -2.32054897955203 3.35468205172424e-13 2.89055513510725e-12 20.2413708350248 20.5884259280146 9.55519200038875 82.2865878284779 82.0563657206248 87.8504043366184 down 19.0 20.0 9.0 87.0 99.0 82.0

CADPS2 -2.91021958020472 4.05061043291132e-13 3.47253748651405e-12 10.6533530710657 7.20594907480513 7.43181600030236 63.370130856414 66.3081743196968 59.9953980835443 down 10.0 7.0 7.0 67.0 80.0 56.0

TSGA10 1.28432934724753 4.16150185498327e-13 3.56579875687489e-12 216.263067342633 197.648888908941 169.870080006911 81.3407649798747 82.8852178996211 74.9942476044304 up 203.0 192.0 160.0 86.0 100.0 70.0

RGS5 -1.13524996049574 4.17833223724503e-13 3.57660172303845e-12 119.317554395936 102.942129640073 114.662304004665 237.401534999402 272.692366889753 228.196781996338 down 112.0 100.0 108.0 251.0 329.0 213.0

FCGRT 3.54696185327466 4.34210044517357e-13 3.70555070736727e-12 51.1360947411153 46.323958338033 67.9480320027644 7.56658278882556 2.48655653698863 4.28538557739602 up 48.0 45.0 64.0 8.0 3.0 4.0

LCP1 2.58590253771519 4.39277555108189e-13 3.74502354480761e-12 73.5081361903532 72.0594907480513 77.5032240031532 7.56658278882556 9.11737396895832 21.4269278869801 up 69.0 70.0 73.0 8.0 11.0 20.0

MMP13 -3.19190044474149 4.40154517443162e-13 3.75061243736779e-12 13.8493589923854 3.0882638892022 4.24675200017278 59.5868394620012 60.5062090667234 72.8515548157324 down 13.0 3.0 4.0 63.0 73.0 68.0

SP6 1.82934936148566 4.87494843640178e-13 4.13528425201033e-12 94.8148423324846 138.971875014099 128.464248005227 25.5372169122862 39.7849045918181 36.4257774078662 up 89.0 135.0 121.0 27.0 48.0 34.0

LYPD5 1.61864469068739 5.07556376704e-13 4.30115308722649e-12 170.453649137051 154.41319446011 140.142816005702 69.0450679480332 46.4157220237878 35.3544310135172 up 160.0 150.0 132.0 73.0 56.0 33.0

WDR72 -8.70114750751644 5.56457088636291e-13 4.69909425797546e-12 0 0 0 74.7200050396524 85.3717744366097 81.4223259705244 down 0.0 0.0 0.0 79.0 103.0 76.0

FAM189A2 1.43829852645106 6.00468358159758e-13 5.0581471841006e-12 156.604290144666 179.119305573727 178.363584007257 58.6410166133981 51.3888350977651 81.4223259705244 up 147.0 174.0 168.0 62.0 62.0 76.0

BRSK1 1.61613781240532 6.01163932250024e-13 5.06148956874523e-12 121.448225010149 181.178148166529 132.711000005399 44.4536738843501 52.2176872767613 44.9965485626582 up 114.0 176.0 125.0 47.0 63.0 42.0

SPRY1 -3.88053733746935 6.27450890189315e-13 5.27494693786947e-12 4.26134122842627 3.0882638892022 4.24675200017278 48.2369652787629 75.4255482886552 46.0678949570072 down 4.0 3.0 4.0 51.0 91.0 43.0

PROL1 -8.67075193136418 7.0833541654712e-13 5.92553183027566e-12 0 0 0 75.6658278882555 88.6871831525945 71.7802084213833 down 0.0 0.0 0.0 80.0 107.0 67.0

ETV4 -2.09063290583742 7.38455922352036e-13 6.17140765892624e-12 17.0453649137051 21.6178472244154 28.6655760011662 113.498741832383 89.5160353315907 83.5650187592224 down 16.0 21.0 27.0 120.0 108.0 78.0

TREH 2.02948261059881 7.86023746489283e-13 6.56246538468628e-12 75.6388068045664 102.942129640073 104.045424004233 26.4830397608894 23.2078610118939 19.2842350982821 up 71.0 100.0 98.0 28.0 28.0 18.0

DRD1 3.52882413568215 8.05922335617497e-13 6.72528293860118e-12 52.2014300482219 63.8241203768454 46.7142720019006 0.945822848603194 5.80196525297347 7.49942476044304 up 49.0 62.0 44.0 1.0 7.0 7.0

MND1 -1.15551559651453 8.29676814726925e-13 6.90989441567065e-12 143.820266459387 108.089236122077 95.5519200038875 247.805586334037 264.403845099791 261.408520221157 down 135.0 105.0 90.0 262.0 319.0 244.0

ERV3-1 1.14340562550924 8.37642991263659e-13 6.97281192727586e-12 413.350099157349 345.885555590646 287.717448011706 187.272924023432 159.968470546269 125.347528138834 up 388.0 336.0 271.0 198.0 193.0 117.0

KCNE1 4.06262073106413 8.85214609893231e-13 7.33636765733431e-12 49.0054241269022 68.9712268588491 38.220768001555 1.89164569720639 4.14426089498105 3.21403918304702 up 46.0 67.0 36.0 2.0 5.0 3.0

RADIL -1.65049198889486 9.35919359038701e-13 7.73388972786126e-12 38.3520710558365 37.0591666704264 55.2077760022461 158.898238565337 123.498974670435 127.490220927532 down 36.0 36.0 52.0 168.0 149.0 119.0

CHD5 -1.36142660789121 9.67901755047261e-13 7.98648598660526e-12 63.9201184263941 61.7652777840439 71.133096002894 183.48963262902 153.337653114299 169.272730307143 down 60.0 60.0 67.0 194.0 185.0 158.0

MFNG 1.11063801026508 9.82524402320218e-13 8.09925225075645e-12 287.640532918773 291.326226881407 241.003176009805 119.173678924002 148.364540040322 110.348678617948 up 270.0 283.0 227.0 126.0 179.0 103.0

MOCOS 1.19425451734907 9.9547993268379e-13 8.19583030048752e-12 206.675049578674 229.560949097363 194.288904007905 92.690639163113 99.4622614795453 82.4936723648734 up 194.0 223.0 183.0 98.0 120.0 77.0

EVI2A 2.95514819198418 1.00196446082954e-12 8.2354575286038e-12 51.1360947411153 67.9418055624483 55.2077760022461 6.62075994022236 8.28852178996211 7.49942476044304 up 48.0 66.0 52.0 7.0 10.0 7.0

ZNF503 1.23195990460296 1.12306696204061e-12 9.16855630697248e-12 1819.59270453802 2377.96319468569 1846.27543207512 964.739305575258 727.732213158673 881.718082549231 up 1708.0 2310.0 1739.0 1020.0 878.0 823.0

FOXQ1 -2.01070259163403 1.13755719288746e-12 9.28237902096026e-12 27.6987179847708 16.4707407424117 26.5422000010799 102.148867649145 91.1737396895832 91.0644435196654 down 26.0 16.0 25.0 108.0 110.0 85.0

ADAMTS16 2.55553153891578 1.23163226355259e-12 1.0025877243912e-11 57.5281065837547 96.7656018616688 84.9350400034556 17.9706341234607 16.5770435799242 5.35673197174503 up 54.0 94.0 80.0 19.0 20.0 5.0

SMOC2 -8.58040663219409 1.26655265193155e-12 1.02854275760884e-11 0 0 0 73.7741821910492 70.4524352146779 78.2082867874774 down 0.0 0.0 0.0 78.0 85.0 73.0

PLCB1 2.07660932629688 1.33301314690455e-12 1.07940930729269e-11 76.7041421116729 101.912708343672 106.168800004319 31.2121540039054 19.0636001169128 17.1415423095841 up 72.0 99.0 100.0 33.0 23.0 16.0

NDRG4 -1.61398465062606 1.34794360359148e-12 1.09045676431899e-11 31.9600592131971 47.3533796344337 55.2077760022461 125.794438864225 137.589461713371 148.917148814512 down 30.0 46.0 52.0 133.0 166.0 139.0

LOC400655 1.27645574160027 1.56389124778564e-12 1.260938492979e-11 201.348373043141 166.766250016919 175.178520007127 69.9908907966364 77.0832526466476 77.1369403931284 up 189.0 162.0 165.0 74.0 93.0 72.0

TLCD2 1.01893239663556 1.61368672569561e-12 1.29861535074981e-11 310.012574368011 301.620439845415 302.58108001231 168.356467051369 160.797322725265 119.990796167089 up 291.0 293.0 285.0 178.0 194.0 112.0

OVOL2 -2.85283349894569 1.65283683182198e-12 1.32770481448978e-11 6.39201184263941 16.4707407424117 5.30844000021597 82.2865878284779 57.1908003507385 65.3521300552893 down 6.0 16.0 5.0 87.0 69.0 61.0

LYPD1 1.73873943171731 1.65680807663248e-12 1.33015776389356e-11 175.780325672584 153.383773163709 119.970744004881 28.3746854580958 61.3350612457196 43.9252021683092 up 165.0 149.0 113.0 30.0 74.0 41.0

GEMIN2 -1.03322949140682 1.71014887143901e-12 1.37233168556025e-11 147.016272380706 118.383449086084 140.142816005702 265.776220457498 266.89040163678 297.834297629023 down 138.0 115.0 132.0 281.0 322.0 278.0

HS3ST3B1 1.02107238890922 1.76615561076435e-12 1.41526376756613e-11 332.384615817249 313.973495402223 305.76614401244 126.740261712828 158.310766188276 185.342926222378 up 312.0 305.0 288.0 134.0 191.0 173.0

FAM19A5 -2.99830651472672 2.04861120624976e-12 1.63156905659948e-11 10.6533530710657 9.26479166760659 3.18506400012958 72.828359342446 52.2176872767613 59.9953980835443 down 10.0 9.0 3.0 77.0 63.0 56.0

PCDH7 -2.95090316254166 2.05631912679588e-12 1.63693825225198e-11 4.26134122842627 8.23537037120586 10.6168800004319 59.5868394620012 61.3350612457196 57.8527052948463 down 4.0 8.0 10.0 63.0 74.0 54.0

CSRNP3 3.32691938959895 2.26405893515087e-12 1.79555984838276e-11 66.0507890406073 49.4122222272351 43.529208001771 5.67493709161917 6.63081743196968 3.21403918304702 up 62.0 48.0 41.0 6.0 8.0 3.0

CEP135 -1.04540480755814 2.28917725805294e-12 1.81463091957963e-11 225.851085106593 165.736828720518 156.06813600635 356.575213923404 432.660837436022 338.545460614286 down 212.0 161.0 147.0 377.0 522.0 316.0

PACRGL -1.08831525167071 2.31313393072619e-12 1.83105087787391e-11 131.036242774108 99.853865750871 146.512944005961 268.613689003307 269.376958173768 263.551213009855 down 123.0 97.0 138.0 284.0 325.0 246.0

TRIB2 -8.39553455549742 2.34555055962172e-12 1.85584430079365e-11 0 1.02942129640073 0 111.607096135177 130.129792102405 139.275031265371 down 0.0 1.0 0.0 118.0 157.0 130.0

GDAP1 1.1835220489444 2.77541035688513e-12 2.18371813495746e-11 201.348373043141 189.413518537735 204.905784008336 96.4739305575258 87.0294787946021 78.2082867874774 up 189.0 184.0 193.0 102.0 105.0 73.0

ANO5 -1.04967889224463 2.8515781689008e-12 2.23844922062927e-11 140.624260538067 112.20692130768 118.909056004838 261.992929063085 260.25958420481 246.409670700271 down 132.0 109.0 112.0 277.0 314.0 230.0

HYAL3 1.00556410414978 2.870216450596e-12 2.25203643691969e-11 274.856509233495 269.708379656992 276.038880011231 138.090135896066 150.022244398314 118.91944977274 up 258.0 262.0 260.0 146.0 181.0 111.0

FBXO41 1.20221586470018 2.92982688941199e-12 2.29668058799811e-11 204.544378964461 235.737476875768 205.96747200838 110.661273286574 77.9121048256438 93.2071363083635 up 192.0 229.0 194.0 117.0 94.0 87.0

C5orf63 1.04412845906557 2.95362698976214e-12 2.31426647578958e-11 272.725838619282 252.208217618179 269.668752010971 145.656718684892 129.300939923409 109.277332223599 up 256.0 245.0 254.0 154.0 156.0 102.0

ARAP2 -1.29755137868619 2.95847572283131e-12 2.31699393179669e-11 71.3774655761401 65.8829629696469 77.5032240031532 157.00659286813 182.347479379166 188.556965405425 down 67.0 64.0 73.0 166.0 220.0 176.0

MPZL2 -2.35250954415641 3.47510784884747e-12 2.70658974526327e-11 15.9800296065985 10.2942129640073 18.0486960007343 63.370130856414 77.0832526466476 85.7077115479204 down 15.0 10.0 17.0 67.0 93.0 80.0

PEAR1 1.36190293217094 3.48734201533654e-12 2.7148701167188e-11 160.865631373092 231.619791690165 221.892792009028 81.3407649798747 62.992765603712 96.4211754914105 up 151.0 225.0 209.0 86.0 76.0 90.0

LOC730102 1.42024670240336 3.66602979038775e-12 2.84874058023709e-11 196.021696507609 149.266087978106 200.659032008164 68.09924509943 83.7140700786173 50.3532805344032 up 184.0 145.0 189.0 72.0 101.0 47.0

ACCS 2.02660879671817 3.77495439153728e-12 2.93069328105598e-11 82.0308186472058 110.148078714878 88.1201040035851 16.0789884262543 30.6675306228598 21.4269278869801 up 77.0 107.0 83.0 17.0 37.0 20.0

MYO3A 8.44038155419642 3.9160463022056e-12 3.03605603475345e-11 58.5934418908613 62.7946990804447 59.4545280024189 0 0 0 up 55.0 61.0 56.0 0.0 0.0 0.0

LOC728613 1.6066540555791 4.0007412820078e-12 3.09888236475593e-11 131.036242774108 128.677662050092 122.094120004967 29.320508306699 49.7311307397726 46.0678949570072 up 123.0 125.0 115.0 31.0 60.0 43.0

TMEM255B 2.19449487574515 4.04019863656541e-12 3.12658587955313e-11 90.5535011040583 94.7067592688674 89.1817920036283 30.2663311553022 9.11737396895832 21.4269278869801 up 85.0 92.0 84.0 32.0 11.0 20.0

LOC101928272 8.33138102601636 4.09749812045819e-12 3.16948028130419e-11 92.6841717182715 127.648240753691 106.168800004319 0.945822848603194 0 0 up 87.0 124.0 100.0 1.0 0.0 0.0

LINC00623 -1.14278656537299 4.31858881378733e-12 3.33440722450125e-11 95.8801776395912 97.7950231580696 129.52593600527 241.184826393815 216.330418718011 257.123134643761 down 90.0 95.0 122.0 255.0 261.0 240.0

NKD1 -1.51401889043825 4.34283720364112e-12 3.34855085251163e-11 50.0707594340087 55.5887500056395 43.529208001771 125.794438864225 133.44520081839 168.201383912794 down 47.0 54.0 41.0 133.0 161.0 157.0

PTPRR -8.29828099978067 4.52821898216387e-12 3.4851444596936e-11 0 1.02942129640073 0 99.3113991033354 130.129792102405 126.418874533183 down 0.0 1.0 0.0 105.0 157.0 118.0

TMSB15A 1.62667035821899 5.14363704440621e-12 3.94267925485254e-11 129.970907467001 115.295185196882 106.168800004319 35.9412682469214 43.9291654867992 33.2117382248192 up 122.0 112.0 100.0 38.0 53.0 31.0

GGT1 1.22948536961996 5.16891995511218e-12 3.95847667448464e-11 168.322978522838 177.060462980926 181.548648007386 75.6658278882555 77.9121048256438 70.7088620270343 up 158.0 172.0 171.0 80.0 94.0 66.0

FAM83A -2.80516287458989 5.34671070464243e-12 4.08908710323443e-11 14.914694299492 4.11768518560293 8.49350400034555 72.828359342446 54.7042438137499 64.2807836609403 down 14.0 4.0 8.0 77.0 66.0 60.0

PRODH -2.32670281290532 5.66788898726144e-12 4.32690578838255e-11 11.7186883781723 17.5001620388124 14.8636320006047 87.0157020714939 67.9658786776893 66.4234764496383 down 11.0 17.0 14.0 92.0 82.0 62.0

TMEM184A 1.38103206575491 5.69548632205429e-12 4.34601523854053e-11 142.75493115228 181.178148166529 153.944760006263 70.9367136452396 53.0465394557575 59.9953980835443 up 134.0 176.0 145.0 75.0 64.0 56.0

SPSB4 -1.38967300979684 5.98588443652463e-12 4.55734302717875e-11 45.8094182055824 83.3831250084593 81.749976003326 174.977226991591 194.780262064109 183.20023343368 down 43.0 81.0 77.0 185.0 235.0 171.0

ADAMTS17 -1.85033051348199 6.66558521863263e-12 5.05708609316189e-11 45.8094182055824 40.1474305596286 16.9870080006911 129.577730258638 135.102905176382 106.063293040552 down 43.0 39.0 16.0 137.0 163.0 99.0

NGF 1.14267568881145 6.72344689015815e-12 5.0959816697664e-11 212.001726114207 273.826064842595 208.090848008466 105.932159043558 97.8045571215528 111.420025012297 up 199.0 266.0 196.0 112.0 118.0 104.0

LOC284344 2.57441188300425 6.92001682699662e-12 5.2379394570743e-11 70.3121302690335 52.5004861164373 62.6395920025485 9.45822848603194 9.11737396895832 12.8561567321881 up 66.0 51.0 59.0 10.0 11.0 12.0

MTMR9LP -1.53089561851832 7.34332202945214e-12 5.53855187795722e-11 63.9201184263941 42.20627315243 38.220768001555 140.927604441876 132.616348639394 143.560416842767 down 60.0 41.0 36.0 149.0 160.0 134.0

UGT1A9 -2.37128666439131 7.82274972934359e-12 5.88181892654596e-11 9.58801776395912 17.5001620388124 16.9870080006911 67.1534222508268 64.6504699617044 97.4925218857595 down 9.0 17.0 16.0 71.0 78.0 91.0

FFAR4 -2.21232120500325 8.52076035617608e-12 6.38680001918685e-11 19.1760355279182 15.441319446011 18.0486960007343 104.040513346351 67.137026498693 72.8515548157324 down 18.0 15.0 17.0 110.0 81.0 68.0

ICAM1 1.92190671784393 8.84846992059633e-12 6.629503779518e-11 96.9455129466977 81.3242824156578 84.9350400034556 18.9164569720639 24.8655653698863 25.7123134643761 up 91.0 79.0 80.0 20.0 30.0 24.0

PHOSPHO1 1.31938877722084 8.96883686780585e-12 6.71671514326397e-11 153.408284223346 203.825416687345 177.301896007213 81.3407649798747 58.8485047087309 74.9942476044304 up 144.0 198.0 167.0 86.0 71.0 70.0

SLC26A10 1.47252245142965 9.27693997890326e-12 6.93212894762334e-11 125.709566238575 126.61881945729 132.711000005399 52.0202566731757 44.7580176657954 41.7825093796112 up 118.0 123.0 125.0 55.0 54.0 39.0

CXCL16 -1.22596791978683 9.86666682063463e-12 7.35010272390284e-11 93.749507025378 85.4419676012608 66.8863440027212 178.760518386004 204.726488212064 191.771004588472 down 88.0 83.0 63.0 189.0 247.0 179.0

HMGN5 1.13175713289592 1.02960855746592e-11 7.65652720082211e-11 273.791173926388 258.384745396584 264.360312010755 99.3113991033354 155.824209651288 106.063293040552 up 257.0 251.0 249.0 105.0 188.0 99.0

FAM222A -1.05013324168319 1.12685519895696e-11 8.34306253073905e-11 158.734960758879 263.531851878587 209.152536008509 479.53218424182 364.694958758333 466.035681541817 down 149.0 256.0 197.0 507.0 440.0 435.0

IQCD -1.80204159652391 1.33531202200755e-11 9.81778891180898e-11 33.0253945203036 23.6766898172168 28.6655760011662 112.55291898378 93.6602962265718 91.0644435196654 down 31.0 23.0 27.0 119.0 113.0 85.0

OLFML2B -2.21852789688287 1.52810987452809e-11 1.11722836747975e-10 17.0453649137051 11.3236342604081 19.1103840007775 73.7741821910492 77.9121048256438 68.5661692383363 down 16.0 11.0 18.0 78.0 94.0 64.0

PRKCQ 8.1279203207435 1.60477779566914e-11 1.17025122077638e-10 102.272189482231 109.118657418478 72.1947840029372 0 0.82885217899621 0 up 96.0 106.0 68.0 0.0 1.0 0.0

NMUR2 -8.21057693093995 1.62080716779506e-11 1.18143173074219e-10 0 0 0 63.370130856414 48.9022785607764 59.9953980835443 down 0.0 0.0 0.0 67.0 59.0 56.0

LINC01410 3.7570299960561 1.63119165896121e-11 1.18848975065819e-10 43.6787475913693 50.4416435236359 44.5908960018142 0 5.80196525297347 4.28538557739602 up 41.0 49.0 42.0 0.0 7.0 4.0

CCNYL2 1.64995230284968 1.80228503672937e-11 1.30808519803751e-10 147.016272380706 131.765925939294 128.464248005227 37.8329139441278 62.1639134247158 27.8550062530741 up 138.0 128.0 121.0 40.0 75.0 26.0

CITED4 -1.21002932487032 1.86177127788468e-11 1.35010297291809e-10 102.272189482231 88.530231490463 67.9480320027644 205.243558146893 183.176331558163 211.055239686754 down 96.0 86.0 64.0 217.0 221.0 197.0

CXCL14 3.18230443686196 2.12279798335984e-11 1.53348391633756e-10 43.6787475913693 44.2651157452315 57.3311520023325 6.62075994022236 4.14426089498105 5.35673197174503 up 41.0 43.0 54.0 7.0 5.0 5.0

COL16A1 -1.46591761203526 2.13778754986387e-11 1.54299621195969e-10 59.6587771979678 56.6181713020403 40.3441440016414 140.927604441876 125.985531207424 167.130037518445 down 56.0 55.0 38.0 149.0 152.0 156.0

RPS6KL1 -1.90700884193793 2.20928403284338e-11 1.59121052365505e-10 25.5680473705576 30.882638892022 20.1720720008207 118.227856075399 87.8583309735983 81.4223259705244 down 24.0 30.0 19.0 125.0 106.0 76.0

SPANXC -8.04979421135377 2.2252159063924e-11 1.60200414170366e-10 0 0 1.06168800004319 91.7448163145099 88.6871831525945 119.990796167089 down 0.0 0.0 1.0 97.0 107.0 112.0

FOXL1 -1.18328785232186 2.49548602596012e-11 1.78973468584947e-10 88.4228304898452 92.6479166760659 90.2434800036715 242.130649242418 176.545514126193 198.199082954566 down 83.0 90.0 85.0 256.0 213.0 185.0

TMEM182 1.67516843212893 2.87386817242078e-11 2.04809957260446e-10 105.46819540355 105.000972232875 100.860360004103 29.320508306699 27.3521219068749 41.7825093796112 up 99.0 102.0 95.0 31.0 33.0 39.0

NMNAT2 -1.60157399267208 3.11526332620305e-11 2.21454304430884e-10 39.417406362943 39.1180092632278 35.0357040014254 109.715450437971 101.119965837538 134.989645687975 down 37.0 38.0 33.0 116.0 122.0 126.0

MERTK 1.40408927384501 3.15987662199451e-11 2.24531501579644e-10 143.820266459387 144.118981496103 145.451256005918 64.3159537050172 40.6137567708143 59.9953980835443 up 135.0 140.0 137.0 68.0 49.0 56.0

ADGRF1 -7.98067756436049 3.20523983703963e-11 2.27522485896645e-10 1.06533530710657 0 0 94.5822848603194 102.77767019553 87.8504043366184 down 1.0 0.0 0.0 100.0 124.0 82.0

C10orf90 1.57627853298002 3.28000977788793e-11 2.32677410541966e-10 108.66420132487 107.059814825676 113.600616004622 31.2121540039054 41.4426089498105 37.4971238022152 up 102.0 104.0 107.0 33.0 50.0 35.0

RYR1 3.7091489405226 3.30485542210112e-11 2.34341778360791e-10 46.874753512689 32.9414814848234 55.2077760022461 1.89164569720639 4.14426089498105 4.28538557739602 up 44.0 32.0 52.0 2.0 5.0 4.0

RORA 2.11971345232752 3.48601928589741e-11 2.46877787220327e-10 89.4881657969518 59.7064351912425 83.8733520034124 22.6997483664767 14.9193392219318 16.0701959152351 up 84.0 58.0 79.0 24.0 18.0 15.0

GLMP -3.44363115479397 3.54861349823667e-11 2.5099587749532e-10 6.39201184263941 7.20594907480513 0 51.0744338245725 47.244574202784 50.3532805344032 down 6.0 7.0 0.0 54.0 57.0 47.0

DHRS3 -1.2343602859273 3.60450081203629e-11 2.54736102444284e-10 70.3121302690335 71.0300694516505 75.3798480030668 185.381278326226 151.679948756307 173.558115884539 down 66.0 69.0 71.0 196.0 183.0 162.0

ITPKB 1.62786800743174 3.80595497344588e-11 2.68413310783402e-10 118.252219088829 101.912708343672 98.7369840040171 28.3746854580958 37.2983480548295 37.4971238022152 up 111.0 99.0 93.0 30.0 45.0 35.0

RIMBP2 6.97210630883057 4.2389782746923e-11 2.98579176604106e-10 88.4228304898452 71.0300694516505 75.3798480030668 0 1.65770435799242 0 up 83.0 69.0 71.0 0.0 2.0 0.0

RHOF -2.1403629419301 4.26871019948548e-11 3.00423559531716e-10 13.8493589923854 17.5001620388124 18.0486960007343 77.5574735854619 64.6504699617044 76.0655939987794 down 13.0 17.0 17.0 82.0 78.0 71.0

SAMD9L -1.85414459550304 4.46337294111516e-11 3.13862754763349e-10 43.6787475913693 17.5001620388124 26.5422000010799 93.6364620117162 114.381600701477 108.20598582925 down 41.0 17.0 25.0 99.0 138.0 101.0

HOXA13 -1.36308267500949 4.48452136645355e-11 3.15088311686948e-10 59.6587771979678 48.3828009308344 60.5162160024621 157.952415716733 130.129792102405 145.703109631465 down 56.0 47.0 57.0 167.0 157.0 136.0

PAG1 2.70664395323771 4.54272461041918e-11 3.18649088614911e-10 56.4627712766481 64.8535416732461 47.7759600019437 8.51240563742875 12.4327826849432 4.28538557739602 up 53.0 63.0 45.0 9.0 15.0 4.0

EAF2 3.12148138990757 4.56148314874814e-11 3.19832469121661e-10 56.4627712766481 41.1768518560293 47.7759600019437 4.72911424301597 8.28852178996211 3.21403918304702 up 53.0 40.0 45.0 5.0 10.0 3.0

PLXNB3 2.6238320744193 4.59954823898002e-11 3.22101476512284e-10 44.7440828984759 82.3537037120586 66.8863440027212 8.51240563742875 6.63081743196968 17.1415423095841 up 42.0 80.0 63.0 9.0 8.0 16.0

ACSS3 3.49390885654441 5.32332987739943e-11 3.70794441295832e-10 67.1161243477138 27.7943750028198 54.1460880022029 6.62075994022236 2.48655653698863 4.28538557739602 up 63.0 27.0 51.0 7.0 3.0 4.0

PLIN2 2.23363629087838 5.48881053060854e-11 3.81849898926114e-10 63.9201184263941 66.9123842660476 69.0097200028076 10.4040513346351 13.2616348639394 19.2842350982821 up 60.0 65.0 65.0 11.0 16.0 18.0

PLAC8 -1.17395787044972 5.5050403725017e-11 3.82664685720881e-10 87.3574951827386 101.912708343672 69.0097200028076 183.48963262902 196.437966422102 203.555814926311 down 82.0 99.0 65.0 194.0 237.0 190.0

CCIN 3.06077764387232 5.66800607130282e-11 3.93508290360122e-10 40.4827416700496 53.5299074128381 53.0844000021597 3.78329139441278 9.11737396895832 4.28538557739602 up 38.0 52.0 50.0 4.0 11.0 4.0

VSIG1 5.22551996797513 6.04223550607114e-11 4.18118747846589e-10 43.6787475913693 50.4416435236359 45.6525840018574 1.89164569720639 1.65770435799242 0 up 41.0 49.0 43.0 2.0 2.0 0.0

ZNF521 4.06594387125481 6.26354101708324e-11 4.32549469341174e-10 47.9400888197956 37.0591666704264 40.3441440016414 3.78329139441278 2.48655653698863 1.07134639434901 up 45.0 36.0 38.0 4.0 3.0 1.0

KL 2.4179282770943 6.84667070477251e-11 4.70899722853619e-10 57.5281065837547 57.647592598441 69.0097200028076 15.1331655776511 12.4327826849432 6.42807836609403 up 54.0 56.0 65.0 16.0 15.0 6.0

COX7A1 -2.35763129784461 8.09558823988077e-11 5.54098039529617e-10 7.45734714974598 13.3824768532095 21.2337600008639 64.3159537050172 89.5160353315907 61.0667444778933 down 7.0 13.0 20.0 68.0 108.0 57.0

SLC7A8 2.37080211616997 8.69243086680492e-11 5.93988619942216e-10 49.0054241269022 62.7946990804447 72.1947840029372 12.2956970318415 12.4327826849432 10.7134639434901 up 46.0 61.0 68.0 13.0 15.0 10.0

MTHFD2L -1.19301869883914 8.79182160118161e-11 6.00538136790389e-10 67.1161243477138 76.1771759336542 102.98373600419 187.272924023432 188.14944463214 187.485619011076 down 63.0 74.0 97.0 198.0 227.0 175.0

SPRY2 -1.10385195002195 9.37162137373735e-11 6.39368771933591e-10 87.3574951827386 92.6479166760659 110.415552004492 225.10583796756 185.662888095151 214.269278869801 down 82.0 90.0 104.0 238.0 224.0 200.0

GPRIN2 1.19594230666358 1.0014368604109e-10 6.82120643963033e-10 161.930966680198 160.589722238514 192.165528007818 67.1534222508268 82.0563657206248 74.9942476044304 up 152.0 156.0 181.0 71.0 99.0 70.0

CXCL3 -1.90295577122682 1.04670919267933e-10 7.12098543132039e-10 23.4373767563445 30.882638892022 14.8636320006047 83.2324106770811 91.1737396895832 84.6363651535714 down 22.0 30.0 14.0 88.0 110.0 79.0

LOC645166 5.18464544812843 1.07354175152352e-10 7.28597647067642e-10 53.2667653553284 39.1180092632278 43.529208001771 1.89164569720639 1.65770435799242 0 up 50.0 38.0 41.0 2.0 2.0 0.0

HS3ST1 -1.61620106674679 1.14686917557853e-10 7.74639706311812e-10 35.1560651345168 54.5593287092388 36.0973920014686 164.573175656956 114.381600701477 107.134639434901 down 33.0 53.0 34.0 174.0 138.0 100.0

IL1A -2.25517870419832 1.15610911784447e-10 7.80258504234473e-10 13.8493589923854 9.26479166760659 19.1103840007775 71.8825364938428 67.9658786776893 61.0667444778933 down 13.0 9.0 18.0 76.0 82.0 57.0

TGM1 -1.67579967257613 1.18527443397738e-10 7.98351845390728e-10 30.8947239060905 31.9120601884227 26.5422000010799 92.690639163113 99.4622614795453 93.2071363083635 down 29.0 31.0 25.0 98.0 120.0 87.0

CALN1 7.9662274197008 1.19052327733545e-10 8.00931863306694e-10 29.8293885989839 51.4710648200366 48.8376480019869 0 0 0 up 28.0 50.0 46.0 0.0 0.0 0.0

CD37 -1.76718128927151 1.24574478310339e-10 8.36090199119311e-10 37.2867357487299 36.0297453740256 19.1103840007775 120.119501772606 109.4084876275 84.6363651535714 down 35.0 35.0 18.0 127.0 132.0 79.0

ZNF98 7.75984678029212 1.44508813978543e-10 9.64150968411389e-10 79.9001480329926 76.1771759336542 63.7012800025917 0.945822848603194 0 0 up 75.0 74.0 60.0 1.0 0.0 0.0

LRRC8B 1.27616850635506 1.44570001373057e-10 9.64179457976213e-10 165.126972601518 146.177824088904 134.834376005486 56.7493709161917 67.9658786776893 58.9240516891953 up 155.0 142.0 127.0 60.0 82.0 55.0

CES1 -4.39071699378149 1.46522365086956e-10 9.76431496684904e-10 2.13067061421314 4.11768518560293 0 42.5620281871437 49.7311307397726 39.6398165909132 down 2.0 4.0 0.0 45.0 60.0 37.0

RET -1.03212345805278 1.54138569822793e-10 1.02516975767496e-09 109.729536631977 110.148078714878 112.538928004579 257.263814820069 220.474679612992 201.413122137613 down 103.0 107.0 106.0 272.0 266.0 188.0

SNTG2 7.88403085829208 1.65067297913798e-10 1.09613485952949e-09 39.417406362943 44.2651157452315 39.2824560015982 0 0 0 up 37.0 43.0 37.0 0.0 0.0 0.0

ATOH8 1.04027424490746 1.69966464186782e-10 1.12646005607359e-09 217.32840264974 211.03136576215 198.535656008077 114.444564680987 96.9757049425566 93.2071363083635 up 204.0 205.0 187.0 121.0 117.0 87.0

OPLAH 1.25118452609379 1.87395907997958e-10 1.24003385995524e-09 133.166913388321 184.266412055731 164.561640006695 71.8825364938428 66.3081743196968 64.2807836609403 up 125.0 179.0 155.0 76.0 80.0 60.0

POU5F1 -1.01764651876784 1.9300809837415e-10 1.27319205080144e-09 108.66420132487 112.20692130768 95.5519200038875 221.322546573147 210.528453465037 208.912546898056 down 102.0 109.0 90.0 234.0 254.0 195.0

NPY4R -1.13689011463913 1.97590110872766e-10 1.30189672430364e-09 106.533530710657 90.5890740832644 79.6266000032396 230.780775059179 202.239931675075 174.629462278888 down 100.0 88.0 75.0 244.0 244.0 163.0

TRIM9 2.51099415589916 2.00647196858424e-10 1.32049864599134e-09 60.7241125050744 48.3828009308344 55.2077760022461 9.45822848603194 12.4327826849432 6.42807836609403 up 57.0 47.0 52.0 10.0 15.0 6.0

CCDC144NL 7.86067373407396 2.01333771110428e-10 1.32450255635365e-09 46.874753512689 39.1180092632278 35.0357040014254 0 0 0 up 44.0 38.0 33.0 0.0 0.0 0.0

CELSR3 1.15294654051846 2.04272589958592e-10 1.34279304381007e-09 164.061637294412 161.619143534915 162.438264006609 72.828359342446 73.7678439306627 72.8515548157324 up 154.0 157.0 153.0 77.0 89.0 68.0

ADGRL3 -1.98893584513146 2.22108121824058e-10 1.45439179887884e-09 19.1760355279182 22.6472685208161 14.8636320006047 67.1534222508268 82.8852178996211 74.9942476044304 down 18.0 22.0 14.0 71.0 100.0 70.0

FAM84A -7.83852674652724 2.23247794554683e-10 1.45959769963579e-09 0 0 0 30.2663311553022 52.2176872767613 50.3532805344032 down 0.0 0.0 0.0 32.0 63.0 47.0

FIBIN 4.67473944101661 2.43744464964577e-10 1.58808893711536e-09 41.5480769771562 38.0885879668271 40.3441440016414 0.945822848603194 1.65770435799242 2.14269278869801 up 39.0 37.0 38.0 1.0 2.0 2.0

PCDH20 -1.05639568516326 2.50025956498527e-10 1.62713780372072e-09 123.578895624362 112.20692130768 93.4285440038011 197.676975358068 233.736314476931 253.909095460714 down 116.0 109.0 88.0 209.0 282.0 237.0

KRTAP2-3 2.07614803310905 2.60765963839948e-10 1.69312971538855e-09 64.9854537335007 70.0006481552498 76.44153600311 19.8622798206671 19.8924522959091 9.64211754914105 up 61.0 68.0 72.0 21.0 24.0 9.0

ACBD7 1.26196645448066 2.65495416036511e-10 1.72251717642991e-09 139.55892523096 144.118981496103 142.266192005788 67.1534222508268 53.8753916347537 56.7813589004973 up 131.0 140.0 134.0 71.0 65.0 53.0

SALL3 4.77649898644705 2.69391457747694e-10 1.74578855939018e-09 53.2667653553284 31.9120601884227 41.4058320016846 0 4.14426089498105 0 up 50.0 31.0 39.0 0.0 5.0 0.0

TACR1 -4.42614616259594 2.81116529073237e-10 1.82037997037486e-09 2.13067061421314 4.11768518560293 0 52.0202566731757 27.3521219068749 56.7813589004973 down 2.0 4.0 0.0 55.0 33.0 53.0

ZNF99 -4.02165487492122 3.24126576888289e-10 2.09488905474537e-09 6.39201184263941 1.02942129640073 1.06168800004319 31.2121540039054 65.4793221407006 39.6398165909132 down 6.0 1.0 1.0 33.0 79.0 37.0

CLDN3 -6.63957167729026 3.37757620202945e-10 2.17717430983177e-09 0 0 2.12337600008639 71.8825364938428 66.3081743196968 71.7802084213833 down 0.0 0.0 2.0 76.0 80.0 67.0

FILIP1L -1.49722039683362 3.50572927625406e-10 2.2572046347299e-09 42.6134122842627 36.0297453740256 36.0973920014686 113.498741832383 100.291113658541 110.348678617948 down 40.0 35.0 34.0 120.0 121.0 103.0

SLC2A2 7.76893992725291 3.64098282778739e-10 2.33984253045214e-09 41.5480769771562 40.1474305596286 31.8506400012958 0 0 0 up 39.0 39.0 30.0 0.0 0.0 0.0

FOXS1 -4.59745503508652 3.66998138581836e-10 2.35676418972795e-09 1.06533530710657 1.02942129640073 3.18506400012958 40.6703824899374 39.7849045918181 47.1392413513562 down 1.0 1.0 3.0 43.0 48.0 44.0

EPHX2 3.07932607563463 3.73588896351611e-10 2.3953807358805e-09 33.0253945203036 43.2356944488308 58.3928400023757 4.72911424301597 5.80196525297347 5.35673197174503 up 31.0 42.0 55.0 5.0 7.0 5.0

ANKRD2 1.0041725970514 3.79155616772513e-10 2.43015366936299e-09 198.152367121822 221.325578726157 204.905784008336 110.661273286574 101.948818016534 98.5638682801085 up 186.0 215.0 193.0 117.0 123.0 92.0

RAG1 2.41055606987395 4.24392741525801e-10 2.7098428350724e-09 58.5934418908613 64.8535416732461 47.7759600019437 7.56658278882556 9.11737396895832 16.0701959152351 up 55.0 63.0 45.0 8.0 11.0 15.0

NUDT6 -1.40237258886473 4.32890055929472e-10 2.76201790864228e-09 49.0054241269022 40.1474305596286 45.6525840018574 119.173678924002 111.066191985492 126.418874533183 down 46.0 39.0 43.0 126.0 134.0 118.0

HMCN1 -2.1482333026114 4.42296781780242e-10 2.81991248903173e-09 17.0453649137051 14.4118981496103 12.7402560005183 57.6951937647949 74.596696109659 63.2094372665913 down 16.0 14.0 12.0 61.0 90.0 59.0

TEX15 7.58367961982278 4.58623473152581e-10 2.91960978399275e-09 75.6388068045664 68.9712268588491 49.8993360020301 0 0.82885217899621 0 up 71.0 67.0 47.0 0.0 1.0 0.0

EPB41L3 4.36999756055894 4.61050185405876e-10 2.93395572531012e-09 38.3520710558365 47.3533796344337 30.7889520012526 0 2.48655653698863 3.21403918304702 up 36.0 46.0 29.0 0.0 3.0 3.0

SLC25A53 1.51063679044178 4.64427595191753e-10 2.95322952798359e-09 128.905572159895 92.6479166760659 125.279184005097 32.1579768525086 46.4157220237878 42.8538557739602 up 121.0 90.0 118.0 34.0 56.0 40.0

C1S -1.09378722033254 4.83260717435375e-10 3.06608110612556e-09 79.9001480329926 83.3831250084593 81.749976003326 177.814695537401 175.716661947197 169.272730307143 down 75.0 81.0 77.0 188.0 212.0 158.0

PTGER2 2.15729875835016 4.99628272696639e-10 3.16400109887143e-09 73.5081361903532 84.41254630486 57.3311520023325 22.6997483664767 17.4058957589204 7.49942476044304 up 69.0 82.0 54.0 24.0 21.0 7.0

STRIP2 1.12803227263457 5.17687198903181e-10 3.27591376519233e-09 192.825690586289 195.590046316139 150.759696006134 87.0157020714939 80.3986613626324 79.2796331818264 up 181.0 190.0 142.0 92.0 97.0 74.0

BTBD19 1.36912953827451 5.2993162910052e-10 3.34964246155329e-09 142.75493115228 159.560300942113 105.107112004276 51.0744338245725 52.2176872767613 54.6386661117993 up 134.0 155.0 99.0 54.0 63.0 51.0

KHDC1 1.16471625649157 5.33435764208239e-10 3.36927734738537e-09 206.675049578674 198.678310205341 243.126552009891 127.686084561431 76.2544004676514 85.7077115479204 up 194.0 193.0 229.0 135.0 92.0 80.0

MAP10 -7.69689198249483 5.47448925656536e-10 3.45649824846132e-09 0 0 0 34.049622549715 32.3252349808522 54.6386661117993 down 0.0 0.0 0.0 36.0 39.0 51.0

CACNA1C 7.69976568422807 5.54334507111802e-10 3.49866861045973e-09 34.0907298274102 40.1474305596286 33.9740160013822 0 0 0 up 32.0 39.0 32.0 0.0 0.0 0.0

ANKRD20A9P 1.98423507123046 5.9355899420123e-10 3.74065824470567e-09 77.7694774187795 72.0594907480513 59.4545280024189 18.9164569720639 14.0904870429356 20.3555814926311 up 73.0 70.0 56.0 20.0 17.0 19.0

BCHE -7.66158376669819 5.96142752811788e-10 3.75554415493927e-09 0 0 0 32.1579768525086 45.5868698447916 39.6398165909132 down 0.0 0.0 0.0 34.0 55.0 37.0

ZNF90 -1.21862819614138 5.97816425261448e-10 3.76468782302191e-09 54.332100662435 76.1771759336542 79.6266000032396 152.277478625114 179.860922842178 156.416573574955 down 51.0 74.0 75.0 161.0 217.0 146.0

FAM201A 1.11892177966785 5.98953005463304e-10 3.77044366872849e-09 246.092455941617 234.708055579367 163.499952006652 101.203044800542 91.1737396895832 104.991946646203 up 231.0 228.0 154.0 107.0 110.0 98.0

APOBEC3C 1.08455139922652 6.24018020018455e-10 3.91223732757684e-09 185.368343436543 169.854513906121 192.165528007818 76.6116507368587 96.1468527635604 84.6363651535714 up 174.0 165.0 181.0 81.0 116.0 79.0

SMARCD3 1.18669868954383 6.77063821598635e-10 4.23696384849681e-09 147.016272380706 149.266087978106 169.870080006911 66.2075994022236 61.3350612457196 78.2082867874774 up 138.0 145.0 160.0 70.0 74.0 73.0

UNC5B -1.11854669285906 7.04479477214581e-10 4.40039909440081e-09 87.3574951827386 118.383449086084 82.8116640033692 238.347357848005 180.689775021174 208.912546898056 down 82.0 115.0 78.0 252.0 218.0 195.0

ENPP5 2.76583610742159 7.44861745601943e-10 4.64749833167474e-09 47.9400888197956 38.0885879668271 59.4545280024189 9.45822848603194 8.28852178996211 3.21403918304702 up 45.0 37.0 56.0 10.0 10.0 3.0

CYP26B1 1.94535924208492 7.83285966666623e-10 4.87825892475463e-09 96.9455129466977 54.5593287092388 82.8116640033692 18.9164569720639 22.3790088328977 19.2842350982821 up 91.0 53.0 78.0 20.0 27.0 18.0

RBM24 -1.01448715804507 8.05077493926246e-10 5.01029123699875e-09 113.990877860403 120.442291678886 94.4902320038443 234.564066453592 194.780262064109 236.76755315113 down 107.0 117.0 89.0 248.0 235.0 221.0

BDH1 1.25722011865445 8.50119609787494e-10 5.27896854464815e-09 168.322978522838 173.972199091724 141.204504005745 87.0157020714939 61.3350612457196 53.5673197174503 up 158.0 169.0 133.0 92.0 74.0 50.0

EBF3 1.62820816673506 8.51561126210906e-10 5.28598221986542e-09 99.0761835609109 108.089236122077 80.6882880032828 23.6455712150799 32.3252349808522 37.4971238022152 up 93.0 105.0 76.0 25.0 39.0 35.0

FBLN7 3.51759027015386 8.83341646298806e-10 5.47723553744574e-09 46.874753512689 36.0297453740256 33.9740160013822 1.89164569720639 5.80196525297347 2.14269278869801 up 44.0 35.0 32.0 2.0 7.0 2.0

TMTC1 -1.65921844473248 9.12939762792795e-10 5.65662315650587e-09 39.417406362943 19.5590046316139 36.0973920014686 87.9615249200971 103.606522374526 108.20598582925 down 37.0 19.0 34.0 93.0 125.0 101.0

MXD1 1.1664392544005 9.42675464460331e-10 5.83446195394885e-09 157.669625451772 140.0012963105 157.129824006393 62.4243080078108 70.4524352146779 69.6375156326853 up 148.0 136.0 148.0 66.0 85.0 65.0

VSTM4 1.63273840492119 9.61229350108954e-10 5.94712388270478e-09 78.8348127258861 91.6184953796652 92.3668560037579 28.3746854580958 25.6944175488825 31.0690454361212 up 74.0 89.0 87.0 30.0 31.0 29.0

SNHG15 -1.43856844795328 9.77794275052151e-10 6.04740234369603e-09 47.9400888197956 38.0885879668271 39.2824560015982 102.148867649145 109.4084876275 128.561567321881 down 45.0 37.0 37.0 108.0 132.0 120.0

BISPR -1.84988295205612 9.84077983606963e-10 6.08182453203282e-09 27.6987179847708 22.6472685208161 15.9253200006479 91.7448163145099 76.2544004676514 70.7088620270343 down 26.0 22.0 15.0 97.0 92.0 66.0

ANXA2R -1.20537907482448 9.92945546490622e-10 6.13439006475242e-09 58.5934418908613 75.1477546372535 73.2564720029804 150.385832927908 144.220279145341 184.271579828029 down 55.0 73.0 69.0 159.0 174.0 172.0

CCDC68 1.04747627151482 1.08820042707412e-09 6.68872105756008e-09 205.609714271568 178.089884277327 186.857088007602 81.3407649798747 101.948818016534 92.1357899140145 up 193.0 173.0 176.0 86.0 123.0 86.0

DDX26B 1.39794098447392 1.09669420854075e-09 6.73401296661476e-09 134.232248695428 109.118657418478 158.191512006436 53.9119023703821 59.6773568877272 37.4971238022152 up 126.0 106.0 149.0 57.0 72.0 35.0

LACC1 1.56395674770643 1.10997669586466e-09 6.81268305360412e-09 83.0961539543123 108.089236122077 109.353864004449 28.3746854580958 40.6137567708143 32.1403918304702 up 78.0 105.0 103.0 30.0 49.0 30.0

SLC46A3 1.5094244839768 1.13285987562353e-09 6.94207313408951e-09 102.272189482231 92.6479166760659 97.6752960039739 34.049622549715 30.6675306228598 38.5684701965642 up 96.0 90.0 92.0 36.0 37.0 36.0

SLC12A8 1.87934220141692 1.15057614247062e-09 7.04145948462874e-09 87.3574951827386 109.118657418478 64.7629680026348 33.1037997011118 21.5501566539015 16.0701959152351 up 82.0 106.0 61.0 35.0 26.0 15.0

KIAA1456 2.20728271551301 1.16441082136162e-09 7.12098170175665e-09 61.789447812181 61.7652777840439 50.9610240020733 15.1331655776511 9.11737396895832 13.9275031265371 up 58.0 60.0 48.0 16.0 11.0 13.0

GEM 1.43038990220783 1.1851538471657e-09 7.24260684379041e-09 122.513560317255 92.6479166760659 130.587624005313 39.7245596413342 46.4157220237878 41.7825093796112 up 115.0 90.0 123.0 42.0 56.0 39.0

MATN3 -1.30310981596071 1.18874823566077e-09 7.2619527991682e-09 56.4627712766481 47.3533796344337 52.0227120021165 137.144313047463 130.958644281401 115.705410589693 down 53.0 46.0 49.0 145.0 158.0 108.0

CD34 -4.54014712463447 1.38376745234493e-09 8.40481199093695e-09 0 0 5.30844000021597 44.4536738843501 45.5868698447916 32.1403918304702 down 0.0 0.0 5.0 47.0 55.0 30.0

NSUN6 1.05460058706218 1.39181159902648e-09 8.4476132165921e-09 205.609714271568 165.736828720518 190.042152007732 95.5281077089226 82.0563657206248 93.2071363083635 up 193.0 161.0 179.0 101.0 99.0 87.0

OSBP2 1.23629329792523 1.40841493560836e-09 8.53920866471208e-09 192.825690586289 201.766574094544 158.191512006436 76.6116507368587 56.3619481717423 103.920600251854 up 181.0 196.0 149.0 81.0 68.0 97.0

PPM1M 1.52831159885879 1.43289237205352e-09 8.68140085214116e-09 118.252219088829 114.265763900481 93.4285440038011 33.1037997011118 49.7311307397726 28.9263526474231 up 111.0 111.0 88.0 35.0 60.0 27.0

FTCD 1.39369319810275 1.48061811868428e-09 8.96093995373767e-09 138.493589923854 106.030393529275 133.772688005442 42.5620281871437 60.5062090667234 39.6398165909132 up 130.0 103.0 126.0 45.0 73.0 37.0

SLITRK2 -7.54078436049066 1.48425418265539e-09 8.97973780506511e-09 0 0 0 47.2911424301597 25.6944175488825 35.3544310135172 down 0.0 0.0 0.0 50.0 31.0 33.0

CP -4.46268968806966 1.49188372661199e-09 9.02267416237311e-09 5.32667653553284 10.2942129640073 0 109.715450437971 119.354713775454 116.776756984042 down 5.0 10.0 0.0 116.0 144.0 109.0

NAV2 1.12844314926708 1.59058301564917e-09 9.58537043226503e-09 235.439102870552 190.442939834135 170.931768006954 70.9367136452396 100.291113658541 101.777907463156 up 221.0 185.0 161.0 75.0 121.0 95.0

TTC39C-AS1 -2.37371243989826 1.6186292504986e-09 9.73348359266832e-09 7.45734714974598 14.4118981496103 9.55519200038875 55.8035480675885 48.9022785607764 58.9240516891953 down 7.0 14.0 9.0 59.0 59.0 55.0

FIGNL2 2.45302181972193 1.6207507234078e-09 9.73945273307136e-09 43.6787475913693 64.8535416732461 45.6525840018574 11.3498741832383 8.28852178996211 8.57077115479204 up 41.0 63.0 43.0 12.0 10.0 8.0

LOC654342 1.34185768489488 1.6933008794772e-09 1.0150218293823e-08 121.448225010149 109.118657418478 117.847368004795 49.1827881273661 48.0734263817802 39.6398165909132 up 114.0 106.0 111.0 52.0 58.0 37.0

AMPD3 2.63128564476256 1.73127737335712e-09 1.03631938885759e-08 47.9400888197956 47.3533796344337 43.529208001771 10.4040513346351 7.45966961096589 4.28538557739602 up 45.0 46.0 41.0 11.0 9.0 4.0

PLCH1 -2.07915350917643 1.83854326056062e-09 1.09975010006698e-08 11.7186883781723 18.5295833352132 24.4188240009935 82.2865878284779 51.3888350977651 98.5638682801085 down 11.0 18.0 23.0 87.0 62.0 92.0

DDR2 -2.41282877097513 1.86321126205823e-09 1.11371908183721e-08 10.6533530710657 5.14710648200366 15.9253200006479 58.6410166133981 62.1639134247158 47.1392413513562 down 10.0 5.0 15.0 62.0 75.0 44.0

H1FX-AS1 -2.43768945528869 2.09464222292553e-09 1.24677579959095e-08 10.6533530710657 10.2942129640073 9.55519200038875 61.4784851592076 65.4793221407006 37.4971238022152 down 10.0 10.0 9.0 65.0 79.0 35.0

PPP2R2B 4.89375262366319 2.15556091851497e-09 1.28168487046836e-08 44.7440828984759 30.882638892022 36.0973920014686 0 1.65770435799242 2.14269278869801 up 42.0 30.0 34.0 0.0 2.0 2.0

ACSM5 7.47560254987101 2.36735771800294e-09 1.40318543537333e-08 30.8947239060905 26.764953706419 35.0357040014254 0 0 0 up 29.0 26.0 33.0 0.0 0.0 0.0

GATA3 5.45137687571897 2.42733227675535e-09 1.43672287799566e-08 40.4827416700496 40.1474305596286 41.4058320016846 0 2.48655653698863 0 up 38.0 39.0 39.0 0.0 3.0 0.0

LOC100128076 2.26235443621414 2.43254523417227e-09 1.43930549307993e-08 52.2014300482219 52.5004861164373 60.5162160024621 7.56658278882556 15.748191400928 10.7134639434901 up 49.0 51.0 57.0 8.0 19.0 10.0

APOLD1 1.50135973655297 2.44019676823340e-09 1.44332867506542e-08 107.598866017763 83.3831250084593 118.909056004838 31.2121540039054 41.4426089498105 36.4257774078662 up 101.0 81.0 112.0 33.0 50.0 34.0

FBXO2 -1.26847119285183 2.47781111116411e-09 1.4645540901298e-08 41.5480769771562 71.0300694516505 70.0714080028508 147.548364382098 140.07601825036 153.202534391908 down 39.0 69.0 66.0 156.0 169.0 143.0

TMEM180 1.17484556128903 2.5497693747488e-09 1.50498582607124e-08 139.55892523096 183.23699075933 155.006448006306 74.7200050396524 58.8485047087309 79.2796331818264 up 131.0 178.0 146.0 79.0 71.0 74.0

TNFAIP8L3 -2.20172594650786 2.57562286903363e-09 1.51971617559839e-08 11.7186883781723 14.4118981496103 10.6168800004319 57.6951937647949 58.8485047087309 52.4959733231013 down 11.0 14.0 10.0 61.0 71.0 49.0

CBR3-AS1 1.51834114946588 2.62354433329187e-09 1.54691406216374e-08 98.0108482538043 88.530231490463 88.1201040035851 34.049622549715 29.8386784438636 32.1403918304702 up 92.0 86.0 83.0 36.0 36.0 30.0

HGD -1.07553720292175 2.83673257616204e-09 1.66797118501163e-08 94.8148423324846 70.0006481552498 104.045424004233 187.272924023432 202.239931675075 175.700808673237 down 89.0 68.0 98.0 198.0 244.0 164.0

MDGA2 2.11591427700361 2.97059612934449e-09 1.74124216024552e-08 63.9201184263941 58.6770138948417 58.3928400023757 9.45822848603194 19.8924522959091 11.7848103378391 up 60.0 57.0 55.0 10.0 24.0 11.0

CORO2B 1.18354926775899 3.41690238397e-09 1.99083167804375e-08 127.840236852788 135.883611124897 151.821384006177 56.7493709161917 62.992765603712 63.2094372665913 up 120.0 132.0 143.0 60.0 76.0 59.0

SELM -1.22078208122709 3.43295675749966e-09 1.99842912275066e-08 74.5734714974598 73.088912044452 47.7759600019437 166.464821354162 137.589461713371 152.131187997559 down 70.0 71.0 45.0 176.0 166.0 142.0

TCHH 1.80302710077654 3.45847552278826e-09 2.01259276386235e-08 70.3121302690335 66.9123842660476 83.8733520034124 17.0248112748575 26.5232697278787 19.2842350982821 up 66.0 65.0 79.0 18.0 32.0 18.0

CECR2 6.32799235392929 3.62976236643132e-09 2.10792507670026e-08 50.0707594340087 49.4122222272351 50.9610240020733 0.945822848603194 0.82885217899621 0 up 47.0 48.0 48.0 1.0 1.0 0.0

LINC00619 4.91170973233316 3.80421306464404e-09 2.20620915149161e-08 25.5680473705576 43.2356944488308 42.4675200017278 0 3.31540871598484 0 up 24.0 42.0 40.0 0.0 4.0 0.0

PCSK6 -2.71382260243995 3.91834892797415e-09 2.26851780040609e-08 9.58801776395912 4.11768518560293 8.49350400034555 65.2617765536204 42.2714611288067 37.4971238022152 down 9.0 4.0 8.0 69.0 51.0 35.0

POU3F3 6.31704901148944 4.02019222998419e-09 2.32554853439552e-08 39.417406362943 59.7064351912425 50.9610240020733 0.945822848603194 0 1.07134639434901 up 37.0 58.0 48.0 1.0 0.0 1.0

HHIP -3.72200374739276 4.02097500427656e-09 2.32554853439552e-08 0 4.11768518560293 4.24675200017278 37.8329139441278 35.6406436968371 37.4971238022152 down 0.0 4.0 4.0 40.0 43.0 35.0

NCKAP5 2.25468673819542 4.12981050311485e-09 2.38686420753209e-08 47.9400888197956 53.5299074128381 55.2077760022461 7.56658278882556 12.4327826849432 12.8561567321881 up 45.0 52.0 52.0 8.0 15.0 12.0

SFTA1P 1.51486024647076 4.34719906275045e-09 2.50993701850691e-08 96.9455129466977 91.6184953796652 106.168800004319 27.4288626094926 44.7580176657954 29.9976990417721 up 91.0 89.0 100.0 29.0 54.0 28.0

CYP4F35P 6.38301219881609 4.6486072536748e-09 2.67484398360228e-08 41.5480769771562 84.41254630486 31.8506400012958 0 0 2.14269278869801 up 39.0 82.0 30.0 0.0 0.0 2.0

USP18 -2.0243062501143 4.67553313012163e-09 2.68942381067098e-08 14.914694299492 10.2942129640073 25.4805120010367 58.6410166133981 67.137026498693 80.3509795761754 down 14.0 10.0 24.0 62.0 81.0 75.0

DLX3 1.47344868577872 4.87186712489406e-09 2.79760776595611e-08 103.337524789337 107.059814825676 100.860360004103 39.7245596413342 46.4157220237878 24.6409670700271 up 97.0 104.0 95.0 42.0 56.0 23.0

CRABP1 -5.30124859377436 5.1073709532272e-09 2.92491088396446e-08 2.13067061421314 0 1.06168800004319 48.2369652787629 39.7849045918181 36.4257774078662 down 2.0 0.0 1.0 51.0 48.0 34.0

SP5 2.16642559504516 5.16481228159244e-09 2.95580810980324e-08 43.6787475913693 70.0006481552498 59.4545280024189 9.45822848603194 13.2616348639394 16.0701959152351 up 41.0 68.0 56.0 10.0 16.0 15.0

FBLL1 1.38018270269478 5.25049768624747e-09 3.00383082759312e-08 111.86020724619 177.060462980926 130.587624005313 58.6410166133981 63.8216177827082 37.4971238022152 up 105.0 172.0 123.0 62.0 77.0 35.0

ZFPM2-AS1 -1.82847116803517 5.39139679552995e-09 3.08027864135842e-08 28.7640532918774 16.4707407424117 18.0486960007343 78.5032964340651 62.992765603712 83.5650187592224 down 27.0 16.0 17.0 83.0 76.0 78.0

PI16 -5.29038832866681 6.13264387678796e-09 3.48497105913412e-08 0 0 3.18506400012958 40.6703824899374 47.244574202784 35.3544310135172 down 0.0 0.0 3.0 43.0 57.0 33.0

FAS -1.78058182013889 6.5733869255864e-09 3.72792683359336e-08 19.1760355279182 18.5295833352132 25.4805120010367 67.1534222508268 68.7947308566855 81.4223259705244 down 18.0 18.0 24.0 71.0 83.0 76.0

NAP1L3 -1.24594749552735 6.70007503600133e-09 3.79596224447701e-08 50.0707594340087 50.4416435236359 52.0227120021165 124.848616015622 116.039305059469 121.062142561438 down 47.0 49.0 49.0 132.0 140.0 113.0

MEST -2.47778347569533 7.18965646265991e-09 4.06382317242105e-08 14.914694299492 9.26479166760659 3.18506400012958 48.2369652787629 54.7042438137499 49.2819341400542 down 14.0 9.0 3.0 51.0 66.0 46.0

CACNA1D 3.47552081866995 7.32176730190966e-09 4.13435793647832e-08 36.2214004416233 33.9709027812242 36.0973920014686 0.945822848603194 1.65770435799242 7.49942476044304 up 34.0 33.0 34.0 1.0 2.0 7.0

FAM181B 1.0667022838831 7.64776062092895e-09 4.30497294363638e-08 183.23767282233 239.855162061371 162.438264006609 101.203044800542 83.7140700786173 95.3498290970615 up 172.0 233.0 153.0 107.0 101.0 89.0

TMEM159 1.1260417340874 7.86617348323492e-09 4.42553898392559e-08 167.257643215731 134.854189828496 197.473968008034 70.9367136452396 73.7678439306627 84.6363651535714 up 157.0 131.0 186.0 75.0 89.0 79.0

SOCS2-AS1 1.71985857532606 7.88533430464874e-09 4.43484605314574e-08 63.9201184263941 89.5596527868637 71.133096002894 22.6997483664767 24.8655653698863 20.3555814926311 up 60.0 87.0 67.0 24.0 30.0 19.0

PLEKHG4B 2.73167456881774 7.99072738176262e-09 4.49262933445266e-08 39.417406362943 52.5004861164373 40.3441440016414 11.3498741832383 2.48655653698863 6.42807836609403 up 37.0 51.0 38.0 12.0 3.0 6.0

ZCCHC18 1.83811694807001 8.137235801673e-09 4.57196598608095e-08 66.0507890406073 64.8535416732461 63.7012800025917 21.7539255178735 16.5770435799242 16.0701959152351 up 62.0 63.0 60.0 23.0 20.0 15.0

ESYT3 1.32782172066988 8.30335126692251e-09 4.66066171178487e-08 108.66420132487 114.265763900481 109.353864004449 49.1827881273661 48.0734263817802 34.2830846191682 up 102.0 111.0 103.0 52.0 58.0 32.0

SDR42E1 -7.23327266089971 8.39927789165343e-09 4.71138302929169e-08 0 0 0 27.4288626094926 33.9829393388446 25.7123134643761 down 0.0 0.0 0.0 29.0 41.0 24.0

LINC00840 4.11767477926189 8.539063743208e-09 4.78345700429708e-08 41.5480769771562 28.8237962992205 28.6655760011662 0.945822848603194 0.82885217899621 4.28538557739602 up 39.0 28.0 27.0 1.0 1.0 4.0

ERBB3 -2.220512725915 9.45137689487539e-09 5.28752723246992e-08 12.7840236852788 6.17652777840439 19.1103840007775 69.9908907966364 47.244574202784 59.9953980835443 down 12.0 6.0 18.0 74.0 57.0 56.0

KIF26A -1.16643728218907 9.71101319103998e-09 5.42203571048837e-08 67.1161243477138 70.0006481552498 55.2077760022461 163.627352808353 129.300939923409 139.275031265371 down 63.0 68.0 52.0 173.0 156.0 130.0

PRKCQ-AS1 7.26619956296574 1.0204707915966e-08 5.69393122847379e-08 27.6987179847708 18.5295833352132 33.9740160013822 0 0 0 up 26.0 18.0 32.0 0.0 0.0 0.0

ARX 1.92947728914401 1.08481449046747e-08 6.03505992397995e-08 50.0707594340087 70.0006481552498 76.44153600311 22.6997483664767 14.9193392219318 13.9275031265371 up 47.0 68.0 72.0 24.0 18.0 13.0

NOL4 7.27956924997623 1.13722678107862e-08 6.30799661803271e-08 34.0907298274102 31.9120601884227 14.8636320006047 0 0 0 up 32.0 31.0 14.0 0.0 0.0 0.0

RXFP1 -7.18093201017966 1.14439628275692e-08 6.34361028465385e-08 0 0 0 25.5372169122862 25.6944175488825 33.2117382248192 down 0.0 0.0 0.0 27.0 31.0 31.0

LOC101927181 1.26498557669747 1.15249471437669e-08 6.3864116655352e-08 112.925542553296 127.648240753691 108.292176004406 51.0744338245725 42.2714611288067 52.4959733231013 up 106.0 124.0 102.0 54.0 51.0 49.0

AHRR -1.00086991554193 1.2364799877366e-08 6.82279185415572e-08 89.4881657969518 128.677662050092 102.98373600419 248.75140918264 201.411079496079 192.842350982821 down 84.0 125.0 97.0 263.0 243.0 180.0

HIST1H2AC 1.17730626214446 1.26280398478735e-08 6.95897836769604e-08 138.493589923854 131.765925939294 118.909056004838 63.370130856414 52.2176872767613 56.7813589004973 up 130.0 128.0 112.0 67.0 63.0 53.0

ZNF385D -4.64922999852868 1.35647316737586e-08 7.46060242056724e-08 2.13067061421314 1.02942129640073 1.06168800004319 28.3746854580958 35.6406436968371 41.7825093796112 down 2.0 1.0 1.0 30.0 43.0 39.0

DMRTA1 2.83101833136625 1.41867146110337e-08 7.78752253761861e-08 42.6134122842627 36.0297453740256 35.0357040014254 7.56658278882556 4.14426089498105 4.28538557739602 up 40.0 35.0 33.0 8.0 5.0 4.0

PTPN20 -7.14607761749578 1.53936973103979e-08 8.42550024032762e-08 0 0 0 24.5913940636831 34.8117915178408 22.4982742813291 down 0.0 0.0 0.0 26.0 42.0 21.0

KRT34 1.95008681599948 1.66667424722124e-08 9.0958317486881e-08 68.1814596548204 49.4122222272351 69.0097200028076 13.2415198804447 21.5501566539015 12.8561567321881 up 64.0 48.0 65.0 14.0 26.0 12.0

ANKEF1 1.10531779551485 1.6732048073096e-08 9.12853121926718e-08 156.604290144666 127.648240753691 143.327880005831 64.3159537050172 68.7947308566855 65.3521300552893 up 147.0 124.0 135.0 68.0 83.0 61.0

SLC47A1 -1.67016878543367 1.68622426450272e-08 9.19659981992147e-08 24.5027120634511 19.5590046316139 25.4805120010367 79.4491192826683 72.9389917516665 68.5661692383363 down 23.0 19.0 24.0 84.0 88.0 64.0

ANOS1 -7.11108282113725 1.71961951382499e-08 9.36667349331039e-08 0 0 0 29.320508306699 23.2078610118939 27.8550062530741 down 0.0 0.0 0.0 31.0 28.0 26.0

IL31RA 5.22611024454269 1.73586386870295e-08 9.44604366714679e-08 37.2867357487299 23.6766898172168 44.5908960018142 0.945822848603194 0.82885217899621 1.07134639434901 up 35.0 23.0 42.0 1.0 1.0 1.0

VASN -1.09772900190017 1.75674133287863e-08 9.55044870955197e-08 64.9854537335007 89.5596527868637 84.9350400034556 161.735707111146 149.193392219318 203.555814926311 down 61.0 87.0 80.0 171.0 180.0 190.0

ADTRP -1.63441398242965 1.8993547387986e-08 1.02993179498234e-07 17.0453649137051 36.0297453740256 29.7272640012094 95.5281077089226 88.6871831525945 72.8515548157324 down 16.0 35.0 28.0 101.0 107.0 68.0

CLDN4 1.20218680683432 1.94355384280447e-08 1.05187866124945e-07 107.598866017763 137.942453717698 141.204504005745 57.6951937647949 60.5062090667234 49.2819341400542 up 101.0 134.0 133.0 61.0 73.0 46.0

SLC19A3 2.88922290593127 2.00776970014908e-08 1.08351764002948e-07 27.6987179847708 47.3533796344337 41.4058320016846 3.78329139441278 8.28852178996211 3.21403918304702 up 26.0 46.0 39.0 4.0 10.0 3.0

PID1 1.03672570895504 2.05527321072958e-08 1.10809446816547e-07 168.322978522838 172.942777795323 175.178520007127 79.4491192826683 101.948818016534 68.5661692383363 up 158.0 168.0 165.0 84.0 123.0 64.0

PSG8 4.68370052060542 2.06174926725261e-08 1.11123234448804e-07 31.9600592131971 41.1768518560293 23.3571360009503 0 1.65770435799242 2.14269278869801 up 30.0 40.0 22.0 0.0 2.0 2.0

FUZ -1.51090072667655 2.13256858119073e-08 1.14757661262296e-07 35.1560651345168 42.20627315243 24.4188240009935 116.336210378193 81.2275135416286 93.2071363083635 down 33.0 41.0 23.0 123.0 98.0 87.0

CPEB1 1.36248547640954 2.24203139773993e-08 1.20189911005425e-07 96.9455129466977 111.177500011279 129.52593600527 57.6951937647949 38.1272002338257 35.3544310135172 up 91.0 108.0 122.0 61.0 46.0 33.0

TMEM130 1.63973393024595 2.2550215765182e-08 1.20848040196831e-07 82.0308186472058 67.9418055624483 79.6266000032396 30.2663311553022 25.6944175488825 17.1415423095841 up 77.0 66.0 75.0 32.0 31.0 16.0

ASPHD1 1.41918251792266 2.30280970139029e-08 1.23214138792014e-07 91.6188364111649 96.7656018616688 87.0584160035419 40.6703824899374 33.1540871598484 28.9263526474231 up 86.0 94.0 82.0 43.0 40.0 27.0

CCDC184 -1.23401529308092 2.46391574016706e-08 1.31543437246864e-07 67.1161243477138 60.7358564876432 39.2824560015982 140.927604441876 130.958644281401 121.062142561438 down 63.0 59.0 37.0 149.0 158.0 113.0

ATP2A1-AS1 -2.55394475361222 2.48251442033375e-08 1.32452895371508e-07 14.914694299492 10.2942129640073 1.06168800004319 54.8577252189853 38.9560524128219 61.0667444778933 down 14.0 10.0 1.0 58.0 47.0 57.0

LINC00704 -5.11048926893872 2.65797033793867e-08 1.41591250077614e-07 1.06533530710657 2.05884259280146 0 41.6162053385406 38.1272002338257 28.9263526474231 down 1.0 2.0 0.0 44.0 46.0 27.0

PF4V1 -5.12437220547621 2.68921985505783e-08 1.43120905889663e-07 0 3.0882638892022 0 42.5620281871437 38.1272002338257 28.9263526474231 down 0.0 3.0 0.0 45.0 46.0 27.0

PRIMA1 -1.66792359507766 2.82029156741743e-08 1.49908186859276e-07 26.6333826776642 16.4707407424117 28.6655760011662 70.9367136452396 70.4524352146779 86.7790579422694 down 25.0 16.0 27.0 75.0 85.0 81.0

C11orf74 1.03279944505618 2.82543401890677e-08 1.50134417441282e-07 217.32840264974 158.530879645713 228.262920009287 114.444564680987 99.4622614795453 80.3509795761754 up 204.0 154.0 215.0 121.0 120.0 75.0

FLRT3 -1.09795416855974 2.93502447714439e-08 1.55567317405588e-07 82.0308186472058 57.647592598441 74.3181600030236 139.981781593273 169.085844515227 147.845802420163 down 77.0 56.0 70.0 148.0 204.0 138.0

ROPN1L 1.31037715546447 2.96900160944103e-08 1.57269816334994e-07 99.0761835609109 94.7067592688674 107.230488004363 42.5620281871437 37.2983480548295 41.7825093796112 up 93.0 92.0 101.0 45.0 45.0 39.0

CFAP47 -1.49071028824537 2.97918006567018e-08 1.57710344726415e-07 39.417406362943 24.7061111136176 29.7272640012094 94.5822848603194 86.2006266156059 82.4936723648734 down 37.0 24.0 28.0 100.0 104.0 77.0

LINC01589 -2.50161058456451 3.19540317485082e-08 1.68262759657982e-07 7.45734714974598 3.0882638892022 13.8019440005615 40.6703824899374 46.4157220237878 50.3532805344032 down 7.0 3.0 13.0 43.0 56.0 47.0

SP8 6.06570845922714 3.30483062723171e-08 1.73700995424465e-07 26.6333826776642 56.6181713020403 41.4058320016846 0 1.65770435799242 0 up 25.0 55.0 39.0 0.0 2.0 0.0

ITIH5 5.12431253393362 3.80250191324164e-08 1.98994076028154e-07 41.5480769771562 29.8532175956212 26.5422000010799 1.89164569720639 0.82885217899621 0 up 39.0 29.0 25.0 2.0 1.0 0.0

ACVRL1 -4.21811338723009 3.97485015167655e-08 2.07628620318843e-07 0 4.11768518560293 1.06168800004319 38.778736792731 33.9829393388446 24.6409670700271 down 0.0 4.0 1.0 41.0 41.0 23.0

MTMR8 -6.99960794481288 3.98119058542952e-08 2.07895710595487e-07 0 0 0 34.049622549715 19.8924522959091 20.3555814926311 down 0.0 0.0 0.0 36.0 24.0 19.0

SERTAD4-AS1 -1.1420703060531 4.00051632346557e-08 2.08776175352763e-07 61.789447812181 56.6181713020403 58.3928400023757 138.090135896066 137.589461713371 113.562717800995 down 58.0 55.0 55.0 146.0 166.0 106.0

PDZK1P1 1.24778601826726 4.14994695987811e-08 2.16374581410696e-07 131.036242774108 110.148078714878 105.107112004276 39.7245596413342 56.3619481717423 49.2819341400542 up 123.0 107.0 99.0 42.0 68.0 46.0

ADAM11 -1.03899917375417 4.32733949334201e-08 2.25000402140005e-07 63.9201184263941 80.2948611192571 77.5032240031532 160.789884262543 149.193392219318 145.703109631465 down 60.0 78.0 73.0 170.0 180.0 136.0

KCNQ5 4.64618532856518 4.40365684405514e-08 2.28757886961957e-07 38.3520710558365 19.5590046316139 35.0357040014254 0.945822848603194 2.48655653698863 0 up 36.0 19.0 33.0 1.0 3.0 0.0

ZMIZ1-AS1 2.29889973498557 4.57750099974758e-08 2.37497295362095e-07 47.9400888197956 39.1180092632278 45.6525840018574 8.51240563742875 11.6039305059469 6.42807836609403 up 45.0 38.0 43.0 9.0 14.0 6.0

MYO5B -1.40761975220645 4.64580202465055e-08 2.40672435160796e-07 33.0253945203036 27.7943750028198 47.7759600019437 97.419753406129 92.0025918685794 98.5638682801085 down 31.0 27.0 45.0 103.0 111.0 92.0

MAST4 -1.66724512053753 4.79911848953602e-08 2.48235319733558e-07 17.0453649137051 28.8237962992205 22.2954480009071 79.4491192826683 63.8216177827082 73.9229012100814 down 16.0 28.0 21.0 84.0 77.0 69.0

DPY19L2P1 5.971402231924 5.18134316766029e-08 2.67027542622955e-07 31.9600592131971 32.9414814848234 52.0227120021165 0 1.65770435799242 0 up 30.0 32.0 49.0 0.0 2.0 0.0

GUCY1A3 1.17037527404952 5.22284309582623e-08 2.68920857274457e-07 115.056213167509 123.530555568088 131.649312005356 51.0744338245725 48.0734263817802 66.4234764496383 up 108.0 120.0 124.0 54.0 58.0 62.0

DLGAP1 -2.44626895943162 5.36089333611436e-08 2.75861279203454e-07 5.32667653553284 7.20594907480513 10.6168800004319 42.5620281871437 39.7849045918181 43.9252021683092 down 5.0 7.0 10.0 45.0 48.0 41.0

EDNRA -4.50534885712138 5.65475471786708e-08 2.9045344123914e-07 1.06533530710657 2.05884259280146 1.06168800004319 23.6455712150799 36.4694958758333 35.3544310135172 down 1.0 2.0 1.0 25.0 44.0 33.0

FAM20A -2.17295184925497 5.78204566966666e-08 2.9663190079998e-07 14.914694299492 8.23537037120586 9.55519200038875 52.0202566731757 53.0465394557575 41.7825093796112 down 14.0 8.0 9.0 55.0 64.0 39.0

ATL1 1.10946574350301 6.42837747950983e-08 3.28298807666254e-07 123.578895624362 127.648240753691 124.217496005054 54.8577252189853 56.3619481717423 63.2094372665913 up 116.0 124.0 117.0 58.0 68.0 59.0

LINGO2 3.06755374650283 6.69849510039892e-08 3.4157888922564e-07 21.3067061421314 42.20627315243 46.7142720019006 8.51240563742875 2.48655653698863 2.14269278869801 up 20.0 41.0 44.0 9.0 3.0 2.0

KRT16 -5.85028277775743 6.79575461706077e-08 3.46039853846706e-07 0 2.05884259280146 0 45.3994967329533 37.2983480548295 38.5684701965642 down 0.0 2.0 0.0 48.0 45.0 36.0

FRG1JP -1.86974957793885 6.79619004573785e-08 3.46039853846706e-07 15.9800296065985 15.441319446011 15.9253200006479 52.9660795217789 53.0465394557575 67.4948228439873 down 15.0 15.0 15.0 56.0 64.0 63.0

TPTE2P1 3.05002883575726 6.8615809349496e-08 3.49264366099899e-07 34.0907298274102 27.7943750028198 41.4058320016846 4.72911424301597 0.82885217899621 7.49942476044304 up 32.0 27.0 39.0 5.0 1.0 7.0

CHRM3 3.80326044203518 7.24932534997229e-08 3.68226600985099e-07 21.3067061421314 36.0297453740256 35.0357040014254 5.67493709161917 0 1.07134639434901 up 20.0 35.0 33.0 6.0 0.0 1.0

IQGAP2 2.45244721655402 7.25157432319722e-08 3.68230422766669e-07 49.0054241269022 40.1474305596286 32.912328001339 6.62075994022236 9.94622614795453 5.35673197174503 up 46.0 39.0 31.0 7.0 12.0 5.0

GPR173 -6.65623159782522 7.32639246591149e-08 3.71806735687659e-07 1.06533530710657 0 0 34.049622549715 37.2983480548295 42.8538557739602 down 1.0 0.0 0.0 36.0 45.0 40.0

NIPSNAP3B 1.1082269505527 7.50189527988109e-08 3.80371463756916e-07 142.75493115228 122.501134271687 146.512944005961 55.8035480675885 76.2544004676514 57.8527052948463 up 134.0 119.0 138.0 59.0 92.0 54.0

MTMR7 1.3872913127312 7.55609627410572e-08 3.82547133542591e-07 105.46819540355 97.7950231580696 94.4902320038443 24.5913940636831 48.0734263817802 40.7111629852622 up 99.0 95.0 89.0 26.0 58.0 38.0

PAMR1 3.97460800199965 7.61395836653949e-08 3.85361382519208e-07 25.5680473705576 39.1180092632278 24.4188240009935 4.72911424301597 0 1.07134639434901 up 24.0 38.0 23.0 5.0 0.0 1.0

PCDH1 -1.70343215185727 7.83469541568999e-08 3.95587899677462e-07 20.2413708350248 23.6766898172168 19.1103840007775 84.1782335256843 57.1908003507385 64.2807836609403 down 19.0 23.0 18.0 89.0 69.0 60.0

CALCB 4.51306985457556 7.90266940848863e-08 3.98901131644212e-07 29.8293885989839 26.764953706419 30.7889520012526 0 0 4.28538557739602 up 28.0 26.0 29.0 0.0 0.0 4.0

MAP3K7CL 1.15345495778996 8.1720630486342e-08 4.11763081629576e-07 104.402860096444 125.589398160889 130.587624005313 57.6951937647949 53.8753916347537 50.3532805344032 up 98.0 122.0 123.0 61.0 65.0 47.0

ZKSCAN7 2.27459714498567 8.52537914057021e-08 4.29055028643076e-07 38.3520710558365 43.2356944488308 46.7142720019006 8.51240563742875 6.63081743196968 11.7848103378391 up 36.0 42.0 44.0 9.0 8.0 11.0

SPANXD -4.09752021334764 8.97305121027807e-08 4.50479476437627e-07 0 3.0882638892022 2.12337600008639 28.3746854580958 31.496382801856 29.9976990417721 down 0.0 3.0 2.0 30.0 38.0 28.0

ZNF716 -6.87799512306836 9.03570486100916e-08 4.53391114767462e-07 0 0 0 26.4830397608894 13.2616348639394 28.9263526474231 down 0.0 0.0 0.0 28.0 16.0 27.0

GALNT18 1.9981795156583 9.43078466338601e-08 4.72375790058424e-07 45.8094182055824 46.323958338033 57.3311520023325 13.2415198804447 13.2616348639394 10.7134639434901 up 43.0 45.0 54.0 14.0 16.0 10.0

C15orf59 -1.22712173275672 9.91165738647503e-08 4.95290490049814e-07 36.2214004416233 54.5593287092388 53.0844000021597 118.227856075399 113.552748522481 104.991946646203 down 34.0 53.0 50.0 125.0 137.0 98.0

ZNF503-AS2 1.38403538385306 1.13872831697944e-07 5.66188954788134e-07 79.9001480329926 86.4713888976615 85.9967280034987 36.8870910955246 29.8386784438636 29.9976990417721 up 75.0 84.0 81.0 39.0 36.0 28.0

ANO3 5.81196723970636 1.15333114921519e-07 5.73113219938556e-07 27.6987179847708 38.0885879668271 39.2824560015982 0.945822848603194 0.82885217899621 0 up 26.0 37.0 37.0 1.0 1.0 0.0

CCDC74A 1.18792155594009 1.16121576445688e-07 5.76862024923742e-07 111.86020724619 107.059814825676 119.970744004881 58.6410166133981 48.9022785607764 40.7111629852622 up 105.0 104.0 113.0 62.0 59.0 38.0

ANKRD30B 5.06021553855119 1.17262463101034e-07 5.82017616446388e-07 52.2014300482219 17.5001620388124 25.4805120010367 0.945822848603194 0 2.14269278869801 up 49.0 17.0 24.0 1.0 0.0 2.0

SH3BP5 1.01323311178036 1.20385671013769e-07 5.96396854001477e-07 138.493589923854 164.707407424117 136.957752005572 76.6116507368587 67.9658786776893 73.9229012100814 up 130.0 160.0 129.0 81.0 82.0 69.0

TLR4 6.59425112786267 1.2613255321114e-07 6.24214271515252e-07 37.2867357487299 26.764953706419 33.9740160013822 0.945822848603194 0 0 up 35.0 26.0 32.0 1.0 0.0 0.0

GNG3 -1.25474771086804 1.26402847607907e-07 6.25369228527439e-07 49.0054241269022 54.5593287092388 32.912328001339 102.148867649145 114.381600701477 109.277332223599 down 46.0 53.0 31.0 108.0 138.0 102.0

C19orf81 1.22474360197576 1.27561390149495e-07 6.30732617960432e-07 108.66420132487 91.6184953796652 123.155808005011 44.4536738843501 50.5599829187688 42.8538557739602 up 102.0 89.0 116.0 47.0 61.0 40.0

ENG 1.01932293825993 1.31752318230884e-07 6.50884885048461e-07 145.9509370736 180.148726870128 148.636320006047 87.9615249200971 63.8216177827082 83.5650187592224 up 137.0 175.0 140.0 93.0 77.0 78.0

SLC6A16 -2.37325600051956 1.43744023092033e-07 7.07238963456009e-07 4.26134122842627 10.2942129640073 9.55519200038875 35.9412682469214 42.2714611288067 47.1392413513562 down 4.0 10.0 9.0 38.0 51.0 44.0

FGF14-AS2 3.40902788563068 1.45294823489729e-07 7.14454081252833e-07 19.1760355279182 35.0003240776249 35.0357040014254 0.945822848603194 4.14426089498105 3.21403918304702 up 18.0 34.0 33.0 1.0 5.0 3.0

DNAH6 2.05785345642465 1.45543849147271e-07 7.15470924130808e-07 55.3974359695416 68.9712268588491 36.0973920014686 17.9706341234607 9.94622614795453 10.7134639434901 up 52.0 67.0 34.0 19.0 12.0 10.0

CYTH4 2.3298156489373 1.46376384506469e-07 7.19146158219136e-07 50.0707594340087 39.1180092632278 33.9740160013822 5.67493709161917 8.28852178996211 10.7134639434901 up 47.0 38.0 32.0 6.0 10.0 10.0

MMP10 -6.77286312004901 1.47245849630701e-07 7.2320808719747e-07 0 0 0 18.9164569720639 28.1809740858712 16.0701959152351 down 0.0 0.0 0.0 20.0 34.0 15.0

NR3C2 -1.67918627945811 1.47607481291442e-07 7.24774125529573e-07 19.1760355279182 16.4707407424117 24.4188240009935 59.5868394620012 62.1639134247158 70.7088620270343 down 18.0 16.0 23.0 63.0 75.0 66.0

GBP1 -1.02054470988766 1.50945646898716e-07 7.40306675872685e-07 80.9654833400992 65.8829629696469 75.3798480030668 129.577730258638 158.310766188276 162.844651941049 down 76.0 64.0 71.0 137.0 191.0 152.0

CPNE7 -1.74309731529558 1.51995669278163e-07 7.44809556717407e-07 17.0453649137051 17.5001620388124 18.0486960007343 56.7493709161917 61.3350612457196 57.8527052948463 down 16.0 17.0 17.0 60.0 74.0 54.0

HPGD 1.68290365628093 1.53657251223525e-07 7.52516286709023e-07 55.3974359695416 62.7946990804447 79.6266000032396 18.9164569720639 16.5770435799242 26.7836598587251 up 52.0 61.0 75.0 20.0 20.0 25.0

CYP26A1 1.96991466735193 1.54232756156557e-07 7.55116441991929e-07 43.6787475913693 45.2945370416322 64.7629680026348 14.1873427290479 14.0904870429356 10.7134639434901 up 41.0 44.0 61.0 15.0 17.0 10.0

MUC16 -4.05256704814629 1.58921976379186e-07 7.77176177789666e-07 3.19600592131971 2.05884259280146 0 30.2663311553022 33.1540871598484 23.5696206756781 down 3.0 2.0 0.0 32.0 40.0 22.0

SYT12 1.68027171496718 1.61886326463892e-07 7.90759622346693e-07 56.4627712766481 64.8535416732461 61.5779040025053 16.0789884262543 21.5501566539015 19.2842350982821 up 53.0 63.0 58.0 17.0 26.0 18.0

MPPED2 -6.72303521874427 1.67264435585408e-07 8.16088576848159e-07 0 0 0 19.8622798206671 19.0636001169128 22.4982742813291 down 0.0 0.0 0.0 21.0 23.0 21.0

RASL11A 1.20503296555512 1.7022811683406e-07 8.30070322155722e-07 109.729536631977 91.6184953796652 108.292176004406 45.3994967329533 43.9291654867992 44.9965485626582 up 103.0 89.0 102.0 48.0 53.0 42.0

FLJ31356 1.71531640245165 1.73557516188748e-07 8.45331893110232e-07 87.3574951827386 75.1477546372535 46.7142720019006 16.0789884262543 24.0367131908901 23.5696206756781 up 82.0 73.0 44.0 17.0 29.0 22.0

BCL11A -1.69020683362236 1.83192680751605e-07 8.89447982783662e-07 19.1760355279182 16.4707407424117 29.7272640012094 80.3949421312715 77.0832526466476 52.4959733231013 down 18.0 16.0 28.0 85.0 93.0 49.0

SATB1-AS1 2.20588282636223 1.85081460414741e-07 8.98361014162098e-07 49.0054241269022 45.2945370416322 36.0973920014686 5.67493709161917 9.94622614795453 12.8561567321881 up 46.0 44.0 34.0 6.0 12.0 12.0

EREG -5.6783870460975 1.88226512212355e-07 9.13103412622362e-07 0 0 2.12337600008639 34.049622549715 37.2983480548295 36.4257774078662 down 0.0 0.0 2.0 36.0 45.0 34.0

NRARP -1.10406088665933 1.94183455510945e-07 9.39579473394863e-07 53.2667653553284 60.7358564876432 56.2694640022893 120.119501772606 111.895044164488 134.989645687975 down 50.0 59.0 53.0 127.0 135.0 126.0

ARHGAP4 1.442221308263 1.95066039187914e-07 9.43531433018538e-07 88.4228304898452 119.412870382485 73.2564720029804 43.5078510357469 34.8117915178408 24.6409670700271 up 83.0 116.0 69.0 46.0 42.0 23.0

CABP4 2.00471784216501 1.99694298389355e-07 9.64866347608575e-07 49.0054241269022 62.7946990804447 40.3441440016414 15.1331655776511 8.28852178996211 14.9988495208861 up 46.0 61.0 38.0 16.0 10.0 14.0

GPM6B -1.91419090118411 2.05679662411116e-07 9.92195330983053e-07 21.3067061421314 10.2942129640073 13.8019440005615 64.3159537050172 62.992765603712 42.8538557739602 down 20.0 10.0 13.0 68.0 76.0 40.0

POSTN -2.57289603651146 2.11914224884768e-07 1.01925808334695e-06 12.7840236852788 5.14710648200366 2.12337600008639 34.9954453983182 42.2714611288067 41.7825093796112 down 12.0 5.0 2.0 37.0 51.0 39.0

CT45A1 6.74557984535247 2.21994965696615e-07 1.06502257686227e-06 24.5027120634511 14.4118981496103 16.9870080006911 0 0 0 up 23.0 14.0 16.0 0.0 0.0 0.0

FAM122C 1.31856366281612 2.24861425325323e-07 1.07785867148018e-06 104.402860096444 108.089236122077 79.6266000032396 49.1827881273661 35.6406436968371 32.1403918304702 up 98.0 105.0 75.0 52.0 43.0 30.0

DACH1 6.71414780665364 2.36910967124753e-07 1.13273265116943e-06 17.0453649137051 19.5590046316139 18.0486960007343 0 0 0 up 16.0 19.0 17.0 0.0 0.0 0.0

BZRAP1 2.86156520716076 2.50292377173274e-07 1.19536308692283e-06 23.4373767563445 40.1474305596286 31.8506400012958 2.83746854580958 4.97311307397726 5.35673197174503 up 22.0 39.0 30.0 3.0 6.0 5.0

DACT2 -6.6971458593099 2.61594905250627e-07 1.24547995923148e-06 0 0 0 17.9706341234607 29.0098262648674 12.8561567321881 down 0.0 0.0 0.0 19.0 35.0 12.0

HOXA11 2.40062652213051 2.62279979658577e-07 1.24839079949882e-06 37.2867357487299 48.3828009308344 28.6655760011662 6.62075994022236 6.63081743196968 8.57077115479204 up 35.0 47.0 27.0 7.0 8.0 8.0

RHOV -1.36020300621166 2.86882095023445e-07 1.36014069121107e-06 24.5027120634511 50.4416435236359 48.8376480019869 99.3113991033354 126.81438338642 91.0644435196654 down 23.0 49.0 46.0 105.0 153.0 85.0

PLVAP -6.67538720758655 3.04262267807307e-07 1.43931941263775e-06 0 0 0 30.2663311553022 14.0904870429356 14.9988495208861 down 0.0 0.0 0.0 32.0 17.0 14.0

FAHD2CP 2.03919035561592 3.15181686420409e-07 1.48848000221961e-06 41.5480769771562 49.4122222272351 52.0227120021165 17.9706341234607 8.28852178996211 8.57077115479204 up 39.0 48.0 49.0 19.0 10.0 8.0

LINCR-0002 -6.37994561762168 3.18012174681744e-07 1.50059226716121e-06 0 0 1.06168800004319 30.2663311553022 34.8117915178408 28.9263526474231 down 0.0 0.0 1.0 32.0 42.0 27.0

RNASE4 -1.22056404704635 3.38873161912005e-07 1.59370110016362e-06 43.6787475913693 43.2356944488308 48.8376480019869 84.1782335256843 114.381600701477 117.848103378391 down 41.0 42.0 46.0 89.0 138.0 110.0

GABRG3 -6.63502679250652 3.47435601443498e-07 1.63170476530437e-06 0 0 0 17.9706341234607 26.5232697278787 12.8561567321881 down 0.0 0.0 0.0 19.0 32.0 12.0

CAMK2N2 1.23924434830853 3.64978890039166e-07 1.71077542813046e-06 88.4228304898452 108.089236122077 109.353864004449 32.1579768525086 47.244574202784 50.3532805344032 up 83.0 105.0 103.0 34.0 57.0 47.0

NRSN2-AS1 1.02969622849992 3.88917927052567e-07 1.81745370600565e-06 187.499014050756 126.61881945729 141.204504005745 66.2075994022236 74.596696109659 82.4936723648734 up 176.0 123.0 133.0 70.0 90.0 77.0

RASGEF1C 1.50594166127394 3.91409839531534e-07 1.82809006938632e-06 69.246794961927 95.7361805652681 59.4545280024189 29.320508306699 23.2078610118939 26.7836598587251 up 65.0 93.0 56.0 31.0 28.0 25.0

OXCT2 2.20931677407838 4.15277138401804e-07 1.93529428460153e-06 38.3520710558365 50.4416435236359 32.912328001339 6.62075994022236 9.11737396895832 10.7134639434901 up 36.0 49.0 31.0 7.0 11.0 10.0

LOC101929723 3.12484393473784 4.30285343015001e-07 2.00358265823918e-06 28.7640532918774 26.764953706419 26.5422000010799 1.89164569720639 3.31540871598484 4.28538557739602 up 27.0 26.0 25.0 2.0 4.0 4.0

C2CD4C -1.82405141853233 4.49091087941999e-07 2.08656144534763e-06 11.7186883781723 24.7061111136176 12.7402560005183 70.9367136452396 52.2176872767613 51.4246269287523 down 11.0 24.0 12.0 75.0 63.0 48.0

LOC100506100 1.10594576939418 4.65754168674351e-07 2.16101769853287e-06 125.709566238575 101.912708343672 114.662304004665 57.6951937647949 54.7042438137499 46.0678949570072 up 118.0 99.0 108.0 61.0 66.0 43.0

MAGEL2 6.6021464808885 4.74033280772608e-07 2.19762555453968e-06 19.1760355279182 13.3824768532095 18.0486960007343 0 0 0 up 18.0 13.0 17.0 0.0 0.0 0.0

ATG4A 1.05859054375775 4.8412200146018e-07 2.24071767888947e-06 128.905572159895 118.383449086084 156.06813600635 58.6410166133981 77.0832526466476 56.7813589004973 up 121.0 115.0 147.0 62.0 93.0 53.0

LRRC7 6.63400817270684 4.92262864826275e-07 2.27466801149948e-06 25.5680473705576 11.3236342604081 14.8636320006047 0 0 0 up 24.0 11.0 14.0 0.0 0.0 0.0

NPR1 -1.96188606011266 5.03585121525425e-07 2.32445012497022e-06 12.7840236852788 15.441319446011 10.6168800004319 36.8870910955246 49.7311307397726 65.3521300552893 down 12.0 15.0 10.0 39.0 60.0 61.0

NCAN -3.39295795582468 5.38592398483224e-07 2.47860777786086e-06 2.13067061421314 1.02942129640073 5.30844000021597 23.6455712150799 21.5501566539015 43.9252021683092 down 2.0 1.0 5.0 25.0 26.0 41.0

C5 1.68688751533373 5.50978918348564e-07 2.5328583112143e-06 57.5281065837547 74.1183333408527 52.0227120021165 17.0248112748575 14.0904870429356 26.7836598587251 up 54.0 72.0 49.0 18.0 17.0 25.0

SYTL1 -1.71795653862121 5.86061622740275e-07 2.68611577089293e-06 17.0453649137051 17.5001620388124 15.9253200006479 60.5326623106044 58.0196525297347 47.1392413513562 down 16.0 17.0 15.0 64.0 70.0 44.0

KRT222 5.54786571462655 5.86416936785808e-07 2.68701728675997e-06 30.8947239060905 22.6472685208161 33.9740160013822 0.945822848603194 0.82885217899621 0 up 29.0 22.0 32.0 1.0 1.0 0.0

RASGRP3 -2.03152502047064 5.88429824989234e-07 2.69478270757438e-06 10.6533530710657 11.3236342604081 10.6168800004319 37.8329139441278 42.2714611288067 53.5673197174503 down 10.0 11.0 10.0 40.0 51.0 50.0

GNG2 1.25035079500043 5.93779708461768e-07 2.71707948712651e-06 95.8801776395912 96.7656018616688 99.7986720040603 29.320508306699 52.2176872767613 40.7111629852622 up 90.0 94.0 94.0 31.0 63.0 38.0

PLEKHB1 2.17777946987675 6.39073185759813e-07 2.91724596248214e-06 50.0707594340087 27.7943750028198 48.8376480019869 11.3498741832383 9.94622614795453 6.42807836609403 up 47.0 27.0 46.0 12.0 12.0 6.0

NPY1R 2.62737624992347 6.4246169596625e-07 2.93192379570805e-06 35.1560651345168 28.8237962992205 28.6655760011662 5.67493709161917 4.97311307397726 4.28538557739602 up 33.0 28.0 27.0 6.0 6.0 4.0

LYPD6B 2.11036426466842 6.51861308899888e-07 2.9740184682909e-06 33.0253945203036 58.6770138948417 48.8376480019869 10.4040513346351 5.80196525297347 17.1415423095841 up 31.0 57.0 46.0 11.0 7.0 16.0

LOC284930 5.49144055352696 6.76170606856603e-07 3.0799489325493e-06 30.8947239060905 25.7355324100183 28.6655760011662 0 0 2.14269278869801 up 29.0 25.0 27.0 0.0 0.0 2.0

PNMT -4.25087866318812 7.11277598489672e-07 3.23030630520511e-06 1.06533530710657 3.0882638892022 0 24.5913940636831 35.6406436968371 19.2842350982821 down 1.0 3.0 0.0 26.0 43.0 18.0

APOL3 -1.13000940225384 7.49291270202032e-07 3.3938486944445e-06 56.4627712766481 45.2945370416322 52.0227120021165 129.577730258638 111.895044164488 94.2784827027125 down 53.0 44.0 49.0 137.0 135.0 88.0

IL7R 1.33696898958542 7.51374375058101e-07 3.40237420836253e-06 83.0961539543123 68.9712268588491 107.230488004363 34.9954453983182 38.1272002338257 28.9263526474231 up 78.0 67.0 101.0 37.0 46.0 27.0

PURG 1.47646596678986 7.8929394577243e-07 3.5664549056775e-06 66.0507890406073 59.7064351912425 104.045424004233 26.4830397608894 28.1809740858712 27.8550062530741 up 62.0 58.0 98.0 28.0 34.0 26.0

CPAMD8 -1.81945163064173 8.09850251619589e-07 3.6515473149949e-06 11.7186883781723 19.5590046316139 12.7402560005183 61.4784851592076 48.0734263817802 46.0678949570072 down 11.0 19.0 12.0 65.0 58.0 43.0

SLC22A3 1.02132444124449 8.29134182653195e-07 3.73551411014498e-06 118.252219088829 119.412870382485 143.327880005831 58.6410166133981 58.0196525297347 71.7802084213833 up 111.0 116.0 135.0 62.0 70.0 67.0

MOB3B -3.02394307073859 8.30175410032864e-07 3.73921070086592e-06 2.13067061421314 3.0882638892022 5.30844000021597 26.4830397608894 33.1540871598484 25.7123134643761 down 2.0 3.0 5.0 28.0 40.0 24.0

TRPM2-AS 2.81026039263357 8.41248904899268e-07 3.78506147383628e-06 30.8947239060905 23.6766898172168 31.8506400012958 4.72911424301597 2.48655653698863 5.35673197174503 up 29.0 23.0 30.0 5.0 3.0 5.0

FAM227A -1.10047176490209 8.76764495517408e-07 3.933366142496e-06 54.332100662435 49.4122222272351 67.9480320027644 130.523553107241 137.589461713371 98.5638682801085 down 51.0 48.0 64.0 138.0 166.0 92.0

PCDHB4 -1.32287225380203 8.78501692497799e-07 3.94011614268274e-06 34.0907298274102 37.0591666704264 26.5422000010799 76.6116507368587 90.3448875105869 77.1369403931284 down 32.0 36.0 25.0 81.0 109.0 72.0

ELMOD1 2.88169978503126 9.19128026770593e-07 4.1103560648083e-06 39.417406362943 25.7355324100183 23.3571360009503 2.83746854580958 6.63081743196968 2.14269278869801 up 37.0 25.0 22.0 3.0 8.0 2.0

LTF -6.42533484929746 1.06047676733866e-06 4.72004110318361e-06 0 0 0 17.9706341234607 10.7750783269507 21.4269278869801 down 0.0 0.0 0.0 19.0 13.0 20.0

SSTR1 -6.14075782302776 1.06899336926261e-06 4.7517049790891e-06 0 0 1.06168800004319 26.4830397608894 26.5232697278787 26.7836598587251 down 0.0 0.0 1.0 28.0 32.0 25.0

CASC19 -6.40265610006144 1.08162851395568e-06 4.80408679245131e-06 0 0 0 15.1331655776511 19.8924522959091 13.9275031265371 down 0.0 0.0 0.0 16.0 24.0 13.0

ZNF479 2.45353779486707 1.08650936223448e-06 4.82323600530716e-06 24.5027120634511 41.1768518560293 46.7142720019006 1.89164569720639 9.94622614795453 8.57077115479204 up 23.0 40.0 44.0 2.0 12.0 8.0

RGS7 2.41171420498288 1.09707850601327e-06 4.86505494551434e-06 28.7640532918774 38.0885879668271 38.220768001555 11.3498741832383 4.14426089498105 4.28538557739602 up 27.0 37.0 36.0 12.0 5.0 4.0

C7orf31 1.36053708563257 1.10183208419493e-06 4.88357810210938e-06 63.9201184263941 73.088912044452 89.1817920036283 30.2663311553022 29.8386784438636 27.8550062530741 up 60.0 71.0 84.0 32.0 36.0 26.0

PSG6 3.39304735033615 1.11361537104993e-06 4.93193317270217e-06 21.3067061421314 19.5590046316139 38.220768001555 0 3.31540871598484 4.28538557739602 up 20.0 19.0 36.0 0.0 4.0 4.0

LAD1 2.00371342339122 1.11850034452826e-06 4.95227282705405e-06 33.0253945203036 60.7358564876432 46.7142720019006 7.56658278882556 10.7750783269507 17.1415423095841 up 31.0 59.0 44.0 8.0 13.0 16.0

LOC100130992 1.67214015539436 1.12121967752632e-06 4.96301576621267e-06 49.0054241269022 66.9123842660476 53.0844000021597 20.8081026692703 12.4327826849432 20.3555814926311 up 46.0 65.0 50.0 22.0 15.0 19.0

NAAA -1.70706553722479 1.12709358711846e-06 4.98641038542351e-06 20.2413708350248 13.3824768532095 15.9253200006479 50.1286109759693 49.7311307397726 62.1380908722423 down 19.0 13.0 15.0 53.0 60.0 58.0

ATP8A2 1.48278685796383 1.16290891434385e-06 5.13815258450309e-06 71.3774655761401 59.7064351912425 61.5779040025053 20.8081026692703 20.7213044749053 27.8550062530741 up 67.0 58.0 58.0 22.0 25.0 26.0

DLX4 1.35420482797036 1.18577867288283e-06 5.23646786200083e-06 66.0507890406073 116.324606493283 83.8733520034124 35.9412682469214 39.7849045918181 27.8550062530741 up 62.0 113.0 79.0 38.0 48.0 26.0

FRMD3 -1.88313034513927 1.20218471993911e-06 5.30338780098138e-06 10.6533530710657 18.5295833352132 15.9253200006479 39.7245596413342 46.4157220237878 81.4223259705244 down 10.0 18.0 15.0 42.0 56.0 76.0

IL16 2.34610859928935 1.22277692179571e-06 5.38861629948475e-06 36.2214004416233 28.8237962992205 47.7759600019437 10.4040513346351 9.11737396895832 2.14269278869801 up 34.0 28.0 45.0 11.0 11.0 2.0

LINC01559 -3.15384202377 1.25692845852709e-06 5.5319220803972e-06 5.32667653553284 0 4.24675200017278 22.6997483664767 34.8117915178408 26.7836598587251 down 5.0 0.0 4.0 24.0 42.0 25.0

NUDT10 2.09110070373619 1.27672298484674e-06 5.61320720563296e-06 60.7241125050744 27.7943750028198 43.529208001771 13.2415198804447 9.11737396895832 8.57077115479204 up 57.0 27.0 41.0 14.0 11.0 8.0

OLFM2 -1.43107244727716 1.33595647512584e-06 5.8675402355799e-06 30.8947239060905 18.5295833352132 38.220768001555 81.3407649798747 89.5160353315907 64.2807836609403 down 29.0 18.0 36.0 86.0 108.0 60.0

ZNF826P 3.86134095157076 1.33890513068483e-06 5.87896654064309e-06 24.5027120634511 23.6766898172168 20.1720720008207 2.83746854580958 0.82885217899621 1.07134639434901 up 23.0 23.0 19.0 3.0 1.0 1.0

LOC101929124 2.88505210402688 1.38309164281339e-06 6.05884986533715e-06 33.0253945203036 22.6472685208161 27.6038880011231 6.62075994022236 2.48655653698863 2.14269278869801 up 31.0 22.0 26.0 7.0 3.0 2.0

KCNMB1 2.76771750805558 1.38434477874604e-06 6.06277160081641e-06 24.5027120634511 51.4710648200366 20.1720720008207 6.62075994022236 3.31540871598484 4.28538557739602 up 23.0 50.0 19.0 7.0 4.0 4.0

HOXA11-AS 2.26760615185356 1.43117635482064e-06 6.25816402959774e-06 31.9600592131971 36.0297453740256 35.0357040014254 6.62075994022236 9.11737396895832 5.35673197174503 up 30.0 35.0 33.0 7.0 11.0 5.0

CATSPER2 1.20070855260456 1.46087889551267e-06 6.38310252514435e-06 83.0961539543123 95.7361805652681 90.2434800036715 46.3453195815565 37.2983480548295 33.2117382248192 up 78.0 93.0 85.0 49.0 45.0 31.0

MICB -1.48162037207515 1.46298276474191e-06 6.39064673407114e-06 21.3067061421314 20.5884259280146 26.5422000010799 68.09924509943 63.8216177827082 58.9240516891953 down 20.0 20.0 25.0 72.0 77.0 55.0

CDX2 6.16111974527412 1.49115144730795e-06 6.5053065973208e-06 14.914694299492 23.6766898172168 33.9740160013822 0.945822848603194 0 0 up 14.0 23.0 32.0 1.0 0.0 0.0

PRDM13 3.5103392050623 1.51298322225681e-06 6.58867243831115e-06 36.2214004416233 25.7355324100183 13.8019440005615 0.945822848603194 1.65770435799242 4.28538557739602 up 34.0 25.0 13.0 1.0 2.0 4.0

METTL7B 1.03832717505952 1.52749827505156e-06 6.64675591558527e-06 118.252219088829 130.736504642893 106.168800004319 66.2075994022236 58.0196525297347 48.2105877457052 up 111.0 127.0 100.0 70.0 70.0 45.0

HMX3 4.60137986275085 1.55553836614843e-06 6.76008720434951e-06 22.3720414492379 20.5884259280146 25.4805120010367 0.945822848603194 0.82885217899621 1.07134639434901 up 21.0 20.0 24.0 1.0 1.0 1.0

HAVCR2 3.40873938105541 1.63994918808161e-06 7.11779124932167e-06 24.5027120634511 20.5884259280146 25.4805120010367 2.83746854580958 0.82885217899621 3.21403918304702 up 23.0 20.0 24.0 3.0 1.0 3.0

ASMTL-AS1 -1.2348987829533 1.80750781622186e-06 7.78915858733105e-06 41.5480769771562 44.2651157452315 27.6038880011231 92.690639163113 93.6602962265718 80.3509795761754 down 39.0 43.0 26.0 98.0 113.0 75.0

SLC27A3 1.50323151508572 1.87924938460755e-06 8.081869656068e-06 82.0308186472058 56.6181713020403 74.3181600030236 32.1579768525086 28.1809740858712 13.9275031265371 up 77.0 55.0 70.0 34.0 34.0 13.0

SAMD14 1.23028596316376 1.90070748939966e-06 8.1617198657618e-06 102.272189482231 80.2948611192571 85.9967280034987 45.3994967329533 29.0098262648674 40.7111629852622 up 96.0 78.0 81.0 48.0 35.0 38.0

CARD11 6.07559998137085 1.91959004207627e-06 8.24071345990167e-06 19.1760355279182 21.6178472244154 27.6038880011231 0 0.82885217899621 0 up 18.0 21.0 26.0 0.0 1.0 0.0

DLGAP1-AS1 -1.02155867541213 1.97644571722042e-06 8.47405478352667e-06 68.1814596548204 59.7064351912425 61.5779040025053 155.114947170924 107.750783269507 122.133488955787 down 64.0 58.0 58.0 164.0 130.0 114.0

DAPK2 -2.82973111813976 2.01237613348133e-06 8.62156087536007e-06 3.19600592131971 3.0882638892022 6.37012800025917 23.6455712150799 43.1003133078029 22.4982742813291 down 3.0 3.0 6.0 25.0 52.0 21.0

KYNU -3.56587098336572 2.07791873792206e-06 8.88887460111102e-06 4.26134122842627 2.05884259280146 0 23.6455712150799 31.496382801856 19.2842350982821 down 4.0 2.0 0.0 25.0 38.0 18.0

CTSS -1.49786881201388 2.08444110998116e-06 8.91227471052016e-06 31.9600592131971 23.6766898172168 14.8636320006047 65.2617765536204 61.3350612457196 72.8515548157324 down 30.0 23.0 14.0 69.0 74.0 68.0

CDKL1 -1.24120476920951 2.12036622565151e-06 9.05216831213121e-06 30.8947239060905 38.0885879668271 36.0973920014686 72.828359342446 87.8583309735983 87.8504043366184 down 29.0 37.0 34.0 77.0 106.0 82.0

NKAIN1 -1.77572154882953 2.19328306705117e-06 9.34227185211136e-06 20.2413708350248 15.441319446011 8.49350400034555 45.3994967329533 56.3619481717423 49.2819341400542 down 19.0 15.0 8.0 48.0 68.0 46.0

PLK5 1.55355853685801 2.20475596329746e-06 9.38642021067077e-06 62.8547831192875 99.853865750871 52.0227120021165 27.4288626094926 29.0098262648674 16.0701959152351 up 59.0 97.0 49.0 29.0 35.0 15.0

FGF22 -3.05722905108492 2.24792686863407e-06 9.55820310106956e-06 1.06533530710657 4.11768518560293 4.24675200017278 27.4288626094926 21.5501566539015 29.9976990417721 down 1.0 4.0 4.0 29.0 26.0 28.0

ATP8A1 -1.00507792385483 2.25073837049699e-06 9.56295660803086e-06 90.5535011040583 68.9712268588491 52.0227120021165 156.060770019527 142.562574787348 125.347528138834 down 85.0 67.0 49.0 165.0 172.0 117.0

KLHL35 1.00555196367925 2.27964355192312e-06 9.67848665904204e-06 129.970907467001 141.0307176069 144.389568005874 89.8531706173035 51.3888350977651 66.4234764496383 up 122.0 137.0 136.0 95.0 62.0 62.0

PRELP 2.80200318137336 2.35944784478652e-06 9.99660220955358e-06 39.417406362943 25.7355324100183 19.1103840007775 2.83746854580958 5.80196525297347 3.21403918304702 up 37.0 25.0 18.0 3.0 7.0 3.0

RASGEF1A 1.25904416360659 2.3598826585599e-06 9.99660220955358e-06 75.6388068045664 90.5890740832644 78.5649120031964 35.9412682469214 26.5232697278787 40.7111629852622 up 71.0 88.0 74.0 38.0 32.0 38.0

HPCAL4 6.34159635498969 2.3968279606921e-06 1.01328339541114e-05 15.9800296065985 8.23537037120586 18.0486960007343 0 0 0 up 15.0 8.0 17.0 0.0 0.0 0.0

ZC3H12B -1.438040776386 2.46364806982476e-06 1.03945699384387e-05 23.4373767563445 43.2356944488308 19.1103840007775 68.09924509943 89.5160353315907 74.9942476044304 down 22.0 42.0 18.0 72.0 108.0 70.0

ZBTB49 -1.10063382583368 2.47970239767253e-06 1.04570969919275e-05 46.874753512689 57.647592598441 41.4058320016846 98.3655762547322 117.697009417462 96.4211754914105 down 44.0 56.0 39.0 104.0 142.0 90.0

NPR3 -2.75916947872654 2.49627157103052e-06 1.05164984862614e-05 27.6987179847708 5.14710648200366 12.7402560005183 114.444564680987 107.750783269507 85.7077115479204 down 26.0 5.0 12.0 121.0 130.0 80.0

SLC34A2 -4.49896170122772 2.50990517648191e-06 1.05660521097424e-05 1.06533530710657 1.02942129640073 1.06168800004319 19.8622798206671 20.7213044749053 31.0690454361212 down 1.0 1.0 1.0 21.0 25.0 29.0

TBX20 6.34291109117295 2.67400690987747e-06 1.12122963003278e-05 20.2413708350248 7.20594907480513 14.8636320006047 0 0 0 up 19.0 7.0 14.0 0.0 0.0 0.0

OSR2 -1.32601871834207 2.68635499002497e-06 1.12584991417672e-05 24.5027120634511 31.9120601884227 35.0357040014254 72.828359342446 66.3081743196968 91.0644435196654 down 23.0 31.0 33.0 77.0 80.0 85.0

TMEM154 1.04374231951014 2.70841701866997e-06 1.13425424712656e-05 134.232248695428 103.971550936474 141.204504005745 45.3994967329533 67.9658786776893 70.7088620270343 up 126.0 101.0 133.0 48.0 82.0 66.0

LOC100996634 3.38459288751294 2.80128732550234e-06 1.1699656630673e-05 26.6333826776642 17.5001620388124 24.4188240009935 3.78329139441278 1.65770435799242 1.07134639434901 up 25.0 17.0 23.0 4.0 2.0 1.0

ARL10 1.30653800987067 2.85581596708035e-06 1.19068477682356e-05 84.1614892614189 92.6479166760659 100.860360004103 42.5620281871437 48.9022785607764 19.2842350982821 up 79.0 90.0 95.0 45.0 59.0 18.0

CASC9 1.10447517685601 2.88709486188231e-06 1.20254209393377e-05 107.598866017763 96.7656018616688 125.279184005097 39.7245596413342 48.0734263817802 66.4234764496383 up 101.0 94.0 118.0 42.0 58.0 62.0

FAM179A -2.82093036138675 2.95888437927114e-06 1.23093078057105e-05 5.32667653553284 4.11768518560293 3.18506400012958 27.4288626094926 43.9291654867992 17.1415423095841 down 5.0 4.0 3.0 29.0 53.0 16.0

GRIK2 -3.08228279499708 2.97903921557298e-06 1.23870702778121e-05 2.13067061421314 2.05884259280146 5.30844000021597 17.9706341234607 37.2983480548295 24.6409670700271 down 2.0 2.0 5.0 19.0 45.0 23.0

NPR2 -1.55705225714449 3.00272765197436e-06 1.24825046440357e-05 25.5680473705576 16.4707407424117 15.9253200006479 56.7493709161917 58.8485047087309 54.6386661117993 down 24.0 16.0 15.0 60.0 71.0 51.0

GOLGA2P9 6.26434582979137 3.04532191999537e-06 1.26533611294387e-05 13.8493589923854 11.3236342604081 14.8636320006047 0 0 0 up 13.0 11.0 14.0 0.0 0.0 0.0

ZMAT1 1.89033587003601 3.07564994298228e-06 1.27762408126826e-05 42.6134122842627 38.0885879668271 46.7142720019006 14.1873427290479 13.2616348639394 6.42807836609403 up 40.0 37.0 44.0 15.0 16.0 6.0

P2RX5 6.4496986488691 3.10189000687645e-06 1.28758420990179e-05 11.7186883781723 4.11768518560293 29.7272640012094 0 0 0 up 11.0 4.0 28.0 0.0 0.0 0.0

DNER -1.54571535305994 3.10236472102496e-06 1.28758420990179e-05 18.1107002208117 15.441319446011 26.5422000010799 52.9660795217789 57.1908003507385 65.3521300552893 down 17.0 15.0 25.0 56.0 69.0 61.0

IL7 -1.84497876063533 3.11105957706269e-06 1.29043460419789e-05 10.6533530710657 12.3530555568088 13.8019440005615 51.0744338245725 36.4694958758333 44.9965485626582 down 10.0 12.0 13.0 54.0 44.0 42.0

NELL2 3.14747925799944 3.16077819037292e-06 1.30913404755299e-05 39.417406362943 18.5295833352132 23.3571360009503 0 6.63081743196968 2.14269278869801 up 37.0 18.0 22.0 0.0 8.0 2.0

FGF9 5.22941324022157 3.26903880302106e-06 1.35265064296963e-05 23.4373767563445 24.7061111136176 22.2954480009071 0 0.82885217899621 1.07134639434901 up 22.0 24.0 21.0 0.0 1.0 1.0

SAMD10 1.2820638030293 3.27411725618499e-06 1.35442115555003e-05 67.1161243477138 91.6184953796652 104.045424004233 43.5078510357469 25.6944175488825 39.6398165909132 up 63.0 89.0 98.0 46.0 31.0 37.0

SCEL -3.4699812062555 3.33543533709116e-06 1.3787751552373e-05 1.06533530710657 2.05884259280146 3.18506400012958 19.8622798206671 22.3790088328977 27.8550062530741 down 1.0 2.0 3.0 21.0 27.0 26.0

ZNF467 2.51092633325426 3.34528637589106e-06 1.3818373861886e-05 21.3067061421314 40.1474305596286 29.7272640012094 2.83746854580958 5.80196525297347 7.49942476044304 up 20.0 39.0 28.0 3.0 7.0 7.0

ANO2 2.31498613885409 3.50127728858628e-06 1.44240363007421e-05 43.6787475913693 35.0003240776249 26.5422000010799 5.67493709161917 3.31540871598484 12.8561567321881 up 41.0 34.0 25.0 6.0 4.0 12.0

SARM1 1.41914782094696 3.56455258297306e-06 1.46704375013517e-05 59.6587771979678 82.3537037120586 61.5779040025053 17.9706341234607 27.3521219068749 31.0690454361212 up 56.0 80.0 58.0 19.0 33.0 29.0

DEPTOR -1.47446684566911 3.57615348744387e-06 1.47146077428465e-05 24.5027120634511 17.5001620388124 32.912328001339 52.0202566731757 83.7140700786173 71.7802084213833 down 23.0 17.0 31.0 55.0 101.0 67.0

LRRC63 6.26590724475274 3.72336299512552e-06 1.52980279256431e-05 20.2413708350248 10.2942129640073 9.55519200038875 0 0 0 up 19.0 10.0 9.0 0.0 0.0 0.0

NTSR1 -6.17447527159908 3.73051134761138e-06 1.53236814327199e-05 0 0 0 14.1873427290479 10.7750783269507 17.1415423095841 down 0.0 0.0 0.0 15.0 13.0 16.0

SHISA8 -1.36777126394913 3.79459456387517e-06 1.55831350089807e-05 27.6987179847708 38.0885879668271 21.2337600008639 90.7989934659067 64.6504699617044 69.6375156326853 down 26.0 37.0 20.0 96.0 78.0 65.0

IFIT2 -1.17810240621673 4.15394680170863e-06 1.69847595512779e-05 43.6787475913693 33.9709027812242 40.3441440016414 74.7200050396524 88.6871831525945 103.920600251854 down 41.0 33.0 38.0 79.0 107.0 97.0

OAS1 -1.62572942553623 4.16752063235779e-06 1.70320384830256e-05 11.7186883781723 18.5295833352132 20.1720720008207 50.1286109759693 48.0734263817802 57.8527052948463 down 11.0 18.0 19.0 53.0 58.0 54.0

LYPD3 -1.41072503042583 4.20511580617108e-06 1.71773961313089e-05 23.4373767563445 25.7355324100183 22.2954480009071 67.1534222508268 53.8753916347537 69.6375156326853 down 22.0 25.0 21.0 71.0 65.0 65.0

CLCN4 -1.02488713133296 4.71324842683892e-06 1.91606499521601e-05 72.4428008832467 52.5004861164373 55.2077760022461 148.494187230702 108.579635448504 109.277332223599 down 68.0 51.0 52.0 157.0 131.0 102.0

OTUD7A 1.13441400736721 4.81456218650137e-06 1.95350139974451e-05 76.7041421116729 140.0012963105 133.772688005442 59.5868394620012 54.7042438137499 44.9965485626582 up 72.0 136.0 126.0 63.0 66.0 42.0

WT1 1.33716972988586 5.03391003395633e-06 2.03762093130753e-05 60.7241125050744 76.1771759336542 65.8246560026781 30.2663311553022 21.5501566539015 28.9263526474231 up 57.0 74.0 62.0 32.0 26.0 27.0

GIPR 2.05182411089211 5.04416730326686e-06 2.04079756669072e-05 46.874753512689 56.6181713020403 22.2954480009071 10.4040513346351 7.45966961096589 12.8561567321881 up 44.0 55.0 21.0 11.0 9.0 12.0

SH3TC2 -1.12170435860142 5.32616833497992e-06 2.14719875284531e-05 31.9600592131971 52.5004861164373 48.8376480019869 96.4739305575258 104.435374553523 88.9217507309674 down 30.0 51.0 46.0 102.0 126.0 83.0

ADPRH 2.53248806020763 5.40175021799181e-06 2.17508078661234e-05 23.4373767563445 25.7355324100183 31.8506400012958 3.78329139441278 5.80196525297347 4.28538557739602 up 22.0 25.0 30.0 4.0 7.0 4.0

RPS6KA6 1.17133891287586 5.53758883603911e-06 2.22639418134925e-05 83.0961539543123 83.3831250084593 75.3798480030668 36.8870910955246 38.9560524128219 31.0690454361212 up 78.0 81.0 71.0 39.0 47.0 29.0

SYT14 -3.27375485467642 5.53838552550516e-06 2.22639418134925e-05 5.32667653553284 2.05884259280146 0 19.8622798206671 25.6944175488825 25.7123134643761 down 5.0 2.0 0.0 21.0 31.0 24.0

LOC101927066 -6.10785050289512 5.5555963869139e-06 2.23278298444416e-05 0 0 0 16.0789884262543 9.11737396895832 14.9988495208861 down 0.0 0.0 0.0 17.0 11.0 14.0

MAP1LC3A -1.54892996276099 5.57772166265096e-06 2.2402043223824e-05 9.58801776395912 23.6766898172168 30.7889520012526 60.5326623106044 63.8216177827082 63.2094372665913 down 9.0 23.0 29.0 64.0 77.0 59.0

FAM86JP 1.2858908770431 5.57802941665227e-06 2.2402043223824e-05 85.2268245685255 59.7064351912425 83.8733520034124 30.2663311553022 25.6944175488825 38.5684701965642 up 80.0 58.0 79.0 32.0 31.0 36.0

PPP1R3G 1.35441155263296 5.60158456162164e-06 2.24913113235057e-05 62.8547831192875 75.1477546372535 75.3798480030668 35.9412682469214 18.2347479379166 29.9976990417721 up 59.0 73.0 71.0 38.0 22.0 28.0

LOC101928370 1.69744953673968 5.7910045654196e-06 2.32023692852904e-05 56.4627712766481 42.20627315243 69.0097200028076 9.45822848603194 26.5232697278787 14.9988495208861 up 53.0 41.0 65.0 10.0 32.0 14.0

EVPL -3.46153952902948 5.94811914038371e-06 2.37812457489025e-05 2.13067061421314 4.11768518560293 0 31.2121540039054 19.8924522959091 18.2128887039331 down 2.0 4.0 0.0 33.0 24.0 17.0

LOC101927045 -2.15664785564259 6.08701913380017e-06 2.43078981910832e-05 8.52268245685255 5.14710648200366 10.6168800004319 31.2121540039054 28.1809740858712 49.2819341400542 down 8.0 5.0 10.0 33.0 34.0 46.0

STYK1 2.40662975494924 6.09175125272723e-06 2.43210620365777e-05 26.6333826776642 29.8532175956212 28.6655760011662 7.56658278882556 3.31540871598484 5.35673197174503 up 25.0 29.0 27.0 8.0 4.0 5.0

DOK1 1.05149417071635 6.22076808469458e-06 2.48010852800015e-05 104.402860096444 124.559976864489 91.3051680037147 55.8035480675885 57.1908003507385 40.7111629852622 up 98.0 121.0 86.0 59.0 69.0 38.0

ZNF337-AS1 1.8344719732334 6.49683986954266e-06 2.58166707459659e-05 43.6787475913693 29.8532175956212 61.5779040025053 13.2415198804447 9.94622614795453 14.9988495208861 up 41.0 29.0 58.0 14.0 12.0 14.0

C8orf34 -3.31989594767991 6.64303446171867e-06 2.63481629083386e-05 3.19600592131971 1.02942129640073 3.18506400012958 11.3498741832383 28.1809740858712 34.2830846191682 down 3.0 1.0 3.0 12.0 34.0 32.0

C10orf11 2.77231358335248 6.9483552404053e-06 2.75140574503193e-05 23.4373767563445 35.0003240776249 20.1720720008207 4.72911424301597 0.82885217899621 6.42807836609403 up 22.0 34.0 19.0 5.0 1.0 6.0

CBLN1 1.62376428069486 7.11636152097041e-06 2.81530042422323e-05 36.2214004416233 75.1477546372535 55.2077760022461 20.8081026692703 19.0636001169128 13.9275031265371 up 34.0 73.0 52.0 22.0 23.0 13.0

SLC9A7P1 2.36537100824606 7.35629458660646e-06 2.90594250144604e-05 23.4373767563445 33.9709027812242 35.0357040014254 1.89164569720639 6.63081743196968 9.64211754914105 up 22.0 33.0 33.0 2.0 8.0 9.0

FOXN3-AS1 1.21704744043255 7.35748960372023e-06 2.90594250144604e-05 63.9201184263941 86.4713888976615 81.749976003326 35.9412682469214 35.6406436968371 27.8550062530741 up 60.0 84.0 77.0 38.0 43.0 26.0

WDFY4 -1.75042739561828 7.40327458416988e-06 2.92266304954178e-05 13.8493589923854 12.3530555568088 12.7402560005183 45.3994967329533 49.7311307397726 35.3544310135172 down 13.0 12.0 12.0 48.0 60.0 33.0

LINC00467 1.0251099779558 7.44083561712938e-06 2.93680697470111e-05 106.533530710657 95.7361805652681 105.107112004276 60.5326623106044 46.4157220237878 43.9252021683092 up 100.0 93.0 99.0 64.0 56.0 41.0

HS1BP3-IT1 1.82428515525977 7.64300747769251e-06 3.01239056938369e-05 31.9600592131971 47.3533796344337 44.5908960018142 14.1873427290479 8.28852178996211 12.8561567321881 up 30.0 46.0 42.0 15.0 10.0 12.0

PDE4B -3.61336648469379 7.9146807728799e-06 3.11584225639288e-05 2.13067061421314 3.0882638892022 0 18.9164569720639 24.8655653698863 20.3555814926311 down 2.0 3.0 0.0 20.0 30.0 19.0

LINC00534 -6.01611447304031 8.18094109338387e-06 3.21525168326086e-05 0 0 0 14.1873427290479 11.6039305059469 11.7848103378391 down 0.0 0.0 0.0 15.0 14.0 11.0

LINC00898 6.15093434825945 8.49893744456403e-06 3.33422881683452e-05 12.7840236852788 5.14710648200366 19.1103840007775 0 0 0 up 12.0 5.0 18.0 0.0 0.0 0.0

GCSAM 1.58858520060626 8.6289707847919e-06 3.38158514052049e-05 46.874753512689 52.5004861164373 44.5908960018142 19.8622798206671 14.0904870429356 13.9275031265371 up 44.0 51.0 42.0 21.0 17.0 13.0

STC2 1.48316370091531 8.765989877688e-06 3.43105056672908e-05 68.1814596548204 41.1768518560293 63.7012800025917 23.6455712150799 19.8924522959091 18.2128887039331 up 64.0 40.0 60.0 25.0 24.0 17.0

LOC728739 6.0595202314733 9.24246557422814e-06 3.60505104368926e-05 13.8493589923854 11.3236342604081 9.55519200038875 0 0 0 up 13.0 11.0 9.0 0.0 0.0 0.0

KCTD19 2.06042590883363 9.2458584818225e-06 3.60554426063704e-05 43.6787475913693 24.7061111136176 46.7142720019006 4.72911424301597 8.28852178996211 14.9988495208861 up 41.0 24.0 44.0 5.0 10.0 14.0

FIRRE -5.9905232093676 9.32411532625546e-06 3.63339854217997e-05 0 0 0 12.2956970318415 10.7750783269507 13.9275031265371 down 0.0 0.0 0.0 13.0 13.0 13.0

HIST1H3H -1.6788952591848 9.47878926785333e-06 3.68958387402195e-05 12.7840236852788 20.5884259280146 11.6785680004751 37.8329139441278 57.1908003507385 49.2819341400542 down 12.0 20.0 11.0 40.0 69.0 46.0

MMP19 1.42226101660526 9.51442682614616e-06 3.70260488019563e-05 67.1161243477138 56.6181713020403 78.5649120031964 25.5372169122862 34.8117915178408 13.9275031265371 up 63.0 55.0 74.0 27.0 42.0 13.0

TTC39A 1.34349063191926 9.68306219110249e-06 3.76477102403663e-05 69.246794961927 66.9123842660476 65.8246560026781 17.9706341234607 24.8655653698863 37.4971238022152 up 65.0 65.0 62.0 19.0 30.0 35.0

SPANXN3 5.05635251186341 1.00623167624357e-05 3.90237284697025e-05 17.0453649137051 17.5001620388124 27.6038880011231 0.945822848603194 0.82885217899621 0 up 16.0 17.0 26.0 1.0 1.0 0.0

HBQ1 -3.04690953679417 1.01044452118303e-05 3.91602154857938e-05 4.26134122842627 4.11768518560293 0 22.6997483664767 23.2078610118939 23.5696206756781 down 4.0 4.0 0.0 24.0 28.0 22.0

ALDH1A2 2.06744809583895 1.01445522272298e-05 3.9297670873376e-05 44.7440828984759 23.6766898172168 33.9740160013822 7.56658278882556 8.28852178996211 8.57077115479204 up 42.0 23.0 32.0 8.0 10.0 8.0

HRASLS2 -5.70174057498871 1.05692326325026e-05 4.08493727571512e-05 1.06533530710657 0 0 20.8081026692703 24.8655653698863 12.8561567321881 down 1.0 0.0 0.0 22.0 30.0 12.0

ST3GAL1 -1.07323963887209 1.0797540816696e-05 4.16652258393689e-05 57.5281065837547 50.4416435236359 36.0973920014686 103.094690497748 85.3717744366097 115.705410589693 down 54.0 49.0 34.0 109.0 103.0 108.0

OASL -5.68363088215019 1.08652728594973e-05 4.19081165728778e-05 0 0 1.06168800004319 16.0789884262543 16.5770435799242 25.7123134643761 down 0.0 0.0 1.0 17.0 20.0 24.0

ALOX12P2 1.28182294347541 1.15620221252368e-05 4.44430802817135e-05 57.5281065837547 81.3242824156578 65.8246560026781 23.6455712150799 31.496382801856 28.9263526474231 up 54.0 79.0 62.0 25.0 38.0 27.0

LOC102724719 5.72993941955224 1.15784849545037e-05 4.44861726308217e-05 23.4373767563445 11.3236342604081 19.1103840007775 0.945822848603194 0 0 up 22.0 11.0 18.0 1.0 0.0 0.0

LOC344887 -5.64531826509039 1.20320480057289e-05 4.61450969474865e-05 0 0 1.06168800004319 18.9164569720639 17.4058957589204 20.3555814926311 down 0.0 0.0 1.0 20.0 21.0 19.0

TSPAN7 2.26676246497064 1.23889062686413e-05 4.73849790451079e-05 30.8947239060905 29.8532175956212 31.8506400012958 3.78329139441278 11.6039305059469 3.21403918304702 up 29.0 29.0 30.0 4.0 14.0 3.0

MCEE 1.06410753172637 1.243577902064e-05 4.75535206793772e-05 138.493589923854 116.324606493283 125.279184005097 83.2324106770811 36.4694958758333 63.2094372665913 up 130.0 113.0 118.0 88.0 44.0 59.0

GSG1L -2.39986484664769 1.24901197456815e-05 4.77397627463549e-05 4.26134122842627 5.14710648200366 7.43181600030236 37.8329139441278 18.2347479379166 33.2117382248192 down 4.0 5.0 7.0 40.0 22.0 31.0

SYNPO2 -2.54183675600543 1.25716659706007e-05 4.80189451052931e-05 4.26134122842627 3.0882638892022 6.37012800025917 30.2663311553022 29.8386784438636 19.2842350982821 down 4.0 3.0 6.0 32.0 36.0 18.0

PDE4A 1.1743142052036 1.26987109789574e-05 4.84823448238763e-05 73.5081361903532 76.1771759336542 87.0584160035419 34.049622549715 43.1003133078029 26.7836598587251 up 69.0 74.0 82.0 36.0 52.0 25.0

GRIN2B -1.47368635363295 1.2884312954723e-05 4.91687905954061e-05 22.3720414492379 38.0885879668271 18.0486960007343 104.040513346351 59.6773568877272 54.6386661117993 down 21.0 37.0 17.0 110.0 72.0 51.0

DCN -5.96767099887291 1.30038317042867e-05 4.96025465144373e-05 0 0 0 17.9706341234607 10.7750783269507 7.49942476044304 down 0.0 0.0 0.0 19.0 13.0 7.0

ASIC2 2.14070527267655 1.30727533788146e-05 4.98429984778572e-05 25.5680473705576 43.2356944488308 32.912328001339 3.78329139441278 12.4327826849432 6.42807836609403 up 24.0 42.0 31.0 4.0 15.0 6.0

UGT2B11 -2.44059001411447 1.32549430579534e-05 5.05035392266601e-05 7.45734714974598 2.05884259280146 6.37012800025917 24.5913940636831 37.2983480548295 23.5696206756781 down 7.0 2.0 6.0 26.0 45.0 22.0

CRABP2 1.07178392301369 1.39335469164809e-05 5.28987639545466e-05 85.2268245685255 77.2065972300549 98.7369840040171 41.6162053385406 38.9560524128219 43.9252021683092 up 80.0 75.0 93.0 44.0 47.0 41.0

SCNN1D 1.08993175960487 1.42211117737551e-05 5.39059372225131e-05 80.9654833400992 96.7656018616688 80.6882880032828 47.2911424301597 34.8117915178408 39.6398165909132 up 76.0 94.0 76.0 50.0 42.0 37.0

PDCD4-AS1 1.16932371942435 1.42642088005412e-05 5.40572029264358e-05 71.3774655761401 77.2065972300549 71.133096002894 36.8870910955246 29.8386784438636 31.0690454361212 up 67.0 75.0 67.0 39.0 36.0 29.0

NPTX1 -2.12127650219016 1.44050999739744e-05 5.45667248566918e-05 10.6533530710657 1.02942129640073 14.8636320006047 32.1579768525086 38.9560524128219 43.9252021683092 down 10.0 1.0 14.0 34.0 47.0 41.0

LOC101927229 4.97021209070093 1.44528923015539e-05 5.47232891346273e-05 14.914694299492 20.5884259280146 23.3571360009503 0 0.82885217899621 1.07134639434901 up 14.0 20.0 22.0 0.0 1.0 1.0

C10orf142 5.66492851603207 1.45483845419551e-05 5.50479415101004e-05 12.7840236852788 20.5884259280146 18.0486960007343 0 0.82885217899621 0 up 12.0 20.0 17.0 0.0 1.0 0.0

TSTD1 -3.06124797974259 1.45626235760774e-05 5.50895139300472e-05 1.06533530710657 3.0882638892022 4.24675200017278 32.1579768525086 24.8655653698863 12.8561567321881 down 1.0 3.0 4.0 34.0 30.0 12.0

COPZ2 -3.55541146502275 1.47282428228555e-05 5.56787398837697e-05 3.19600592131971 0 2.12337600008639 24.5913940636831 14.0904870429356 23.5696206756781 down 3.0 0.0 2.0 26.0 17.0 22.0

IKZF2 -1.29246676093849 1.47861215060051e-05 5.5885073251166e-05 34.0907298274102 24.7061111136176 21.2337600008639 63.370130856414 67.137026498693 65.3521300552893 down 32.0 24.0 20.0 67.0 81.0 61.0

LOC101929122 -2.86630255141452 1.49938474295523e-05 5.6619655696972e-05 2.13067061421314 2.05884259280146 5.30844000021597 26.4830397608894 23.2078610118939 19.2842350982821 down 2.0 2.0 5.0 28.0 28.0 18.0

CD6 4.26839392978897 1.51722685422274e-05 5.72551189809159e-05 15.9800296065985 19.5590046316139 19.1103840007775 0 0.82885217899621 2.14269278869801 up 15.0 19.0 18.0 0.0 1.0 2.0

HYDIN2 -3.84410087277274 1.61414470382906e-05 6.06961404725067e-05 4.26134122842627 0 0 22.6997483664767 16.5770435799242 21.4269278869801 down 4.0 0.0 0.0 24.0 20.0 20.0

KIF5C 1.34523406666131 1.74598972779656e-05 6.54078609025659e-05 51.1360947411153 70.0006481552498 55.2077760022461 20.8081026692703 24.0367131908901 24.6409670700271 up 48.0 68.0 52.0 22.0 29.0 23.0

PTPRN2 4.96364476072361 1.85516359965307e-05 6.92670737891182e-05 13.8493589923854 20.5884259280146 23.3571360009503 0 1.65770435799242 0 up 13.0 20.0 22.0 0.0 2.0 0.0

SNHG4 1.0542859323068 1.96193522021925e-05 7.30283072522833e-05 103.337524789337 91.6184953796652 82.8116640033692 43.5078510357469 35.6406436968371 55.7100125061483 up 97.0 89.0 78.0 46.0 43.0 52.0

KCNH5 5.9767475996433 2.06849306725826e-05 7.6792181808799e-05 14.914694299492 4.11768518560293 13.8019440005615 0 0 0 up 14.0 4.0 13.0 0.0 0.0 0.0

AREG -5.54824931820278 2.07069163988813e-05 7.68569596400197e-05 1.06533530710657 0 0 12.2956970318415 18.2347479379166 22.4982742813291 down 1.0 0.0 0.0 13.0 22.0 21.0

ADCY2 3.89982844709435 2.07124900670788e-05 7.68608065139793e-05 15.9800296065985 18.5295833352132 20.1720720008207 0 3.31540871598484 0 up 15.0 18.0 19.0 0.0 4.0 0.0

MALRD1 5.5777710718003 2.07591230720017e-05 7.6983262880847e-05 18.1107002208117 14.4118981496103 15.9253200006479 0 0.82885217899621 0 up 17.0 14.0 15.0 0.0 1.0 0.0

F12 1.06392348660777 2.10002668162656e-05 7.78264099469566e-05 77.7694774187795 84.41254630486 85.9967280034987 36.8870910955246 37.2983480548295 44.9965485626582 up 73.0 82.0 81.0 39.0 45.0 42.0

C14orf105 -1.0563029646057 2.11256825586028e-05 7.82398475169943e-05 44.7440828984759 35.0003240776249 47.7759600019437 87.0157020714939 96.9757049425566 80.3509795761754 down 42.0 34.0 45.0 92.0 117.0 75.0

PTPN5 -5.88944437062082 2.1491225492144e-05 7.95414812840112e-05 0 0 0 9.45822848603194 18.2347479379166 6.42807836609403 down 0.0 0.0 0.0 10.0 22.0 6.0

LIN7A 2.84495666543352 2.15754315910993e-05 7.98356948783797e-05 33.0253945203036 15.441319446011 20.1720720008207 4.72911424301597 0.82885217899621 4.28538557739602 up 31.0 15.0 19.0 5.0 1.0 4.0

LOC440461 -1.27852142301613 2.16094392321423e-05 7.98953316806078e-05 31.9600592131971 41.1768518560293 18.0486960007343 85.1240563742875 72.9389917516665 63.2094372665913 down 30.0 40.0 17.0 90.0 88.0 59.0

ATP2B2 -3.75823423471116 2.19177210786284e-05 8.09431425925364e-05 1.06533530710657 1.02942129640073 2.12337600008639 22.6997483664767 14.0904870429356 20.3555814926311 down 1.0 1.0 2.0 24.0 17.0 19.0

PDE6A -2.01596417799585 2.19360594638378e-05 8.09932099645624e-05 7.45734714974598 9.26479166760659 9.55519200038875 27.4288626094926 51.3888350977651 26.7836598587251 down 7.0 9.0 9.0 29.0 62.0 25.0

PCDH19 -1.37798689671482 2.38643434365459e-05 8.76353734695617e-05 15.9800296065985 27.7943750028198 23.3571360009503 66.2075994022236 48.9022785607764 59.9953980835443 down 15.0 27.0 22.0 70.0 59.0 56.0

CCDC158 3.66528149860263 2.40968577745234e-05 8.84508712243613e-05 28.7640532918774 9.26479166760659 20.1720720008207 0.945822848603194 3.31540871598484 0 up 27.0 9.0 19.0 1.0 4.0 0.0

ARHGDIB 1.54200853590966 2.52335613871566e-05 9.24415721490836e-05 53.2667653553284 47.3533796344337 39.2824560015982 11.3498741832383 16.5770435799242 20.3555814926311 up 50.0 46.0 37.0 12.0 20.0 19.0

GUCA1C 2.92201064624785 2.53680920515665e-05 9.28354891668905e-05 25.5680473705576 11.3236342604081 32.912328001339 0.945822848603194 5.80196525297347 2.14269278869801 up 24.0 11.0 31.0 1.0 7.0 2.0

VSNL1 -5.7968265044914 2.55663923752501e-05 9.35005800597444e-05 0 0 0 12.2956970318415 8.28852178996211 11.7848103378391 down 0.0 0.0 0.0 13.0 10.0 11.0

TOX2 -3.330481318609 2.57103537852655e-05 9.39864896681912e-05 15.9800296065985 15.441319446011 1.06168800004319 131.469375955844 96.9757049425566 98.5638682801085 down 15.0 15.0 1.0 139.0 117.0 92.0

C9orf84 1.79075987537651 2.57303906219635e-05 9.4039442747802e-05 54.332100662435 60.7358564876432 27.6038880011231 5.67493709161917 17.4058957589204 18.2128887039331 up 51.0 59.0 26.0 6.0 21.0 17.0

PTHLH -1.97464417811644 2.60382489117859e-05 9.50825472226024e-05 7.45734714974598 12.3530555568088 7.43181600030236 23.6455712150799 34.8117915178408 49.2819341400542 down 7.0 12.0 7.0 25.0 42.0 46.0

C10orf10 2.54610928904622 2.66919478395445e-05 9.73227715027731e-05 14.914694299492 30.882638892022 32.912328001339 3.78329139441278 1.65770435799242 8.57077115479204 up 14.0 30.0 31.0 4.0 2.0 8.0

FLT1 5.87318295869667 2.77271364094915e-05 0.000100836773460023 6.39201184263941 9.26479166760659 14.8636320006047 0 0 0 up 6.0 9.0 14.0 0.0 0.0 0.0

PRR15 -4.80230109788788 2.80208088076871e-05 0.000101882915046624 1.06533530710657 0 1.06168800004319 25.5372169122862 14.0904870429356 19.2842350982821 down 1.0 0.0 1.0 27.0 17.0 18.0

LINC00494 2.37433168441315 2.93925289914572e-05 0.000106664404694791 22.3720414492379 23.6766898172168 26.5422000010799 3.78329139441278 5.80196525297347 4.28538557739602 up 21.0 23.0 25.0 4.0 7.0 4.0

LINC00106 -1.84465439243815 2.95099139092434e-05 0.000107067453763672 10.6533530710657 7.20594907480513 11.6785680004751 41.6162053385406 33.1540871598484 31.0690454361212 down 10.0 7.0 11.0 44.0 40.0 29.0

CYB5R2 -5.48957780831588 2.98436870062097e-05 0.000108070127807865 0 1.02942129640073 0 22.6997483664767 18.2347479379166 9.64211754914105 down 0.0 1.0 0.0 24.0 22.0 9.0

TNXB -1.57004659967839 3.06057607277865e-05 0.00011066415938713 19.1760355279182 12.3530555568088 14.8636320006047 56.7493709161917 38.1272002338257 42.8538557739602 down 18.0 12.0 14.0 60.0 46.0 40.0

UGT2A3 1.67295601752526 3.12421800174611e-05 0.000112748728056198 45.8094182055824 31.9120601884227 45.6525840018574 8.51240563742875 13.2616348639394 17.1415423095841 up 43.0 31.0 43.0 9.0 16.0 16.0

PAK6 1.93355046210139 3.21086705660788e-05 0.000115678621733172 27.6987179847708 33.9709027812242 33.9740160013822 8.51240563742875 10.7750783269507 5.35673197174503 up 26.0 33.0 32.0 9.0 13.0 5.0

NTN3 2.5740123499155 3.2335780025857e-05 0.000116472063286842 24.5027120634511 24.7061111136176 18.0486960007343 1.89164569720639 4.14426089498105 5.35673197174503 up 23.0 24.0 17.0 2.0 5.0 5.0

CFAP221 1.47323121314936 3.25924300093162e-05 0.000117296741949823 45.8094182055824 73.088912044452 43.529208001771 23.6455712150799 21.5501566539015 12.8561567321881 up 43.0 71.0 41.0 25.0 26.0 12.0

LINC00847 1.06181156773223 3.4094797047505e-05 0.000122495410813306 83.0961539543123 73.088912044452 90.2434800036715 44.4536738843501 32.3252349808522 41.7825093796112 up 78.0 71.0 85.0 47.0 39.0 39.0

CLDN14 -1.53069841151057 3.51757699743135e-05 0.000126004978508114 15.9800296065985 15.441319446011 18.0486960007343 53.9119023703821 33.9829393388446 55.7100125061483 down 15.0 15.0 17.0 57.0 41.0 52.0

LRRN2 1.14290326955888 3.56010140412428e-05 0.000127447417129893 71.3774655761401 66.9123842660476 75.3798480030668 30.2663311553022 38.9560524128219 26.7836598587251 up 67.0 65.0 71.0 32.0 47.0 25.0

OR12D2 4.89260367246474 3.58276753152185e-05 0.000128231738821002 29.8293885989839 11.3236342604081 13.8019440005615 0 1.65770435799242 0 up 28.0 11.0 13.0 0.0 2.0 0.0

PTH2R 1.04006036094211 3.58373359178468e-05 0.000128239220627023 78.8348127258861 81.3242824156578 94.4902320038443 42.5620281871437 47.244574202784 33.2117382248192 up 74.0 79.0 89.0 45.0 57.0 31.0

KCND1 1.17816582727986 3.58622856049107e-05 0.000128301397707959 68.1814596548204 79.2654398228564 57.3311520023325 29.320508306699 27.3521219068749 34.2830846191682 up 64.0 77.0 54.0 31.0 33.0 32.0

PIR -1.72404036091243 3.58942505282072e-05 0.000128361537671064 18.1107002208117 16.4707407424117 4.24675200017278 41.6162053385406 43.9291654867992 42.8538557739602 down 17.0 16.0 4.0 44.0 53.0 40.0

ERBB4 -4.08014956865901 3.67801347022798e-05 0.000131390865005614 0 2.05884259280146 1.06168800004319 18.9164569720639 18.2347479379166 16.0701959152351 down 0.0 2.0 1.0 20.0 22.0 15.0

PSG11 3.09630711368617 3.72183325005271e-05 0.000132732326854511 18.1107002208117 14.4118981496103 24.4188240009935 0.945822848603194 1.65770435799242 4.28538557739602 up 17.0 14.0 23.0 1.0 2.0 4.0

NTRK3 4.17164732453081 3.74463044785952e-05 0.000133461055726363 10.6533530710657 18.5295833352132 21.2337600008639 0 1.65770435799242 1.07134639434901 up 10.0 18.0 20.0 0.0 2.0 1.0

LECT2 2.65874909911269 3.79486220964119e-05 0.000135109217804375 26.6333826776642 24.7061111136176 18.0486960007343 0.945822848603194 7.45966961096589 2.14269278869801 up 25.0 24.0 17.0 1.0 9.0 2.0

SERPINF2 3.76144337071881 3.89201653956098e-05 0.000138422759143739 13.8493589923854 21.6178472244154 14.8636320006047 1.89164569720639 1.65770435799242 0 up 13.0 21.0 14.0 2.0 2.0 0.0

FAM155B -3.03843906614155 3.93586228092304e-05 0.000139894055893488 1.06533530710657 4.11768518560293 2.12337600008639 26.4830397608894 15.748191400928 18.2128887039331 down 1.0 4.0 2.0 28.0 19.0 17.0

ELOVL2-AS1 4.78949942799352 3.95576478998935e-05 0.000140542482261786 22.3720414492379 15.441319446011 13.8019440005615 0.945822848603194 0.82885217899621 0 up 21.0 15.0 13.0 1.0 1.0 0.0

APOE 1.59651074714093 4.02429540609473e-05 0.000142827496709082 42.6134122842627 39.1180092632278 53.0844000021597 20.8081026692703 15.748191400928 7.49942476044304 up 40.0 38.0 50.0 22.0 19.0 7.0

PTPRCAP 1.10537759189939 4.10440465772969e-05 0.000145396517988166 95.8801776395912 84.41254630486 76.44153600311 30.2663311553022 54.7042438137499 33.2117382248192 up 90.0 82.0 72.0 32.0 66.0 31.0

NBPF6 5.76697054719862 4.10842880069204e-05 0.000145508642867914 7.45734714974598 11.3236342604081 9.55519200038875 0 0 0 up 7.0 11.0 9.0 0.0 0.0 0.0

XK 1.3085100406223 4.22698085143163e-05 0.00014945743189992 61.789447812181 47.3533796344337 61.5779040025053 19.8622798206671 28.1809740858712 20.3555814926311 up 58.0 46.0 58.0 21.0 34.0 19.0

RDM1 1.23077255083378 4.37737290711614e-05 0.000154549181005726 64.9854537335007 73.088912044452 66.8863440027212 38.778736792731 18.2347479379166 31.0690454361212 up 61.0 71.0 63.0 41.0 22.0 29.0

GRHL1 1.41743310350433 4.58723852945584e-05 0.000161521140488426 42.6134122842627 94.7067592688674 59.4545280024189 16.0789884262543 24.8655653698863 33.2117382248192 up 40.0 92.0 56.0 17.0 30.0 31.0

CALY 2.40221359294974 4.59722778275443e-05 0.000161771995512796 14.914694299492 35.0003240776249 25.4805120010367 3.78329139441278 3.31540871598484 7.49942476044304 up 14.0 34.0 24.0 4.0 4.0 7.0

ERICH5 3.41744064102916 4.60928693212515e-05 0.000162128988019518 20.2413708350248 14.4118981496103 15.9253200006479 1.89164569720639 0.82885217899621 2.14269278869801 up 19.0 14.0 15.0 2.0 1.0 2.0

ARRDC3-AS1 1.58134275022562 4.71925305891112e-05 0.000165824822273293 33.0253945203036 44.2651157452315 43.529208001771 15.1331655776511 12.4327826849432 12.8561567321881 up 31.0 43.0 41.0 16.0 15.0 12.0

TERT -2.37796620742799 4.74600337538635e-05 0.00016662652265087 2.13067061421314 9.26479166760659 3.18506400012958 23.6455712150799 24.8655653698863 27.8550062530741 down 2.0 9.0 3.0 25.0 30.0 26.0

ADGRE1 1.01370201134548 4.808598477233e-05 0.000168614486036694 80.9654833400992 103.971550936474 80.6882880032828 50.1286109759693 45.5868698447916 35.3544310135172 up 76.0 101.0 76.0 53.0 55.0 33.0

CLDN7 -1.90679794473079 4.90071200249982e-05 0.000171773352622278 9.58801776395912 5.14710648200366 11.6785680004751 40.6703824899374 28.1809740858712 29.9976990417721 down 9.0 5.0 11.0 43.0 34.0 28.0

NOX4 -5.65938229518103 4.91050396579264e-05 0.000172080962309738 0 0 0 8.51240563742875 9.11737396895832 11.7848103378391 down 0.0 0.0 0.0 9.0 11.0 11.0

SDHAP3 1.18365560503506 5.00965840063524e-05 0.000175446792033825 73.5081361903532 55.5887500056395 88.1201040035851 40.6703824899374 29.0098262648674 25.7123134643761 up 69.0 54.0 83.0 43.0 35.0 24.0

TRAF1 1.40230297386758 5.0223821259825e-05 0.000175819700793849 50.0707594340087 68.9712268588491 52.0227120021165 33.1037997011118 16.5770435799242 14.9988495208861 up 47.0 67.0 49.0 35.0 20.0 14.0

CELF2 3.02299898575311 5.0490806136106e-05 0.000176578996238519 17.0453649137051 12.3530555568088 31.8506400012958 0 3.31540871598484 4.28538557739602 up 16.0 12.0 30.0 0.0 4.0 4.0

NTM 5.76319976148322 5.04928369409318e-05 0.000176578996238519 8.52268245685255 14.4118981496103 5.30844000021597 0 0 0 up 8.0 14.0 5.0 0.0 0.0 0.0

PDX1 5.41253922252525 5.06357154729779e-05 0.000177042109414292 10.6533530710657 11.3236342604081 21.2337600008639 0 0.82885217899621 0 up 10.0 11.0 20.0 0.0 1.0 0.0

BFSP1 -1.06031600265881 5.10985513827604e-05 0.00017851298420787 42.6134122842627 32.9414814848234 35.0357040014254 74.7200050396524 74.596696109659 81.4223259705244 down 40.0 32.0 33.0 79.0 90.0 76.0

XKR4 3.68623306580694 5.15182370394925e-05 0.000179830812991758 13.8493589923854 17.5001620388124 18.0486960007343 0.945822848603194 0 3.21403918304702 up 13.0 17.0 17.0 1.0 0.0 3.0

SLC27A6 2.45606897504222 5.25880827688613e-05 0.000183338697013724 20.2413708350248 17.5001620388124 29.7272640012094 3.78329139441278 3.31540871598484 5.35673197174503 up 19.0 17.0 28.0 4.0 4.0 5.0

MAB21L1 1.27054806883844 5.25881185826259e-05 0.000183338697013724 54.332100662435 62.7946990804447 63.7012800025917 26.4830397608894 31.496382801856 16.0701959152351 up 51.0 61.0 60.0 28.0 38.0 15.0

SERPINE3 2.6167797038113 5.40911997146741e-05 0.000188307629093009 24.5027120634511 24.7061111136176 13.8019440005615 2.83746854580958 4.14426089498105 3.21403918304702 up 23.0 24.0 13.0 3.0 5.0 3.0

SOX2 5.3733018460181 5.41059212942536e-05 0.00018832017808191 10.6533530710657 15.441319446011 15.9253200006479 0.945822848603194 0 0 up 10.0 15.0 15.0 1.0 0.0 0.0

PRRX2 -1.48445609139262 5.45097236235203e-05 0.000189686671771248 12.7840236852788 15.441319446011 21.2337600008639 46.3453195815565 38.9560524128219 53.5673197174503 down 12.0 15.0 20.0 49.0 47.0 50.0

LUM -2.65284131390959 5.55741341249221e-05 0.000193231903135505 2.13067061421314 6.17652777840439 2.12337600008639 26.4830397608894 23.2078610118939 16.0701959152351 down 2.0 6.0 2.0 28.0 28.0 15.0

KCNH2 4.69212958552419 5.70100831187455e-05 0.000197859210823919 12.7840236852788 20.5884259280146 15.9253200006479 0 0 2.14269278869801 up 12.0 20.0 15.0 0.0 0.0 2.0

FRRS1 1.16575726954963 5.70234394615901e-05 0.000197865027545952 70.3121302690335 60.7358564876432 70.0714080028508 37.8329139441278 23.2078610118939 28.9263526474231 up 66.0 59.0 66.0 40.0 28.0 27.0

LOC101927239 -5.64061530801863 5.71978629636431e-05 0.000198429612656996 0 0 0 6.62075994022236 11.6039305059469 10.7134639434901 down 0.0 0.0 0.0 7.0 14.0 10.0

EGLN3 1.68403227329995 5.73971295140079e-05 0.000198998643873781 54.332100662435 27.7943750028198 39.2824560015982 17.0248112748575 9.11737396895832 11.7848103378391 up 51.0 27.0 37.0 18.0 11.0 11.0

RUNDC3B 1.58625947258293 5.7874510288147e-05 0.000200530620634324 37.2867357487299 43.2356944488308 57.3311520023325 15.1331655776511 22.3790088328977 7.49942476044304 up 35.0 42.0 54.0 16.0 27.0 7.0

NPBWR1 2.15510279752928 5.79154718939891e-05 0.000200631512041754 28.7640532918774 27.7943750028198 22.2954480009071 2.83746854580958 8.28852178996211 6.42807836609403 up 27.0 27.0 21.0 3.0 10.0 6.0

LOC389895 3.70779360372386 5.81603020082304e-05 0.000201397284550168 20.2413708350248 11.3236342604081 16.9870080006911 1.89164569720639 1.65770435799242 0 up 19.0 11.0 16.0 2.0 2.0 0.0

CLDN16 -2.4485153914419 5.8534448285875e-05 0.0002026514518624 4.26134122842627 6.17652777840439 2.12337600008639 20.8081026692703 22.3790088328977 25.7123134643761 down 4.0 6.0 2.0 22.0 27.0 24.0

DCHS2 2.68357130851268 5.871584556051e-05 0.000203154906820882 24.5027120634511 19.5590046316139 15.9253200006479 1.89164569720639 4.14426089498105 3.21403918304702 up 23.0 19.0 15.0 2.0 5.0 3.0

SLC22A2 -1.16386934331129 5.89935619393187e-05 0.000204074114611407 25.5680473705576 36.0297453740256 26.5422000010799 60.5326623106044 69.6235830356817 67.4948228439873 down 24.0 35.0 25.0 64.0 84.0 63.0

KBTBD8 1.52192266147124 6.08683445149433e-05 0.000210044765957046 41.5480769771562 40.1474305596286 40.3441440016414 13.2415198804447 12.4327826849432 17.1415423095841 up 39.0 39.0 38.0 14.0 15.0 16.0

PLTP 1.13014416757068 6.17045627128879e-05 0.000212800344535082 64.9854537335007 97.7950231580696 72.1947840029372 38.778736792731 25.6944175488825 43.9252021683092 up 61.0 95.0 68.0 41.0 31.0 41.0

HPD 2.42120278811192 6.24099115799064e-05 0.000215057750643534 19.1760355279182 24.7061111136176 21.2337600008639 4.72911424301597 4.14426089498105 3.21403918304702 up 18.0 24.0 20.0 5.0 5.0 3.0

RAPGEF5 -2.27172895922492 6.60611328888201e-05 0.000227039073064843 4.26134122842627 12.3530555568088 1.06168800004319 22.6997483664767 33.1540871598484 29.9976990417721 down 4.0 12.0 1.0 24.0 40.0 28.0

HLA-DPA1 2.82408796653594 6.71584581453292e-05 0.00023052974285347 20.2413708350248 21.6178472244154 22.2954480009071 0 7.45966961096589 1.07134639434901 up 19.0 21.0 21.0 0.0 9.0 1.0

JAK3 1.19888705806571 7.18171355632167e-05 0.000245328146086084 54.332100662435 56.6181713020403 73.2564720029804 29.320508306699 22.3790088328977 28.9263526474231 up 51.0 55.0 69.0 31.0 27.0 27.0

ENTPD2 -1.17482683079667 7.20765918386798e-05 0.000246115191644273 31.9600592131971 28.8237962992205 22.2954480009071 66.2075994022236 61.3350612457196 59.9953980835443 down 30.0 28.0 21.0 70.0 74.0 56.0

NIPAL2 1.41815711522971 7.21611478646182e-05 0.000246326471121126 66.0507890406073 37.0591666704264 50.9610240020733 15.1331655776511 24.8655653698863 17.1415423095841 up 62.0 36.0 48.0 16.0 30.0 16.0

FBXO15 2.0479829644313 7.31295684725562e-05 0.000249358874783636 40.4827416700496 29.8532175956212 21.2337600008639 6.62075994022236 3.31540871598484 12.8561567321881 up 38.0 29.0 20.0 7.0 4.0 12.0

ANXA10 -1.87523861275918 7.47833286804729e-05 0.000254639113135118 11.7186883781723 4.11768518560293 11.6785680004751 24.5913940636831 36.4694958758333 39.6398165909132 down 11.0 4.0 11.0 26.0 44.0 37.0

CYP1A1 -3.32506239767596 7.64944468045521e-05 0.000260047346752782 1.06533530710657 3.0882638892022 1.06168800004319 19.8622798206671 11.6039305059469 21.4269278869801 down 1.0 3.0 1.0 21.0 14.0 20.0

MAGEB17 -5.25093013141017 7.86368616110837e-05 0.000266848644970304 1.06533530710657 0 0 19.8622798206671 12.4327826849432 10.7134639434901 down 1.0 0.0 0.0 21.0 15.0 10.0

FAM25A -5.27100151867275 8.10634285887692e-05 0.000274752797177621 0 0 1.06168800004319 13.2415198804447 8.28852178996211 22.4982742813291 down 0.0 0.0 1.0 14.0 10.0 21.0

STAMBPL1 1.02195520262199 8.13869499329749e-05 0.00027579414520196 100.141518868017 84.41254630486 102.98373600419 42.5620281871437 33.1540871598484 67.4948228439873 up 94.0 82.0 97.0 45.0 40.0 63.0

JHDM1D-AS1 -1.07460127807216 8.58080504303352e-05 0.000289848130466576 29.8293885989839 31.9120601884227 43.529208001771 83.2324106770811 68.7947308566855 69.6375156326853 down 28.0 31.0 41.0 88.0 83.0 65.0

TM4SF4 1.29366135518794 8.90302659107812e-05 0.000299895149041287 40.4827416700496 66.9123842660476 57.3311520023325 19.8622798206671 25.6944175488825 21.4269278869801 up 38.0 65.0 54.0 21.0 31.0 20.0

PRRT2 1.18311811479127 9.05571678280816e-05 0.000304674959882364 56.4627712766481 103.971550936474 72.1947840029372 35.9412682469214 43.1003133078029 22.4982742813291 up 53.0 101.0 68.0 38.0 52.0 21.0

TNFRSF9 1.23625739811559 9.06035120811094e-05 0.000304770352393565 53.2667653553284 64.8535416732461 48.8376480019869 23.6455712150799 26.5232697278787 20.3555814926311 up 50.0 63.0 46.0 25.0 32.0 19.0

GUCY1B2 1.09257854149376 9.20579265483714e-05 0.000309355539720177 89.4881657969518 91.6184953796652 72.1947840029372 30.2663311553022 57.1908003507385 29.9976990417721 up 84.0 89.0 68.0 32.0 69.0 28.0

FREM2 3.34863390218335 9.26339144077795e-05 0.000310982661527504 19.1760355279182 10.2942129640073 19.1103840007775 0.945822848603194 0.82885217899621 3.21403918304702 up 18.0 10.0 18.0 1.0 1.0 3.0

KRT4 -5.56307435700195 9.26980796470627e-05 0.000311136411575439 0 0 0 14.1873427290479 5.80196525297347 7.49942476044304 down 0.0 0.0 0.0 15.0 7.0 7.0

DLGAP1-AS2 -1.1148180215033 9.2771007181975e-05 0.000311319505083728 31.9600592131971 26.764953706419 45.6525840018574 83.2324106770811 82.8852178996211 58.9240516891953 down 30.0 26.0 43.0 88.0 100.0 55.0

LRRC66 -1.46154160134353 9.28182461206812e-05 0.000311416337746948 12.7840236852788 24.7061111136176 12.7402560005183 52.0202566731757 39.7849045918181 47.1392413513562 down 12.0 24.0 12.0 55.0 48.0 44.0

PSG10P 2.65818392003804 9.3745545856543e-05 0.000314402998774468 24.5027120634511 17.5001620388124 16.9870080006911 5.67493709161917 2.48655653698863 1.07134639434901 up 23.0 17.0 16.0 6.0 3.0 1.0

TGFBR3L 1.4210119253853 9.40775477276379e-05 0.000315391580943239 41.5480769771562 66.9123842660476 46.7142720019006 17.9706341234607 27.3521219068749 11.7848103378391 up 39.0 65.0 44.0 19.0 33.0 11.0

DMRT1 -4.55123967044168 9.48747242884307e-05 0.000317881720068773 2.13067061421314 0 0 17.0248112748575 13.2616348639394 19.2842350982821 down 2.0 0.0 0.0 18.0 16.0 18.0

ADAMTS10 1.3323261898389 9.48766219992748e-05 0.000317881720068773 37.2867357487299 65.8829629696469 57.3311520023325 17.0248112748575 23.2078610118939 23.5696206756781 up 35.0 64.0 54.0 18.0 28.0 22.0

SPOCD1 2.45522811826788 9.49904383769752e-05 0.000318200123809761 20.2413708350248 27.7943750028198 18.0486960007343 0.945822848603194 6.63081743196968 4.28538557739602 up 19.0 27.0 17.0 1.0 8.0 4.0

SYT7 -1.10409002108253 9.69270427335538e-05 0.00032436667402339 29.8293885989839 36.0297453740256 33.9740160013822 58.6410166133981 87.8583309735983 67.4948228439873 down 28.0 35.0 32.0 62.0 106.0 63.0

HHIPL1 1.72753912182345 0.000101818263220334 0.000339461007469487 24.5027120634511 50.4416435236359 32.912328001339 13.2415198804447 11.6039305059469 7.49942476044304 up 23.0 49.0 31.0 14.0 14.0 7.0

CD274 1.32014155850392 0.000101862670338336 0.000339542234461121 54.332100662435 50.4416435236359 47.7759600019437 19.8622798206671 26.5232697278787 13.9275031265371 up 51.0 49.0 45.0 21.0 32.0 13.0

SMIM6 1.18510497061314 0.000104039322302396 0.00034652499406264 68.1814596548204 52.5004861164373 59.4545280024189 21.7539255178735 32.3252349808522 24.6409670700271 up 64.0 51.0 56.0 23.0 39.0 23.0

MEF2C 1.5828066772073 0.0001047338054585 0.000348700995374801 60.7241125050744 29.8532175956212 37.1590800015118 17.9706341234607 9.94622614795453 14.9988495208861 up 57.0 29.0 35.0 19.0 12.0 14.0

RNF157-AS1 2.38495635125596 0.000105121271782532 0.000349784785699489 19.1760355279182 23.6766898172168 20.1720720008207 2.83746854580958 5.80196525297347 3.21403918304702 up 18.0 23.0 19.0 3.0 7.0 3.0

CEBPA -1.03162711718715 0.000106422992830124 0.000353613033980689 29.8293885989839 58.6770138948417 39.2824560015982 91.7448163145099 76.2544004676514 94.2784827027125 down 28.0 57.0 37.0 97.0 92.0 88.0

ISG20 -2.13067802566785 0.000106456383590215 0.000353613033980689 7.45734714974598 2.05884259280146 8.49350400034555 29.320508306699 21.5501566539015 27.8550062530741 down 7.0 2.0 8.0 31.0 26.0 26.0

GNAT2 1.94939499555185 0.00010698624623039 0.000355292493852736 35.1560651345168 25.7355324100183 22.2954480009071 9.45822848603194 6.63081743196968 5.35673197174503 up 33.0 25.0 21.0 10.0 8.0 5.0

LRRC10B -3.91517996054689 0.000112203878264103 0.000371846230129299 1.06533530710657 1.02942129640073 1.06168800004319 12.2956970318415 23.2078610118939 11.7848103378391 down 1.0 1.0 1.0 13.0 28.0 11.0

CD99P1 -1.2591575731543 0.000113448232058301 0.000375500791533336 14.914694299492 33.9709027812242 25.4805120010367 52.9660795217789 57.1908003507385 68.5661692383363 down 14.0 33.0 24.0 56.0 69.0 64.0

MIR762HG -1.37354691717264 0.000114964618212764 0.000380371217289886 21.3067061421314 23.6766898172168 11.6785680004751 41.6162053385406 53.8753916347537 51.4246269287523 down 20.0 23.0 11.0 44.0 65.0 48.0

LPAR3 -1.28113554577793 0.000116777093990375 0.000385765205186612 33.0253945203036 28.8237962992205 16.9870080006911 70.9367136452396 77.9121048256438 41.7825093796112 down 31.0 28.0 16.0 75.0 94.0 39.0

C10orf95 1.28942497312962 0.000118937950948327 0.000392520726488344 52.2014300482219 67.9418055624483 41.4058320016846 25.5372169122862 17.4058957589204 23.5696206756781 up 49.0 66.0 39.0 27.0 21.0 22.0

JPH3 -1.95831527356573 0.000120238812756031 0.00039604228817561 6.39201184263941 11.3236342604081 5.30844000021597 18.9164569720639 36.4694958758333 34.2830846191682 down 6.0 11.0 5.0 20.0 44.0 32.0

PDGFRA -2.36893987049188 0.000123235180305993 0.00040496681947304 4.26134122842627 6.17652777840439 2.12337600008639 19.8622798206671 20.7213044749053 24.6409670700271 down 4.0 6.0 2.0 21.0 25.0 23.0

FGB 2.38971753224593 0.000123492563910553 0.000405655232236721 19.1760355279182 21.6178472244154 23.3571360009503 0.945822848603194 4.97311307397726 6.42807836609403 up 18.0 21.0 22.0 1.0 6.0 6.0

NPFFR2 -1.72175158194712 0.000126786152073906 0.000415829122193992 8.52268245685255 11.3236342604081 10.6168800004319 24.5913940636831 41.4426089498105 34.2830846191682 down 8.0 11.0 10.0 26.0 50.0 32.0

ITK 5.18641854094046 0.000130847726531127 0.000428486465771754 17.0453649137051 8.23537037120586 11.6785680004751 0 0.82885217899621 0 up 16.0 8.0 11.0 0.0 1.0 0.0

TMEM217 1.35309255902635 0.000131072323258914 0.000429138994202937 37.2867357487299 50.4416435236359 70.0714080028508 21.7539255178735 14.9193392219318 25.7123134643761 up 35.0 49.0 66.0 23.0 18.0 24.0

TCAP -1.31047815714193 0.000131524166563787 0.000430535146201072 31.9600592131971 15.441319446011 30.7889520012526 84.1782335256843 67.9658786776893 40.7111629852622 down 30.0 15.0 29.0 89.0 82.0 38.0

HHIP-AS1 -1.93814099456805 0.000133217087492321 0.000435655880718132 10.6533530710657 4.11768518560293 7.43181600030236 23.6455712150799 33.1540871598484 27.8550062530741 down 10.0 4.0 7.0 25.0 40.0 26.0

OR10H1 2.08140147910065 0.000133258810000157 0.000435708211040853 22.3720414492379 31.9120601884227 36.0973920014686 9.45822848603194 0.82885217899621 11.7848103378391 up 21.0 31.0 34.0 10.0 1.0 11.0

PCAT7 1.1362423406954 0.000134165692062747 0.000438250448041445 56.4627712766481 68.9712268588491 55.2077760022461 25.5372169122862 30.6675306228598 25.7123134643761 up 53.0 67.0 52.0 27.0 37.0 24.0

XKR8 -3.47285718163056 0.000139488958864246 0.000454586949434461 1.06533530710657 2.05884259280146 1.06168800004319 12.2956970318415 18.2347479379166 16.0701959152351 down 1.0 2.0 1.0 13.0 22.0 15.0

HOXC13 1.69784157965628 0.000139824020084703 0.000455591248362159 27.6987179847708 41.1768518560293 43.529208001771 8.51240563742875 6.63081743196968 20.3555814926311 up 26.0 40.0 41.0 9.0 8.0 19.0

DMRT2 3.56076344822647 0.000143737515531139 0.00046780278829923 14.914694299492 17.5001620388124 11.6785680004751 2.83746854580958 0.82885217899621 0 up 14.0 17.0 11.0 3.0 1.0 0.0

FST 2.84400059109546 0.000145993098854838 0.000474596640683354 15.9800296065985 16.4707407424117 22.2954480009071 5.67493709161917 0 2.14269278869801 up 15.0 16.0 21.0 6.0 0.0 2.0

SDK2 -1.36347741278661 0.000154205555890918 0.000499759348917572 20.2413708350248 14.4118981496103 16.9870080006911 47.2911424301597 40.6137567708143 44.9965485626582 down 19.0 14.0 16.0 50.0 49.0 42.0

LKAAEAR1 -2.33736259629409 0.000155344363994431 0.000503331713002525 3.19600592131971 8.23537037120586 2.12337600008639 24.5913940636831 15.748191400928 28.9263526474231 down 3.0 8.0 2.0 26.0 19.0 27.0

LINC01444 4.49871751044966 0.000155458504977079 0.000503530989352144 14.914694299492 11.3236342604081 15.9253200006479 0.945822848603194 0.82885217899621 0 up 14.0 11.0 15.0 1.0 1.0 0.0

C4BPB -5.44502288789868 0.000164381333981637 0.00053040377098075 0 0 0 8.51240563742875 4.14426089498105 12.8561567321881 down 0.0 0.0 0.0 9.0 5.0 12.0

GFRA2 3.93631653396274 0.00016679005611301 0.000537254905981059 14.914694299492 12.3530555568088 14.8636320006047 0 2.48655653698863 0 up 14.0 12.0 14.0 0.0 3.0 0.0

LINC01152 5.46994234370493 0.000172306320190284 0.000554285807828221 6.39201184263941 10.2942129640073 6.37012800025917 0 0 0 up 6.0 10.0 6.0 0.0 0.0 0.0

MYBPH 5.47443898603937 0.000173449821952025 0.000557752464667295 5.32667653553284 7.20594907480513 10.6168800004319 0 0 0 up 5.0 7.0 10.0 0.0 0.0 0.0

STXBP6 -5.43418412935495 0.000174640714442296 0.000561262322643236 0 0 0 4.72911424301597 6.63081743196968 13.9275031265371 down 0.0 0.0 0.0 5.0 8.0 13.0

EVI2B 2.06590050632107 0.000174833754068316 0.000561776136934233 18.1107002208117 25.7355324100183 26.5422000010799 4.72911424301597 6.63081743196968 5.35673197174503 up 17.0 25.0 25.0 5.0 8.0 5.0

HCG4 -1.46347758960505 0.000177730988929732 0.000570328272867901 14.914694299492 15.441319446011 14.8636320006047 49.1827881273661 29.0098262648674 47.1392413513562 down 14.0 15.0 14.0 52.0 35.0 44.0

PDE6G 2.06788560412166 0.000178971279206011 0.000573982103322573 19.1760355279182 21.6178472244154 29.7272640012094 4.72911424301597 6.63081743196968 5.35673197174503 up 18.0 21.0 28.0 5.0 8.0 5.0

ANTXRL -3.82577433684201 0.000180765531762917 0.000578859755777658 2.13067061421314 1.02942129640073 0 18.9164569720639 9.94622614795453 16.0701959152351 down 2.0 1.0 0.0 20.0 12.0 15.0

LOC100506178 -5.0471911581113 0.000180851755111128 0.000579026409295504 0 1.02942129640073 0 8.51240563742875 14.9193392219318 13.9275031265371 down 0.0 1.0 0.0 9.0 18.0 13.0

LGI3 3.2978352974534 0.000182236286359405 0.000582798318091055 19.1760355279182 12.3530555568088 13.8019440005615 0 3.31540871598484 1.07134639434901 up 18.0 12.0 13.0 0.0 4.0 1.0

SSTR5 -5.44743095741071 0.000183076342422271 0.000585353806711308 0 0 0 9.45822848603194 12.4327826849432 3.21403918304702 down 0.0 0.0 0.0 10.0 15.0 3.0

LOC101928034 1.10193179818146 0.000188109242263302 0.000599768598520673 56.4627712766481 80.2948611192571 54.1460880022029 29.320508306699 30.6675306228598 28.9263526474231 up 53.0 78.0 51.0 31.0 37.0 27.0

CTLA4 -4.5084697319913 0.000193764965885118 0.000616524891452648 0 0 2.12337600008639 5.67493709161917 15.748191400928 26.7836598587251 down 0.0 0.0 2.0 6.0 19.0 25.0

PSG7 1.65730164945049 0.000195802011118232 0.000622772449932942 33.0253945203036 32.9414814848234 28.6655760011662 6.62075994022236 11.6039305059469 11.7848103378391 up 31.0 32.0 27.0 7.0 14.0 11.0

LINC01133 -5.39882366511231 0.000204498334286923 0.000648362676926909 0 0 0 7.56658278882556 12.4327826849432 4.28538557739602 down 0.0 0.0 0.0 8.0 15.0 4.0

C5orf49 3.46142076612537 0.000205255239395161 0.000650518943939014 14.914694299492 14.4118981496103 12.7402560005183 1.89164569720639 0 2.14269278869801 up 14.0 14.0 12.0 2.0 0.0 2.0

PHF24 -2.10222172207058 0.000205794150833973 0.000651982965237985 4.26134122842627 8.23537037120586 4.24675200017278 31.2121540039054 21.5501566539015 19.2842350982821 down 4.0 8.0 4.0 33.0 26.0 18.0

ASCL5 1.61313136688608 0.000207910058249523 0.000658440154561024 43.6787475913693 26.764953706419 30.7889520012526 9.45822848603194 9.94622614795453 13.9275031265371 up 41.0 26.0 29.0 10.0 12.0 13.0

TMC3-AS1 5.4072108163401 0.000214336690236704 0.000677652768310892 7.45734714974598 7.20594907480513 7.43181600030236 0 0 0 up 7.0 7.0 7.0 0.0 0.0 0.0

HLA-DQB1 5.05032351585117 0.000215601725626201 0.000681143832918286 8.52268245685255 13.3824768532095 11.6785680004751 0.945822848603194 0 0 up 8.0 13.0 11.0 1.0 0.0 0.0

DUSP19 -1.07532197897681 0.000216910566137472 0.000684640393211995 42.6134122842627 35.0003240776249 30.7889520012526 79.4491192826683 96.1468527635604 51.4246269287523 down 40.0 34.0 29.0 84.0 116.0 48.0

ELMO1 1.13294617173571 0.000220606474674121 0.000695657796161507 56.4627712766481 61.7652777840439 61.5779040025053 18.9164569720639 34.8117915178408 27.8550062530741 up 53.0 60.0 58.0 20.0 42.0 26.0

LOC101928233 -4.41944697499454 0.000221885453023231 0.000699040277889814 0 2.05884259280146 0 8.51240563742875 14.0904870429356 22.4982742813291 down 0.0 2.0 0.0 9.0 17.0 21.0

SH3BGR 1.37801857364852 0.000223316814549907 0.000702896105253702 59.6587771979678 35.0003240776249 52.0227120021165 28.3746854580958 14.0904870429356 13.9275031265371 up 56.0 34.0 49.0 30.0 17.0 13.0

ISM2 -1.38256643525111 0.000226190914476371 0.000711413681995865 15.9800296065985 15.441319446011 16.9870080006911 43.5078510357469 48.9022785607764 33.2117382248192 down 15.0 15.0 16.0 46.0 59.0 31.0

KCND3 -2.33374243755926 0.000227077249475584 0.000714068796383217 7.45734714974598 1.02942129640073 5.30844000021597 34.049622549715 14.9193392219318 20.3555814926311 down 7.0 1.0 5.0 36.0 18.0 19.0

THRB-AS1 2.85914008126109 0.000234292624238848 0.000735802197739355 15.9800296065985 18.5295833352132 12.7402560005183 0.945822848603194 3.31540871598484 2.14269278869801 up 15.0 18.0 12.0 1.0 4.0 2.0

MIR4478 -5.38688806402886 0.000240985839332832 0.000755701613901919 0 0 0 5.67493709161917 14.0904870429356 4.28538557739602 down 0.0 0.0 0.0 6.0 17.0 4.0

MIR503HG 1.06549248728218 0.000242573844515772 0.000760259190767285 49.0054241269022 72.0594907480513 78.5649120031964 31.2121540039054 33.9829393388446 29.9976990417721 up 46.0 70.0 74.0 33.0 41.0 28.0

NOVA1 1.27500281529094 0.000242622561199204 0.000760271214708567 83.0961539543123 37.0591666704264 67.9480320027644 16.0789884262543 31.496382801856 29.9976990417721 up 78.0 36.0 64.0 17.0 38.0 28.0

SRPK3 1.66978013916286 0.000248282766065322 0.000777145243375195 29.8293885989839 43.2356944488308 27.6038880011231 16.0789884262543 9.94622614795453 5.35673197174503 up 28.0 42.0 26.0 17.0 12.0 5.0

LOC100270746 -1.36450415729606 0.000255399384708415 0.000797358197007105 20.2413708350248 11.3236342604081 20.1720720008207 43.5078510357469 36.4694958758333 53.5673197174503 down 19.0 11.0 19.0 46.0 44.0 50.0

NR2E1 1.52033233417116 0.000255773806311666 0.000798380003486204 39.417406362943 37.0591666704264 29.7272640012094 14.1873427290479 8.28852178996211 14.9988495208861 up 37.0 36.0 28.0 15.0 10.0 14.0

TLL1 -2.66984104958774 0.000262295725377694 0.000817532582869944 1.06533530710657 2.05884259280146 5.30844000021597 17.0248112748575 22.3790088328977 13.9275031265371 down 1.0 2.0 5.0 18.0 27.0 13.0

DDIT4L -3.76147628655364 0.000262880271336463 0.000819053116873216 0 0 3.18506400012958 14.1873427290479 15.748191400928 12.8561567321881 down 0.0 0.0 3.0 15.0 19.0 12.0

C1orf228 2.89462347946352 0.000265870977571237 0.000827762242245314 15.9800296065985 15.441319446011 19.1103840007775 0 0.82885217899621 6.42807836609403 up 15.0 15.0 18.0 0.0 1.0 6.0

TNFAIP6 -4.37728701246833 0.000266771503329861 0.000830239872146943 2.13067061421314 0 0 22.6997483664767 12.4327826849432 8.57077115479204 down 2.0 0.0 0.0 24.0 15.0 8.0

MARCH4 1.10558512525741 0.000276768547433031 0.00085884945842014 49.0054241269022 67.9418055624483 57.3311520023325 24.5913940636831 25.6944175488825 31.0690454361212 up 46.0 66.0 54.0 26.0 31.0 29.0

TMPRSS4 -5.40953539909186 0.000280283151129219 0.000869437205663609 0 0 0 1.89164569720639 14.0904870429356 8.57077115479204 down 0.0 0.0 0.0 2.0 17.0 8.0

ZP1 3.42197002434545 0.000284655005611398 0.000882190961408174 17.0453649137051 11.3236342604081 11.6785680004751 0.945822848603194 1.65770435799242 1.07134639434901 up 16.0 11.0 11.0 1.0 2.0 1.0

INPP5J 1.34727871483281 0.000285196192985532 0.000883383344153393 31.9600592131971 50.4416435236359 44.5908960018142 15.1331655776511 15.748191400928 19.2842350982821 up 30.0 49.0 42.0 16.0 19.0 18.0

TJP3 -2.51191018899719 0.000286217995030028 0.000886062287245738 5.32667653553284 5.14710648200366 0 22.6997483664767 24.0367131908901 12.8561567321881 down 5.0 5.0 0.0 24.0 29.0 12.0

CGB 1.63564559363396 0.000288677278153606 0.000893022843667292 25.5680473705576 36.0297453740256 28.6655760011662 9.45822848603194 9.94622614795453 9.64211754914105 up 24.0 35.0 27.0 10.0 12.0 9.0

CSF2 1.71534985944731 0.000291775259134765 0.000901783048666834 29.8293885989839 28.8237962992205 24.4188240009935 9.45822848603194 8.28852178996211 7.49942476044304 up 28.0 28.0 23.0 10.0 10.0 7.0

MRGPRF -5.3457579153076 0.000298378925033476 0.000920513383731031 0 0 0 4.72911424301597 4.14426089498105 14.9988495208861 down 0.0 0.0 0.0 5.0 5.0 14.0

FES -2.04225557767289 0.000298980094323959 0.000922200072441343 4.26134122842627 6.17652777840439 6.37012800025917 17.0248112748575 26.5232697278787 25.7123134643761 down 4.0 6.0 6.0 18.0 32.0 24.0

CYGB 2.16084346275557 0.000299123241698712 0.000922305736144191 21.3067061421314 23.6766898172168 19.1103840007775 6.62075994022236 1.65770435799242 6.42807836609403 up 20.0 23.0 18.0 7.0 2.0 6.0

KRT42P -5.33695033514257 0.000301080074592466 0.000927663962094647 0 0 0 14.1873427290479 4.97311307397726 4.28538557739602 down 0.0 0.0 0.0 15.0 6.0 4.0

TCAM1P 1.40711953589999 0.000308224295793381 0.000947951991782837 33.0253945203036 43.2356944488308 36.0973920014686 13.2415198804447 13.2616348639394 16.0701959152351 up 31.0 42.0 34.0 14.0 16.0 15.0

C10orf55 -1.3325223338452 0.000312876187794693 0.000961037646644081 12.7840236852788 33.9709027812242 14.8636320006047 53.9119023703821 42.2714611288067 59.9953980835443 down 12.0 33.0 14.0 57.0 51.0 56.0

LINC00648 2.87186662764507 0.000318283035152767 0.000976052609610404 18.1107002208117 22.6472685208161 7.43181600030236 0.945822848603194 2.48655653698863 3.21403918304702 up 17.0 22.0 7.0 1.0 3.0 3.0

PAX2 5.34165263180384 0.000318773323711474 0.000977379204284591 8.52268245685255 4.11768518560293 8.49350400034555 0 0 0 up 8.0 4.0 8.0 0.0 0.0 0.0

ANK1 -1.22393240349031 0.000322220923037212 0.00098705649841779 18.1107002208117 21.6178472244154 21.2337600008639 53.9119023703821 45.5868698447916 42.8538557739602 down 17.0 21.0 20.0 57.0 55.0 40.0

PROZ 1.45709738921886 0.000329415588716518 0.00100800218079079 33.0253945203036 52.5004861164373 29.7272640012094 11.3498741832383 16.5770435799242 13.9275031265371 up 31.0 51.0 28.0 12.0 20.0 13.0

PLXNC1 -1.19160105292857 0.000335653692600202 0.00102486906140004 27.6987179847708 20.5884259280146 20.1720720008207 43.5078510357469 62.992765603712 49.2819341400542 down 26.0 20.0 19.0 46.0 76.0 46.0

POU5F1B -3.08181340192364 0.000337696877778325 0.00102980830055172 3.19600592131971 1.02942129640073 1.06168800004319 12.2956970318415 18.2347479379166 13.9275031265371 down 3.0 1.0 1.0 13.0 22.0 13.0

CNTN1 -4.26845681507604 0.000340232174948608 0.00103660666252328 1.06533530710657 0 1.06168800004319 14.1873427290479 16.5770435799242 9.64211754914105 down 1.0 0.0 1.0 15.0 20.0 9.0

ARHGAP24 -1.88918528751849 0.00034056047774612 0.00103742033681339 8.52268245685255 7.20594907480513 4.24675200017278 23.6455712150799 25.6944175488825 24.6409670700271 down 8.0 7.0 4.0 25.0 31.0 23.0

TNNC1 -4.331476468658 0.000343943574460027 0.00104678479183486 0 2.05884259280146 0 6.62075994022236 17.4058957589204 18.2128887039331 down 0.0 2.0 0.0 7.0 21.0 17.0

ASB9 1.30702407313552 0.000348606999581323 0.00106002559197767 35.1560651345168 48.3828009308344 47.7759600019437 13.2415198804447 22.3790088328977 17.1415423095841 up 33.0 47.0 45.0 14.0 27.0 16.0

HAND2 2.37553809139524 0.000350260450587108 0.00106486217389548 15.9800296065985 28.8237962992205 14.8636320006047 5.67493709161917 0.82885217899621 5.35673197174503 up 15.0 28.0 14.0 6.0 1.0 5.0

LOC100270804 -3.3577693575884 0.000352367463790568 0.00107088353724654 1.06533530710657 1.02942129640073 2.12337600008639 17.9706341234607 17.4058957589204 7.49942476044304 down 1.0 1.0 2.0 19.0 21.0 7.0

RAP2C-AS1 1.33135950571119 0.000353318410910522 0.00107358096517027 33.0253945203036 51.4710648200366 41.4058320016846 15.1331655776511 14.9193392219318 20.3555814926311 up 31.0 50.0 39.0 16.0 18.0 19.0

GOLGA7B 4.95377202233254 0.000354908182656305 0.00107802485461678 6.39201184263941 15.441319446011 9.55519200038875 0 0.82885217899621 0 up 6.0 15.0 9.0 0.0 1.0 0.0

PI3 -2.61722236888182 0.000367526923388251 0.00111316039374164 3.19600592131971 4.11768518560293 1.06168800004319 14.1873427290479 14.0904870429356 23.5696206756781 down 3.0 4.0 1.0 15.0 17.0 22.0

LMOD1 1.64298493508422 0.000390616422195499 0.0011784580929638 24.5027120634511 37.0591666704264 29.7272640012094 14.1873427290479 6.63081743196968 8.57077115479204 up 23.0 36.0 28.0 15.0 8.0 8.0

MCF2L2 1.3419921177267 0.000390954586265413 0.00117905833920885 40.4827416700496 32.9414814848234 61.5779040025053 22.6997483664767 17.4058957589204 12.8561567321881 up 38.0 32.0 58.0 24.0 21.0 12.0

LOC388282 -1.7434889394644 0.000402071551458951 0.00121150695156788 3.19600592131971 13.3824768532095 9.55519200038875 27.4288626094926 27.3521219068749 33.2117382248192 down 3.0 13.0 9.0 29.0 33.0 31.0

DLX6-AS1 5.26156056379852 0.000407979766476576 0.00122843534378123 6.39201184263941 7.20594907480513 6.37012800025917 0 0 0 up 6.0 7.0 6.0 0.0 0.0 0.0

VAX1 2.96542609944639 0.000408226832268936 0.00122896082079896 17.0453649137051 12.3530555568088 15.9253200006479 0 0.82885217899621 5.35673197174503 up 16.0 12.0 15.0 0.0 1.0 5.0

VWA7 1.07286426008006 0.000412856540793965 0.00124201559244357 49.0054241269022 70.0006481552498 74.3181600030236 23.6455712150799 39.7849045918181 27.8550062530741 up 46.0 68.0 70.0 25.0 48.0 26.0

C1GALT1C1L 1.53563975936836 0.000418104735756385 0.00125624232417757 30.8947239060905 24.7061111136176 49.8993360020301 9.45822848603194 14.9193392219318 11.7848103378391 up 29.0 24.0 47.0 10.0 18.0 11.0

CDKL2 2.52849615446338 0.000420912524053057 0.00126355806440879 21.3067061421314 9.26479166760659 23.3571360009503 5.67493709161917 2.48655653698863 1.07134639434901 up 20.0 9.0 22.0 6.0 3.0 1.0

FRMPD1 1.98021692350221 0.000421346442425051 0.0012645949123941 28.7640532918774 19.5590046316139 18.0486960007343 4.72911424301597 6.63081743196968 5.35673197174503 up 27.0 19.0 17.0 5.0 8.0 5.0

ITGA2B -1.02693078495399 0.00042190962542076 0.00126520606383921 27.6987179847708 27.7943750028198 36.0973920014686 69.9908907966364 59.6773568877272 56.7813589004973 down 26.0 27.0 34.0 74.0 72.0 53.0

LOC100129203 5.2595576493576 0.000424279051103271 0.0012716360802706 5.32667653553284 8.23537037120586 6.37012800025917 0 0 0 up 5.0 8.0 6.0 0.0 0.0 0.0

GSC -1.17875057228384 0.000424587727033827 0.00127233612169698 27.6987179847708 22.6472685208161 25.4805120010367 48.2369652787629 43.1003133078029 81.4223259705244 down 26.0 22.0 24.0 51.0 52.0 76.0

APOBEC3G -2.31747512641805 0.00042528735119324 0.00127398191498028 1.06533530710657 6.17652777840439 4.24675200017278 21.7539255178735 16.5770435799242 19.2842350982821 down 1.0 6.0 4.0 23.0 20.0 18.0

P2RX6 1.75662547334861 0.000426111516518347 0.00127554852267552 27.6987179847708 24.7061111136176 23.3571360009503 7.56658278882556 8.28852178996211 6.42807836609403 up 26.0 24.0 22.0 8.0 10.0 6.0

ARSI 4.29487629281405 0.000440878273028715 0.00131603135596589 19.1760355279182 10.2942129640073 7.43181600030236 0 0.82885217899621 1.07134639434901 up 18.0 10.0 7.0 0.0 1.0 1.0

C10orf99 2.57236981623803 0.000441880296269642 0.00131879003150242 15.9800296065985 16.4707407424117 16.9870080006911 4.72911424301597 3.31540871598484 0 up 15.0 16.0 16.0 5.0 4.0 0.0

ANKRD18DP 5.25773647984794 0.000445610486452494 0.0013289862043143 5.32667653553284 9.26479166760659 5.30844000021597 0 0 0 up 5.0 9.0 5.0 0.0 0.0 0.0

EID3 -1.54765585025596 0.000447803128125018 0.00133458567741693 8.52268245685255 14.4118981496103 9.55519200038875 29.320508306699 30.6675306228598 35.3544310135172 down 8.0 14.0 9.0 31.0 37.0 33.0

HSD17B3 1.530316840243 0.000456744084561577 0.00136051429443874 24.5027120634511 37.0591666704264 32.912328001339 11.3498741832383 11.6039305059469 9.64211754914105 up 23.0 36.0 31.0 12.0 14.0 9.0

CA11 1.00045601408167 0.000457757193305058 0.00136322051327256 60.7241125050744 68.9712268588491 78.5649120031964 44.4536738843501 24.8655653698863 35.3544310135172 up 57.0 67.0 74.0 47.0 30.0 33.0

ACSM3 1.85383939454295 0.000466826300179007 0.00138810558627916 21.3067061421314 24.7061111136176 24.4188240009935 6.62075994022236 8.28852178996211 4.28538557739602 up 20.0 24.0 23.0 7.0 10.0 4.0

L3MBTL4 4.30694948271628 0.000491656859801747 0.00145861071892147 4.26134122842627 20.5884259280146 12.7402560005183 0.945822848603194 0 1.07134639434901 up 4.0 20.0 12.0 1.0 0.0 1.0

B3GALT5-AS1 1.05352617711917 0.000492426390412314 0.00146012656285395 52.2014300482219 68.9712268588491 59.4545280024189 34.9954453983182 21.5501566539015 31.0690454361212 up 49.0 67.0 56.0 37.0 26.0 29.0

AMBP 1.58106494693746 0.000497046944316376 0.00147176634097525 35.1560651345168 37.0591666704264 20.1720720008207 10.4040513346351 10.7750783269507 9.64211754914105 up 33.0 36.0 19.0 11.0 13.0 9.0

WIPF3 1.46492706124164 0.000498623907651206 0.00147617773429071 42.6134122842627 43.2356944488308 22.2954480009071 12.2956970318415 14.9193392219318 11.7848103378391 up 40.0 42.0 21.0 13.0 18.0 11.0

TSIX -5.17825141651502 0.000498748073778736 0.00147628732654408 0 0 0 3.78329139441278 6.63081743196968 10.7134639434901 down 0.0 0.0 0.0 4.0 8.0 10.0

NOTUM 1.55716967607486 0.000504417808014734 0.00149150596400237 25.5680473705576 55.5887500056395 25.4805120010367 12.2956970318415 14.0904870429356 9.64211754914105 up 24.0 54.0 24.0 13.0 17.0 9.0

ZFHX4-AS1 -2.90951875748834 0.000509716983296119 0.00150638619976208 3.19600592131971 0 3.18506400012958 19.8622798206671 19.8924522959091 7.49942476044304 down 3.0 0.0 3.0 21.0 24.0 7.0

NCAM2 -1.54571433491307 0.000522759295405423 0.00154304625617143 18.1107002208117 8.23537037120586 7.43181600030236 33.1037997011118 33.1540871598484 32.1403918304702 down 17.0 8.0 7.0 35.0 40.0 30.0

KANK4 -3.81700393262365 0.000524915619893467 0.0015486016372336 2.13067061421314 1.02942129640073 0 17.0248112748575 24.8655653698863 2.14269278869801 down 2.0 1.0 0.0 18.0 30.0 2.0

LINC00408 2.8692820565766 0.000533619118919411 0.001572635329592 12.7840236852788 13.3824768532095 14.8636320006047 2.83746854580958 1.65770435799242 1.07134639434901 up 12.0 13.0 14.0 3.0 2.0 1.0

RTEL1 -1.39113516311494 0.000536810764292109 0.00158176628058938 14.914694299492 11.3236342604081 19.1103840007775 52.9660795217789 35.6406436968371 29.9976990417721 down 14.0 11.0 18.0 56.0 43.0 28.0

FA2H -1.93401710822941 0.00053966105808582 0.00158905932973645 7.45734714974598 10.2942129640073 2.12337600008639 17.9706341234607 23.2078610118939 35.3544310135172 down 7.0 10.0 2.0 19.0 28.0 33.0

KGFLP2 1.6679501228287 0.000552497327154507 0.00162403344126277 39.417406362943 40.1474305596286 16.9870080006911 14.1873427290479 5.80196525297347 10.7134639434901 up 37.0 39.0 16.0 15.0 7.0 10.0

NALCN-AS1 -2.22329629000692 0.000555144298679753 0.00163153095413515 7.45734714974598 4.11768518560293 1.06168800004319 17.0248112748575 21.5501566539015 20.3555814926311 down 7.0 4.0 1.0 18.0 26.0 19.0

C1orf220 1.21876001143238 0.000561180089955469 0.00164841177802075 42.6134122842627 44.2651157452315 48.8376480019869 17.9706341234607 25.6944175488825 13.9275031265371 up 40.0 43.0 46.0 19.0 31.0 13.0

LOC101929380 -5.13124998703615 0.000562107005272155 0.00165035004966587 0 0 0 4.72911424301597 9.11737396895832 6.42807836609403 down 0.0 0.0 0.0 5.0 11.0 6.0

ADGRF2 -2.19043610248123 0.000563397307297458 0.00165325640505989 3.19600592131971 4.11768518560293 5.30844000021597 24.5913940636831 14.9193392219318 18.2128887039331 down 3.0 4.0 5.0 26.0 18.0 17.0

C11orf87 -1.89455910925396 0.000565226619924089 0.00165771103558069 3.19600592131971 10.2942129640073 6.37012800025917 17.0248112748575 28.1809740858712 28.9263526474231 down 3.0 10.0 6.0 18.0 34.0 27.0

MBL1P 4.80646059620239 0.000566118431637803 0.00165975185738047 7.45734714974598 10.2942129640073 10.6168800004319 0 0.82885217899621 0 up 7.0 10.0 10.0 0.0 1.0 0.0

ABI3BP 1.27429090407562 0.000567450031723198 0.00166336797670721 42.6134122842627 38.0885879668271 52.0227120021165 24.5913940636831 19.0636001169128 10.7134639434901 up 40.0 37.0 49.0 26.0 23.0 10.0

GNG7 1.3153110461653 0.000574306811914803 0.00168146218736173 29.8293885989839 62.7946990804447 52.0227120021165 14.1873427290479 15.748191400928 28.9263526474231 up 28.0 61.0 49.0 15.0 19.0 27.0

LOC388849 1.67525990598453 0.000574317604254721 0.00168146218736173 23.4373767563445 26.764953706419 30.7889520012526 12.2956970318415 6.63081743196968 6.42807836609403 up 22.0 26.0 29.0 13.0 8.0 6.0

CHGA -4.82633092449695 0.000578290810638275 0.00169156462453021 0 0 1.06168800004319 19.8622798206671 5.80196525297347 6.42807836609403 down 0.0 0.0 1.0 21.0 7.0 6.0

SLC25A41 1.25228245854162 0.000578367314361214 0.00169156462453021 52.2014300482219 57.647592598441 32.912328001339 22.6997483664767 13.2616348639394 24.6409670700271 up 49.0 56.0 31.0 24.0 16.0 23.0

SOWAHA -1.3098589276452 0.000581498631541153 0.00170042927987349 17.0453649137051 24.7061111136176 10.6168800004319 37.8329139441278 52.2176872767613 39.6398165909132 down 16.0 24.0 10.0 40.0 63.0 37.0

RGS22 -5.12425893930671 0.000582014928665179 0.00170135166377707 0 0 0 7.56658278882556 8.28852178996211 4.28538557739602 down 0.0 0.0 0.0 8.0 10.0 4.0

WT1-AS 2.85797540630882 0.000582566218365136 0.0017026693821783 9.58801776395912 16.4707407424117 14.8636320006047 1.89164569720639 1.65770435799242 2.14269278869801 up 9.0 16.0 14.0 2.0 2.0 2.0

ZFPM2 1.28322735686727 0.000587256638850666 0.0017154901641887 36.2214004416233 47.3533796344337 64.7629680026348 10.4040513346351 24.8655653698863 25.7123134643761 up 34.0 46.0 61.0 11.0 30.0 24.0

C18orf61 5.18650438832746 0.000588481728105118 0.0017187724955346 5.32667653553284 5.14710648200366 8.49350400034555 0 0 0 up 5.0 5.0 8.0 0.0 0.0 0.0

CCDC102B 1.06068411805911 0.00060027911018595 0.00174991019214421 53.2667653553284 52.5004861164373 73.2564720029804 36.8870910955246 22.3790088328977 26.7836598587251 up 50.0 51.0 69.0 39.0 27.0 25.0

MAFB -1.60048228341075 0.000616019019154662 0.00179178608936813 8.52268245685255 15.441319446011 6.37012800025917 23.6455712150799 32.3252349808522 36.4257774078662 down 8.0 15.0 6.0 25.0 39.0 34.0

CEND1 1.6835468627942 0.000629693782336688 0.00182873524306249 35.1560651345168 36.0297453740256 18.0486960007343 7.56658278882556 5.80196525297347 14.9988495208861 up 33.0 35.0 17.0 8.0 7.0 14.0

NUP62CL 1.10934014137406 0.000647261468684016 0.00187685882908374 47.9400888197956 63.8241203768454 42.4675200017278 25.5372169122862 21.5501566539015 24.6409670700271 up 45.0 62.0 40.0 27.0 26.0 23.0

GPR45 1.83196462206035 0.000656300898124945 0.00190079282171937 27.6987179847708 21.6178472244154 24.4188240009935 12.2956970318415 4.14426089498105 4.28538557739602 up 26.0 21.0 23.0 13.0 5.0 4.0

ATP6V0A4 1.14471360731206 0.000673977266541843 0.00194765863105063 42.6134122842627 54.5593287092388 46.7142720019006 23.6455712150799 24.8655653698863 16.0701959152351 up 40.0 53.0 44.0 25.0 30.0 15.0

C10orf25 1.67890651674398 0.000697508069513701 0.00201291085137344 41.5480769771562 23.6766898172168 20.1720720008207 7.56658278882556 6.63081743196968 12.8561567321881 up 39.0 23.0 19.0 8.0 8.0 12.0

FRG1DP -2.61688089879347 0.000704888913443693 0.00203247969255083 0 2.05884259280146 6.37012800025917 22.6997483664767 19.0636001169128 9.64211754914105 down 0.0 2.0 6.0 24.0 23.0 9.0

SLC22A20 1.22911390567269 0.000705458941511497 0.00203377713907501 51.1360947411153 48.3828009308344 46.7142720019006 32.1579768525086 19.0636001169128 10.7134639434901 up 48.0 47.0 44.0 34.0 23.0 10.0

SYTL5 2.15516367414843 0.000706805848784955 0.00203696683879162 17.0453649137051 23.6766898172168 13.8019440005615 4.72911424301597 3.31540871598484 4.28538557739602 up 16.0 23.0 13.0 5.0 4.0 4.0

ABCC6P1 5.25836916515328 0.000713608601917087 0.00205307909586879 9.58801776395912 9.26479166760659 1.06168800004319 0 0 0 up 9.0 9.0 1.0 0.0 0.0 0.0

LOC101928674 -1.85124936119516 0.000720592743657787 0.00207129468179769 9.58801776395912 3.0882638892022 7.43181600030236 31.2121540039054 20.7213044749053 20.3555814926311 down 9.0 3.0 7.0 33.0 25.0 19.0

PSMB8-AS1 -1.2375999993281 0.000721189618635804 0.0020724261475302 15.9800296065985 17.5001620388124 22.2954480009071 47.2911424301597 51.3888350977651 32.1403918304702 down 15.0 17.0 21.0 50.0 62.0 30.0

GOLGA8T 3.08855126458074 0.000726563011749418 0.00208645150348112 12.7840236852788 16.4707407424117 9.55519200038875 0.945822848603194 3.31540871598484 0 up 12.0 16.0 9.0 1.0 4.0 0.0

LOC100996255 1.56346402097945 0.000727193293965301 0.00208790752538512 19.1760355279182 37.0591666704264 32.912328001339 9.45822848603194 9.11737396895832 11.7848103378391 up 18.0 36.0 31.0 10.0 11.0 11.0

SSPO 4.80985052702508 0.000737000588962382 0.00211284311677488 4.26134122842627 7.20594907480513 16.9870080006911 0 0.82885217899621 0 up 4.0 7.0 16.0 0.0 1.0 0.0

SH2D5 1.27638174252496 0.000739649579577168 0.00211971982372479 49.0054241269022 30.882638892022 43.529208001771 21.7539255178735 13.2616348639394 16.0701959152351 up 46.0 30.0 41.0 23.0 16.0 15.0

LMCD1-AS1 2.77750679548387 0.000739838891220262 0.00211990372416631 7.45734714974598 19.5590046316139 16.9870080006911 0.945822848603194 4.14426089498105 1.07134639434901 up 7.0 19.0 16.0 1.0 5.0 1.0

PCDHB17P 2.29317586411164 0.000746973565036856 0.00213926157087478 12.7840236852788 22.6472685208161 14.8636320006047 2.83746854580958 4.14426089498105 3.21403918304702 up 12.0 22.0 14.0 3.0 5.0 3.0

VASH2 -2.32109192752791 0.000751219922394856 0.0021506955358068 3.19600592131971 3.0882638892022 4.24675200017278 12.2956970318415 14.9193392219318 25.7123134643761 down 3.0 3.0 4.0 13.0 18.0 24.0

FAM81A 1.1792338003211 0.000776446419300296 0.00221879256797352 66.0507890406073 41.1768518560293 40.3441440016414 17.0248112748575 27.3521219068749 20.3555814926311 up 62.0 40.0 38.0 18.0 33.0 19.0

LINC01127 -1.89794807711173 0.00078341530468493 0.00223832944195694 4.26134122842627 3.0882638892022 12.7402560005183 25.5372169122862 17.4058957589204 32.1403918304702 down 4.0 3.0 12.0 27.0 21.0 30.0

ATP6V1B1-AS1 2.13348697765377 0.000802757079699186 0.00228934426432731 15.9800296065985 17.5001620388124 30.7889520012526 7.56658278882556 6.63081743196968 0 up 15.0 17.0 29.0 8.0 8.0 0.0

KC6 4.10137270878142 0.000808880921153255 0.00230564408620834 8.52268245685255 10.2942129640073 13.8019440005615 0.945822848603194 0 1.07134639434901 up 8.0 10.0 13.0 1.0 0.0 1.0

SEMA5B 5.09929944545625 0.000836980793506776 0.00238013339634124 4.26134122842627 7.20594907480513 6.37012800025917 0 0 0 up 4.0 7.0 6.0 0.0 0.0 0.0

GSTO2 1.3428588147703 0.000856703103965162 0.00243091299517083 41.5480769771562 44.2651157452315 27.6038880011231 19.8622798206671 14.9193392219318 9.64211754914105 up 39.0 43.0 26.0 21.0 18.0 9.0

TNNT2 -1.70315201489485 0.000873443644720092 0.00247551201966845 8.52268245685255 7.20594907480513 7.43181600030236 31.2121540039054 25.6944175488825 18.2128887039331 down 8.0 7.0 7.0 33.0 31.0 17.0

LOC101929710 -1.53904912763265 0.000878936773270267 0.00248902776189081 11.7186883781723 7.20594907480513 10.6168800004319 27.4288626094926 32.3252349808522 25.7123134643761 down 11.0 7.0 10.0 29.0 39.0 24.0

PTH1R 1.61105421359414 0.000885432981650673 0.00250571912949589 17.0453649137051 35.0003240776249 38.220768001555 7.56658278882556 7.45966961096589 14.9988495208861 up 16.0 34.0 36.0 8.0 9.0 14.0

FCMR 2.26358472792835 0.000905741340117055 0.00255806240439862 15.9800296065985 20.5884259280146 12.7402560005183 4.72911424301597 3.31540871598484 2.14269278869801 up 15.0 20.0 12.0 5.0 4.0 2.0

LOC101929626 4.74689086970613 0.000909413347571693 0.00256800501881388 3.19600592131971 13.3824768532095 10.6168800004319 0 0.82885217899621 0 up 3.0 13.0 10.0 0.0 1.0 0.0

NKAIN4 -1.24187697733607 0.000913389132154524 0.00257837225440721 14.914694299492 12.3530555568088 26.5422000010799 43.5078510357469 39.7849045918181 43.9252021683092 down 14.0 12.0 25.0 46.0 48.0 41.0

SIGLEC15 -1.44998446455055 0.000921456354990609 0.00259811428987033 13.8493589923854 15.441319446011 6.37012800025917 36.8870910955246 30.6675306228598 29.9976990417721 down 13.0 15.0 6.0 39.0 37.0 28.0

ISM1 -2.20761573406104 0.000927696763528973 0.00261309996245108 4.26134122842627 5.14710648200366 2.12337600008639 23.6455712150799 15.748191400928 13.9275031265371 down 4.0 5.0 2.0 25.0 19.0 13.0

POPDC2 -2.90410855144766 0.00092861190509382 0.00261524283828584 1.06533530710657 3.0882638892022 1.06168800004319 12.2956970318415 14.0904870429356 12.8561567321881 down 1.0 3.0 1.0 13.0 17.0 12.0

LINC00620 -2.44052494010559 0.000932241370175939 0.0026245917917202 3.19600592131971 3.0882638892022 3.18506400012958 9.45822848603194 12.4327826849432 29.9976990417721 down 3.0 3.0 3.0 10.0 15.0 28.0

PDZRN3 -5.0937997101284 0.000936075419947132 0.00263494809137661 0 0 0 6.62075994022236 1.65770435799242 11.7848103378391 down 0.0 0.0 0.0 7.0 2.0 11.0

DLEU7 4.69023859182347 0.000963522815851453 0.00270771010294021 6.39201184263941 13.3824768532095 6.37012800025917 0.945822848603194 0 0 up 6.0 13.0 6.0 1.0 0.0 0.0

AGAP11 -1.17541731514299 0.000966157661590962 0.00271421406092055 15.9800296065985 23.6766898172168 18.0486960007343 39.7245596413342 49.7311307397726 40.7111629852622 down 15.0 23.0 17.0 42.0 60.0 38.0

ITGAX 1.98636783315601 0.000969648987907659 0.00272221641616767 14.914694299492 26.764953706419 18.0486960007343 7.56658278882556 3.31540871598484 4.28538557739602 up 14.0 26.0 17.0 8.0 4.0 4.0

LPPR5 4.08792331639525 0.000977037872696283 0.00274114302144337 12.7840236852788 7.20594907480513 11.6785680004751 0.945822848603194 0.82885217899621 0 up 12.0 7.0 11.0 1.0 1.0 0.0

EBF1 2.8161569595951 0.000981710306121271 0.00275242843192558 11.7186883781723 10.2942129640073 16.9870080006911 0.945822848603194 3.31540871598484 1.07134639434901 up 11.0 10.0 16.0 1.0 4.0 1.0

PRLR 5.18594893070044 0.000983397095400647 0.00275670143903475 11.7186883781723 6.17652777840439 1.06168800004319 0 0 0 up 11.0 6.0 1.0 0.0 0.0 0.0

SP2-AS1 1.22608098866294 0.000983798866228311 0.00275737140865446 42.6134122842627 35.0003240776249 40.3441440016414 19.8622798206671 17.4058957589204 12.8561567321881 up 40.0 34.0 38.0 21.0 21.0 12.0

SELL -4.62081367345439 0.00100747754738621 0.00282047093913774 0 1.02942129640073 0 13.2415198804447 9.11737396895832 5.35673197174503 down 0.0 1.0 0.0 14.0 11.0 5.0

HIC1 -1.1253967152663 0.00100835320698537 0.00282245593627431 27.6987179847708 18.5295833352132 26.5422000010799 71.8825364938428 39.7849045918181 47.1392413513562 down 26.0 18.0 25.0 76.0 48.0 44.0

HEYL -2.50068109511189 0.00101785719090688 0.00284717648843502 1.06533530710657 6.17652777840439 1.06168800004319 17.9706341234607 12.4327826849432 17.1415423095841 down 1.0 6.0 1.0 19.0 15.0 16.0

RPS6KA2-IT1 -5.07326950836474 0.00103088485616807 0.00288076368582764 0 0 0 4.72911424301597 12.4327826849432 2.14269278869801 down 0.0 0.0 0.0 5.0 15.0 2.0

OR2L13 1.84457107675645 0.00103741750071496 0.00289662971190234 23.4373767563445 17.5001620388124 22.2954480009071 4.72911424301597 8.28852178996211 4.28538557739602 up 22.0 17.0 21.0 5.0 10.0 4.0

KIF5A 2.32842431114403 0.00107905261629372 0.0030077995476048 19.1760355279182 15.441319446011 12.7402560005183 5.67493709161917 1.65770435799242 2.14269278869801 up 18.0 15.0 12.0 6.0 2.0 2.0

LEF1 2.48240468585249 0.00109656262259229 0.00305271500849851 17.0453649137051 13.3824768532095 11.6785680004751 1.89164569720639 2.48655653698863 3.21403918304702 up 16.0 13.0 11.0 2.0 3.0 3.0

DNM1P46 2.83095690940643 0.00109969562568619 0.00305992836713603 10.6533530710657 13.3824768532095 15.9253200006479 4.72911424301597 0.82885217899621 0 up 10.0 13.0 15.0 5.0 1.0 0.0

MIR99AHG -2.69580994263659 0.00110272855383429 0.00306735988537813 3.19600592131971 2.05884259280146 1.06168800004319 11.3498741832383 16.5770435799242 12.8561567321881 down 3.0 2.0 1.0 12.0 20.0 12.0

RHD 1.73496282139226 0.00111117862409088 0.00308579768722944 26.6333826776642 16.4707407424117 25.4805120010367 8.51240563742875 6.63081743196968 5.35673197174503 up 25.0 16.0 24.0 9.0 8.0 5.0

UNC13C -4.60114211837978 0.00111220764865756 0.00308814908511048 0 0 1.06168800004319 5.67493709161917 14.0904870429356 7.49942476044304 down 0.0 0.0 1.0 6.0 17.0 7.0

FRMD4B 1.82659463903519 0.00111556041007468 0.0030959360004366 23.4373767563445 31.9120601884227 13.8019440005615 3.78329139441278 9.11737396895832 6.42807836609403 up 22.0 31.0 13.0 4.0 11.0 6.0

SLFN12L 2.4773598942712 0.00112267920886401 0.00311365189884682 18.1107002208117 18.5295833352132 9.55519200038875 4.72911424301597 3.31540871598484 0 up 17.0 18.0 9.0 5.0 4.0 0.0

MFAP2 -1.95676996594395 0.00113354531579341 0.00314121669385577 10.6533530710657 5.14710648200366 1.06168800004319 20.8081026692703 19.0636001169128 25.7123134643761 down 10.0 5.0 1.0 22.0 23.0 24.0

LINC00689 5.01339541742196 0.00113728247941065 0.00314899725420341 5.32667653553284 6.17652777840439 5.30844000021597 0 0 0 up 5.0 6.0 5.0 0.0 0.0 0.0

LOC101059948 1.19676827269823 0.00114475258203775 0.00316709272247583 59.6587771979678 33.9709027812242 53.0844000021597 32.1579768525086 15.748191400928 16.0701959152351 up 56.0 33.0 50.0 34.0 19.0 15.0

ZNF843 1.75754103901897 0.00114820122131382 0.00317559652066222 33.0253945203036 24.7061111136176 14.8636320006047 10.4040513346351 6.63081743196968 4.28538557739602 up 31.0 24.0 14.0 11.0 8.0 4.0

GDF7 -1.88344887168259 0.00115707204315209 0.00319856403573702 3.19600592131971 4.11768518560293 11.6785680004751 17.9706341234607 32.3252349808522 19.2842350982821 down 3.0 4.0 11.0 19.0 39.0 18.0

CA3-AS1 -3.98211227284874 0.0011849660578106 0.00326820661336886 2.13067061421314 0 0 11.3498741832383 13.2616348639394 8.57077115479204 down 2.0 0.0 0.0 12.0 16.0 8.0

BAALC 1.80681896804104 0.00118783191710245 0.00327504438081306 37.2867357487299 19.5590046316139 16.9870080006911 4.72911424301597 11.6039305059469 4.28538557739602 up 35.0 19.0 16.0 5.0 14.0 4.0

MGARP 1.18431972802675 0.00119101253837311 0.00328327947925802 37.2867357487299 45.2945370416322 58.3928400023757 11.3498741832383 24.0367131908901 26.7836598587251 up 35.0 44.0 55.0 12.0 29.0 25.0

LOC100130238 1.04557318945766 0.00121273235962718 0.00333609713739599 46.874753512689 78.2360185264557 58.3928400023757 28.3746854580958 19.8924522959091 41.7825093796112 up 44.0 76.0 55.0 30.0 24.0 39.0

DMBT1 -2.0814703308872 0.00122716021193923 0.00337414283237308 5.32667653553284 5.14710648200366 2.12337600008639 19.8622798206671 17.4058957589204 16.0701959152351 down 5.0 5.0 2.0 21.0 21.0 15.0

SRL -3.95727685450471 0.00125763524038391 0.00345513152320846 2.13067061421314 0 0 10.4040513346351 11.6039305059469 10.7134639434901 down 2.0 0.0 0.0 11.0 14.0 10.0

ALG1L 3.46936397506916 0.0012864958467788 0.00352470316099513 11.7186883781723 11.3236342604081 8.49350400034555 0 0.82885217899621 2.14269278869801 up 11.0 11.0 8.0 0.0 1.0 2.0

C4orf47 -1.12446939895368 0.00129154507449475 0.00353739265350704 25.5680473705576 17.5001620388124 21.2337600008639 59.5868394620012 39.7849045918181 40.7111629852622 down 24.0 17.0 20.0 63.0 48.0 38.0

FAM95B1 4.57589946213974 0.00134055713070297 0.00366511262009495 6.39201184263941 8.23537037120586 9.55519200038875 0 0.82885217899621 0 up 6.0 8.0 9.0 0.0 1.0 0.0

VGLL2 4.57444192251469 0.00134533237618583 0.00367757470592027 6.39201184263941 9.26479166760659 8.49350400034555 0 0.82885217899621 0 up 6.0 9.0 8.0 0.0 1.0 0.0

ALOX5AP 1.9620239341525 0.00135108857223671 0.00369152264736934 21.3067061421314 26.764953706419 15.9253200006479 1.89164569720639 3.31540871598484 11.7848103378391 up 20.0 26.0 15.0 2.0 4.0 11.0

FCGBP 2.46962227000399 0.00136637962538243 0.00372789029698477 8.52268245685255 17.5001620388124 15.9253200006479 2.83746854580958 1.65770435799242 3.21403918304702 up 8.0 17.0 15.0 3.0 2.0 3.0

IDO2 -3.97019019163211 0.00137302165880341 0.00374420265617029 1.06533530710657 0 1.06168800004319 12.2956970318415 4.97311307397726 16.0701959152351 down 1.0 0.0 1.0 13.0 6.0 15.0

MCF2L-AS1 2.57986762192874 0.00139318455869473 0.00379674170274914 10.6533530710657 16.4707407424117 12.7402560005183 0.945822848603194 1.65770435799242 4.28538557739602 up 10.0 16.0 12.0 1.0 2.0 4.0

RAMP1 3.98305171766962 0.0014194682439404 0.00386277783973501 8.52268245685255 11.3236342604081 9.55519200038875 0.945822848603194 0.82885217899621 0 up 8.0 11.0 9.0 1.0 1.0 0.0

HS3ST4 5.11184154600197 0.00142244517931467 0.00387025720166889 4.26134122842627 1.02942129640073 12.7402560005183 0 0 0 up 4.0 1.0 12.0 0.0 0.0 0.0

LAG3 1.07145158998935 0.00143719388396805 0.00390724833805468 47.9400888197956 41.1768518560293 45.6525840018574 21.7539255178735 19.0636001169128 23.5696206756781 up 45.0 40.0 43.0 23.0 23.0 22.0

LINC00052 -2.68324746849627 0.00144777704128871 0.00393475743292649 3.19600592131971 1.02942129640073 2.12337600008639 17.9706341234607 14.9193392219318 7.49942476044304 down 3.0 1.0 2.0 19.0 18.0 7.0

TMEM145 -1.71786708230077 0.00146192966872589 0.00397003664447204 7.45734714974598 6.17652777840439 6.37012800025917 25.5372169122862 19.8924522959091 20.3555814926311 down 7.0 6.0 6.0 27.0 24.0 19.0

ZNF280A 1.4908219626866 0.00148304689014407 0.00402178767689893 24.5027120634511 30.882638892022 24.4188240009935 9.45822848603194 7.45966961096589 11.7848103378391 up 23.0 30.0 23.0 10.0 9.0 11.0

IMPG1 2.29577624233419 0.00153249035180904 0.00414570210150832 25.5680473705576 10.2942129640073 16.9870080006911 1.89164569720639 0.82885217899621 8.57077115479204 up 24.0 10.0 16.0 2.0 1.0 8.0

IGFALS 1.66778512239398 0.00154669232797852 0.00418078315556983 22.3720414492379 29.8532175956212 16.9870080006911 6.62075994022236 5.80196525297347 9.64211754914105 up 21.0 29.0 16.0 7.0 7.0 9.0

EEF1DP3 3.53286813878843 0.00154724044842701 0.00418093048274902 8.52268245685255 6.17652777840439 18.0486960007343 2.83746854580958 0 0 up 8.0 6.0 17.0 3.0 0.0 0.0

DRAXIN -2.40071986127943 0.00156786340712253 0.00423260655245507 1.06533530710657 9.26479166760659 0 26.4830397608894 11.6039305059469 17.1415423095841 down 1.0 9.0 0.0 28.0 14.0 16.0

LVCAT1 -2.36871689043274 0.00156936555212955 0.0042359866878704 3.19600592131971 2.05884259280146 5.30844000021597 26.4830397608894 23.2078610118939 4.28538557739602 down 3.0 2.0 5.0 28.0 28.0 4.0

IL4I1 -1.21540216071704 0.00159703107695734 0.00430585810021603 13.8493589923854 20.5884259280146 13.8019440005615 40.6703824899374 33.1540871598484 38.5684701965642 down 13.0 20.0 13.0 43.0 40.0 36.0

KRT9 -4.88359458780898 0.00160745325837044 0.00433257886981628 0 0 0 2.83746854580958 5.80196525297347 8.57077115479204 down 0.0 0.0 0.0 3.0 7.0 8.0

LINC00518 -1.66639183385795 0.00161475232594486 0.00434948392455175 7.45734714974598 11.3236342604081 4.24675200017278 29.320508306699 26.5232697278787 17.1415423095841 down 7.0 11.0 4.0 31.0 32.0 16.0

SAA2 -3.03735991265949 0.00163324093647327 0.00439509157487803 2.13067061421314 0 2.12337600008639 10.4040513346351 12.4327826849432 11.7848103378391 down 2.0 0.0 2.0 11.0 15.0 11.0

EFNB3 1.10892212516014 0.00164180251438668 0.00441672774237102 40.4827416700496 53.5299074128381 42.4675200017278 14.1873427290479 27.3521219068749 21.4269278869801 up 38.0 52.0 40.0 15.0 33.0 20.0

PKIB 1.76746238076347 0.00164688352152294 0.00442898981657384 21.3067061421314 20.5884259280146 19.1103840007775 8.51240563742875 4.14426089498105 5.35673197174503 up 20.0 20.0 18.0 9.0 5.0 5.0

VIM-AS1 1.07559162253109 0.00165341201899658 0.00444584120663524 50.0707594340087 59.7064351912425 48.8376480019869 26.4830397608894 34.8117915178408 12.8561567321881 up 47.0 58.0 46.0 28.0 42.0 12.0

TRPM8 2.06864032250783 0.00165974616244939 0.00445862670344 19.1760355279182 11.3236342604081 23.3571360009503 2.83746854580958 7.45966961096589 2.14269278869801 up 18.0 11.0 22.0 3.0 9.0 2.0

DCDC5 -3.07009979435684 0.00166011006098077 0.00445889716711816 2.13067061421314 2.05884259280146 0 7.56658278882556 10.7750783269507 17.1415423095841 down 2.0 2.0 0.0 8.0 13.0 16.0

NNT-AS1 1.5693266664367 0.00167086927067483 0.00448566171873718 31.9600592131971 26.764953706419 25.4805120010367 12.2956970318415 13.2616348639394 2.14269278869801 up 30.0 26.0 24.0 13.0 16.0 2.0

ST3GAL4-AS1 1.12854960700975 0.00168593983073959 0.00452396970263403 49.0054241269022 33.9709027812242 40.3441440016414 17.0248112748575 19.0636001169128 20.3555814926311 up 46.0 33.0 38.0 18.0 23.0 19.0

BVES-AS1 2.92667708847356 0.00169769279734378 0.0045526224453069 9.58801776395912 13.3824768532095 11.6785680004751 0.945822848603194 3.31540871598484 0 up 9.0 13.0 11.0 1.0 4.0 0.0

CASC15 -1.38487020407747 0.00169910563225394 0.00455448784105518 13.8493589923854 20.5884259280146 6.37012800025917 47.2911424301597 26.5232697278787 33.2117382248192 down 13.0 20.0 6.0 50.0 32.0 31.0

GAL3ST2 3.93069630900354 0.00169919499146805 0.00455448784105518 7.45734714974598 13.3824768532095 8.49350400034555 0 0 2.14269278869801 up 7.0 13.0 8.0 0.0 0.0 2.0

LOC389834 1.32045968873554 0.00170440104500234 0.00456627450614259 54.332100662435 27.7943750028198 38.220768001555 12.2956970318415 9.94622614795453 26.7836598587251 up 51.0 27.0 36.0 13.0 12.0 25.0

KLHL7-AS1 1.43334220023512 0.00173950184808857 0.00465442446795457 29.8293885989839 32.9414814848234 39.2824560015982 14.1873427290479 4.14426089498105 20.3555814926311 up 28.0 32.0 37.0 15.0 5.0 19.0

AARD 2.83403479985265 0.00174606414757684 0.00467124552431329 18.1107002208117 3.0882638892022 20.1720720008207 0.945822848603194 0.82885217899621 4.28538557739602 up 17.0 3.0 19.0 1.0 1.0 4.0

LINC00470 4.91898535246606 0.00174950258973827 0.00467970533241217 3.19600592131971 6.17652777840439 6.37012800025917 0 0 0 up 3.0 6.0 6.0 0.0 0.0 0.0

EFHC2 1.49533717240574 0.00176879012213437 0.00472681884665661 38.3520710558365 17.5001620388124 33.9740160013822 5.67493709161917 13.2616348639394 12.8561567321881 up 36.0 17.0 32.0 6.0 16.0 12.0

PALM3 -3.32479783785998 0.00179881439833868 0.0048040226876647 1.06533530710657 1.02942129640073 1.06168800004319 9.45822848603194 12.4327826849432 9.64211754914105 down 1.0 1.0 1.0 10.0 15.0 9.0

CATSPER2P1 1.04157221074321 0.00180200002393217 0.00481101346026965 53.2667653553284 41.1768518560293 45.6525840018574 25.5372169122862 18.2347479379166 24.6409670700271 up 50.0 40.0 43.0 27.0 22.0 23.0

ANKRD20A4 2.33938714333404 0.00180869491889109 0.00482434854034358 11.7186883781723 16.4707407424117 13.8019440005615 2.83746854580958 4.14426089498105 1.07134639434901 up 11.0 16.0 13.0 3.0 5.0 1.0

PTGER1 2.61957678110904 0.00185844202538191 0.0049500012437059 21.3067061421314 13.3824768532095 5.30844000021597 2.83746854580958 2.48655653698863 1.07134639434901 up 20.0 13.0 5.0 3.0 3.0 1.0

AQP4 2.88143739528395 0.00186897498600671 0.0049764910818852 15.9800296065985 13.3824768532095 5.30844000021597 0.945822848603194 1.65770435799242 2.14269278869801 up 15.0 13.0 5.0 1.0 2.0 2.0

FAM26F 2.1394545947097 0.00189506728314825 0.00504358833881088 19.1760355279182 10.2942129640073 20.1720720008207 0.945822848603194 4.97311307397726 5.35673197174503 up 18.0 10.0 19.0 1.0 6.0 5.0

DLX6 3.95046476607732 0.00190223698933998 0.00505949043796818 7.45734714974598 10.2942129640073 10.6168800004319 0 1.65770435799242 0 up 7.0 10.0 10.0 0.0 2.0 0.0

TMEM92-AS1 1.4717224001948 0.00190980020376356 0.00507721523097219 31.9600592131971 28.8237962992205 20.1720720008207 13.2415198804447 7.45966961096589 8.57077115479204 up 30.0 28.0 19.0 14.0 9.0 8.0

PCDHA5 1.6635944724634 0.00192539032536043 0.00511625288025186 18.1107002208117 43.2356944488308 18.0486960007343 12.2956970318415 8.28852178996211 4.28538557739602 up 17.0 42.0 17.0 13.0 10.0 4.0

LOC101929555 3.41568326970198 0.00192965944345786 0.00512667202258081 10.6533530710657 7.20594907480513 11.6785680004751 0.945822848603194 1.65770435799242 0 up 10.0 7.0 11.0 1.0 2.0 0.0

CLDN9 1.62864478772757 0.00193330772084778 0.0051340700409408 22.3720414492379 24.7061111136176 35.0357040014254 1.89164569720639 9.94622614795453 14.9988495208861 up 21.0 24.0 33.0 2.0 12.0 14.0

NAPSA -4.8591223522645 0.00195347527755603 0.00518499132145279 0 0 0 1.89164569720639 7.45966961096589 7.49942476044304 down 0.0 0.0 0.0 2.0 9.0 7.0

PDPN -4.8256649493245 0.00195370717856158 0.00518499132145279 0 0 0 3.78329139441278 8.28852178996211 4.28538557739602 down 0.0 0.0 0.0 4.0 10.0 4.0

UBD 4.92746269726385 0.00196593733622114 0.00521499819536267 9.58801776395912 3.0882638892022 3.18506400012958 0 0 0 up 9.0 3.0 3.0 0.0 0.0 0.0

C11orf96 1.69071813103394 0.00199232463239497 0.00528003430425074 23.4373767563445 30.882638892022 19.1103840007775 1.89164569720639 8.28852178996211 12.8561567321881 up 22.0 30.0 18.0 2.0 10.0 12.0

ST8SIA6 -2.50400999376215 0.00200171612028977 0.00530395201375759 4.26134122842627 0 3.18506400012958 9.45822848603194 17.4058957589204 14.9988495208861 down 4.0 0.0 3.0 10.0 21.0 14.0

LOC101926935 1.41693250694396 0.00200197574828607 0.00530395201375759 34.0907298274102 28.8237962992205 25.4805120010367 17.0248112748575 6.63081743196968 9.64211754914105 up 32.0 28.0 24.0 18.0 8.0 9.0

CCDC36 -1.19784395546941 0.00200289178102115 0.00530554914315846 20.2413708350248 16.4707407424117 15.9253200006479 31.2121540039054 55.5330959927461 33.2117382248192 down 19.0 16.0 15.0 33.0 67.0 31.0

LOC541472 -4.80131717418997 0.00200869779838667 0.00531926539075664 0 0 0 3.78329139441278 4.97311307397726 7.49942476044304 down 0.0 0.0 0.0 4.0 6.0 7.0

SLC1A3 4.12429846825506 0.00204100605582216 0.00539470160487244 15.9800296065985 1.02942129640073 14.8636320006047 0 1.65770435799242 0 up 15.0 1.0 14.0 0.0 2.0 0.0

FAM86HP 1.06449975921987 0.00204351077239173 0.00539963694966712 82.0308186472058 31.9120601884227 58.3928400023757 23.6455712150799 30.6675306228598 27.8550062530741 up 77.0 31.0 55.0 25.0 37.0 26.0

PTCH2 1.03303950026353 0.00205678353417899 0.0054313192625085 42.6134122842627 50.4416435236359 50.9610240020733 20.8081026692703 18.2347479379166 32.1403918304702 up 40.0 49.0 48.0 22.0 22.0 30.0

FYB -4.43332446474167 0.00209405656994082 0.00552285821186323 0 0 1.06168800004319 6.62075994022236 4.14426089498105 13.9275031265371 down 0.0 0.0 1.0 7.0 5.0 13.0

LINC00397 1.74156688621478 0.00211611770757837 0.00557756868933836 24.5027120634511 18.5295833352132 19.1103840007775 2.83746854580958 9.11737396895832 6.42807836609403 up 23.0 18.0 18.0 3.0 11.0 6.0

LCAL1 -3.34879911323747 0.00214799648928587 0.0056554337159625 0 3.0882638892022 0 13.2415198804447 9.11737396895832 9.64211754914105 down 0.0 3.0 0.0 14.0 11.0 9.0

ARHGAP28 1.79334720068106 0.00215016939299786 0.00565939551233432 34.0907298274102 19.5590046316139 15.9253200006479 2.83746854580958 12.4327826849432 4.28538557739602 up 32.0 19.0 15.0 3.0 15.0 4.0

GSG1 1.37090449528715 0.00221433137783345 0.00581833000473067 21.3067061421314 32.9414814848234 30.7889520012526 10.4040513346351 10.7750783269507 11.7848103378391 up 20.0 32.0 29.0 11.0 13.0 11.0

GNGT2 1.05358534218505 0.00222447966760722 0.0058422768324444 39.417406362943 41.1768518560293 47.7759600019437 17.9706341234607 22.3790088328977 21.4269278869801 up 37.0 40.0 45.0 19.0 27.0 20.0

PIWIL3 -4.79250041944719 0.0022303603119809 0.00585318414948977 0 0 0 8.51240563742875 3.31540871598484 4.28538557739602 down 0.0 0.0 0.0 9.0 4.0 4.0

JAKMIP3 1.14907646658204 0.00223851276627537 0.0058709407432582 31.9600592131971 46.323958338033 39.2824560015982 14.1873427290479 15.748191400928 23.5696206756781 up 30.0 45.0 37.0 15.0 19.0 22.0

ACOXL 4.92569672152171 0.0022447717957193 0.00588644492561686 2.13067061421314 3.0882638892022 10.6168800004319 0 0 0 up 2.0 3.0 10.0 0.0 0.0 0.0

LOC101927136 -4.80829160910667 0.00225041174757177 0.00590032115831385 0 0 0 3.78329139441278 9.11737396895832 3.21403918304702 down 0.0 0.0 0.0 4.0 11.0 3.0

CCNI2 1.81935014486951 0.00226414606013586 0.00593357584447733 23.4373767563445 15.441319446011 16.9870080006911 6.62075994022236 5.80196525297347 3.21403918304702 up 22.0 15.0 16.0 7.0 7.0 3.0

LRRC75B -1.04642390567734 0.00230291886432813 0.00602679523585951 29.8293885989839 21.6178472244154 15.9253200006479 41.6162053385406 47.244574202784 50.3532805344032 down 28.0 21.0 15.0 44.0 57.0 47.0

CNGA4 3.4808424182479 0.00231078062371419 0.00604550173987929 12.7840236852788 13.3824768532095 4.24675200017278 0 2.48655653698863 0 up 12.0 13.0 4.0 0.0 3.0 0.0

WBP2NL 1.31197317398665 0.00231204780108304 0.00604788291388923 37.2867357487299 38.0885879668271 20.1720720008207 13.2415198804447 12.4327826849432 12.8561567321881 up 35.0 37.0 19.0 14.0 15.0 12.0

LOC101928797 4.90947427943417 0.00235183512789783 0.0061415272185277 3.19600592131971 10.2942129640073 2.12337600008639 0 0 0 up 3.0 10.0 2.0 0.0 0.0 0.0

HNF4A -4.35824226210257 0.0023671159703853 0.00617762202100246 0 1.02942129640073 0 5.67493709161917 9.94622614795453 7.49942476044304 down 0.0 1.0 0.0 6.0 12.0 7.0

FAM133A -4.35238690214104 0.00239508133416052 0.00624387162214208 1.06533530710657 0 0 6.62075994022236 9.94622614795453 6.42807836609403 down 1.0 0.0 0.0 7.0 12.0 6.0

LINC01119 2.1845771412423 0.00242436507106992 0.00631438258055418 13.8493589923854 18.5295833352132 19.1103840007775 9.45822848603194 0.82885217899621 1.07134639434901 up 13.0 18.0 18.0 10.0 1.0 1.0

TMEM61 1.44697235358291 0.00243342721908259 0.00633506333045322 22.3720414492379 27.7943750028198 27.6038880011231 7.56658278882556 7.45966961096589 13.9275031265371 up 21.0 27.0 26.0 8.0 9.0 13.0

DBNDD2 1.39801044327955 0.00243611525614791 0.00633876310956157 28.7640532918774 20.5884259280146 33.9740160013822 9.45822848603194 13.2616348639394 8.57077115479204 up 27.0 20.0 32.0 10.0 16.0 8.0

PROSER2-AS1 1.53453230239689 0.00245375237596289 0.00637719626400911 22.3720414492379 30.882638892022 19.1103840007775 8.51240563742875 10.7750783269507 5.35673197174503 up 21.0 30.0 18.0 9.0 13.0 5.0

SPATA31E1 -1.56040634314827 0.00246041559731828 0.00639353278395024 10.6533530710657 4.11768518560293 9.55519200038875 20.8081026692703 24.0367131908901 26.7836598587251 down 10.0 4.0 9.0 22.0 29.0 25.0

LOC101928539 1.97586616264771 0.00246745295018948 0.00640690572903277 18.1107002208117 17.5001620388124 14.8636320006047 2.83746854580958 7.45966961096589 2.14269278869801 up 17.0 17.0 14.0 3.0 9.0 2.0

EGFLAM 4.82097667871979 0.00249072838485692 0.00646041017294077 3.19600592131971 5.14710648200366 6.37012800025917 0 0 0 up 3.0 5.0 6.0 0.0 0.0 0.0

KRT6A -4.74921611669978 0.00250923132577091 0.0065034239073683 0 0 0 3.78329139441278 7.45966961096589 4.28538557739602 down 0.0 0.0 0.0 4.0 9.0 4.0

LINC00668 4.436699644382 0.00252670504547083 0.00654270612508037 4.26134122842627 12.3530555568088 5.30844000021597 0 0.82885217899621 0 up 4.0 12.0 5.0 0.0 1.0 0.0

AOC1 4.92550332653854 0.00254114057224924 0.0065760649700431 10.6533530710657 4.11768518560293 1.06168800004319 0 0 0 up 10.0 4.0 1.0 0.0 0.0 0.0

SLC5A2 1.33423969753243 0.00255858420946235 0.00661918395056387 20.2413708350248 45.2945370416322 35.0357040014254 17.0248112748575 8.28852178996211 14.9988495208861 up 19.0 44.0 33.0 18.0 10.0 14.0

CCDC181 1.21138525759547 0.00256371089028882 0.00663042175289964 43.6787475913693 38.0885879668271 25.4805120010367 18.9164569720639 11.6039305059469 16.0701959152351 up 41.0 37.0 24.0 20.0 14.0 15.0

MC5R 3.42698448589274 0.00256639651095358 0.0066359403276082 8.52268245685255 14.4118981496103 6.37012800025917 0 2.48655653698863 0 up 8.0 14.0 6.0 0.0 3.0 0.0

PWRN4 3.34500128495659 0.00261509816490728 0.00675404221886406 6.39201184263941 9.26479166760659 12.7402560005183 1.89164569720639 0.82885217899621 0 up 6.0 9.0 12.0 2.0 1.0 0.0

LINGO1 -1.20918795503166 0.002618755041366 0.00676245585377135 8.52268245685255 20.5884259280146 19.1103840007775 44.4536738843501 33.9829393388446 33.2117382248192 down 8.0 20.0 18.0 47.0 41.0 31.0

ABCA17P 2.00797141532108 0.00262828304739839 0.00678499159142467 19.1760355279182 13.3824768532095 18.0486960007343 2.83746854580958 1.65770435799242 8.57077115479204 up 18.0 13.0 17.0 3.0 2.0 8.0

KATNAL2 1.05458823936343 0.002629859053236 0.00678802565317962 55.3974359695416 83.3831250084593 45.6525840018574 47.2911424301597 18.2347479379166 23.5696206756781 up 52.0 81.0 43.0 50.0 22.0 22.0

MAPK15 -1.19216203457236 0.00263449915131449 0.00679793078800722 9.58801776395912 17.5001620388124 21.2337600008639 40.6703824899374 31.496382801856 38.5684701965642 down 9.0 17.0 20.0 43.0 38.0 36.0

OLFM1 -2.42885171291132 0.00264236871592084 0.00681616050673048 3.19600592131971 1.02942129640073 3.18506400012958 18.9164569720639 9.11737396895832 11.7848103378391 down 3.0 1.0 3.0 20.0 11.0 11.0

LINC01094 -1.92731121205128 0.00267139434913406 0.00688264947138114 9.58801776395912 1.02942129640073 5.30844000021597 28.3746854580958 19.8924522959091 11.7848103378391 down 9.0 1.0 5.0 30.0 24.0 11.0

TRIM17 1.08645806166946 0.00270590564990259 0.00696097823984052 57.5281065837547 53.5299074128381 50.9610240020733 9.45822848603194 30.6675306228598 36.4257774078662 up 54.0 52.0 48.0 10.0 37.0 34.0

ST6GALNAC3 2.98191806526898 0.00271187926090893 0.00697211028681094 41.5480769771562 29.8532175956212 54.1460880022029 7.56658278882556 0 8.57077115479204 up 39.0 29.0 51.0 8.0 0.0 8.0

C9orf170 4.82068685351885 0.00276340792828756 0.0070916725201017 2.13067061421314 5.14710648200366 7.43181600030236 0 0 0 up 2.0 5.0 7.0 0.0 0.0 0.0

SPATA41 3.33565868141174 0.00277513673080528 0.00711961475387572 7.45734714974598 5.14710648200366 15.9253200006479 0.945822848603194 0.82885217899621 1.07134639434901 up 7.0 5.0 15.0 1.0 1.0 1.0

BEX5 3.118139357426 0.00278191325752795 0.00713483884671059 4.26134122842627 7.20594907480513 21.2337600008639 0 1.65770435799242 2.14269278869801 up 4.0 7.0 20.0 0.0 2.0 2.0

LOC646214 1.00962792377341 0.00279118028203581 0.00715319122204034 63.9201184263941 39.1180092632278 43.529208001771 19.8622798206671 29.0098262648674 23.5696206756781 up 60.0 38.0 41.0 21.0 35.0 22.0

KAZALD1 1.16825161072103 0.0028050823560529 0.00718555800870046 25.5680473705576 48.3828009308344 38.220768001555 15.1331655776511 15.748191400928 19.2842350982821 up 24.0 47.0 36.0 16.0 19.0 18.0

SLC6A9 -1.30568012463814 0.0028113943473206 0.00719846134274652 12.7840236852788 8.23537037120586 18.0486960007343 40.6703824899374 24.8655653698863 31.0690454361212 down 12.0 8.0 17.0 43.0 30.0 29.0

GJB2 1.30435518088613 0.00282576155606747 0.00723087624770135 40.4827416700496 22.6472685208161 30.7889520012526 13.2415198804447 15.748191400928 8.57077115479204 up 38.0 22.0 29.0 14.0 19.0 8.0

LOC101929427 4.82316592141901 0.00285905920190264 0.00730945712046948 2.13067061421314 4.11768518560293 8.49350400034555 0 0 0 up 2.0 4.0 8.0 0.0 0.0 0.0

LOC400043 1.98760497564314 0.00287563804782225 0.00734962409929222 14.914694299492 27.7943750028198 15.9253200006479 10.4040513346351 4.14426089498105 0 up 14.0 27.0 15.0 11.0 5.0 0.0

GOLGA8O 1.70870743532315 0.00287771909450467 0.00735272420224872 14.914694299492 18.5295833352132 25.4805120010367 4.72911424301597 4.97311307397726 8.57077115479204 up 14.0 18.0 24.0 5.0 6.0 8.0

ATP8B5P 1.80381939195415 0.00290631427687024 0.00742242783810972 15.9800296065985 20.5884259280146 18.0486960007343 3.78329139441278 8.28852178996211 3.21403918304702 up 15.0 20.0 17.0 4.0 10.0 3.0

SGMS1-AS1 1.25517302897047 0.00292107856616884 0.00745451505135586 23.4373767563445 32.9414814848234 45.6525840018574 16.0789884262543 9.94622614795453 17.1415423095841 up 22.0 32.0 43.0 17.0 12.0 16.0

LOC339260 4.8137662339358 0.00293635210606364 0.00749010761582864 4.26134122842627 8.23537037120586 2.12337600008639 0 0 0 up 4.0 8.0 2.0 0.0 0.0 0.0

ENTPD8 -1.53346418439935 0.00294575556827417 0.00750844106629017 7.45734714974598 8.23537037120586 7.43181600030236 21.7539255178735 20.7213044749053 24.6409670700271 down 7.0 8.0 7.0 23.0 25.0 23.0

LINC01537 -4.68699953089647 0.0029707218812893 0.00756524784561647 0 0 0 3.78329139441278 5.80196525297347 5.35673197174503 down 0.0 0.0 0.0 4.0 7.0 5.0

ZNF483 1.34595225365097 0.00301700770527258 0.00767395522889267 33.0253945203036 23.6766898172168 24.4188240009935 10.4040513346351 10.7750783269507 10.7134639434901 up 31.0 23.0 23.0 11.0 13.0 10.0

HABP2 2.46566242531915 0.00303440078610376 0.00771118351584125 9.58801776395912 17.5001620388124 9.55519200038875 3.78329139441278 0.82885217899621 2.14269278869801 up 9.0 17.0 9.0 4.0 1.0 2.0

MCMDC2 -1.86676337428744 0.0030624012001496 0.0077776726132735 3.19600592131971 3.0882638892022 8.49350400034555 18.9164569720639 20.7213044749053 13.9275031265371 down 3.0 3.0 8.0 20.0 25.0 13.0

CHRDL1 -3.21700851099832 0.00307421635436743 0.00779832660122556 2.13067061421314 0 1.06168800004319 9.45822848603194 8.28852178996211 11.7848103378391 down 2.0 0.0 1.0 10.0 10.0 11.0

SMIM10 1.78020020679212 0.0031399642252119 0.007951725293217 29.8293885989839 17.5001620388124 10.6168800004319 5.67493709161917 5.80196525297347 5.35673197174503 up 28.0 17.0 10.0 6.0 7.0 5.0

TIMD4 1.55651566851018 0.00317613156930173 0.00803759617328524 25.5680473705576 16.4707407424117 24.4188240009935 4.72911424301597 8.28852178996211 9.64211754914105 up 24.0 16.0 23.0 5.0 10.0 9.0

ACOT4 1.43825567793602 0.00317795729173946 0.00804101516386354 20.2413708350248 26.764953706419 27.6038880011231 11.3498741832383 5.80196525297347 10.7134639434901 up 19.0 26.0 26.0 12.0 7.0 10.0

NUTM2A 1.00212036203031 0.00320909884804997 0.00811375141581589 39.417406362943 61.7652777840439 50.9610240020733 35.9412682469214 20.7213044749053 19.2842350982821 up 37.0 60.0 48.0 38.0 25.0 18.0

IGF2BP1 2.96777648960729 0.0032428948057885 0.00819068678036437 7.45734714974598 10.2942129640073 11.6785680004751 0 1.65770435799242 2.14269278869801 up 7.0 10.0 11.0 0.0 2.0 2.0

FOXG1-AS1 -4.70568110628707 0.00325997167394652 0.00823254623682977 0 0 0 1.89164569720639 5.80196525297347 7.49942476044304 down 0.0 0.0 0.0 2.0 7.0 7.0

HOXC10 1.94539409454544 0.00329330026441265 0.00831051787265758 15.9800296065985 9.26479166760659 28.6655760011662 7.56658278882556 4.14426089498105 2.14269278869801 up 15.0 9.0 27.0 8.0 5.0 2.0

EPHA7 -2.7523539813591 0.00331060823223715 0.00835170565213662 4.26134122842627 0 1.06168800004319 8.51240563742875 18.2347479379166 8.57077115479204 down 4.0 0.0 1.0 9.0 22.0 8.0

MMP28 -1.01529575660603 0.00334433663308493 0.00842926090826643 18.1107002208117 19.5590046316139 37.1590800015118 50.1286109759693 59.6773568877272 40.7111629852622 down 17.0 19.0 35.0 53.0 72.0 38.0

POF1B -4.76093291513921 0.00338166611699356 0.008515746101066 0 0 0 7.56658278882556 0.82885217899621 7.49942476044304 down 0.0 0.0 0.0 8.0 1.0 7.0

NBPF4 4.2997879290889 0.00338552479591183 0.00852166271066068 6.39201184263941 7.20594907480513 6.37012800025917 0.945822848603194 0 0 up 6.0 7.0 6.0 1.0 0.0 0.0

GAP43 4.83133596021203 0.00338897793854229 0.00852908724987467 4.26134122842627 1.02942129640073 9.55519200038875 0 0 0 up 4.0 1.0 9.0 0.0 0.0 0.0

CACNG6 -3.26695411631746 0.00340260789953052 0.00855963728269978 1.06533530710657 2.05884259280146 0 6.62075994022236 5.80196525297347 18.2128887039331 down 1.0 2.0 0.0 7.0 7.0 17.0

CTD-2201I18.1 -4.7236044837297 0.00341559370490265 0.00858969081963346 0 0 0 9.45822848603194 1.65770435799242 4.28538557739602 down 0.0 0.0 0.0 10.0 2.0 4.0

CALCRL 1.76706777188688 0.00346855745085105 0.00871272725659286 17.0453649137051 18.5295833352132 15.9253200006479 4.72911424301597 4.14426089498105 6.42807836609403 up 16.0 18.0 15.0 5.0 5.0 6.0

SLC16A10 1.16968092421984 0.0034686323859777 0.00871272725659286 45.8094182055824 32.9414814848234 38.220768001555 17.0248112748575 9.11737396895832 26.7836598587251 up 43.0 32.0 36.0 18.0 11.0 25.0

HMGCLL1 2.77365610926251 0.00349361216531531 0.00876767260450983 17.0453649137051 7.20594907480513 7.43181600030236 0.945822848603194 2.48655653698863 1.07134639434901 up 16.0 7.0 7.0 1.0 3.0 1.0

B4GALNT2 2.19172507314597 0.00350654268296435 0.00879491161525261 14.914694299492 12.3530555568088 11.6785680004751 1.89164569720639 2.48655653698863 4.28538557739602 up 14.0 12.0 11.0 2.0 3.0 4.0

C22orf34 4.71903028857487 0.00353486946888638 0.00885940062182798 5.32667653553284 3.0882638892022 5.30844000021597 0 0 0 up 5.0 3.0 5.0 0.0 0.0 0.0

SLC34A3 1.24162755343616 0.00359149116296315 0.00898893752024716 30.8947239060905 52.5004861164373 21.2337600008639 15.1331655776511 13.2616348639394 16.0701959152351 up 29.0 51.0 20.0 16.0 16.0 15.0

OR2AG2 4.71874393548148 0.00360115038442381 0.00900819366688413 4.26134122842627 3.0882638892022 6.37012800025917 0 0 0 up 4.0 3.0 6.0 0.0 0.0 0.0

LOC100507468 4.71874393548148 0.00360115038442381 0.00900819366688413 4.26134122842627 3.0882638892022 6.37012800025917 0 0 0 up 4.0 3.0 6.0 0.0 0.0 0.0

LRRC4 -1.61992305741625 0.00360179580833671 0.00900847792606288 5.32667653553284 5.14710648200366 10.6168800004319 14.1873427290479 24.8655653698863 25.7123134643761 down 5.0 5.0 10.0 15.0 30.0 24.0

LOC283352 4.71581796029649 0.00363102962344088 0.00907380838349701 3.19600592131971 4.11768518560293 6.37012800025917 0 0 0 up 3.0 4.0 6.0 0.0 0.0 0.0

LANCL3 1.74177816104029 0.00364645188954367 0.00911075147623449 9.58801776395912 19.5590046316139 30.7889520012526 4.72911424301597 5.80196525297347 7.49942476044304 up 9.0 19.0 29.0 5.0 7.0 7.0

GALNT3 1.02331511150168 0.00366612475568361 0.00915202292598629 38.3520710558365 38.0885879668271 43.529208001771 20.8081026692703 19.8924522959091 18.2128887039331 up 36.0 37.0 41.0 22.0 24.0 17.0

EYA1 1.79645484658394 0.00366621178133075 0.00915202292598629 12.7840236852788 19.5590046316139 19.1103840007775 4.72911424301597 6.63081743196968 3.21403918304702 up 12.0 19.0 18.0 5.0 8.0 3.0

ACTN2 2.21721302257864 0.00371135482036975 0.00925380492450156 13.8493589923854 10.2942129640073 14.8636320006047 0.945822848603194 4.14426089498105 3.21403918304702 up 13.0 10.0 14.0 1.0 5.0 3.0

WAS 1.72477986886746 0.00375693279857429 0.00935230589387927 11.7186883781723 24.7061111136176 25.4805120010367 1.89164569720639 8.28852178996211 8.57077115479204 up 11.0 24.0 24.0 2.0 10.0 8.0

ABCC6 1.41773890198029 0.00378163931343064 0.00940551607245853 24.5027120634511 29.8532175956212 18.0486960007343 8.51240563742875 9.94622614795453 8.57077115479204 up 23.0 29.0 17.0 9.0 12.0 8.0

LINC00607 -1.50483248625945 0.00389597551117802 0.00966646347009118 7.45734714974598 10.2942129640073 9.55519200038875 30.2663311553022 34.8117915178408 11.7848103378391 down 7.0 10.0 9.0 32.0 42.0 11.0

GFAP 1.05917781266851 0.00391615437118411 0.009710136862977 44.7440828984759 37.0591666704264 32.912328001339 17.9706341234607 20.7213044749053 16.0701959152351 up 42.0 36.0 31.0 19.0 25.0 15.0

C11orf21 4.711105988466 0.00393399145961318 0.0097486564256652 5.32667653553284 6.17652777840439 2.12337600008639 0 0 0 up 5.0 6.0 2.0 0.0 0.0 0.0

IL21-AS1 -1.53767665177222 0.00393488752077242 0.00974945072427742 14.914694299492 3.0882638892022 8.49350400034555 34.9954453983182 18.2347479379166 23.5696206756781 down 14.0 3.0 8.0 37.0 22.0 22.0

SLC7A11-AS1 -2.24067889969816 0.00398086204266709 0.00985471328405385 2.13067061421314 1.02942129640073 5.30844000021597 13.2415198804447 15.748191400928 10.7134639434901 down 2.0 1.0 5.0 14.0 19.0 10.0

EMX1 2.54489334828275 0.00401968616420609 0.00994355777185327 8.52268245685255 15.441319446011 8.49350400034555 1.89164569720639 2.48655653698863 1.07134639434901 up 8.0 15.0 8.0 2.0 3.0 1.0

CYP46A1 1.21107835525281 0.00402088813170383 0.00994390343034898 23.4373767563445 37.0591666704264 40.3441440016414 17.9706341234607 16.5770435799242 8.57077115479204 up 22.0 36.0 38.0 19.0 20.0 8.0

NEUROD2 -2.84701340000259 0.00408444330042173 0.0100880654396694 2.13067061421314 0 2.12337600008639 10.4040513346351 8.28852178996211 11.7848103378391 down 2.0 0.0 2.0 11.0 10.0 11.0

LOC101928324 4.70812165204769 0.00408524964085785 0.0100880654396694 4.26134122842627 7.20594907480513 2.12337600008639 0 0 0 up 4.0 7.0 2.0 0.0 0.0 0.0

SOX18 -1.43061864261811 0.00410581946195673 0.010132951877265 4.26134122842627 16.4707407424117 9.55519200038875 22.6997483664767 23.2078610118939 36.4257774078662 down 4.0 16.0 9.0 24.0 28.0 34.0

CCM2L -1.9616567927746 0.00411351531343939 0.0101489876798228 2.13067061421314 4.11768518560293 5.30844000021597 13.2415198804447 15.748191400928 16.0701959152351 down 2.0 4.0 5.0 14.0 19.0 15.0
[truncated: 55,578 more chars]
