# Supplementary material for: CYP27B1 Downregulation: A New Molecular Mechanism Regulating EZH2 in Ovarian Cancer Tumorigenicity
Source: Front Cell Dev Biol. 2020 Oct 14;8:561804. doi: 10.3389/fcell.2020.561804 (PMC7591459; doi:10.3389/fcell.2020.561804)

**Supplementary S4.** Changes of biological behaviors and relative genes expression in rescue experiment. a. Western blotting results; b. Transwell migration and invasion assay; c, d. Cell growth curve. * p < 0.05, ** p < 0.01.


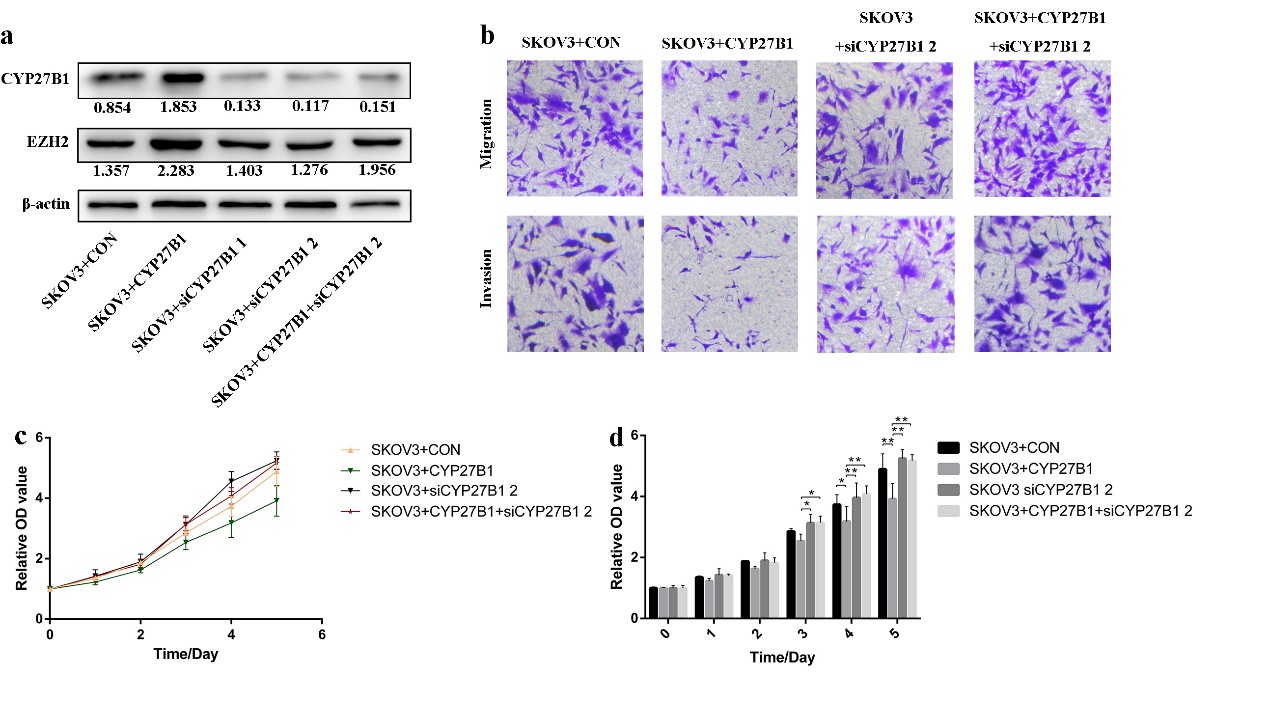

Supplement: Supplementary file 5 [file Table_4.DOCX]
