# Supplementary material for: CYP27B1 Downregulation: A New Molecular Mechanism Regulating EZH2 in Ovarian Cancer Tumorigenicity
Source: Front Cell Dev Biol. 2020 Oct 14;8:561804. doi: 10.3389/fcell.2020.561804 (PMC7591459; doi:10.3389/fcell.2020.561804)
Supplement: Supplementary file 6 [file Table_5.docx]

**Supplementary S5.** Characteristics of ovarian cancer patients

| **Characteristic** | **No.** | **%** |
| --- | --- | --- |
| **Age (y), median (range)** | 53(28-71) | 100% |
| **Stage** |  |  |
| I–II | 33 | 20.6% |
| III | 85 | 53.1% |
| IV | 42 | 26.3% |
| **Histological subtype** |  |  |
| Serous | 160 | 100% |
| **Differentiation grade** |  |  |
| G1 | 28 | 17.5% |
| G2 | 55 | 34.4% |
| G3 | 67 | 41.9% |
| Unknown | 10 | 6.2% |
| **Residual disease** |  |  |
| optimal | 115 | 71.9% |
| suboptimal | 26 | 16.3% |
| unknown | 19 | 11.8% |
