# Supplementary material for: CYP27B1 Downregulation: A New Molecular Mechanism Regulating EZH2 in Ovarian Cancer Tumorigenicity
Source: Front Cell Dev Biol. 2020 Oct 14;8:561804. doi: 10.3389/fcell.2020.561804 (PMC7591459; doi:10.3389/fcell.2020.561804)

**Supplementary S6.** OS and disease-free survival (DFS) curves for patients with ovarian cancer (N=160) stratified according to the EZH2 and CYP27B1 subtype (a, b).


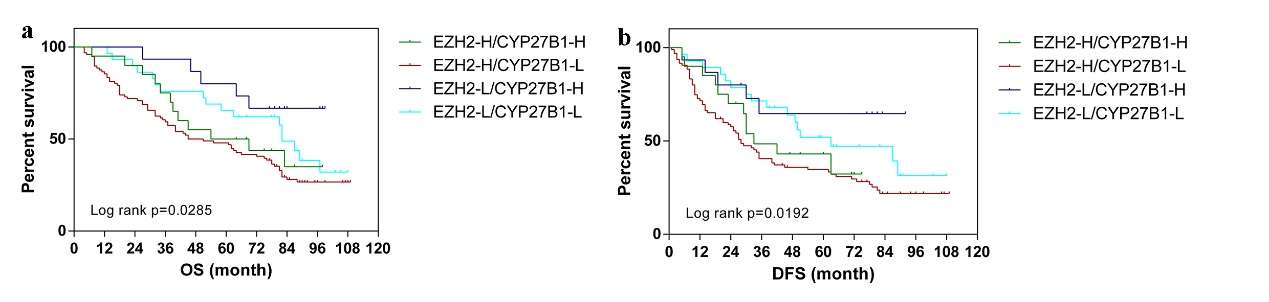

Supplement: Supplementary file 7 [file Table_6.DOCX]
